# Genome-wide association study of body fat distribution traits in Hispanics/Latinos from the HCHS/SOL

**DOI:** 10.1093/hmg/ddab166

**Published:** 2021-06-24

**Authors:** Anne E Justice, Kristin Young, Stephanie M Gogarten, Tamar Sofer, Misa Graff, Shelly Ann M Love, Yujie Wang, Yann C Klimentidis, Miguel Cruz, Xiuqing Guo, Fernando Hartwig, Lauren Petty, Jie Yao, Matthew A Allison, Jennifer E Below, Thomas A Buchanan, Yii-Der Ida Chen, Mark O Goodarzi, Craig Hanis, Heather M Highland, Willa A Hsueh, Eli Ipp, Esteban Parra, Walter Palmas, Leslie J Raffel, Jerome I Rotter, Jingyi Tan, Kent D Taylor, Adan Valladares, Anny H Xiang, Lisa Sánchez-Johnsen, Carmen R Isasi, Kari E North

**Affiliations:** 1 Department of Population Health Sciences, Geisinger Health System, Danville, PA 17822, USA; 2 Department of Epidemiology, University of North Carolina at Chapel Hill, Chapel Hill, NC 27516, USA; 3 Department of Biostatistics, University of Washington, Seattle, WA 98195, USA; 4 Department of Epidemiology and Biostatistics, University of Arizona, Tucson, AZ 85724, USA; 5 Unidad de Investigacion Medica en Bioquimica, Hospital de Especialidades, Centro Medico Nacional Siglo XXI (CMNSXX1)-IMSS, Mexico City 06720, Mexico; 6 The Institute for Translational Genomics and Population Sciences, Department of Pediatrics, The Lundquist Institute for Biomedical Innovation at Harbor-UCLA Medical Center, Torrance, CA 90502, USA; 7 Center for Epidemiological Research, Universidade Federal de Pelotas, Pelotas 96020, Brazil; 8 Division of Genetic Medicine, Department of Medicine, Vanderbilt Genetics Institute, Vanderbilt University Medical Center, Nashville, TN 37232, USA; 9 Division of Preventive Medicine, Department of Family Medicine, University of California San Diego, La Jolla, CA 92093, USA; 10 Department of Medicine, Keck School of Medicine and Diabetes and Obesity Research Institute, University of Southern California, Los Angeles, CA 90033, USA; 11 Division of Endocrinology, Diabetes, and Metabolism, Department of Medicine, Cedars-Sinai Medical Center, Los Angeles, CA 90048, USA; 12 Human Genetics Center, Department of Epidemiology, Human Genetics and Environmental Sciences, School of Public Health, The University of Texas Health Science Center at Houston, Houston, TX 77030, USA; 13 Division of Endocrinology, Diabetes and Metabolism, Department of Medicine, Wexner Medical Center, The Ohio State University, Columbus, OH 43210, USA; 14 Department of Medicine, Endocrinology, Diabetes & Metabolism, The Lundquist Institute for Biomedical Innovation at Harbor-UCLA Medical Center, Torrance, CA 90502, USA; 15 Department of Anthropology, University of Toronto at Mississauga, Mississauga, ON L5L 1C6, Canada; 16 Department of Medicine, Columbia University Medical Center, New York, NY 10032, USA; 17 Department of PediatrIcs, Division of Genetic and Genomic Medicine, University of California, Irvine, CA 92868, USA; 18 Department of Research and Evaluation, Kaiser Permanente Southern California, Pasadena, CA 91101, USA; 19 Department of Family Medicine, Rush University Medical Center, Chicago, IL 60612, USA; 20 Department of Epidemiology & Population Health, Albert Einstein College of Medicine, Bronx, NY 10467, USA; 21 Department of Pediatrics, Albert Einstein College of Medicine, Bronx, NY 10467, USA

## Abstract

Central obesity is a leading health concern with a great burden carried by ethnic minority populations, especially Hispanics/Latinos. Genetic factors contribute to the obesity burden overall and to inter-population differences. We aimed to identify the loci associated with central adiposity measured as waist-to-hip ratio (WHR), waist circumference (WC) and hip circumference (HIP) adjusted for body mass index (adjBMI) by using the Hispanic Community Health Study/Study of Latinos (HCHS/SOL); determine if differences in associations differ by background group within HCHS/SOL and determine whether previously reported associations generalize to HCHS/SOL. Our analyses included 7472 women and 5200 men of mainland (Mexican, Central and South American) and Caribbean (Puerto Rican, Cuban and Dominican) background residing in the USA. We performed genome-wide association analyses stratified and combined across sexes using linear mixed-model regression. We identified 16 variants for waist-to-hip ratio adjusted for body mass index (WHRadjBMI), 22 for waist circumference adjusted for body mass index (WCadjBMI) and 28 for hip circumference adjusted for body mass index (HIPadjBMI), which reached suggestive significance (*P* < 1 × 10^−6^). Many loci exhibited differences in strength of associations by ethnic background and sex. We brought a total of 66 variants forward for validation in cohorts (*N* = 34 161) with participants of Hispanic/Latino, African and European descent. We confirmed four novel loci (*P* < 0.05 and consistent direction of effect, and *P* < 5 × 10^−8^ after meta-analysis), including two for WHRadjBMI (rs13301996, rs79478137); one for WCadjBMI (rs3168072) and one for HIPadjBMI (rs28692724). Also, we generalized previously reported associations to HCHS/SOL, (8 for WHRadjBMI, 10 for WCadjBMI and 12 for HIPadjBMI). Our study highlights the importance of large-scale genomic studies in ancestrally diverse Hispanic/Latino populations for identifying and characterizing central obesity susceptibility that may be ancestry-specific.

## Introduction

Obesity, and especially central obesity, is a leading risk factor for metabolic and cardiovascular diseases (CVDs), with the greatest burden carried by minority populations (1–4), particularly Hispanic/Latino Americans and African Americans (5). Emerging evidence suggests that genetic factors may contribute not only to the obesity burden overall, explaining 40–70% of the inter-individual variation (6), but also to population-specific differences in obesity susceptibility (7–12). For example, although a majority of the >1000 genome-wide association study (GWAS)-identified obesity [body mass index (BMI), waist-to-hip ratio (WHR), waist circumference (WC), hip circumference (HIP) and body fat percentage] loci generalize across populations (13–20), recent studies in populations of Asian (19,20) and African (16,21) ancestry have revealed a number of novel and population-specific loci. These observations highlight the importance of large-scale genomic studies in ancestrally diverse populations, including Hispanic/Latinos, to identify obesity-susceptibility loci, and more specifically, alleles that are ancestry-specific and may thus partly explain disparities. However, no large-scale GWAS for any obesity-related traits has been performed in Hispanic/Latino populations despite their increased prevalence of obesity.

While obesity is commonly assessed by BMI, measures of central adiposity, such as WHR and WC, are predictors of increased cardiometabolic risk independent of BMI (22–25). Here, we consider three measures of central obesity: WHR, WC and HIP after accounting for overall body size, measured as BMI [waist-to-hip ratio adjusted for body mass index (WHRadjBMI), waist circumference adjusted for body mass index (WCadjBMI) and hip circumference adjusted for body mass index (HIPadjBMI)]. Larger WHR indicates higher visceral fat and is associated with increased risk for type 2 diabetes (T2D) and CVD (26–28), while smaller WHR indicates a proportionately greater fat accumulation around the hips and is associated with lower risk for T2D, hypertension and dyslipidemia (29). Previous GWAS have identified WHR, WC and HIP loci, which are enriched for association with other cardiometabolic traits and suggested that different fat distribution patterns can have distinct genetic underpinnings (30–32). Identifying genetic risk variants across these traits in Hispanic/Latinos may provide insights into these mechanisms and highlight population-specific variants that increase susceptibility to obesity in specific groups.

We aimed to: (1) identify novel genetic loci associated with central obesity, measured here as WHRadjBMI, WCadjBMI and HIPadjBMI, in Hispanics/Latinos; (2) determine if differences in genetic associations by background group (mainland or Caribbean) and sex exist in Hispanic Community Health Study/Study of Latinos (HCHS/SOL) and (3) assess generalization of central adiposity-associated loci, discovered in European, African and multi-ethnic studies, to Hispanics/Latinos.

## Results

### Discovery

**Figure 1:**
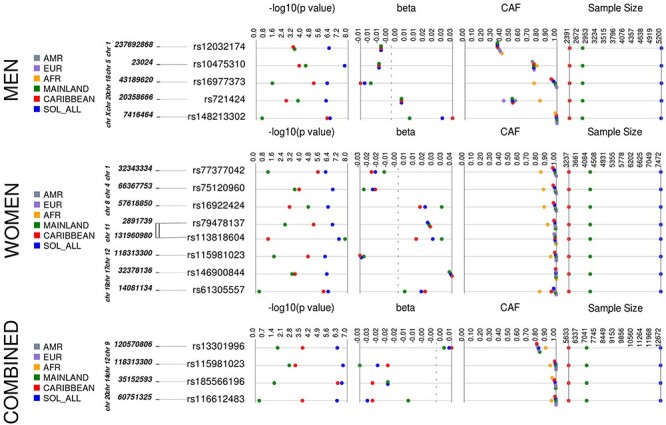
WHRadjBMI Synthesis View plot that shows −log10 *P*-values, beta (effect estimate), effect/coded allele frequency (CAF) and sample size across analysis samples for all loci that reached suggestive significance in one or more of our discovery strata. This chart also shows the CAF of each of our top loci by background group and by 1000 genomes reference panel. European, EUR; Latin American, AMR; African, AFR.

**Figure 2:**
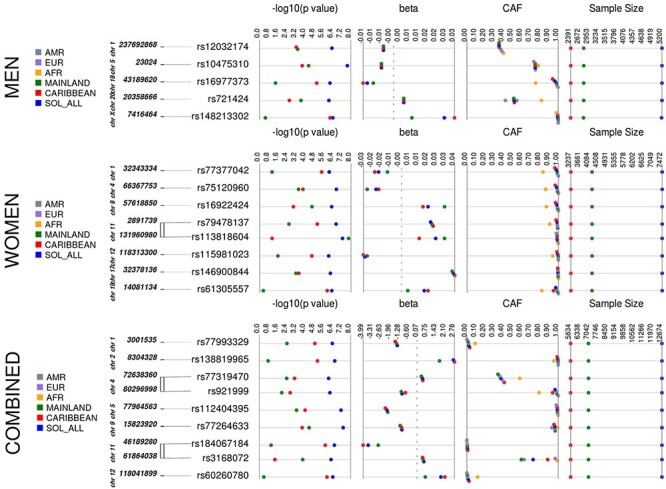
WCadjBMI Synthesis View plot that shows −log10 *P*-values, beta (effect estimate), effect/CAF, and sample size across analysis samples for all loci that reached suggestive significance in one or more of our discovery strata. This chart also shows the CAF of each of our top loci by background group and by 1000 genomes reference panel. European, EUR; Latin American, AMR; African, AFR.

**Figure 3:**
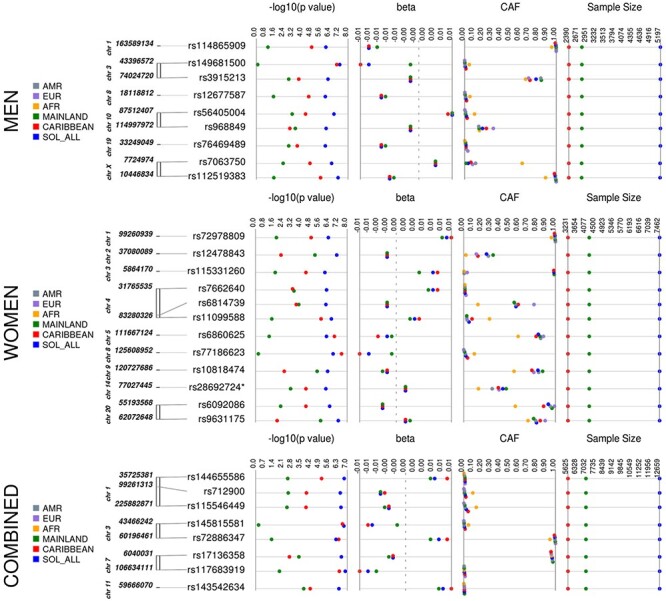
HIPadjBMI Synthesis View plot that shows the −log10 *P*-values, beta (effect estimate), effect/CAF and sample size across analysis samples for all loci that reached suggestive significance in one or more of our discovery strata. This chart also shows the CAF of each of our top loci by background group and by 1000 genomes reference panel. European, EUR; Latin American, AMR; African, AFR.

We identified 16 loci for WHRadjBMI, 22 for WCadjBMI and 28 for HIPadjBMI, which exhibited suggestive evidence of association in the HCHS/SOL (*N* = 12 472, 58% women, Supplementary Material, Table S1) in at least one stratum (Table 1, Figs 1–3, Supplementary Material, Tables S2–S4, Supplementary Material, Figs S1–S21). For WHRadjBMI, we identified four loci that reach suggestive significance (*P* < 1 × 10^−6^) in the combined sexes, including rs12435790 near *KIAA0391*, which is within a previously reported WHRadjBMI locus [+/−500 Kb from tag single nucleotide polymorphism (SNP)] (33). We also identified five loci for men only, including one reaching genome-wide significance (GWS, *P* < 5 × 10^−8^). A total of eight suggestive loci were identified in the women-only analyses, including one, rs115981023 in *TAOK3*, which also reached suggestive significance in the combined sexes analysis and identified rs79478137 in solute carrier family 22 (organic cation transporter), member 18 antisense (*SLC22A18AS*) near a previously implicated WHRadjBMI locus (34). For WCadjBMI, we identified nine loci, including one GWS locus in the combined sexes; 11 for men only, including two SNPs that reach GWS, and two for women only. Of the WCadjBMI loci identified, two were nearby previously reported WCadjBMI loci, rs6809759 near *PROK2* (men-only) (14,15,17) and rs77319470 near *ADAMTS3* (sexes-combined) (15,17,35). For HIPadjBMI, we identified eight loci that reach *P* < 1 × 10^−6^ for the combined sexes; nine for men only, including one in a locus that reached suggestive significance for the combined sexes as well (near *ANO10*), and 12 for women only, including one SNP in a locus that reached suggestive significance for the combined sexes as well (near *LPPR4*). Of the WCadjBMI loci, rs10818474 near *MEGF9* was within 500 Kb of a recently reported WHRadjBMI association in women (14).

**Table 1:** Summary of association results for all loci that passed replication criteria

Stratum	dbSNPID	CHR	POS (GRCh38)	Nearest gene	EAF	Other allele	Stage	EAF	Beta	SE	*P*	*N*
**WHRadjBMI**												
AA^b^-combined	rs13301996	9	120 570 806	*CDK5RAP2*	T	G	SOL	0.8080	0.0050	0.0010	5.69E-07	12 672
							Replication	0.8720	0.0036	0.0014	1.10E-02	12 496
							SOL + replication	0.8295	0.0045	0.0008	**2.88E-08**	25 168
HL^c^−women	rs79478137	11	2 891 739	*SLC22A18AS*	T	C	SOL	0.0150	-0.0230	0.0040	2.03E-07	7472
							Replication	0.0169	-0.0116	0.0054	3.12E-02	6582
							SOL + replication	0.0157	-0.0189	0.0032	**3.64E-09**	14 054
**WCadjBMI**												
EUR^d^-combined	rs3168072	11	61 864 038	*FADS2*	A	T	SOL	0.7250	0.5140	0.1020	5.28E-07	12 674
							Replication	0.9750	2.0132	0.6323	1.45E-03	8845
							SOL + replication	0.7313	0.5520	0.1007	**4.21E-08**	21 519
**HIPadjBMI**												
EUR^d^-women	rs28692724^e^	14	77 027 445	*IRF2BPL*	T	C	SOL	0.4250	0.0020	0.0004	7.32E-07	7462
							Replication	0.303	0.789	0.305	9.62E-03	4678
							SOL + replication	0.3781	5.4900^a^		**4.02E-08**	12 140

EAF, estimated allele frequency; CHR, chromosome; pos, position; SE, standard error. Genome-wide significant (P<5×10^−8^) values are highlighted in bold.

^a^For rs28692724, SOL analyses were performed on log10-transformed HIP, while replication analyses in European descent population used untransformed hip measurements. In the SOL + replication analyses, an *z*-score is provided instead of a beta.

^b^AA replication samples included: ARIC Study, Multi-Ethnic Study of Atherosclerosis (MESA) Study, Women’s Health Initiative (WHI) Study.

^c^Hispanic Latino (HL) replication samples included: Genetics of Latinos Diabetic Retinopathy (GOLDR), HCHS/SOL, Mexican–American Hypertension Study (HTN), MACAD, MESA, Mexico-City, 1982 Pelotas Birth Cohort (PELOTAS), Starr County Health Studies (STARR), WHI.

^d^European American (EA) replication samples included: ARIC.

^e^rs28692724 is <500 Kb from a previously reported SNP nominally associated with WHRadjBMI (PMID: 28552196).

### Association differences by genetic ancestry

All of the top loci were directionally consistent in each background group, yet many of the loci exhibited effect heterogeneity by background group (Table 2, Figs 1–3, Supplementary Material, Tables S5–S7), as exhibited by moderate-to-high *I*^2^ values [*I*-squared heterogeneity (ISQ) > 65%) and/or significant interaction across background groups (*P*_diff_ < 0.05). For example, rs113818604 (β = 0.0269, *P* = 5.47 × 10^−8^), *I*^2^ = 78.5%, *P*_diff_ = 0.38) in *NTM* is significantly associated with WHRadjBMI in women from the mainland background groups [*N* = 4220, minor allele frequency (MAF) = 0.014, β = 0.0343, *P* = 1.63 × 10^−8^] but not in women from Caribbean background groups (*N* = 3238, MAF = 0.013, β = 0.0144, *P* = 0.08) (Supplementary Material, Table S5). Also, for the women-only primary analysis, the rs77186623 in *LOC105375745* locus associated with HIPadjBMI (β = −0.006, *P* = 1.74 × 10^−7^) exhibited nominally significant interaction by background group (*I*^2^ = 55.3%, *P*_diff_ = 0.042) and was GWS in the Caribbean group (*N* = 3231, MAF = 0.041, β = −0.0078, *P* = 3.05 × 10^−8^) but not significant in the mainland group (*N* = 4216, MAF = 0.008, β = −0.0015, *P* = 0.567, Supplementary Material, Table S7). Additional examples that cannot be explained because of power (i.e. MAF and sample size are similar) for WHRadjBMI include rs77377042 near *MARCKSL1* and rs61305557 in *C19orf67* for women and rs16977373 near *RIT2* for men; for WCadjBMI in women-only, these include rs76842062 in *MAP4K4* and rs76941364 near *COBL*; and for HIPadjBMI, these include rs6860625 near *NREP* for women and rs145815581 in *ANO10* for the combined sexes.

**Table 2:** Summary of top association results in SOL background group analyses

Stratum	dnSNPID	CHR	POS (GRCh38)	Nearest gene	EAF	Other allele	Background group	EAF	Beta	SE	*P*	*N*	*P*diff	EAF			
														AFR	EUR	AMR	
**WHRadjBMI**																	
AA-combined	rs13301996	9	120 570 806	*CDK5RAP2*	T	G	SOL	0.8080	0.0050	0.0010	5.69E-07	12 672	1.88E-01	0.886	0.809	0.794	
							Mainland	0.8220	0.0034	0.0014	1.95E-02	7013					
							Caribbean	0.7890	0.0061	0.0017	2.52E-04	5633					
HL-women	rs79478137	11	2 891 739	*SLC22A18AS*	T	C	SOL	0.0150	-0.0230	0.0040	2.03E-07	7472	3.97E-01	0.095	0.011	0.010	
							Mainland	0.0080	-0.0241	0.0080	2.72E-03	4220					
							Caribbean	0.0250	-0.0250	0.0056	8.87E-06	3238					
**WCadjBMI**																	
EUR-combined	rs3168072	11	61 864 038	*FADS2*	A	T	SOL	0.7250	0.5140	0.1020	5.28E-07	12 674	3.94E-01	0.990	0.967	0.630	
							Mainland	0.5969	0.4552	0.1204	1.57E-04	7013					
							Caribbean	0.8846	0.4170	0.2075	4.45E-02	5635					
**HIPadjBMI**																	
EUR-Women	rs28692724^a^	14	77 027 445	*IRF2BPL*	T	C	SOL	0.4250	0.0020	0.0004	7.32E-07	7462	2.31E-01	0.160	0.384	0.310	
							Mainland	0.462	0.0017	0.001	8.70E-04	4216					
							Caribbean	0.3765	0.0025	0.0006	4.02E-05	3232					

^a^EAF for reference population obtained from ExAC; all other estimated EAFs are from 1000 Genomes Project Phase 3.

For other loci, allele frequency and linkage disequilibrium (LD) differences across Hispanic/Latino populations likely contributed to observed differences in the magnitude of effect and significance levels (Supplementary Material, Table S8). For example, while the magnitude of effect for the rs115981023 *TAOK3* association with WHRadjBMI in women (β = −0.029, *P* = 8.88x10^−7^, *I*^2^ = 0, *P*_diff_ = 0.391) was similar across background groups, the *P*-value was far more significant in the Caribbean background group (MAF = 0.016, β = −0.030, *P* = 2.72 × 10^−5^) when compared with the mainland (MAF = 0.003, β = −0.027, *P* = 0.025), likely because of the higher MAF in the Caribbean group. Of note, the minor allele at this SNP is more common in the 1000 Genomes AFR compared with the EUR and AMR reference samples (Supplementary Material, Table S5), and the local ancestry of participants at this locus indicate that those with African ancestry exhibit the highest MAF (Supplementary Material, Table S8). Additional loci where the significance level differences between Caribbean and mainland background groups appear to be driven by increased MAF owing to African ancestry in Caribbean populations include the *SLC22A18AS* and *CDH4* loci for WHRadjBMI; *LOC102723448*, *FZD7*, *WSB2* and *ACTRT2* loci for WCadjBMI; and *COQ2*, *LPPR4*, *TMEM63A* and *FHIT* loci for HIPadjBMI (Supplementary Material, Tables S5–S8). Rs12478843 in *HEATR5B* (β = −0.002, *P* = 8.2 × 10^−8^, *I*^2^ = 1.7%, *P*_diff_ = 0.385) is more significantly associated with HIPadjBMI in mainland (MAF = 0.320, β = −0.002, *P* = 6.50 × 10^−6^) women when compared with Caribbean (MAF = 0.154, β = −0.002, *P* = 6.03 × 10^−3^), likely reflecting the higher MAF among those from mainland Latin America with greater Native American ancestry (Supplementary Material, Table S8). Similarly, differences in effect magnitude between mainland and Caribbean background groups for the *TAF4* (HIPadjBMI in women) and the *ESRRG* (WCadjBMI in men) loci may also be owing to higher MAF in the mainland group because of a greater proportion of Native American ancestry (Supplementary Material, Tables S6–S8).

### Replication

We brought 66 variants forward for replication in nine cohort studies (*N* up to 34 161), with participants of Hispanic/Latino, African and European descent, and for further examination of replication by ancestral background (Supplementary Material, Tables S1–S4). Our criteria for replication included both nominal evidence of an association (*P* < 0.05), consistent direction of effect between the replication results and the HCHS/SOL discovery results for any ancestry/sex stratum and genome-wide significance (*P* < 5 × 10^−8^) when meta-analyzed together with HCHS/SOL. Based on these criteria, we were able to replicate four novel loci (Table 1) after combining our HCHS/SOL discovery sample with specific ancestry results. For WHRadjBMI in men and women combined, rs13301996 was significant after meta-analyzing HCHS/SOL with the African American replication sample (*P* = 2.88 × 10^−8^). For WHRadjBMI in women only, rs79478137 was GWS after combining HCHS/SOL with the Hispanic/Latino replication sample (*P* = 3.64 × 10^−9^). For WCadjBMI in men and women combined, rs3168072 was significant after combining HCHS/SOL with the European American replication sample (*P* = 4.21 × 10^−8^). For HIPadjBMI in women only, rs28692724 was significant after meta-analyzing HCHS/SOL with the European American replication sample (*P* = 4.02 × 10^−8^).

Of note, for rs13301996, which only replicated in African Americans, we saw a larger effect size in the Caribbean background group compared with the mainland, although this is not a significant difference (Table 2, Supplementary Material, Table S5, Supplementary Material, Fig. S2A). This finding may provide insight into why the variants were more successful upon replication with a particular ancestry. For the remaining loci, there is little difference in effect magnitude between the Caribbean and the mainland background groups, which could explain differences in replication by ancestral group.

### Generalization of previous loci

We examined previously reported association regions from the Genetic Investigation of Anthropometric Traits (GIANT) Consortium (14) to assess generalization to the HCHS/SOL (Supplementary Material, Tables S9–S11, Supplementary Material, Figs S22–S30). To account for the differences in LD between GIANT (primarily European descent populations) and HCHS/SOL (highly admixed Hispanic/Latino populations), we report generalization results based on the lead generalized SNP (the SNP with lowest *r*-value in the region of the previously reported variant in GIANT). In sex-combined analyses, there were a total of 12 association regions across the genome, which generalized to HCHS/SOL for WHRadjBMI (*r* < 0.05), including three for both women-only and sexes-combined, three for women-only and six for the sexes-combined analysis (Supplementary Material, Table S9). A total of 15 association regions generalized to HCHS/SOL for WCadjBMI, including seven sex-specific loci (two for men, five for women; Supplementary Material, Table S10), one for the sexes-combined only and seven for more than one stratum. Of note, we identified rs6809759 near *PROK2,* which was significantly associated with WCadjBMI in HCHS/SOL for men-only and sexes-combined and was within 500 kb (+/−) of rs12330322, as identified in Shungin *et al*. (14). However, this previously identified locus did not generalize to HCHS/SOL (*r* > 0.05) and may represent an independent association signal in a known region [i.e. all GIANT variants at this locus with *P* < 1 × 10^−6^ exhibit *r* > 0.05 in HCHS/SOL and rs6809759 had a *P* > 1 × 10^−6^ in Shungin *et al*. (14) (*P* = 1.4 × 10^−1^)]. A total of 40 regions generalized to HCHS/SOL for HIPadjBMI, including 29 for sexes-combined, three of which were significant for both women-only and sexes-combined analyses (Supplementary Material, Table S11).

Because some of the SNPs previously reported by GIANT may not have generalized owing to lack of power in HCHS/SOL, we calculated individual-level genetic scores based on trait-increasing alleles for each central adiposity phenotype (Supplementary Material, Table S12) and sex stratum (three association tests per phenotype). For genetic scores based on SNPs with *P*-value < 1 × 10^−7^ in GIANT, all association tests were significant (*P* < 0.05). For genetic scores calculated from GIANT SNPs with 1 × 10^−7^ < *P* < 1 × 10^−6^, six of the nine association tests were significant. Given that only three out of 27 analyses had a *P* > 0.05, there is considerable overlap in the association results of Hispanics/Latinos to those previously reported in the GIANT multi-ethnic analysis.

### Biological curation

We examined the four SNPs (i.e. rs13301996, rs79478137, rs28692724 and rs3168072) in novel loci identified in the replication analyses (Table 1) for association with other phenotypes, gene expression and metabolites in publicly available data using Phenoscanner (36,37), and we assessed the potential regulatory role of these variants and those in LD using publicly available databases, including RegulomeDB (38), Haploreg (39), UCSC GenomeBrowser (40) and GTeX (41). Known associations with these variants meeting Bonferroni-corrected significance after correcting for number of reported associations in Phenoscanner for the four variants within each category (*P* < 0.05/7631 = 6.55 × 10^−5^ for GWAS; *P* < 0.05/88 = 5.68 × 10^−4^ for gene expression; *P* < 0.05/488 = *P* < 1.02 × 10^−4^ for metabolites) are provided in Supplementary Material, Tables S13–S15.

WHRadjBMI-associated variant, rs13301996, which is intronic to cyclin-dependent kinase 5 (CDK5) regulatory subunit-associated protein 2 (*CDK5RAP2*), was significantly associated with the expression of 15 genes and one lncRNA across 17 tissue types (Supplementary Material, Table S13). The most significant of these associations was with multiple epidermal growth factor-like domains 9 (*MEGF9*) in whole blood (*P* = 1.8 × 10^−149^), a gene that rests 30 Kb upstream of rs1330996. This SNP is also significantly associated with expression of *MEGF9* in subcutaneous adipose tissue, sun-exposed skin and T-cells. Additionally, our lead variant in *CDK5RAP2* is associated with the expression of *MEGF9* in whole blood and the testis and with the expression of proteasome (prosome, macropain) 26S subunit, non-ATPase, 5 (*PSMD5*) and/or *PSMD5-AS1* in several relevant tissues, including whole blood, tibial artery, tibial nerve, lung, thyroid, esophagus muscle, skeletal muscle, liver, cerebellum and subcutaneous adipose tissues, among others. There is additional support for a regulatory role of rs13301996 and those with which it is in high LD (*r*^2^ > 0.8). For example, our lead SNP lies just outside of a DNase hypersentivitiy cluster; lies within a region with evidence of histone modification in nine tissues, including brain, skin, muscle and heart; and likely falls in a transcription factor binding site active in skeletal and lung tissue; etc. (Supplementary Material, Table S16) (38–40). While there are multiple lines of evidence for a regulatory role of this variant and multiple genes, rs13301996 has RegulomeDB score of 6, indicating little evidence of binding.

WHRadjBMI-associated SNP, rs79478137, is a low-frequency variant (MAF = 1.6%) intronic in *SLC22A18AS*. This region is subject to genomic imprinting (42), which has been linked with Beckwith-Wiedemann syndrome, a disease caused by an increased rate of growth in children (43–45). Our lead variant is associated with two Electronic Health Record (EHR)-derived phenotypes (cause of death: multisystem degeneration; and cause of death: tongue, unspecified) (Supplementary Material, Table S13) in Phenoscanner. There is limited evidence of a regulatory role for our lead SNP (RegulomeDB score = 4), but rs79478137 is in perfect LD with several variants with evidence of regulation (histone modification, open chromatin, DNAse hypersensitivity and transcription factor binding) in more than 50 tissues, including blood, pancreas, liver and skeletal muscle, hippocampal tissues, etc. (Supplementary Material, Table S16) (38–40).

WCadjBMI-associated SNP, rs3168072, was significantly associated with existing GWAS traits present in Phenoscanner, including ‘cause of death: other specified respiratory disorders’ (Supplementary Material, Table S13). Additionally, rs3168072 is significantly associated with the expression of several genes in whole blood but is most significantly associated with the expression of transmembrane protein 258 (*TMEM258*) (Supplementary Material, Table S14). Rs3168072 is ~95 Kb upstream of *TMEM258*. Our lead variant is likely to play a role in gene expression regulation (RegulomeDB score = 2b, ‘likely to affect binding’) (38). Additionally, our lead variant and those in high LD (*R*^2^ > 0.8) lie within known DNase hypersentivitiy regions and within active areas of histone modification, open chromatin and likely gene enhancer regions (Supplementary Material, Table S16) (38–40). Our lead SNP associated with WCadjBMI, rs3168072, is significantly associated with five lipid-related metabolites (Supplementary Material, Table S15), including ‘Other polyunsaturated fatty acids than 18:2’, ‘CH2 groups in fatty acids’, ‘Ratio of bis allylic bonds to double bonds in lipids’, ‘CH2 groups to double bonds ratio’ and ‘Ratio of bis allylic bonds to total fatty acids in lipids’.

Our lead SNP associated with HIPadjBMI in women, rs28692724 (NC_000014.9:g.77027445C>T), is a synonymous variant exonic to interferon regulatory factor 2-binding protein-like (*IRF2BPL*) that is significantly associated with expression of the same gene in whole blood (Supplementary Material, Table S14). Additionally, this variant lies in a known CCCTC-binding factor (CTCF)-binding site (RegulomDB Score = 2b), among other transcription factors, and a DNAse Hypersentivity cluster (Supplementary Material, Table S16) (38–40).

## Discussion

We performed the first large-scale GWAS of three central adiposity traits (i.e. WHRadjBMI, WCadjBMI and HIPadjBMI) in a sample of approximately 12 672 Hispanic/Latino individuals. We identified 16 variants that were suggestively associated (*P* < 1 × 10^−6^) with WHRadjBMI, 22 for WCadjBMI and 28 for HIPadjBMI. Of these 66 variants that were suggestively associated with the three central adiposity traits, four novel loci replicated after meta-analysis with replication samples. Additionally, we demonstrated that eight previously identified GWAS loci generalized to Hispanic/Latino study participants for WHRadjBMI, 10 for WCadjBMI and 12 for HIPadjBMI in HCHS/SOL.

### Discovery of four novel loci

Given the large number of published GWAS on central adiposity measures, it may seem surprising that four novel loci (rs13301996, rs79478137, rs28692724 and rs3168072) were mapped. There are a few explanations for these novel findings, including (1) previous GWAS were primarily conducted in European populations. Indeed, all four novel SNPs were absent from previous GIANT HapMap imputed analyses (14), and one (rs28692724) of the four absent from a more recent GWAS that included Europeans from the UK Biobank (33); (2) the consideration of a broad spectrum of ancestrally diverse Hispanic/Latino populations, including not just those of Mexican ancestry but also those with ancestry from the Caribbean, Central, and/or South America (46); (3) the use of the entire 1000 Genomes Phase I Reference panel, including populations with Native American ancestry: Mexico (MXL), Colombia (CLM) and Puerto Rico (PUR); (4) demonstrated differences in the patterning of body composition by ancestry (47,48). More specifically, African ancestry populations have lower body fat percentages than men and women of non-Hispanic European, Native American and East Asian ancestry at the same BMI. Additionally, non-Hispanic African ancestry men and women have greater skeletal and muscle mass than their non-Hispanic European ancestry counterparts who, in turn, have greater skeletal and muscle mass than men and women of East Asian origin (47,49–51).

Recent GWAS for coding variation of WHRadjBMI identified the importance of central adiposity genes in lipid regulation, storage and homeostasis (52). Similarly, we found a novel association of a variant in *FADS2* (rs3168072) with WCadjBMI following meta-analysis of HCHS/SOL results with the results from an independent sample of European descent individuals, which further implies a role of this locus in central adiposity and lipid homeostasis. Genetic variations in the *FADS2* gene has been associated with several traits related to obesity and cardiometabolic health, including fatty acid metabolism and adipose tissue inflammation, leading to an interaction between weight loss and *FADS2* genes in the regulation of adipose tissue inflammation (53). A nearby variant, rs174546 (*R*^2^ = 0.3523, *D*′ = 0.916 in AMR), in *FADS1* has previously been associated with four lipid traits (54). The A allele (MAF = 38%) for our lead SNP is associated with greater WC in our samples and is nearly fixed among sub-Saharan African populations (99% in 1000 Genomes AFR) at very high frequency in European populations (97% in EUR) and at a lower frequency in East Asian (75% in EAS) and Native American populations (63% in AMR). Rs3168072 is intronic to *FADS2*—a member of the fatty acid desaturase (FADS) gene family—and is involved in the endogenous conversion of short-chain polyunsaturated fatty acids to long chain fatty acids. The *FADS* cluster of genes appears to have been under strong selection in several human populations, which likely explains the large differences in allele frequencies across global populations (55–58) and why previous GWASs of waist traits that primarily focused on European descent populations did not detect an association signal in this region.

We identified a novel association for WHRadjBMI with rs13301996 following meta-analysis with an independent sample of African descent individuals. Rs13301996 is intronic to *CDK5RAP2*, which encodes a regulator of CDK5 activity (59), interacts with CDK5R1 and pericentrin (PCNT) (59), plays a role in centriole engagement and microtubule nucleation (60) and has been linked to primary microcephaly and Alzheimer’s disease (61,62). In addition, we identified a novel association for WHRadjBMI with rs79478137 (*P*-value = 3.64E^−9^) in Hispanic/Latino women. Rs79478137 is intronic to the antisense *SLC22A18AS* gene, which is highly expressed in the liver and kidney as well as in the gastrointestinal tract and placenta. Very little is known of the biological role of this gene (63), and *SLC22A18AS* has no counterpart in mice or other rodents (64). Thus, although its genomic organization is known, the regulation and function of this gene is not understood (65).

Lastly, we identified a novel association for HIPadjBMI at rs28692724 following meta-analysis with an independent sample of European women. Rs28692724 is a synonymous variant in *IRF2BPL*, which encodes a transcription factor that, acting within the neuroendocrine system, plays a role in regulating female reproductive function (66).

### Differences in association by background group

Many of the loci mapped in this study displayed effect heterogeneity by background group. For example, the *NTM* locus associated with WHRadjBMI in women, displayed nearly 3-fold the effect size in the mainland background group when compared with the Caribbean background group. Also, for the women-only primary analysis, rs77186623 in the *LOC105375745* locus displayed a 4-fold greater effect in the Caribbean background group compared with the mainland group. These and other loci displaying heterogeneity by background group (i.e. *MARCKSL1*, *C19orf67*, *RIT2*, *MAP4K4*, *COBL*, *NREP* and *ANO10*) were not validated in replication analyses, possibly due in part to heterogeneity by background group.

### Limitations

A limitation of this study was the small sample size within each HCHS/SOL background group. However, the use of genetic analysis groups in our main analyses accounted for the heterogeneity of genetic effects among ethnic groups often ignored in GWAS studies. Compared with self-identified background groups, genetic analysis groups are more genetically homogeneous and lack principal component outliers in stratified analysis, which may hinder detection of and adjustment for important population structure when ignored (67). In addition, genetic analysis groups allow all individuals to be classified in a specific group, whereas many individuals in HCHS/SOL have a missing or non-specific self-identified background (67). Therefore, by using genetic analysis groups in our analysis rather than self-identified groups, we have increased our study’s power to detect novel and previously documented associations with central adiposity traits (67). Owing to the diverse background of our discovery population, another limitation was the lack of an ideal replication study. We attempted to overcome this limitation by focusing on both multi-ethnic meta-analyses, which would validate those associations that generalize across ancestries, and meta-analyses stratified by ancestry, which may allow for validation of more population-specific associations. However, it is possible that the limited Native American ancestry present across our replication cohorts may have hindered replication, and further analyses in more diverse Hispanic/Latino populations are needed to confirm the relevance of promising central adiposity associated loci identified in our study. Last, we attempted to leverage bioinformatics databases to assist in evaluating the potential functional effects of our top associations, including lookup of previous evidence of cis regulation of gene expression. However, a possible limitation of these lookups is the lack of diversity in resources like GTEx, which are derived from European ancestry populations (Supplementary Material, Table S14), and thus our tag SNP may not be well represented owing to differences in the LD structure. Future investigations into the potential regulatory function of our associated loci are needed in ancestrally relevant sample populations and available Omics data.

## Conclusion

We identified four novel loci for central adiposity traits in a large population of Hispanic/Latino Americans. We also found that several previously identified central adiposity loci discovered in European American populations generalized to Hispanic/Latino Americans. Many of the loci interrogated exhibit background-group-specific effects, likely owing to population history (admixture and natural selection), that have resulted in changes in LD, or allele frequency differences or owing to variation in etiology. These observations highlight the importance of large-scale genomic studies in ancestrally diverse populations for identifying obesity-susceptibility loci that generalize and those that are ancestry-specific.

## Materials and Methods

### Study sample

Details on the study and sampling design of the HCHS/SOL have been previously described (68). Briefly, HCHS/SOL is a community-based prospective cohort study of 16 415 self-identified Hispanic/Latino adults who were aged 18–74 years at screening from randomly selected households in four US field centers (Chicago, IL; Miami, FL; Bronx, NY and San Diego, CA) with baseline examination (2008–2011) and yearly telephone follow-up assessment for at least 3 years. The HCHS/SOL cohort includes participants who self-identified as being Central American (*n* = 1732), Cuban (*n* = 2348), Dominican (*n* = 1473), Mexican (*n* = 6472), Puerto-Rican (*n* = 2728) and South American (*n* = 1072). The goals of the HCHS/SOL are to describe the prevalence of risk and the protective factors for chronic conditions (e.g. CVD, diabetes and pulmonary disease) and to quantify all-cause mortality, fatal and non-fatal CVD and pulmonary disease and pulmonary disease exacerbation over time. The baseline clinical examination (69) included comprehensive biological (e.g. anthropometrics, blood draw, oral glucose tolerance test, ankle brachial pressure index and electrocardiogram), behavioral (e.g. dietary intake assessed with two 24 h recalls, physical activity assessment by accelerometer and self-report, overnight sleep exam for apneic events, tobacco and alcohol assessed by self-report) and socio-demographic (e.g. socioeconomic status and migration history) assessments. This study was approved by the institutional review boards at each field center where all subjects gave written informed consent.

Participants in HCHS/SOL self-identified their background as Mexican, Central American, South American (mainland), Puerto Rican, Cuban or Dominican (Caribbean). Some participants chose ‘more than one’, ‘other’ or chose not to self-identify. We addressed the missing or inconsistent data in self-identified background groups by defining ‘genetic analysis groups’ described in Conomos *et al*. (67). To increase power in this analysis, we chose to stratify by the broader mainland or Caribbean categories rather than more specific groups. In this paper, we will use the term ‘background group’ to refer to a super-group of genetic analysis groups by geographic region, mainland or Caribbean. Hispanics/Latinos have admixed ancestry from three continents: Africa, America and Europe. In general, participants from the mainland group have higher proportions of American ancestry and lower African ancestry, while participants in the Caribbean group have higher proportions of African ancestry (67).

### Phenotypes

All variables were taken from the baseline visit. Participants were dressed in scrub suits or light non-constricting clothing, and shoes were removed for weight and height measurements. WC and HIP were measured using Gulick II 150 and 250 cm anthropometric tape and rounded to the nearest centimeter (cm). Height was measured using a wall-mounted stadiometer and rounded to the nearest cm, and weight measured with a Tanita Body Composition Analyzer, TBF-300A, to the nearest tenth of a kg. Height and weight were used to calculate BMI (kg/m^2^). We applied a log10 transformation on HIP owing to its non-normal trait distribution.

### Genotyping

Our analyses included 7472 women and 5200 men of mainland (Mexican, Central and South American) or Caribbean (Puerto Rican, Cuban and Dominican) ancestry residing in the USA. All participants were genotyped on the Illumina SOL Omni2.5M custom content array, which was subsequently used to impute millions of additional variants, based on the entire 1000 Genomes Phase I Reference panel, including populations with Native American ancestry: MXL, CLM and PUR. Pre-phasing was performed using SHAPEIT, followed by imputation with IMPUTE2 (70,71).

### Discovery analyses

Owing to known differences in genetic effects on waist and hip traits between men and women (14,32,72), we analyzed associations stratified by sex for each trait, in addition to the entire sample. We used linear mixed-model regression, assuming an additive genetic model adjusted for age, age^2^, study center, sample weights, genetic analysis background group (67,73), principal components to account for ancestry, population structure using kinship coefficients and sample eigenvectors, household, census block group and sex in the combined analysis. Kinship, household and block group were treated as random effects in each model. Sample weights were incorporated in our models as a fixed effect to account for oversampling of the communities in the 45–74 age group (*n* = 9714, 59.2%), which was intended to facilitate the examination of HCHS/SOL target outcomes. HCHS/SOL sampling weights are the product of a ‘base weight’ (reciprocal of the probability of selection) and three adjustments: (1) non-response adjustments made relative to the sampling frame, (2) trimming to handle extreme values (to avoid a few weights with extreme values being overly influential in the analyses) and (3) calibration of weights to the 2010 US Census according to age, sex and Hispanic background. We used genetic analysis groups in our analyses that accounted for heterogeneity of genetic effects among ethnic groups. Compared with self-identified background groups, genetic analysis groups are more genetically homogeneous and lack principal component outliers in stratified analysis, which may hinder detection of and adjustment for important population structure when ignored (67). In addition, genetic analysis groups allow all individuals to be classified in a specific group, whereas many individuals in HCHS/SOL have a missing or non-specific self-identified background (67). Also, we conducted stratified analyses by region (mainland vs. Caribbean) to identify potential heterogeneity in effect by background group. We examined heterogeneity across background group using *I*^2^ statistics calculated using METAL (74) and tested for significant interaction (*P*_diff_ < 0.05) by background group using EasyStrata (75).

To decrease the number of spurious associations, we filtered all results on MAF < 0.5%, Hardy–Weinberg Equilibrium (HWE) *P* < 1 × 10^−7^, minor allele count [MAC (effective *N*)] < 30 (67). Additionally, we categorized suggestive loci as those with variants reaching *P* < 1 × 10^−6^ and with at least one additional variant within 500 kb+/− with a *P* < 1 × 10^−5^. We used regional association plots produced in LocusZoom to visualize association regions using unrelated individuals from HCHS/SOL for LD (http://locuszoom.sph.umich.edu/).

### Local ancestry estimation

We estimated local ancestry (African, Native American and European) using RFMix (76), which applies a conditional-random-field-based approach for estimation to inform differences by background group. We used 236 456 genotyped SNPs available in both HCHS/SOL and reference-panel datasets in the Human Genome Diversity Project (HGDP) (77), HapMap 3 (78) and 1000 Genomes phase 1 for detecting African, Native American and European ancestry. We used BEAGLE (v.4) to phase and impute sporadic missing genotypes in the HCHS/SOL and reference-panel datasets (79).

### Replication and meta-analyses

An aim of our study was to identify genetic variants that associate with central adiposity, which may vary by ancestry. Therefore, we sought to replicate our association findings using 1000 Genomes imputed GWAS data available in independent cohorts, including eight studies with Hispanics/Latinos (HL: *N* up to 12 341), three studies with African Americans (AA; *N* up to 12 496) and one study with European-Americans (EUR: *N* up to 8845). Study design and descriptive statistics for each replication study are provided in Supplementary Material, Table S1. Each replication study excluded individuals who were pregnant or exhibited extreme values for waist or hip measures (outside of ±4 SD from the mean). Each study used measures from a single visit with the greatest sample size. We used linear regression (or linear mixed effects models if the study had related individuals) association analyses on the trait residuals after adjustment for age, age^2^, principal components to account for ancestry, BMI, other study specific factors (e.g. study center) and sex in the sex-combined analysis, stratified by race/ethnicity where applicable for each SNP that reached suggestive significance (*P* < 1 × 10^−6^) in the discovery analysis.

We employed a fixed-effects meta-analysis using the inverse variance-weighted method for WHRadjBMI and WCadjBMI. For HIPadjBMI, owing to trait transformations, we used sample-size-weighted meta-analysis. All meta-analyses were implemented in METAL (80). We conducted meta-analyses stratified by race/ethnicity group and combined across groups. We included SNPs with a study- and stratum-specific imputation quality (Rsq) greater than 0.4, HWE *P*-value greater than 1 × 10^−7^ and a MAC greater than five. To declare statistical significance for replicated loci, we required in each replication sample a trait and stratum-specific *P* < 0.05 with a consistent direction of effect with discovery and genome-wide significance (*P* < 5 × 10^−8^) when meta-analyzed together with HCHS/SOL.

### Generalization

To examine whether previously reported association regions generalized to the HCHS/SOL, we downloaded the publicly available multi-ethnic (European, Asian and African ancestry) GWAS results from the GIANT consortium (14) for WHRadjBMI, WCadjBMI and HIPadjBMI (https://portals.broadinstitute.org/collaboration/giant/index.php/GIANT_consortium_data_files#GIANT_consortium_2012-2015_GWAS_Metadata_is_Available_Here_for_Download) in men, women and sexes-combined, and then we applied the framework of Sofer *et al*. (2017) for generalization testing (81). We took all variant associations with *P* < 1 × 10^−6^ in GIANT and identified the matching association test in HCHS/SOL. For each such association, we calculated a directional False Discovery Rate (FDR) *r*-value by combining the *P*-values from GIANT and HCHS/SOL, while accounting for multiple testing and for the direction of estimated associations in each of the studies. An association was declared as generalized, while controlling for the FDR at the 0.05 level, if its *r*-value was smaller than 0.05. Multiple SNPs from the same region were tested. Therefore, in an iterative procedure, we pruned the results list by identifying the SNP with the lowest *r*-value in an analysis, then finding all SNPs in a 1 MB region around it and removing them from the list. Thus, the number of generalized regions is the number of generalized SNPs in the pruned list.

We also hypothesized that some regions did not generalize owing to lack of power (the HCHS/SOL sample size is much smaller than the GIANT sample size). To test this, we took all tested SNPs from the non-generalized regions and considered the GIANT multi-ethnic GWAS results. In an iterative procedure, we pruned the list by first identifying the SNP with lowest GIANT *P*-value in the analysis, then found all SNPs in a 1 MB region around it and removed them from the list. We repeated until no SNPs remained. All the SNPs in the pruned list were selected solely based on their GIANT *P*-values. Since there were many such variants, we further grouped them according to their *P*-values. Groups were formed by trait, sex (men, women and combined) and GIANT *P*-value (between 10^−6^ and 10^−7^, between 10^−7^ and 10^−8^ and smaller than 10^−8^). For each such group of SNPs, we created a genetic risk score (GRS) in HCHS/SOL. For each sex stratum and each group of SNPs, the value of the GRS was the sum of all trait increasing alleles in that group. We chose an unweighted GRS as effect sizes derived from primarily European ancestry GWAS are not easily transferable to admixed populations (82). We tested the GRS in the appropriate analysis group (men, women and combined). A low *P*-value implies that some of the SNPs in the group are likely associated with the trait in HCHS/SOL.

### Biological curation

To gain further insight into the possible functional role of the identified variants and to assess the relevance of our identified variants with other phenotypes, we conducted lookups of our replicated variants in multiple publicly available databases, including PhenoScanner (36), RegulomeDB (38), Haploreg (39) and UCSC GenomeBrowser (40). Additionally, we conducted lookups of nearby genes in GTeX (41). The R package HaploR was used to query HaploReg and RegulomeDB (https://cran.r-project.org/web/packages/haploR/vignettes/haplor-vignette.html).

## Supplementary Material

Supplementary_Materials_ddab166
