# Supplementary material for: Genome-wide association study of body fat distribution traits in Hispanics/Latinos from the HCHS/SOL
Source: Hum Mol Genet. 2021 Jun 24;30(22):2190–204. doi: 10.1093/hmg/ddab166 (PMC8561424; doi:10.1093/hmg/ddab166)
Supplement: Supplementary_Materials_ddab166 [file supplementary_materials_ddab166.pdf]

Supplementary Materials

Table of Contents

Supplementary Figures		Pages 3-100
Supplementary Figure 1	Manhattan Plot: WHRadjBMI, Sexes-Combined	3
Supplementary Figure 2	Locus Zoom Plots: WHRadjBMI, Sexes-Combined	4-8
Supplementary Figure 3	Manhattan Plot: WHRadjBMI, Women	9
Supplementary Figure 4	Locus Zoom Plots: WHRadjBMI, Women	10-18
Supplementary Figure 5	Manhattan Plot: WHRadjBMI, Men	19
Supplementary Figure 6	Locus Zoom Plots: WHRadjBMI, Men	20-25
Supplementary Figure 7	QQ Plots: WHRadjBMI	26
Supplementary Figure 8	Manhattan Plot: WCadjBMI, Sexes-Combined	27
Supplementary Figure 9	Locus Zoom Plots: WCadjBMI, Sexes-Combined	28-37
Supplementary Figure 10	Manhattan Plot: WCadjBMI, Women	38
Supplementary Figure 11	Locus Zoom Plots: WCadjBMI, Women	39-41
Supplementary Figure 12	Manhattan Plot: WCadjBMI, Men	42
Supplementary Figure 13	Locus Zoom Plots: WCadjBMI, Men	43-54
Supplementary Figure 14	QQ Plots: WCadjBMI	55
Supplementary Figure 15	Manhattan Plot: HIPadjBMI, Sexes-Combined	56
Supplementary Figure 16	Locus Zoom Plots: HIPadjBMI, Sexes-Combined	57-65
Supplementary Figure 17	Manhattan Plot: HIPadjBMI, Women	66
Supplementary Figure 18	Locus Zoom Plots: HIPadjBMI, Women	67-79
Supplementary Figure 19	Manhattan Plot: HIPadjBMI, Men	80
Supplementary Figure 20	Locus Zoom Plots: HIPadjBMI, Men	81-90
Supplementary Figure 21	QQ Plots: HIPadjBMI	91
Supplementary Figure 22	Generalization of Known Loci: WHRadjBMI, Sexes-Combined	92
Supplementary Figure 23	Generalization of Known Loci: WHRadjBMI, Women	93
Supplementary Figure 24	Generalization of Known Loci: WHRadjBMI, Men	94
Supplementary Figure 25	Generalization of Known Loci: WCadjBMI, Sexes-Combined	95
Supplementary Figure 26	Generalization of Known Loci: WCadjBMI, Women	96
Supplementary Figure 27	Generalization of Known Loci: WCadjBMI, Men	97
Supplementary Figure 28	Generalization of Known Loci: HIPadjBMI, Sexes-Combined	98
Supplementary Figure 29	Generalization of Known Loci: HIPadjBMI, Women	99
Supplementary Figure 30	Generalization of Known Loci: HIPadjBMI, Men	100
**Supplementary Tables**		**Pages 101-121**
Supplementary Table 1	Study Descriptives and Genotyping Info	101
Supplementary Table 2	Discovery and Replication Results: WHRadjBMI	102
Supplementary Table 3	Discovery and Replication Results: WCadjBMI	103-104
Supplementary Table 4	Discovery and Replication Results: HIPadjBMI	105-106
Supplementary Table 5	Association by Background Group: WHRadjBMI	107
Supplementary Table 6	Association by Background Group: WCadjBMI	108
Supplementary Table 7	Association by Background Group: HIPadjBMI	109
Supplementary Table 8	Local Ancestry	110-111
Supplementary Table 9	Top Variant Generalization: WHRadjBMI	112
Supplementary Table 10	Top Variant Generalization: WCadjBMI	113

Supplementary Table 11	Top Variant Generalization: HIPadjBMI	114
Supplementary Table 12	Genetic Risk Score Generalization	115
Supplementary Table 13	Phenoscanner Results: GWAS	116
Supplementary Table 14	Phenoscanner Results: eQTL	117-118
Supplementary Table 15	Phenoscanner Results: mQTL	119
Supplementary Table 16	HaploReg Results (Note: Full results in separate file)	120-123

Supplementary Figure 1. Manhattan plot. Manhattan plot of the sexes-combined analysis for WHRadjBMI. All suggestively significant (*P*<1×10^{-6}) variants are highlighted in orange if they are >500 Kb from any previously-reported WHRadjBMI associated variants. Previously reported loci (+/- 500 Kb) are highlighted in blue if any variant in the locus reached suggestive significance. All suggestively significant loci that meet our criteria for replication are annotated with the closest gene. †Replicated in African American meta-analysis. ‡ Replicated in Hispanic/Latino meta-analysis. ¥ Replicated in European American meta-analysis.

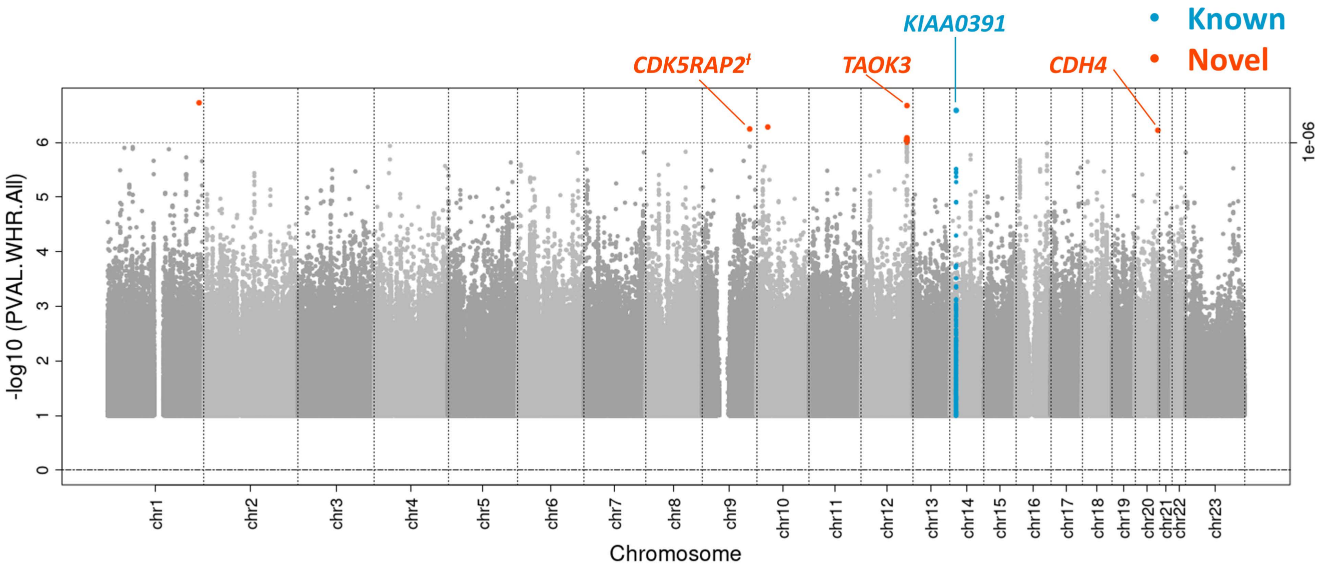

Supplementary Figure 2. Locus Zoom Plots. Regional association plots for suggestively significant loci in the HCHS/SOL WHRadjBMI sexes-combined analysis. The plots appear in chromosome:position order. Dot color reflects R^2 calculated from the 1000 Genomes AMR reference dataset. Point symbols represent variant functional classifications: a) rs13301996, *CDK5RAP2*; b) rs115981023, *TAOK3*; c) rs185566196, *KIAA0391*; d) rs116612483, *CDH4*.

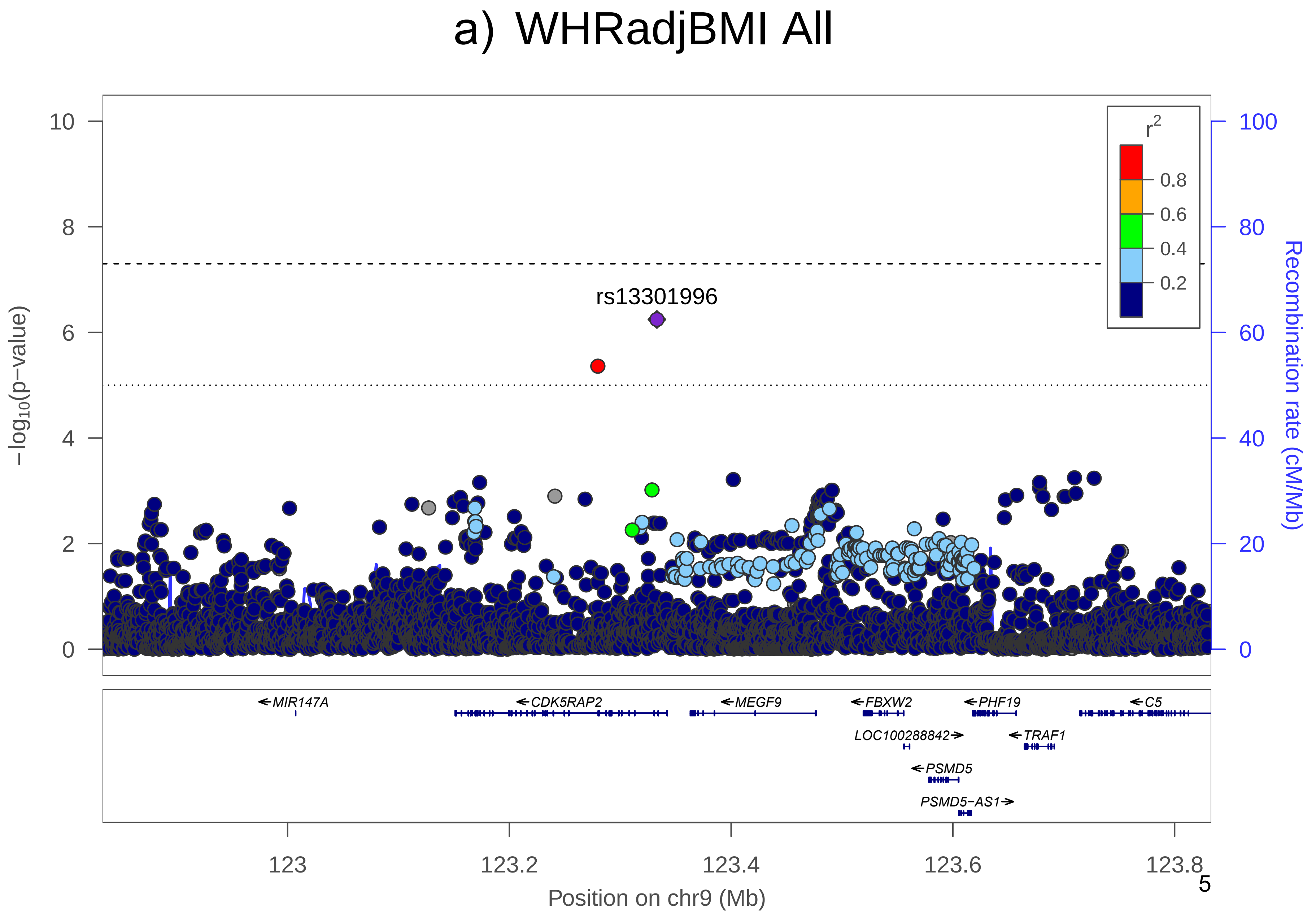
a) WHRadjBMI All
rs13301996
r²
0.8
0.6
0.4
0.2
−log10(p−value)
Recombination rate (cM/Mb)
0
2
4
6
8
10
0
20
40
60
80
100
MIR147A
CDK5RAP2
MEGF9
FBXW2
PHF19
C5
LOC100288842
TRAF1
PSMD5
PSMD5−AS1
123
123.2
123.4
123.6
123.8
Position on chr9 (Mb)

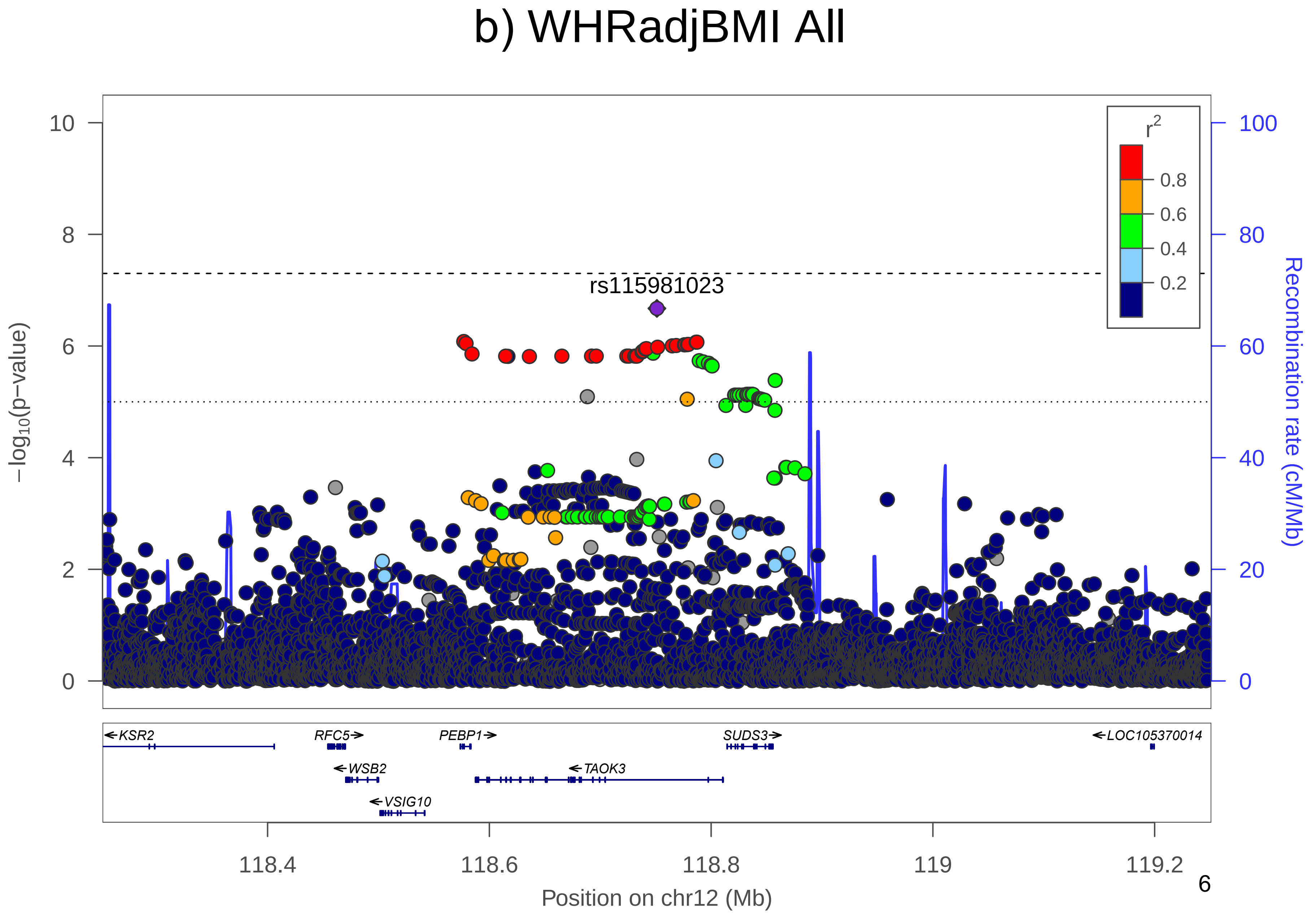

b) WHRadjBMI All
rs115981023
r2
0.8
0.6
0.4
0.2
10
8
6
4
2
0
−log10(p−value)
100
80
60
40
20
0
Recombination rate (cM/Mb)
KSR2
RFC5
PEBP1
SUDS3
LOC105370014
WSB2
TAOK3
VSIG10
118.4
118.6
118.8
119
119.2
Position on chr12 (Mb)

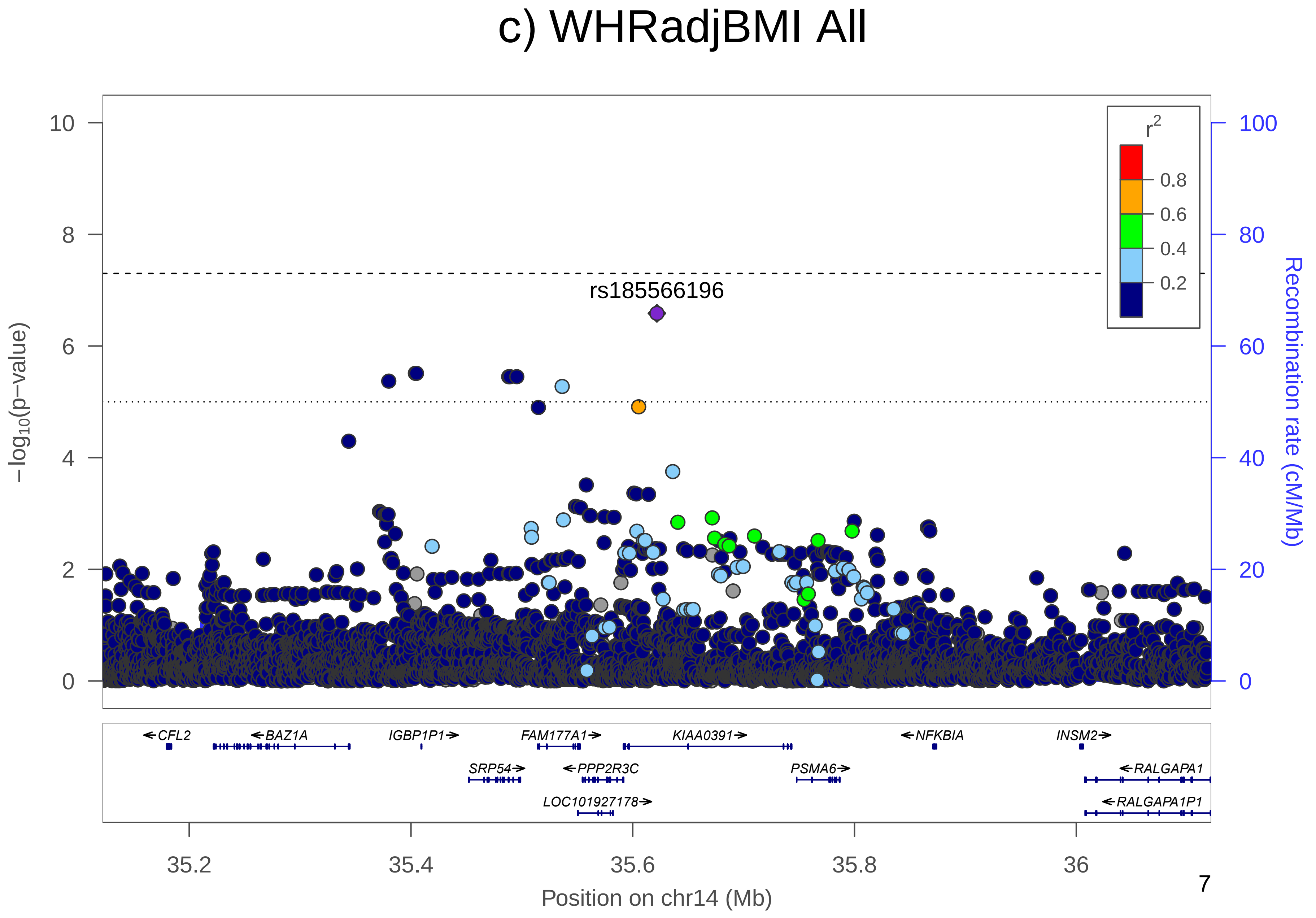
c) WHRadjBMI All
−log10(p−value)
10
8
6
4
2
0
Recombination rate (cM/Mb)
100
80
60
40
20
0
r2
0.8
0.6
0.4
0.2
rs185566196
CFL2
BAZ1A
IGBP1P1
FAM177A1
KIAA0391
NFKBIA
INSM2
SRP54
PPP2R3C
PSMA6
RALGAPA1
LOC101927178
RALGAPA1P1
35.2
35.4
35.6
35.8
36
Position on chr14 (Mb)

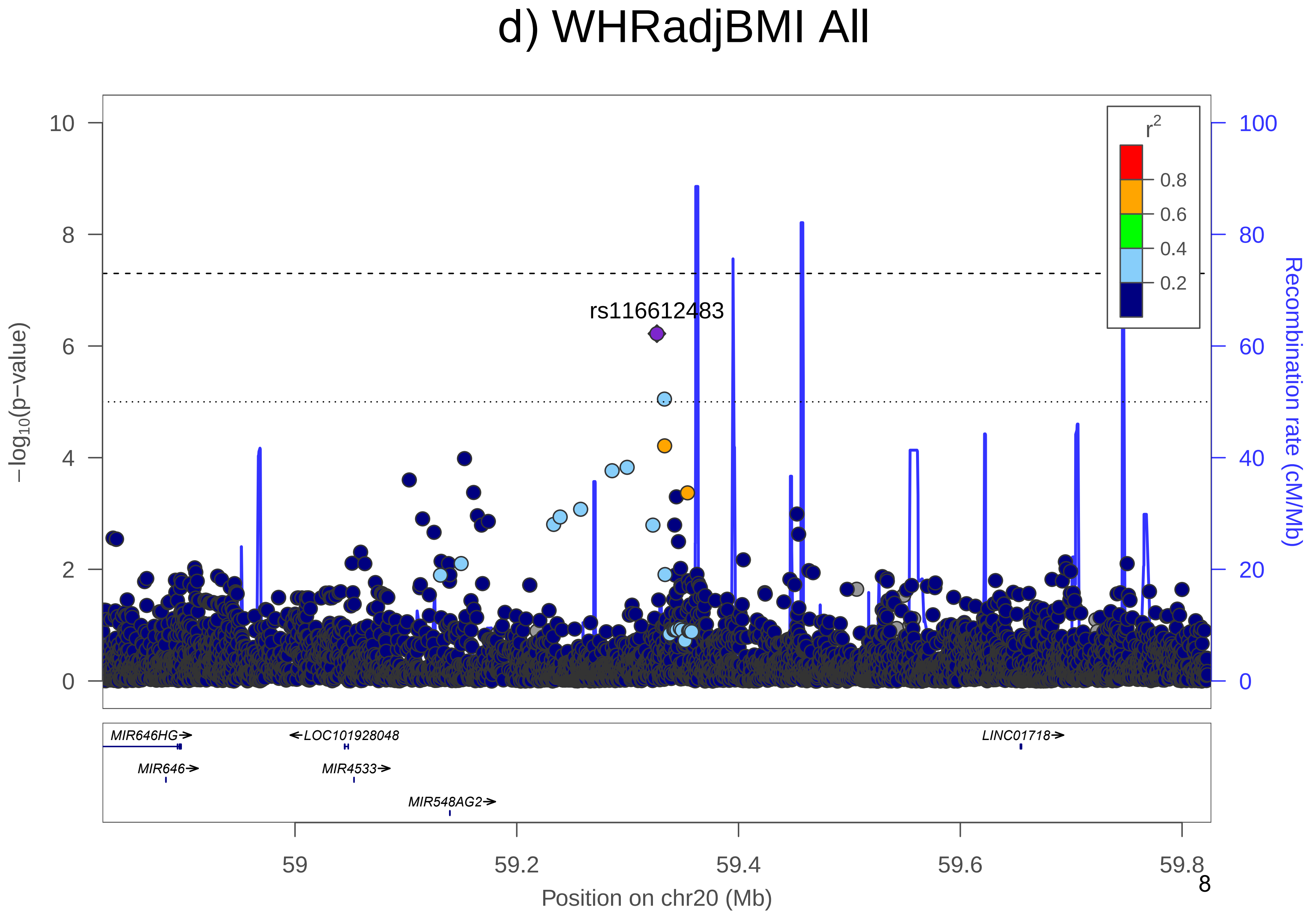

d) WHRadjBMI All
rs116612483
−log10(p−value)
Recombination rate (cM/Mb)
r2
0.8
0.6
0.4
0.2
0
2
4
6
8
10
0
20
40
60
80
100
MIR646HG
LOC101928048
LINC01718
MIR646
MIR4533
MIR548AG2
59
59.2
59.4
59.6
59.8
Position on chr20 (Mb)

Supplementary Figure 3. Manhattan plot. Manhattan plot of the women-only analysis for WHRadjBMI. All suggestively significant (*P*<1x10^{-6}) variants are highlighted in orange if they are >500 Kb from any previously-reported WHRadjBMI associated variants. Previously reported loci (+/- 500 Kb) are highlighted in blue if any variant in the locus reached suggestive significance. All suggestively significant loci that meet our criteria for replication are annotated with the closest gene. †Replicated in African American meta-analysis. ‡ Replicated in Hispanic/Latino meta-analysis. ¥ Replicated in European American meta-analysis.

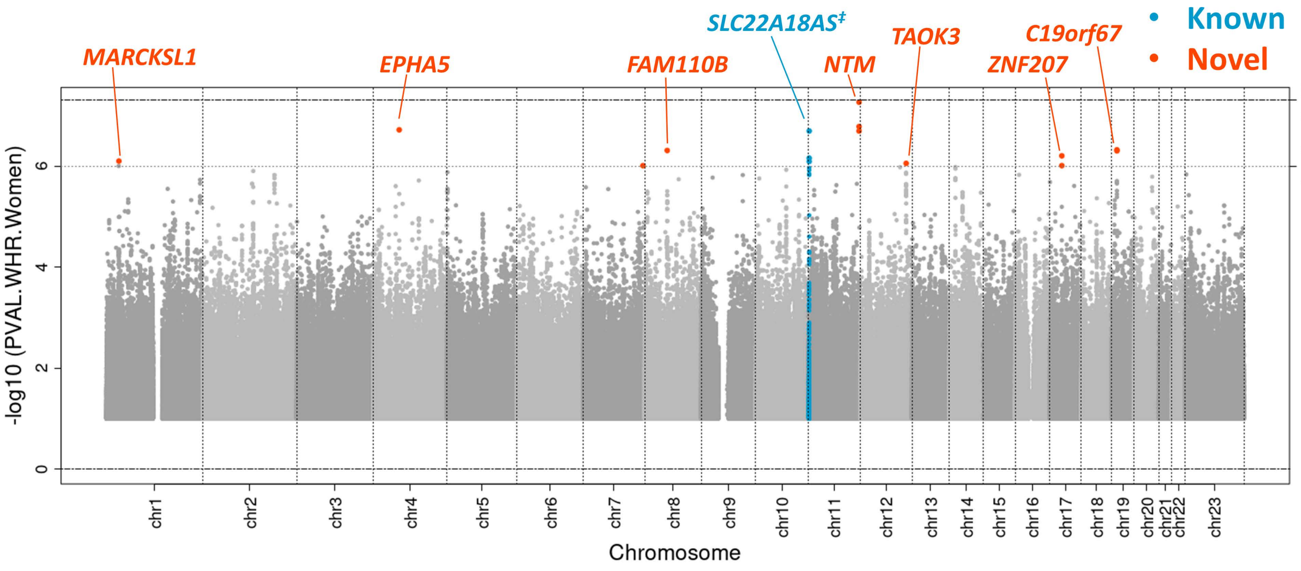

Supplementary Figure 4. Locus Zoom Plots. Regional association plots for suggestively significant loci in the HCHS/SOL WHRadjBMI women-only analysis. The plots appear in chromosome:position order. Dot color reflects R^2 calculated from the 1000 Genomes AMR reference dataset. Point symbols represent variant functional classifications: a) rs77377042, *MARCKSL1*; b) rs75120960, *EPHA5*; c) rs16922424, *FAM110B*; d) rs79478137, *SLC22A18AS*; e) rs113818604, *NTM*; f) rs115981023, *TAOK3*; g) rs146900844, *ZNF207*; h) rs61305557, *C19orf67*.

a) WHRadjBMI Women

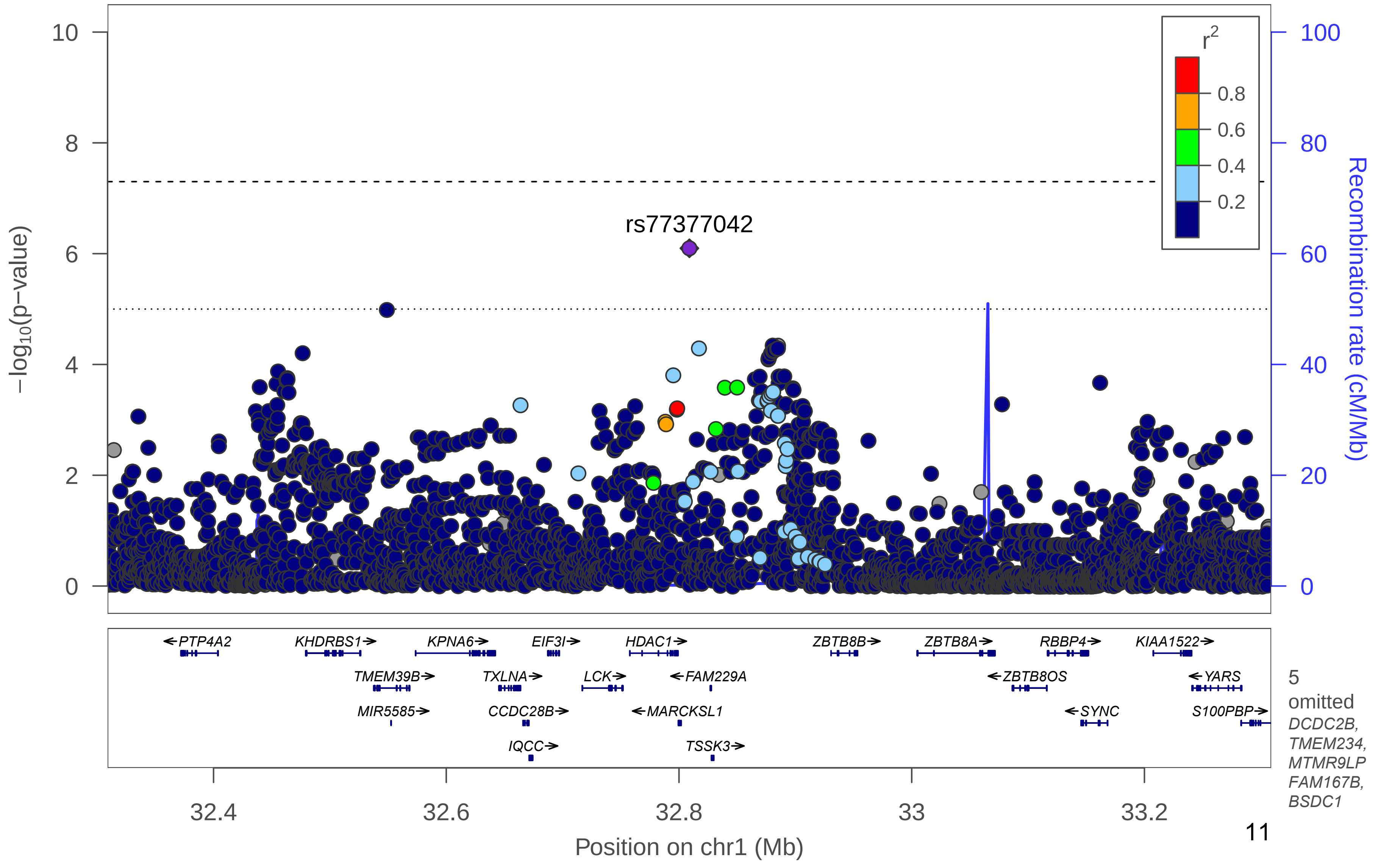

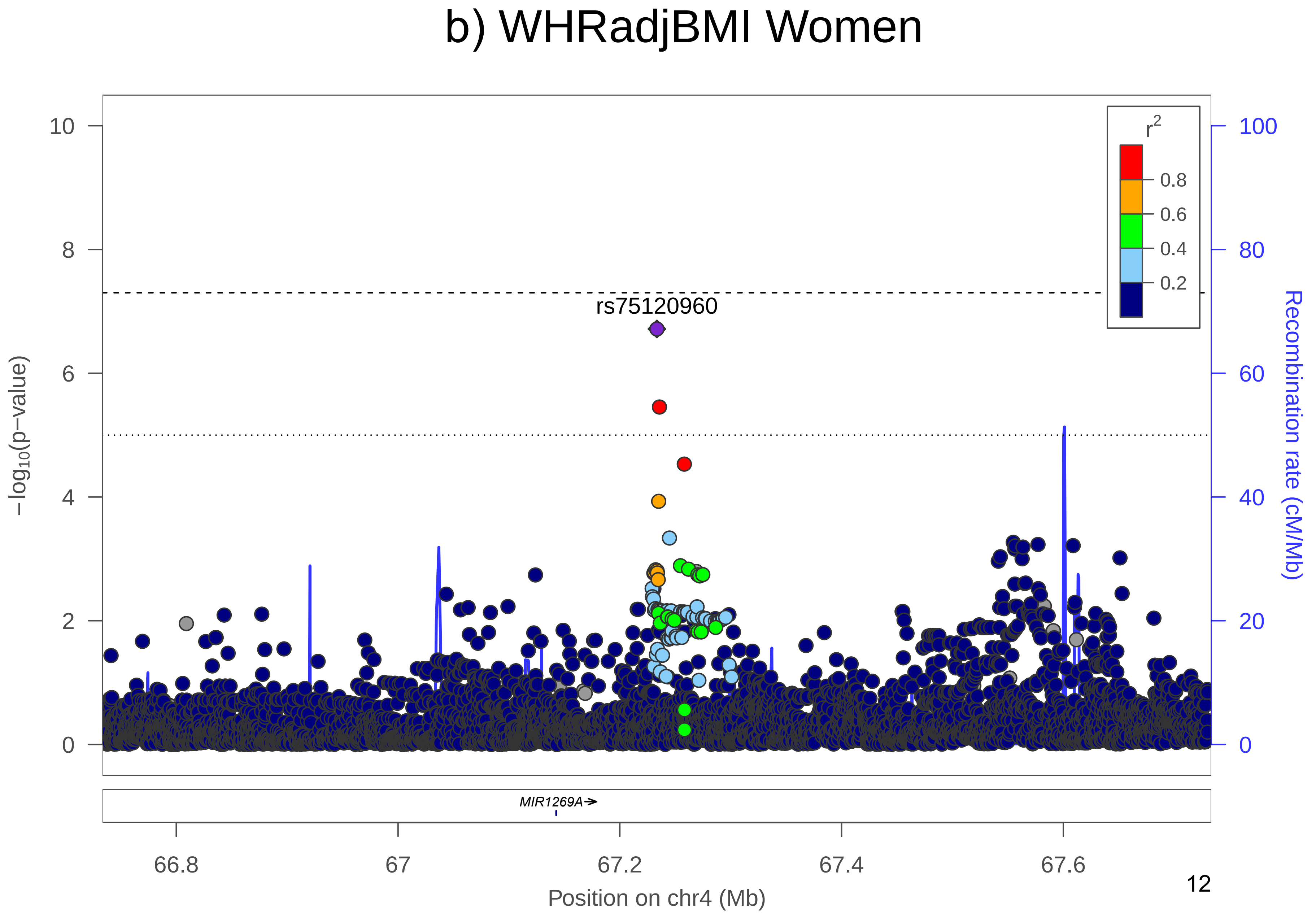
b) WHRadjBMI Women
r2
0.8
0.6
0.4
0.2
rs75120960
−log10(p−value)
Recombination rate (cM/Mb)
10
8
6
4
2
0
100
80
60
40
20
0
MIR1269A
66.8
67
67.2
67.4
67.6
Position on chr4 (Mb)

c) WHRadjBMI Women

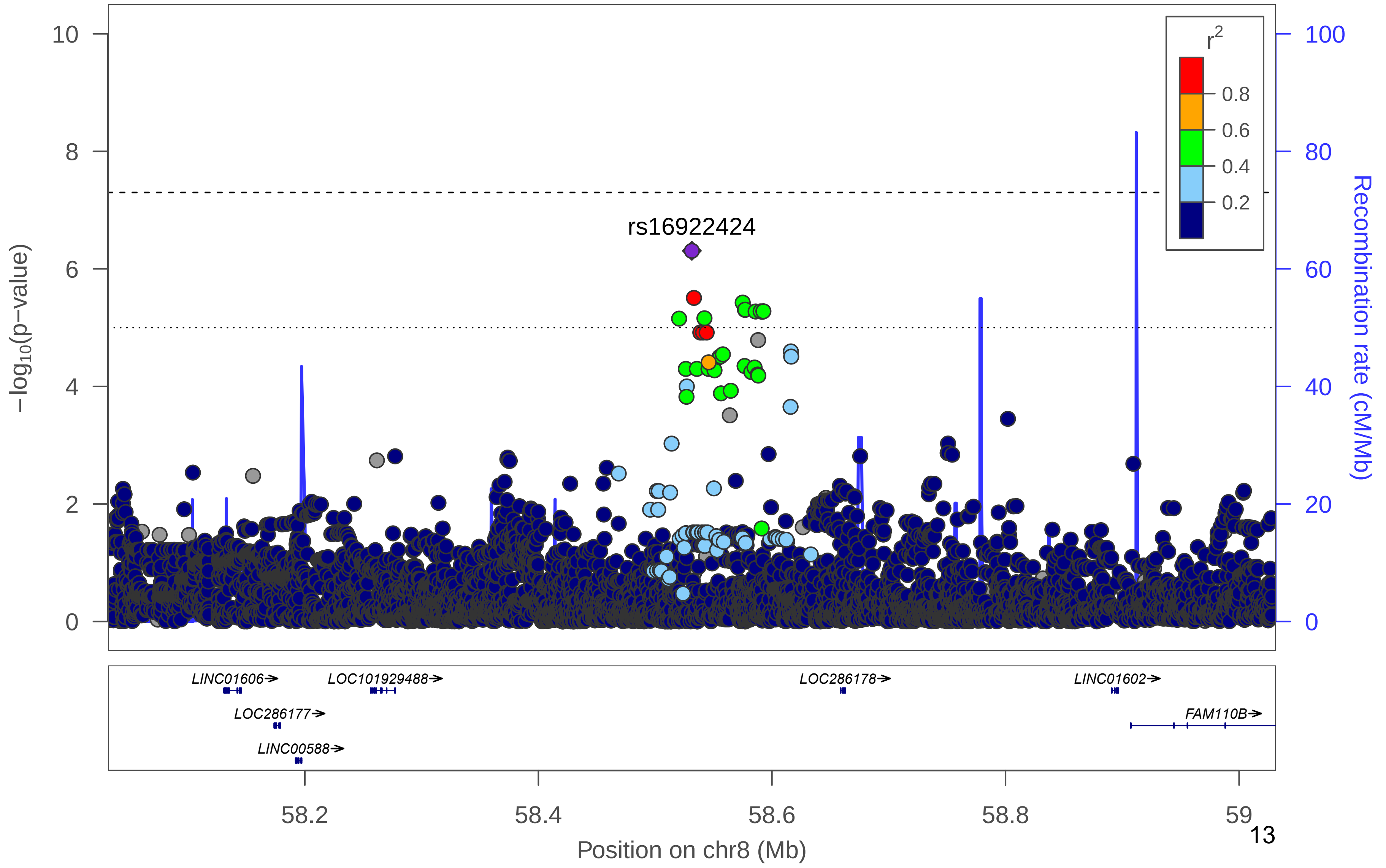

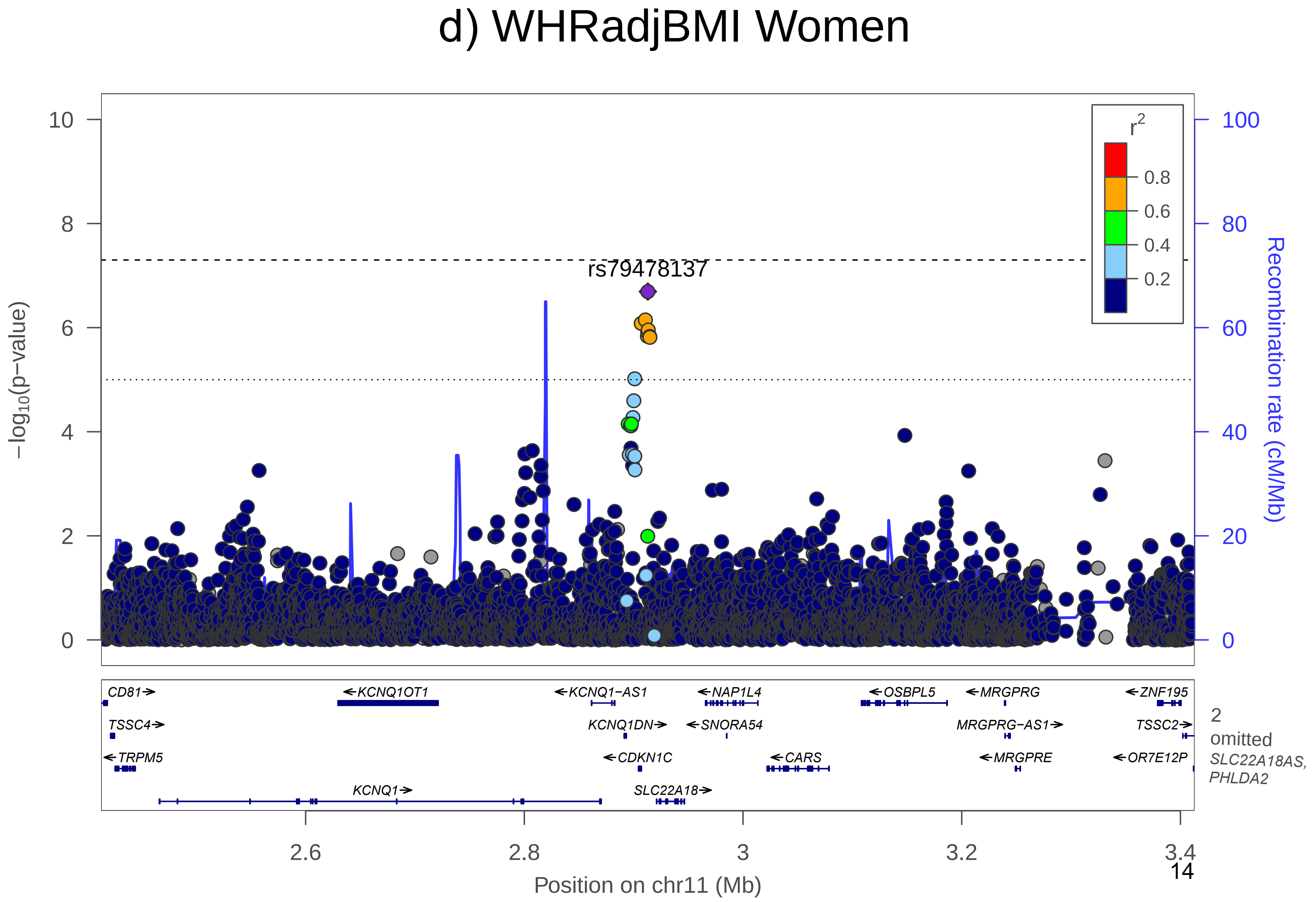

d) WHRadjBMI Women
−log10(p−value)
Recombination rate (cM/Mb)
r2
0.8
0.6
0.4
0.2
rs79478137
0
2
4
6
8
10
0
20
40
60
80
100
CD81→
←KCNQ1OT1
←KCNQ1−AS1
←NAP1L4
←OSBPL5
←MRGPRG
←ZNF195
TSSC4→
KCNQ1DN→
←SNORA54
MRGPRG−AS1→
TSSC2→
←TRPM5
←CDKN1C
←CARS
←MRGPRE
←OR7E12P
KCNQ1→
SLC22A18→
2
omitted
SLC22A18AS,
PHLDA2
2.6
2.8
3
3.2
3.4
Position on chr11 (Mb)

e) WHRadjBMI Women

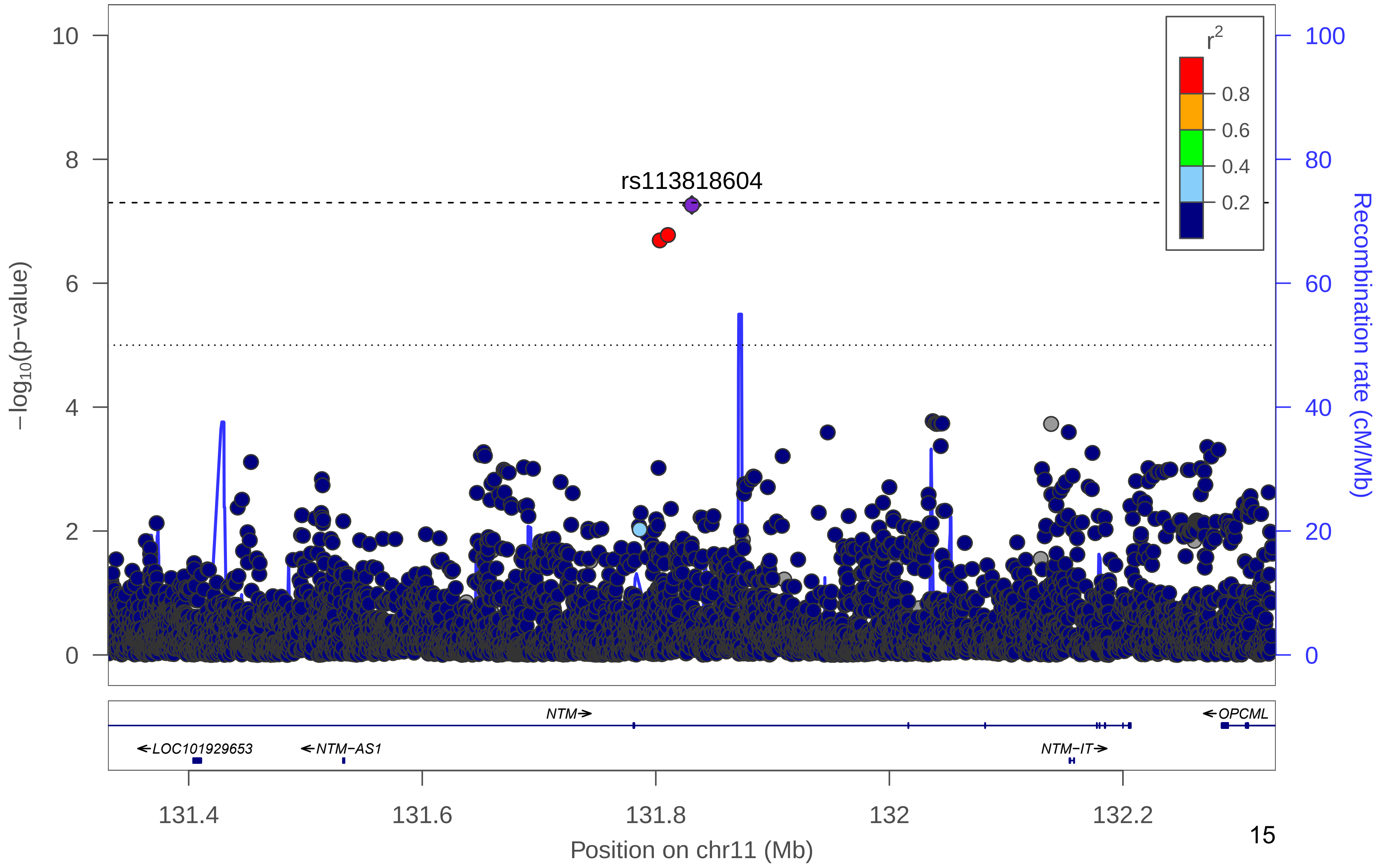

f) WHRadjBMI Women

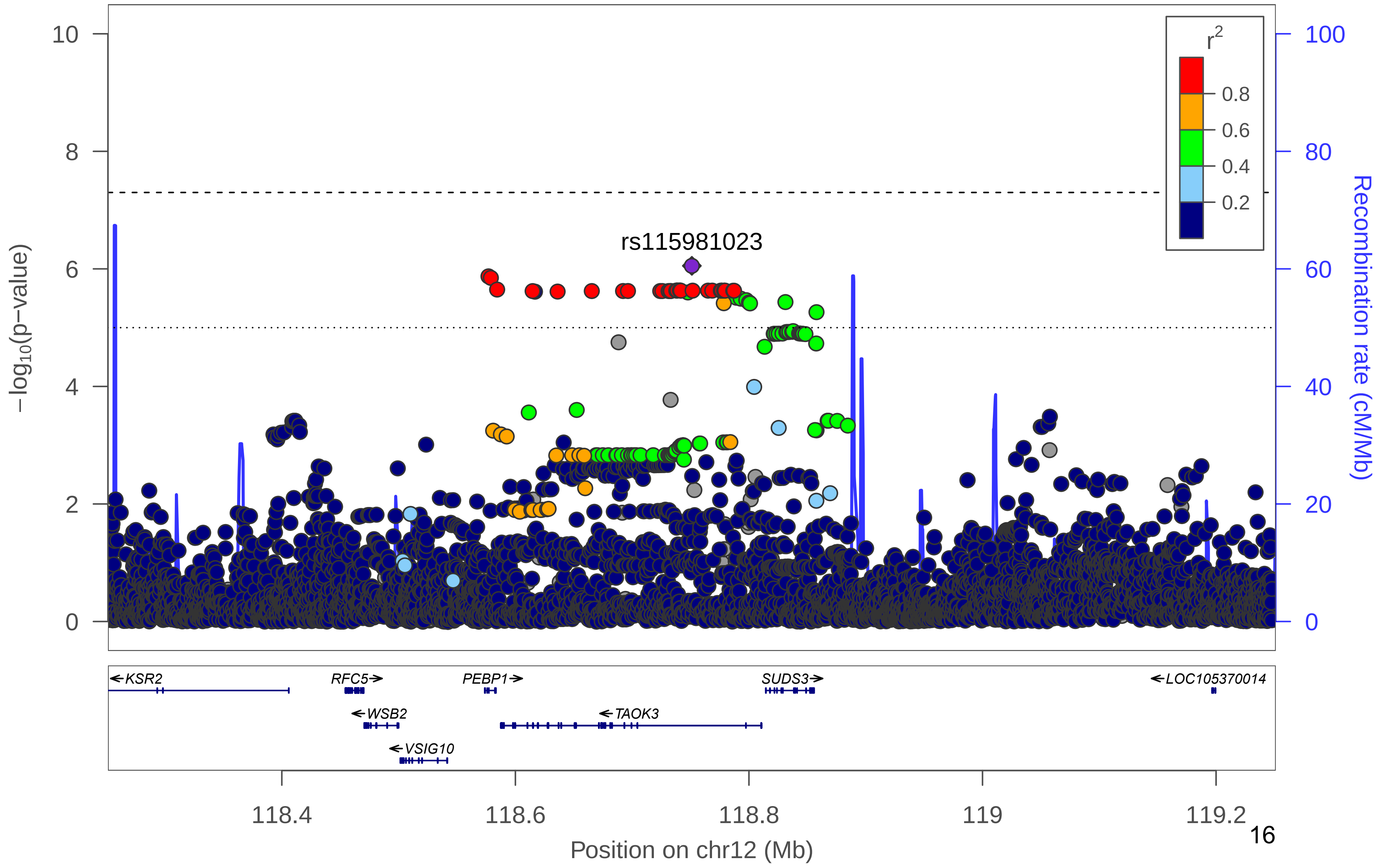

g) WHRadjBMI Women

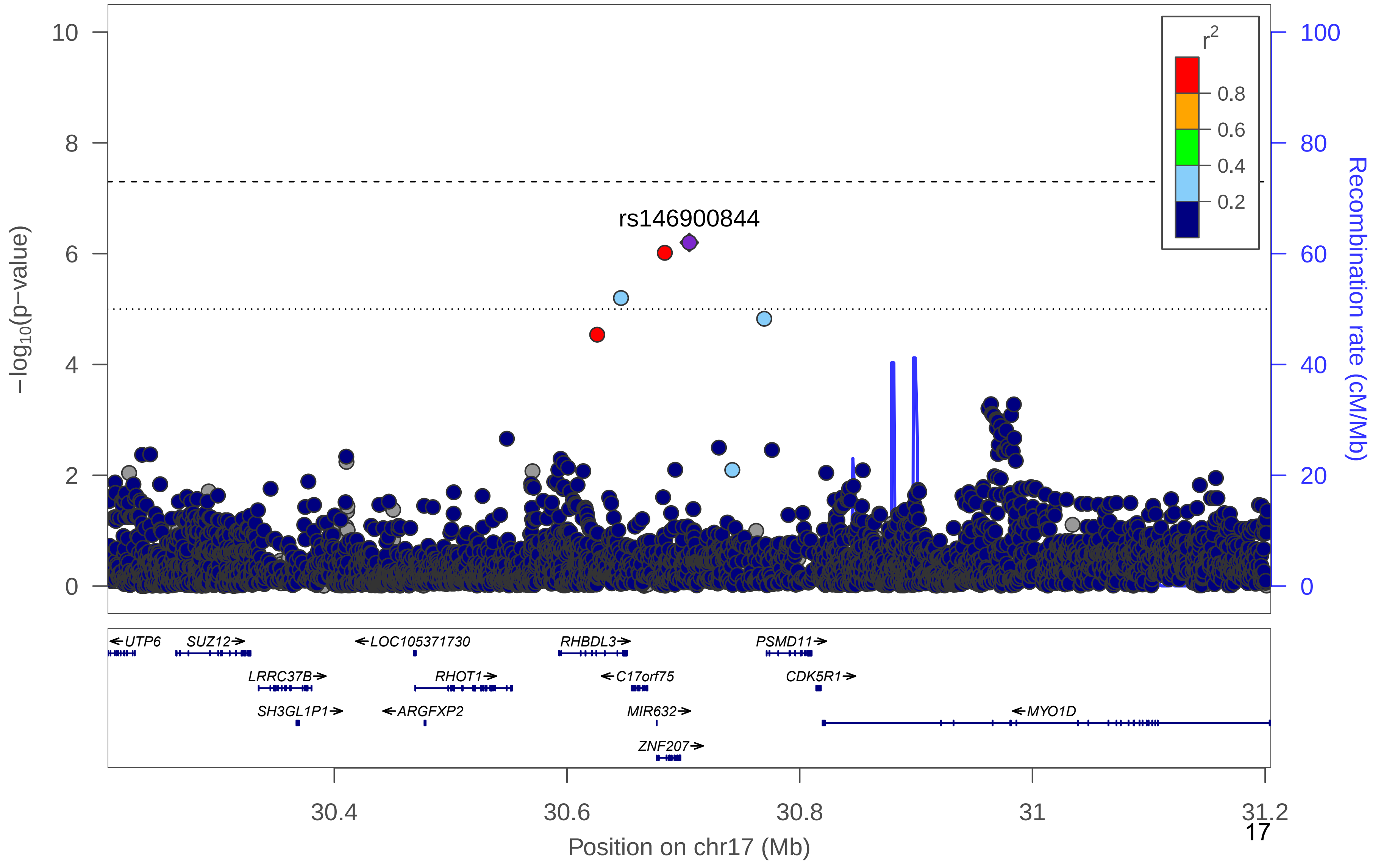

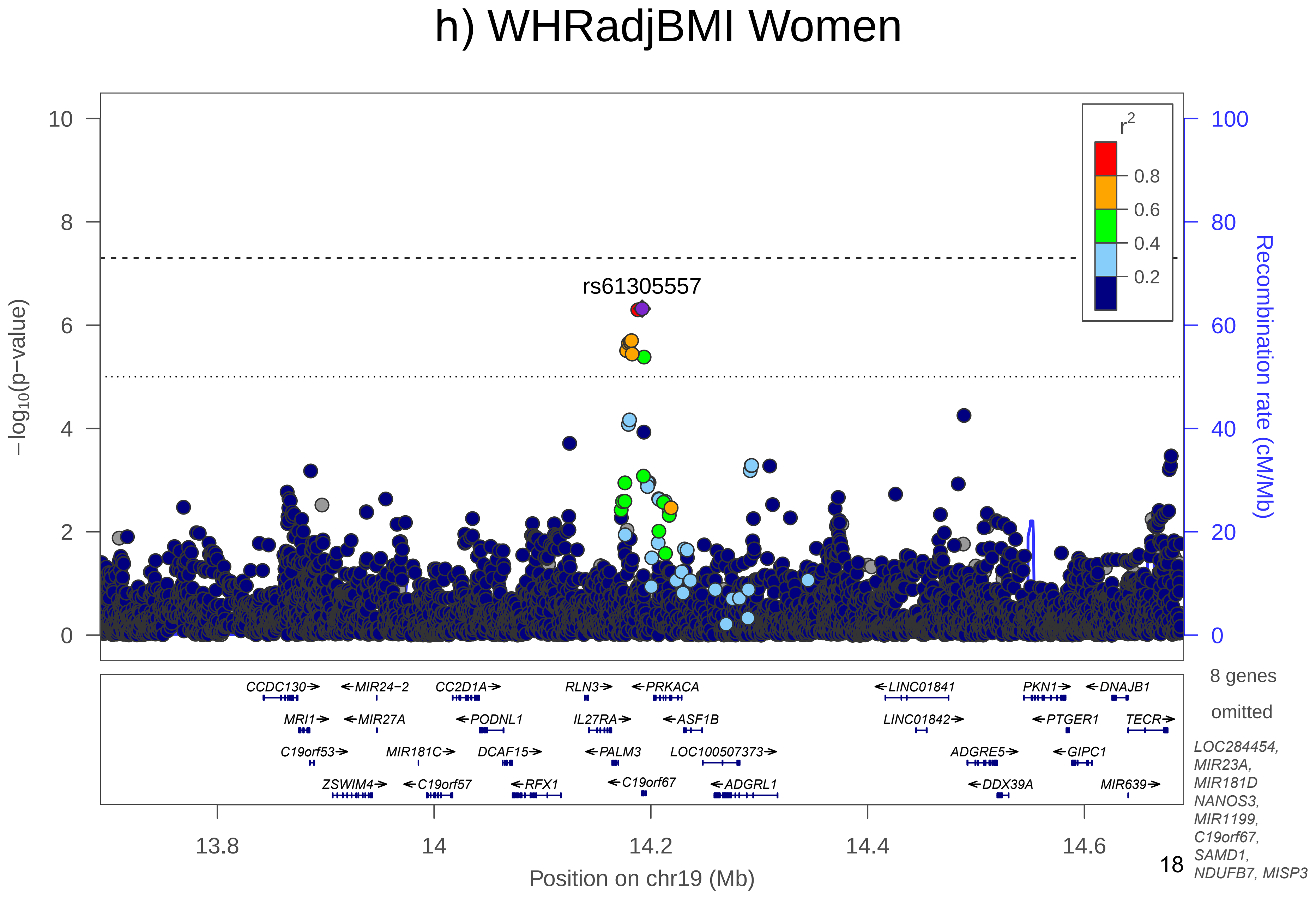

h) WHRadjBMI Women
rs61305557
r2
0.8
0.6
0.4
0.2
−log10(p−value)
Recombination rate (cM/Mb)
Position on chr19 (Mb)
13.8
14
14.2
14.4
14.6
CCDC130
MIR24−2
CC2D1A
RLN3
PRKACA
LINC01841
PKN1
DNAJB1
MRI1
MIR27A
PODNL1
IL27RA
ASF1B
LINC01842
PTGER1
TECR
C19orf53
MIR181C
DCAF15
PALM3
LOC100507373
ADGRE5
GIPC1
ZSWIM4
C19orf57
RFX1
C19orf67
ADGRL1
DDX39A
MIR639
8 genes omitted
LOC284454, MIR23A, MIR181D NANOS3, MIR1199, C19orf67, SAMD1, NDUFB7, MISP3

Supplementary Figure 5. Manhattan plot. Manhattan plot of the men-only analysis for WHRadjBMI. All suggestively significant ($P<1x10^{-6}$) variants are highlighted in orange if they are >500 Kb from any previously-reported WHRadjBMI associated variants. Previously reported loci (+/- 500 Kb) are highlighted in blue if any variant in the locus reached suggestive significance. All suggestively significant loci that meet our criteria for replication are annotated with the closest gene. †Replicated in African American meta-analysis. ‡ Replicated in Hispanic/Latino meta-analysis. ¥ Replicated in European American meta-analysis.

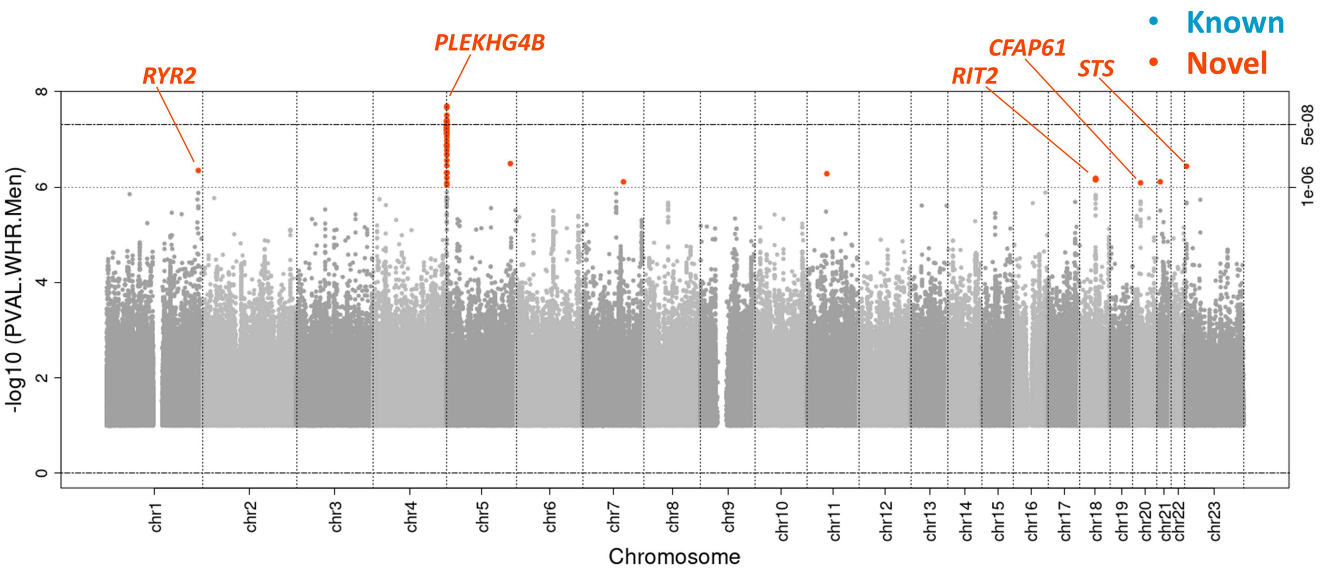

Supplementary Figure 6. Locus Zoom Plots. Regional association plots for suggestively significant loci in the HCHS/SOL WHRadjBMI men-only analysis. The plots appear in chromosome:position order. Dot color reflects R^2 calculated from the 1000 Genomes AMR reference dataset. Point symbols represent variant functional classifications: a) rs12032174, *RYR2*; b) rs10475310, *PLEKHG4B*; c) rs16977373, *RIT2*; d) rs721424, *CFAP61*; e) rs148213302, *STS*.

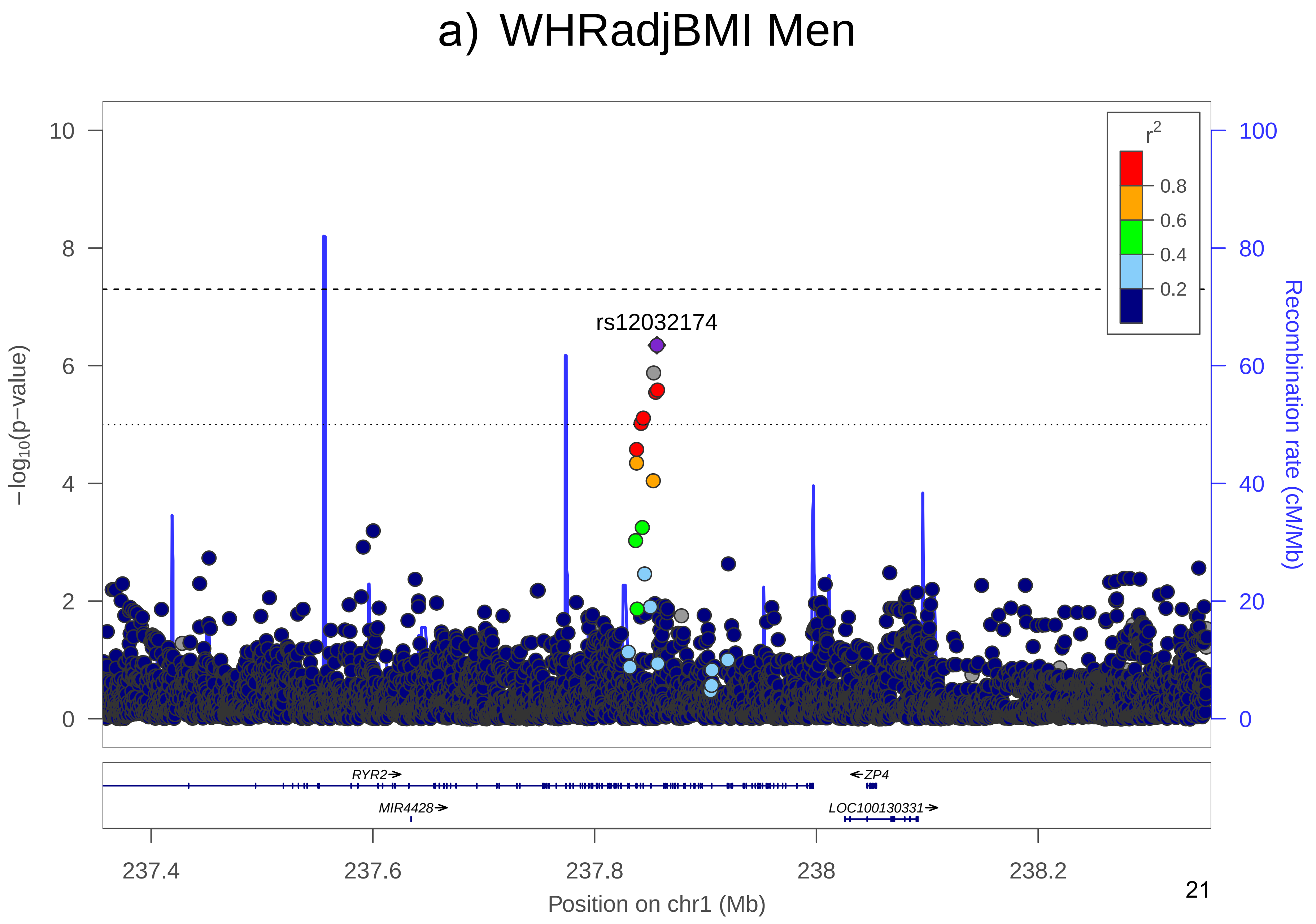
a) WHRadjBMI Men
rs12032174
r2
0.8
0.6
0.4
0.2
−log10(p−value)
Recombination rate (cM/Mb)
RYR2
ZP4
MIR4428
LOC100130331
237.4
237.6
237.8
238
238.2
Position on chr1 (Mb)

b) WHRadjBMI Men

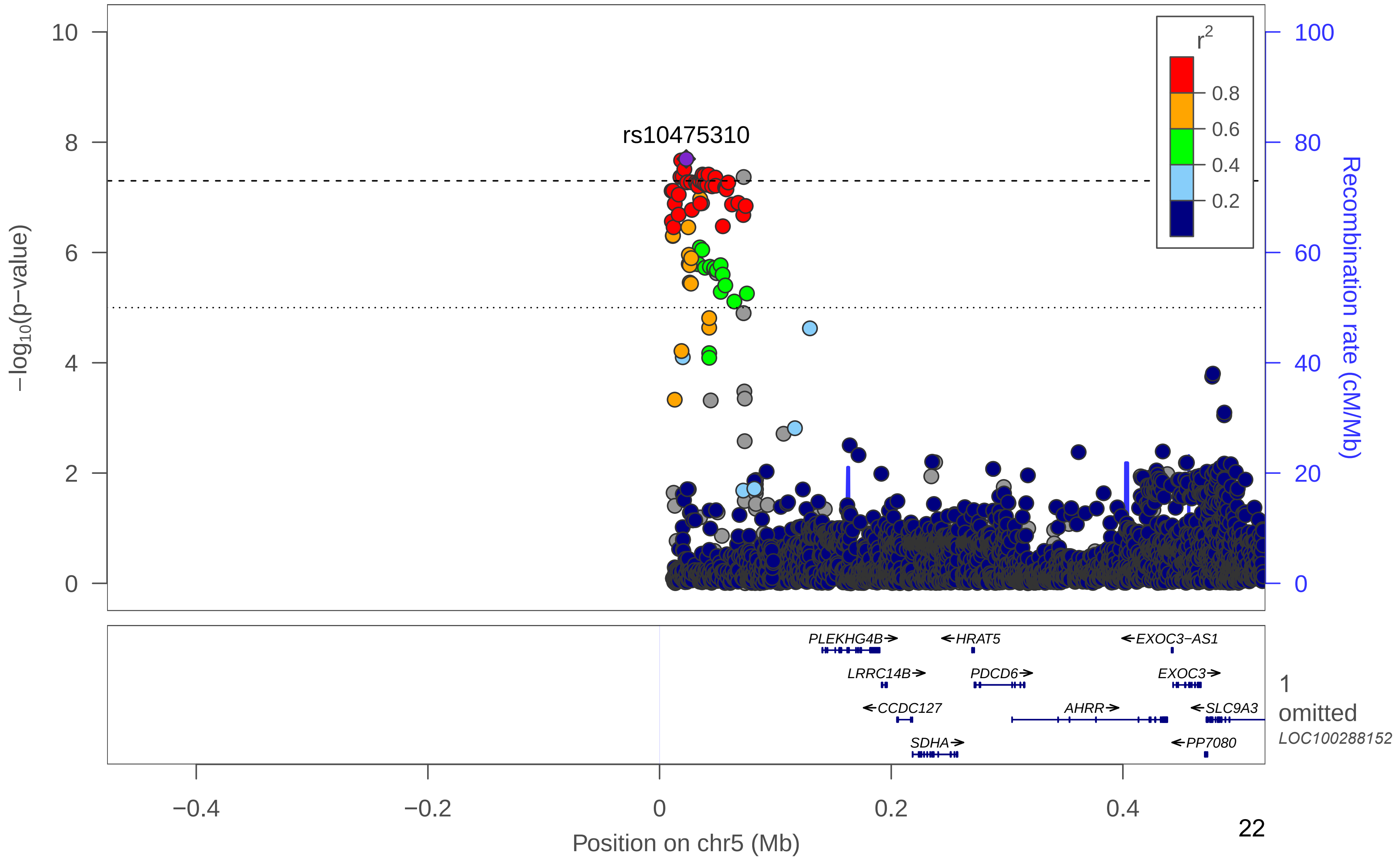

c) WHRadjBMI Men

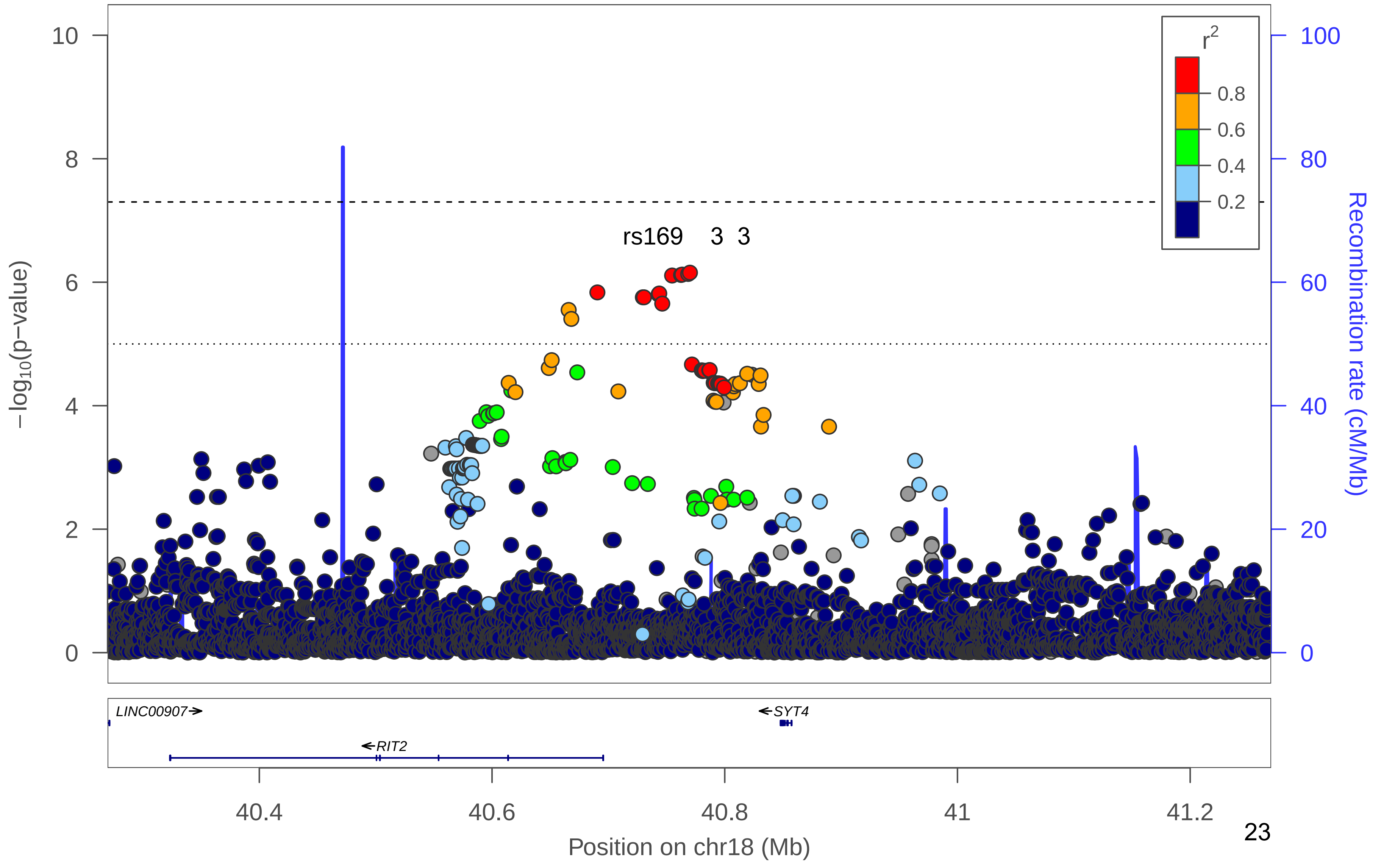

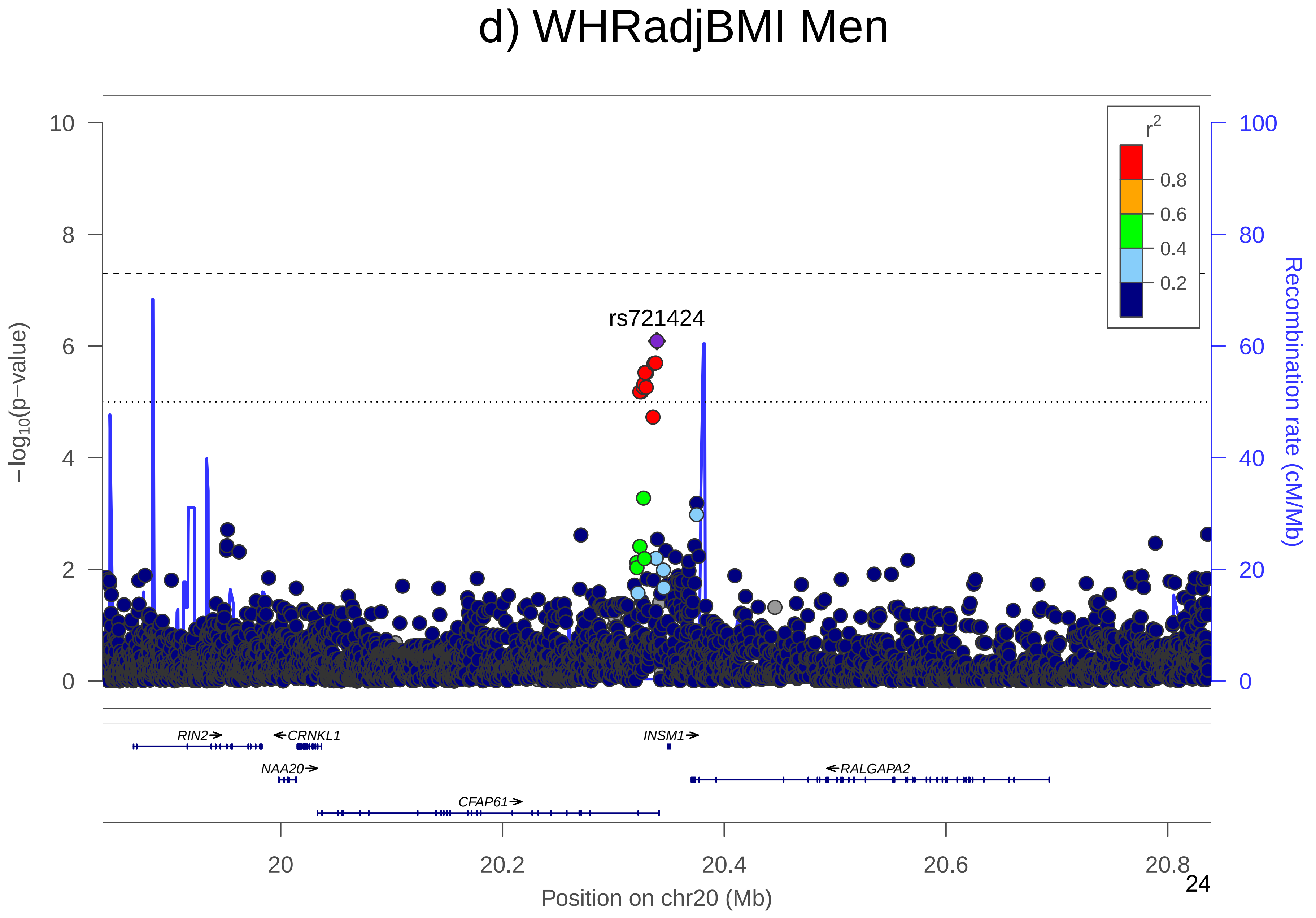

d) WHRadjBMI Men
rs721424
r2
0.8
0.6
0.4
0.2
−log10(p−value)
Recombination rate (cM/Mb)
RIN2
CRNKL1
INSM1
NAA20
RALGAPA2
CFAP61
Position on chr20 (Mb)

e) WHRadjBMI Men

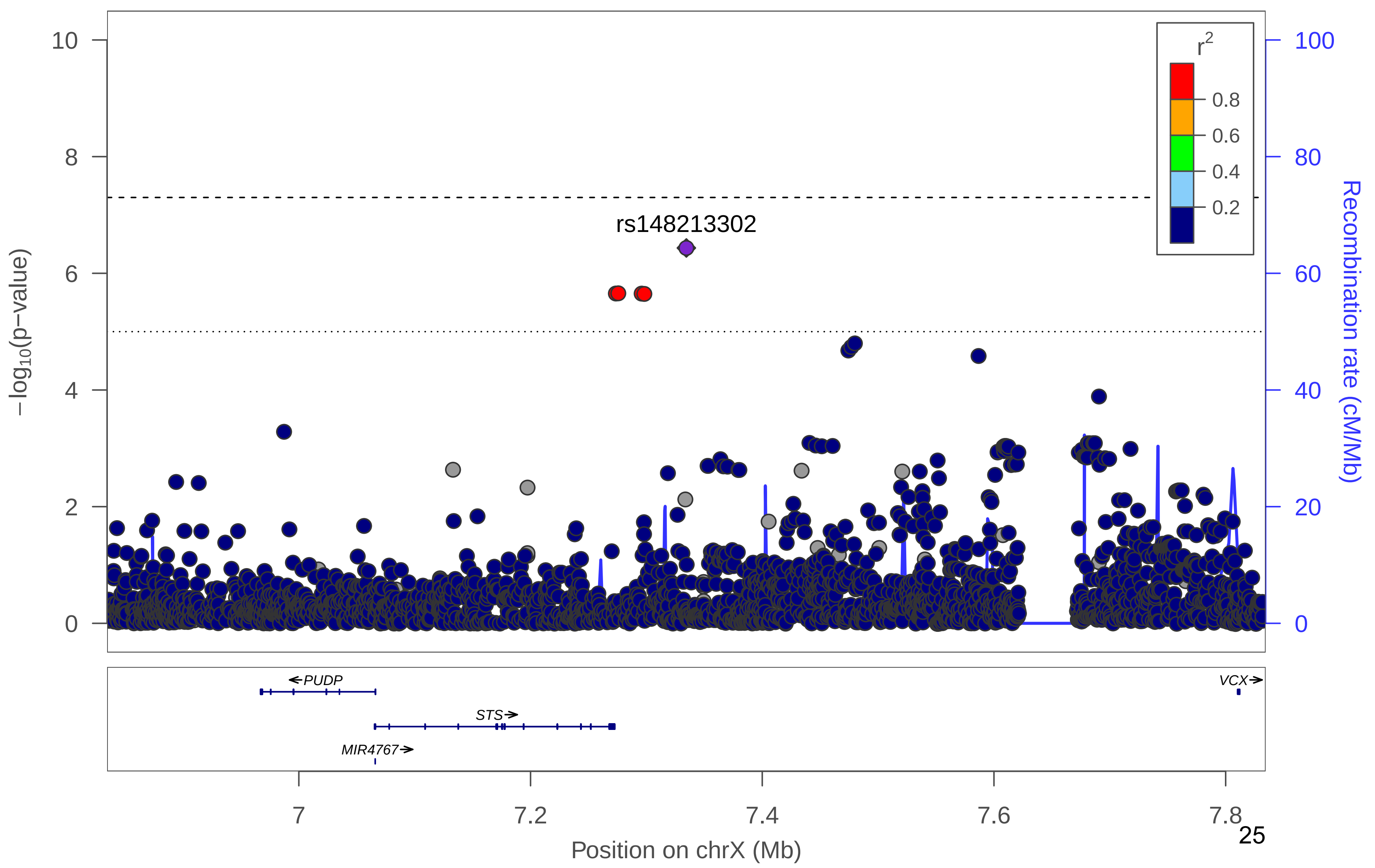

Supplementary Figure 7. QQ Plots. QQ Plots for WHRadjBMI, including sexes-combined (black), women-only (orange), men-only (blue).

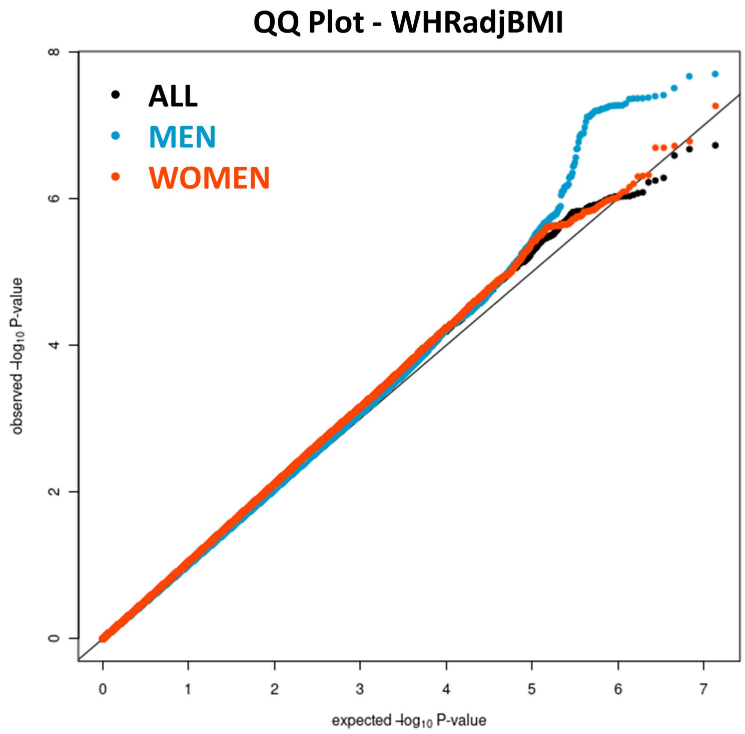

Supplementary Figure 8. Manhattan plot. Manhattan plot of the sexes-combined analysis for WCadjBMI. All suggestively significant (P<1x10[-6]) variants are highlighted in orange if they are >500 Kb from any previously-reported WCadjBMI associated variants. Previously reported loci (+/- 500 Kb) are highlighted in blue if any variant in the locus reached suggestive significance. All suggestively significant loci that meet our criteria for replication are annotated with the closest gene. †Replicated in African American meta-analysis. ‡ Replicated in Hispanic/Latino meta-analysis. ¥ Replicated in European American meta-analysis.

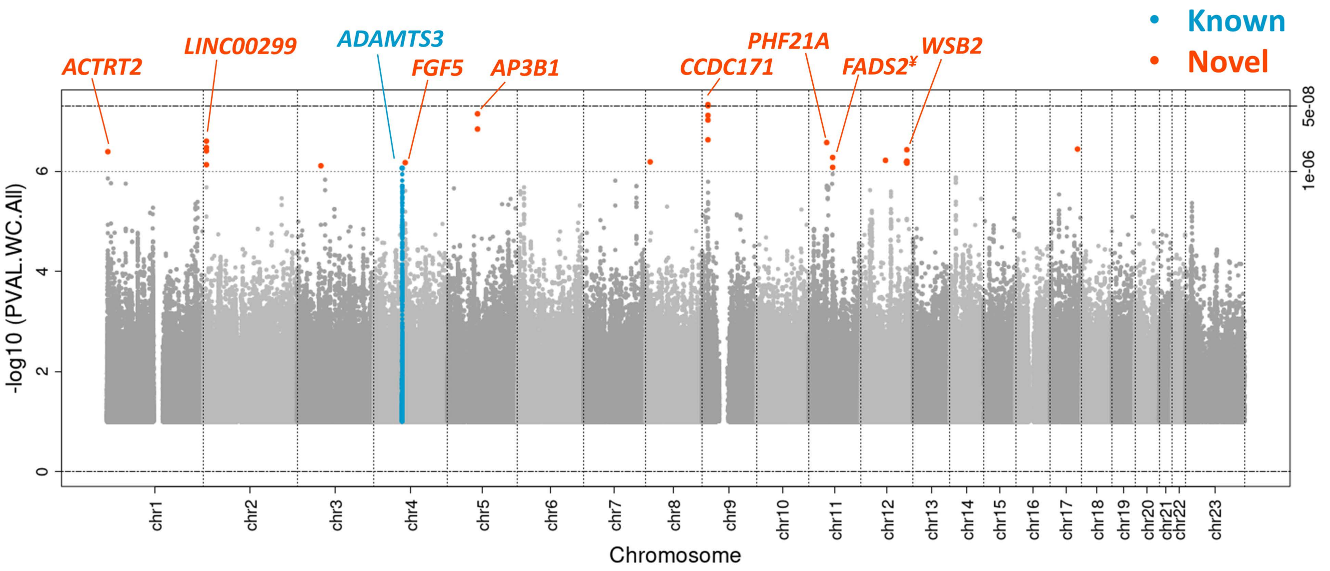

Supplementary Figure 9. Locus Zoom Plots. Regional association plots for suggestively significant loci in the HCHS/SOL WCadjBMI sexes-combined analysis. The plots appear in chromosome:position order. Dot color reflects R^2 calculated from the 1000 Genomes AMR reference dataset. Point symbols represent variant functional classifications: a) rs77993329, *ACTRT2*; b) rs138819965, *LINC00299*; c) rs77319470, *ADAMTS3*; d) rs921999, *FGF5*; e) rs112404395, *AP3B1*; f) rs77264633, *CCDC171*; g) rs184067184, *PHF21A*; h) rs3168072, *FADS2*; i) rs60260780, *WSB2*.

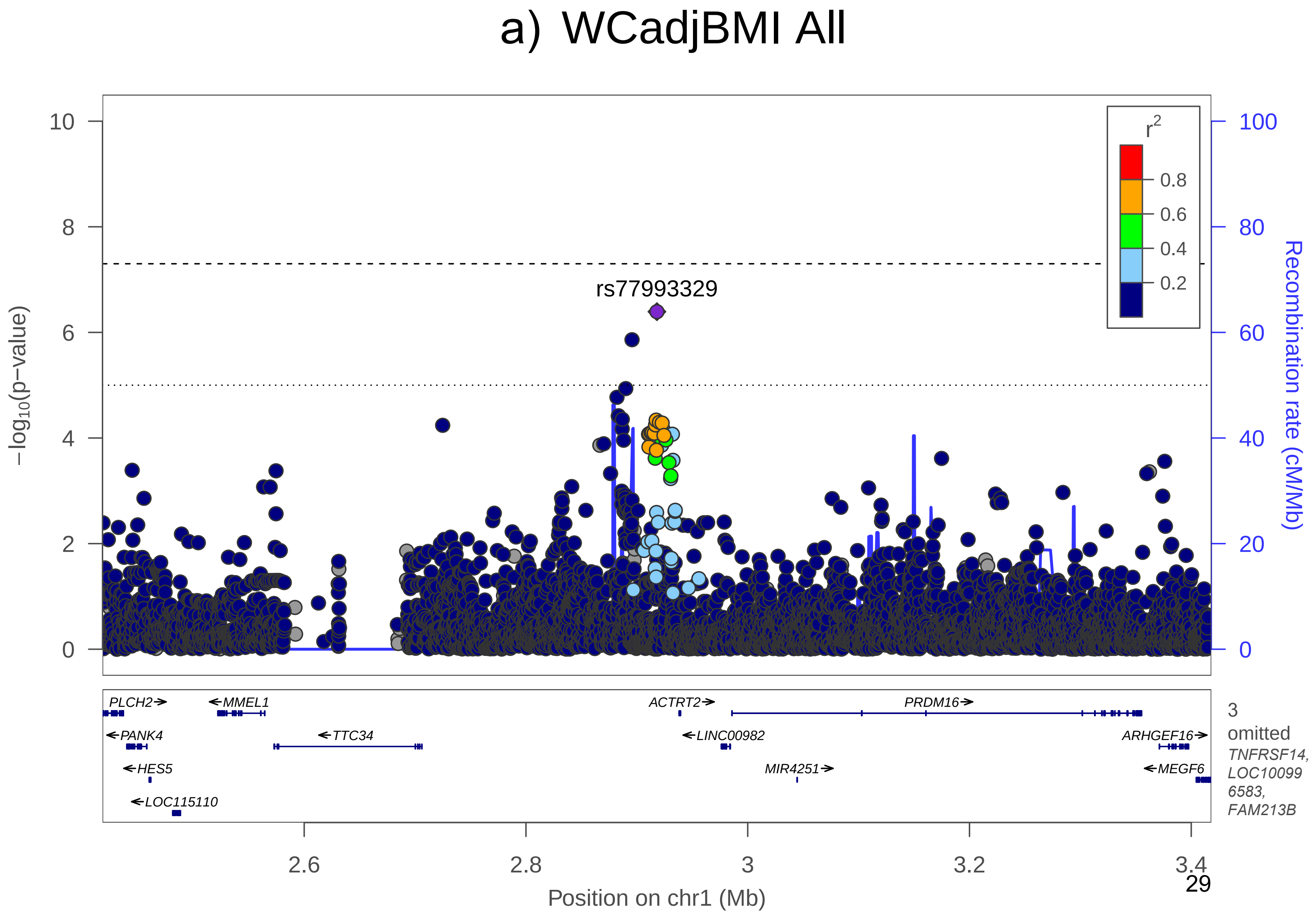

a) WCadjBMI All
rs77993329
r2
0.8
0.6
0.4
0.2
−log10(p−value)
Recombination rate (cM/Mb)
PLCH2
MMEL1
ACTRT2
PRDM16
PANK4
TTC34
LINC00982
ARHGEF16
HES5
MIR4251
MEGF6
LOC115110
3 omitted
TNFRSF14, LOC100996583, FAM213B
2.6
2.8
3
3.2
3.4
Position on chr1 (Mb)

b) WCadjBMI All

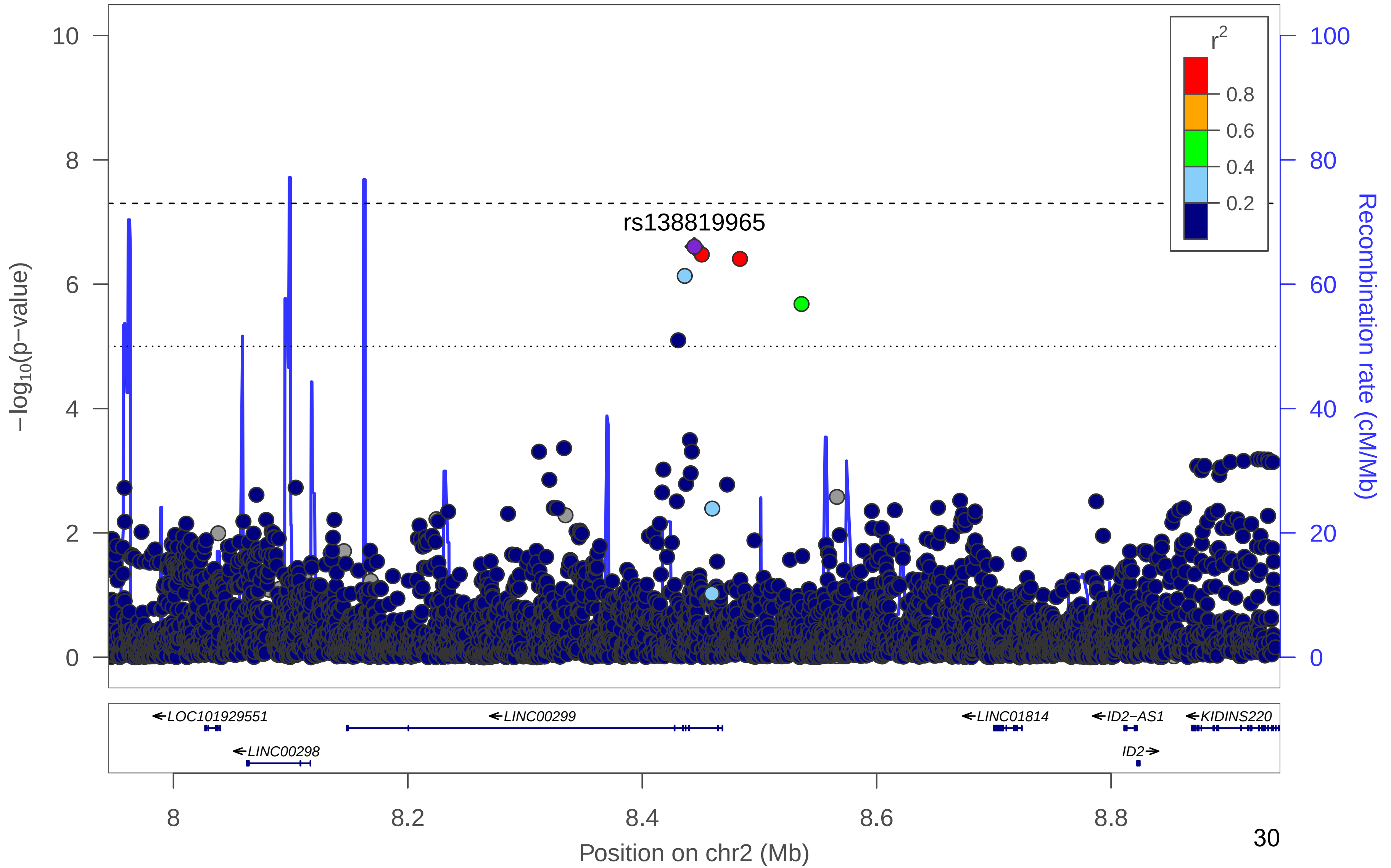

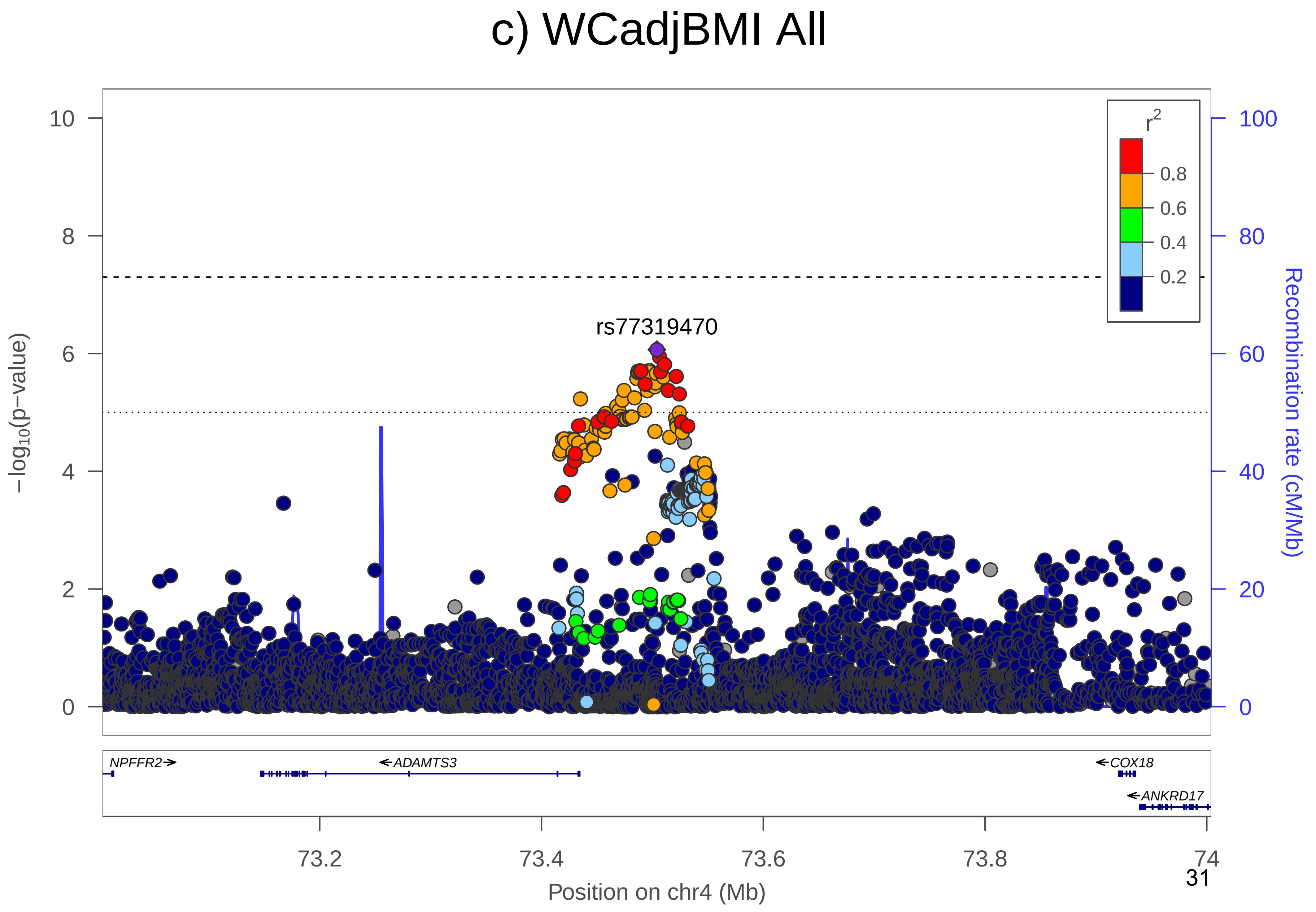
c) WCadjBMI All
rs77319470
r2
0.8
0.6
0.4
0.2
−log10(p−value)
Recombination rate (cM/Mb)
10
8
6
4
2
0
100
80
60
40
20
0
NPFFR2
ADAMTS3
COX18
ANKRD17
73.2
73.4
73.6
73.8
74
Position on chr4 (Mb)

d) WCadjBMI All

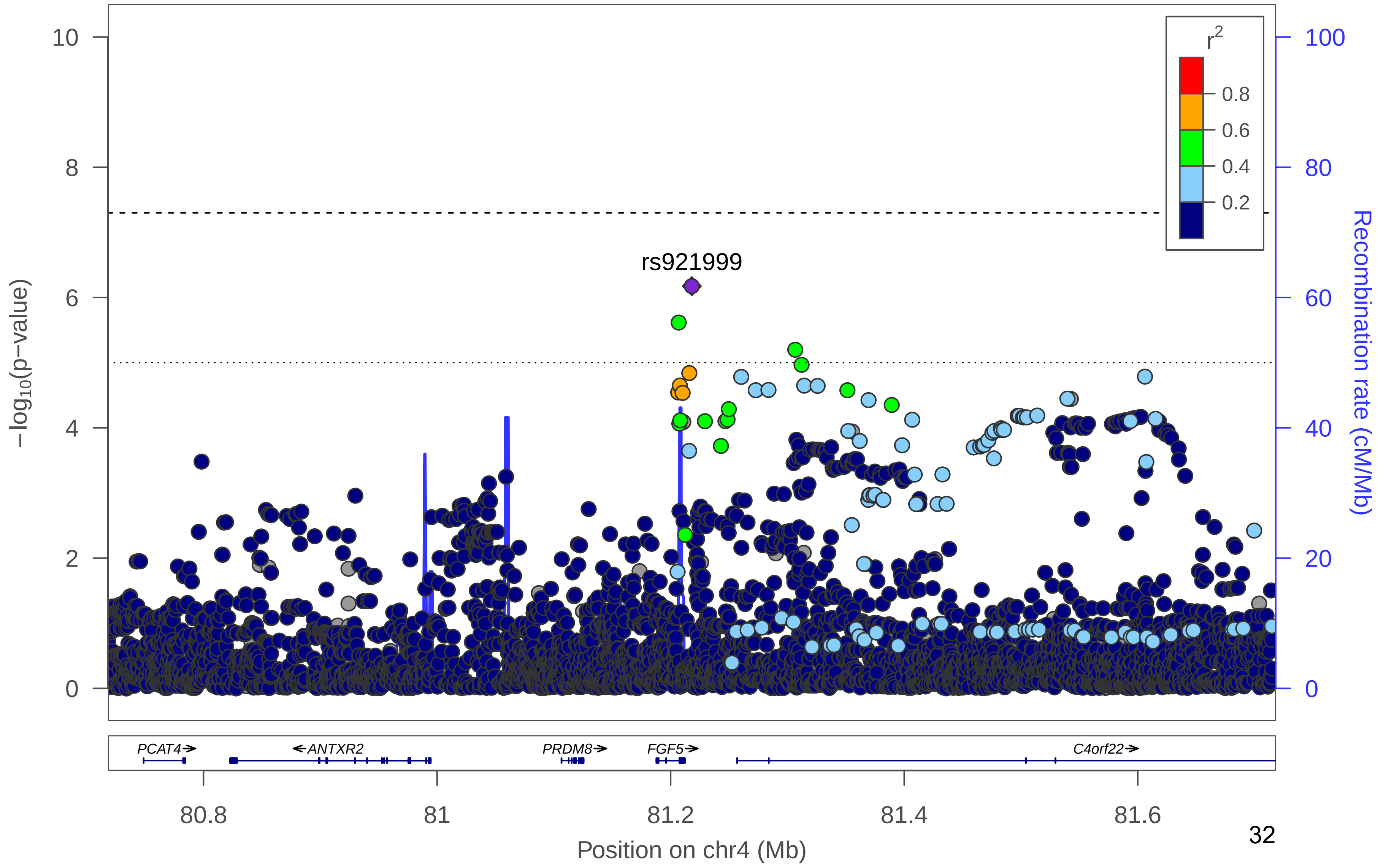

e) WCadjBMI All

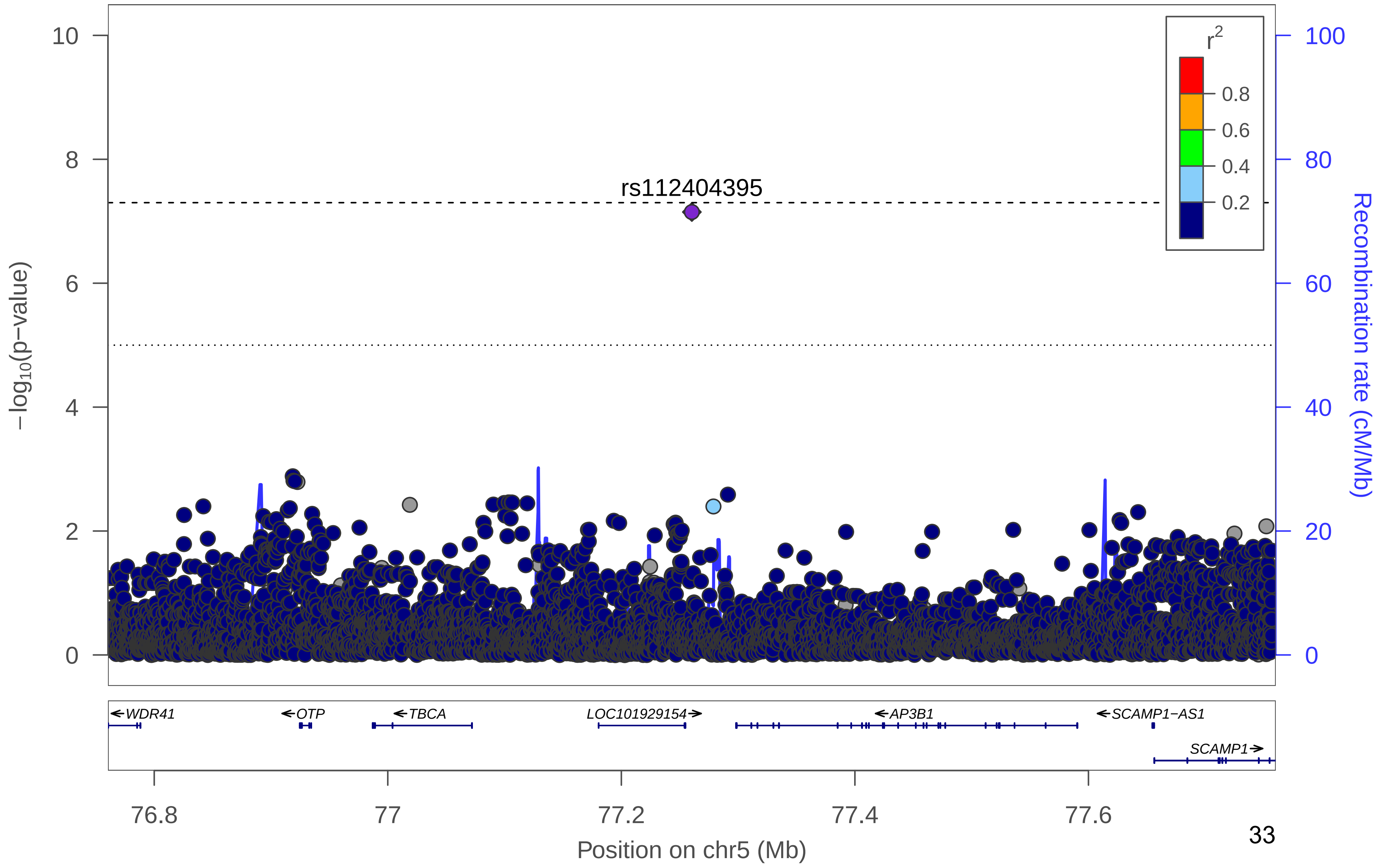

f) WCadjBMI All

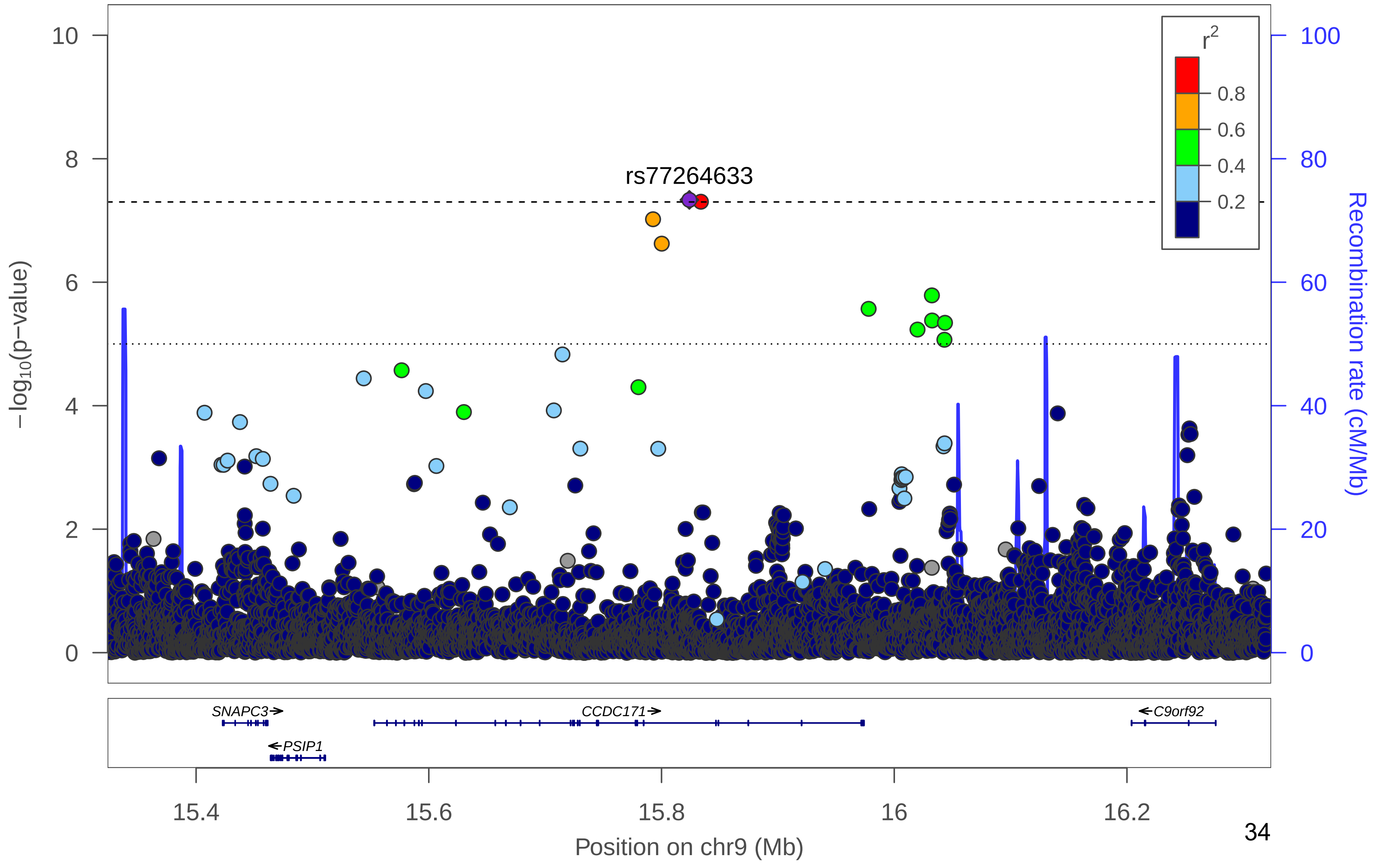

g) WCadjBMI All

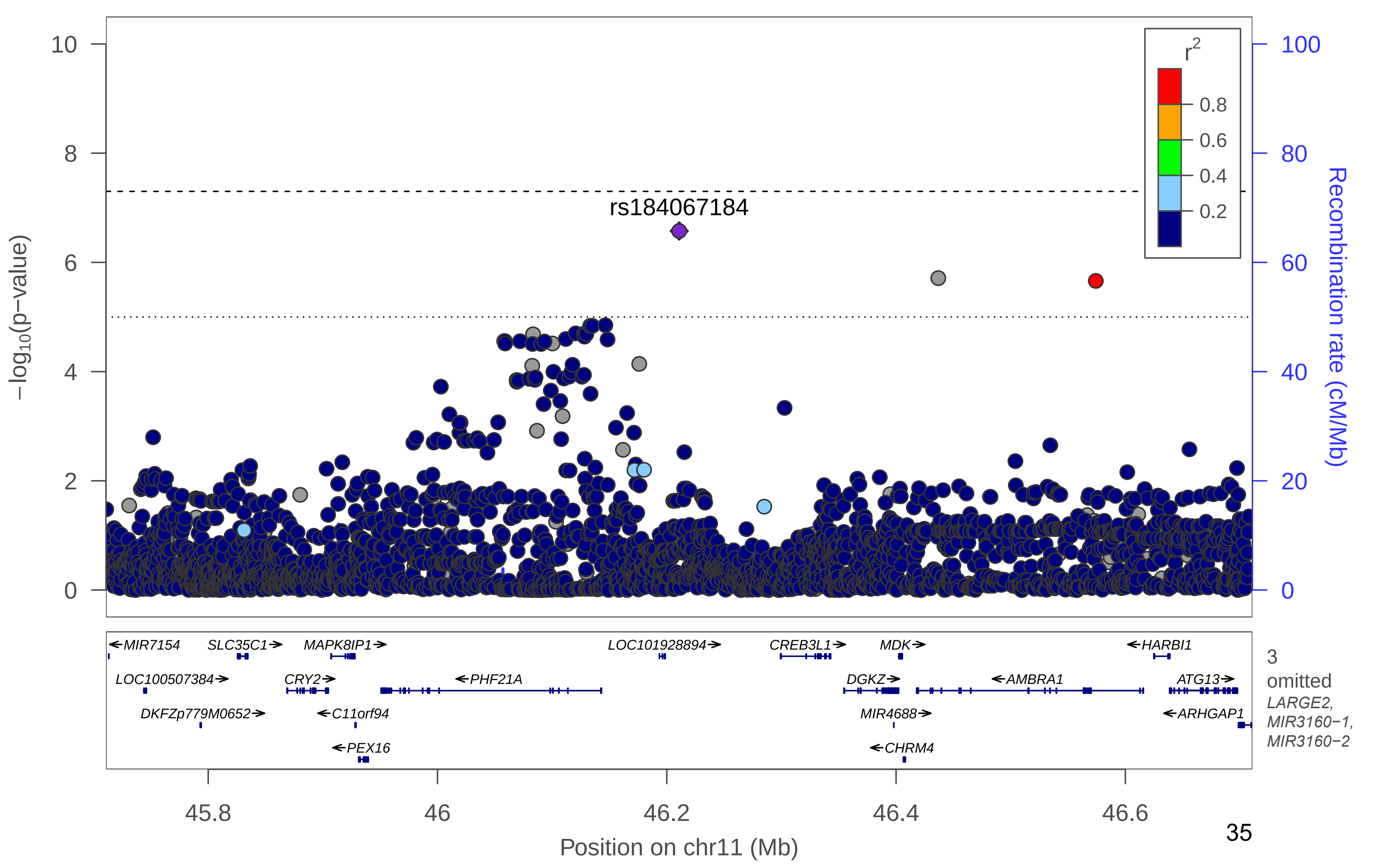

h) WCadjBMI All

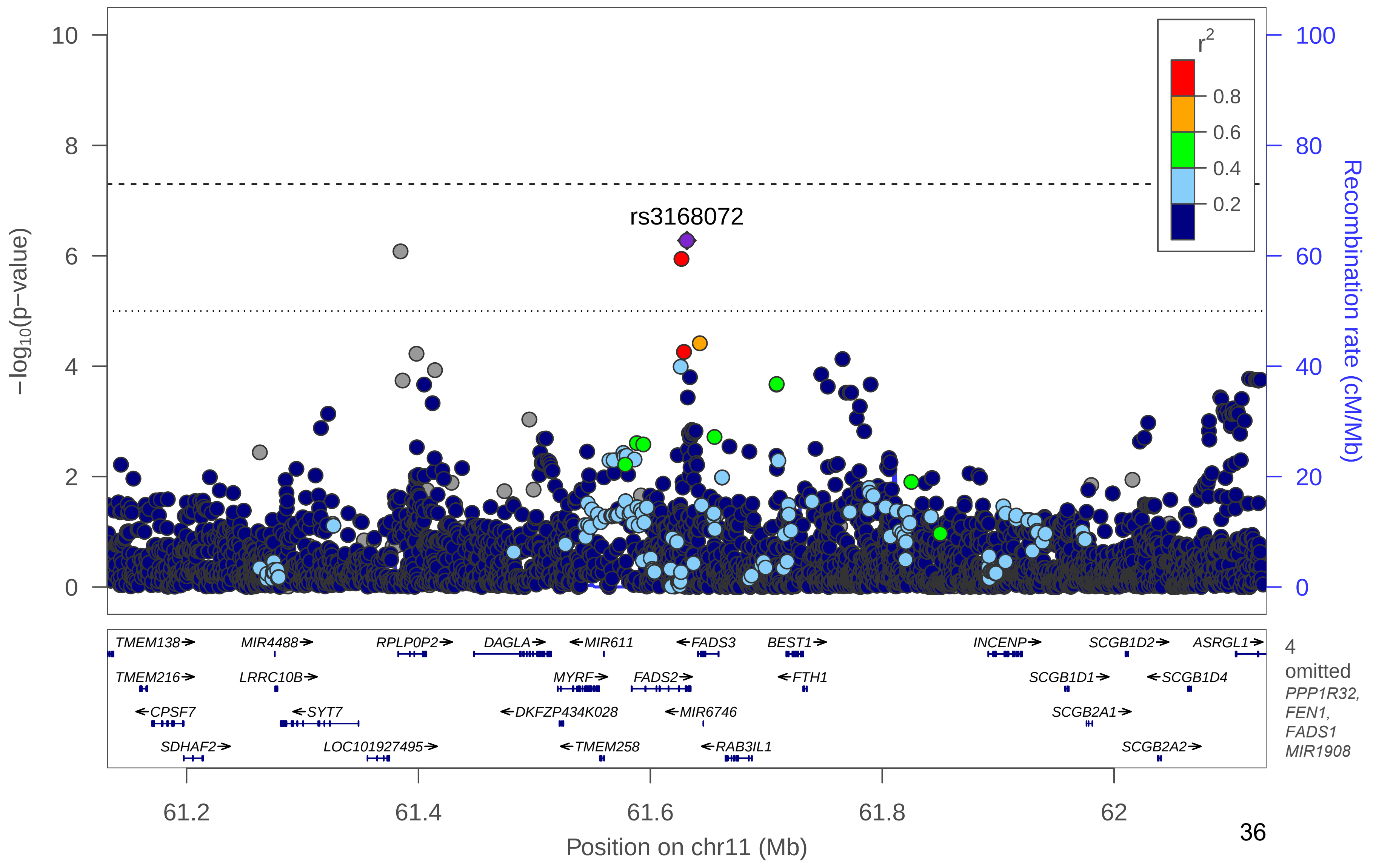

i) WCadjBMI All

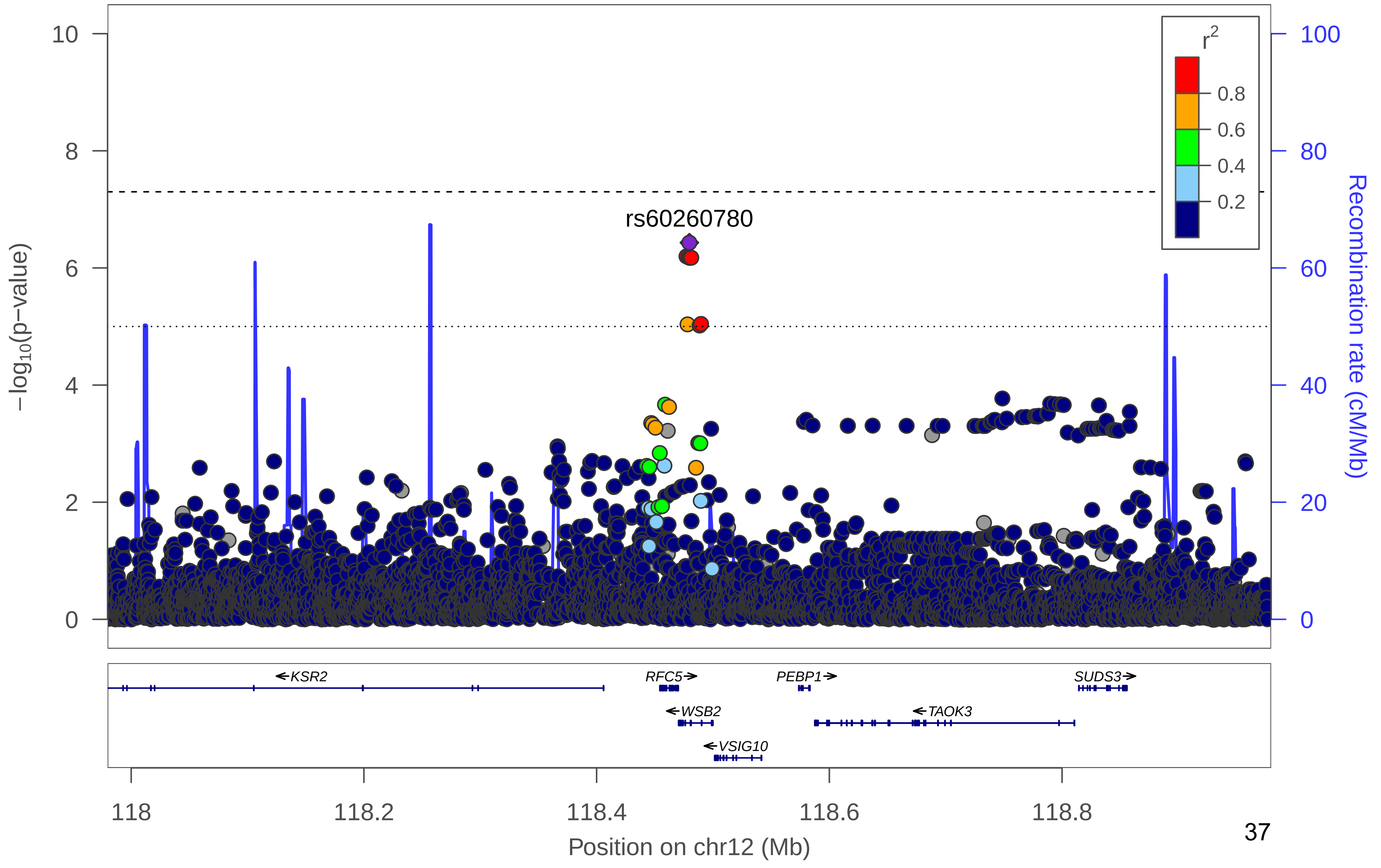

Supplementary Figure 10. Manhattan plot. Manhattan plot of the women-only analysis for WCadjBMI. All suggestively significant (*P*<1x10^{-6}) variants are highlighted in orange if they are >500 Kb from any previously-reported WCadjBMI associated variants. Previously reported loci (+/- 500 Kb) are highlighted in blue if any variant in the locus reached suggestive significance. All suggestively significant loci that meet our criteria for replication are annotated with the closest gene. †Replicated in African American meta-analysis. ‡ Replicated in Hispanic/Latino meta-analysis. ¥ Replicated in European American meta-analysis.

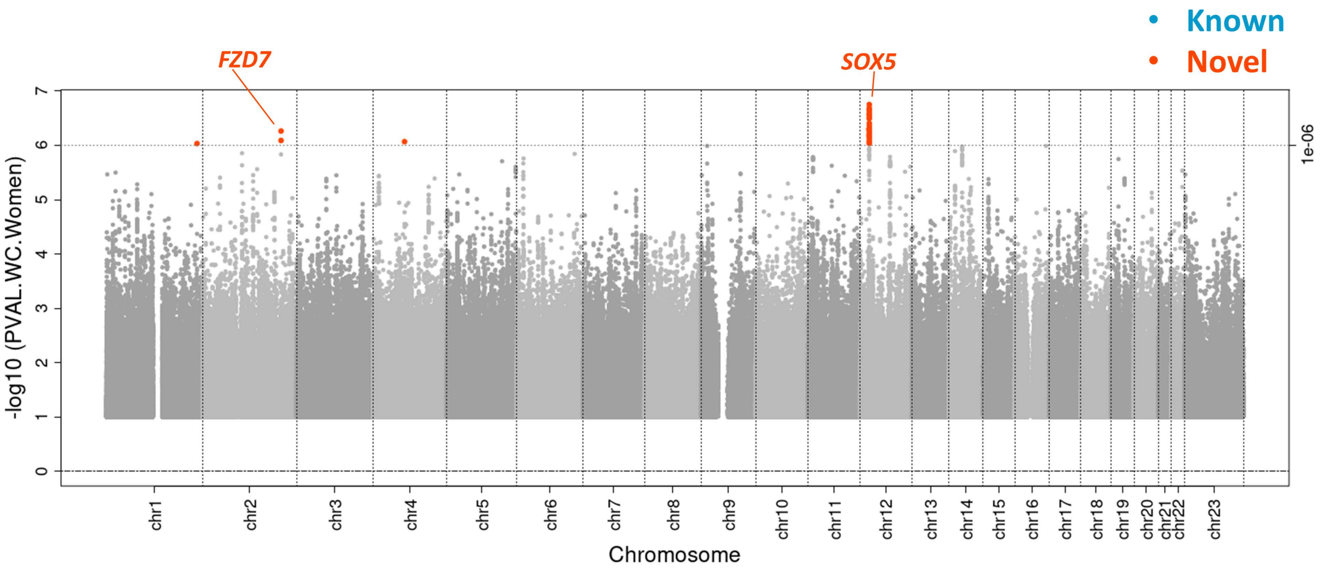

Supplementary Figure 11. Locus Zoom Plots. Regional association plots for suggestively significant loci in the HCHS/SOL WCadjBMI women-only analysis. The plots appear in chromosome:position order. Dot color reflects R^2 calculated from the 1000 Genomes AMR reference dataset. Point symbols represent variant functional classifications: a) rs112469617, *FZD7*; b) rs17385466, *SOX5*.

a) WCadjBMI Women

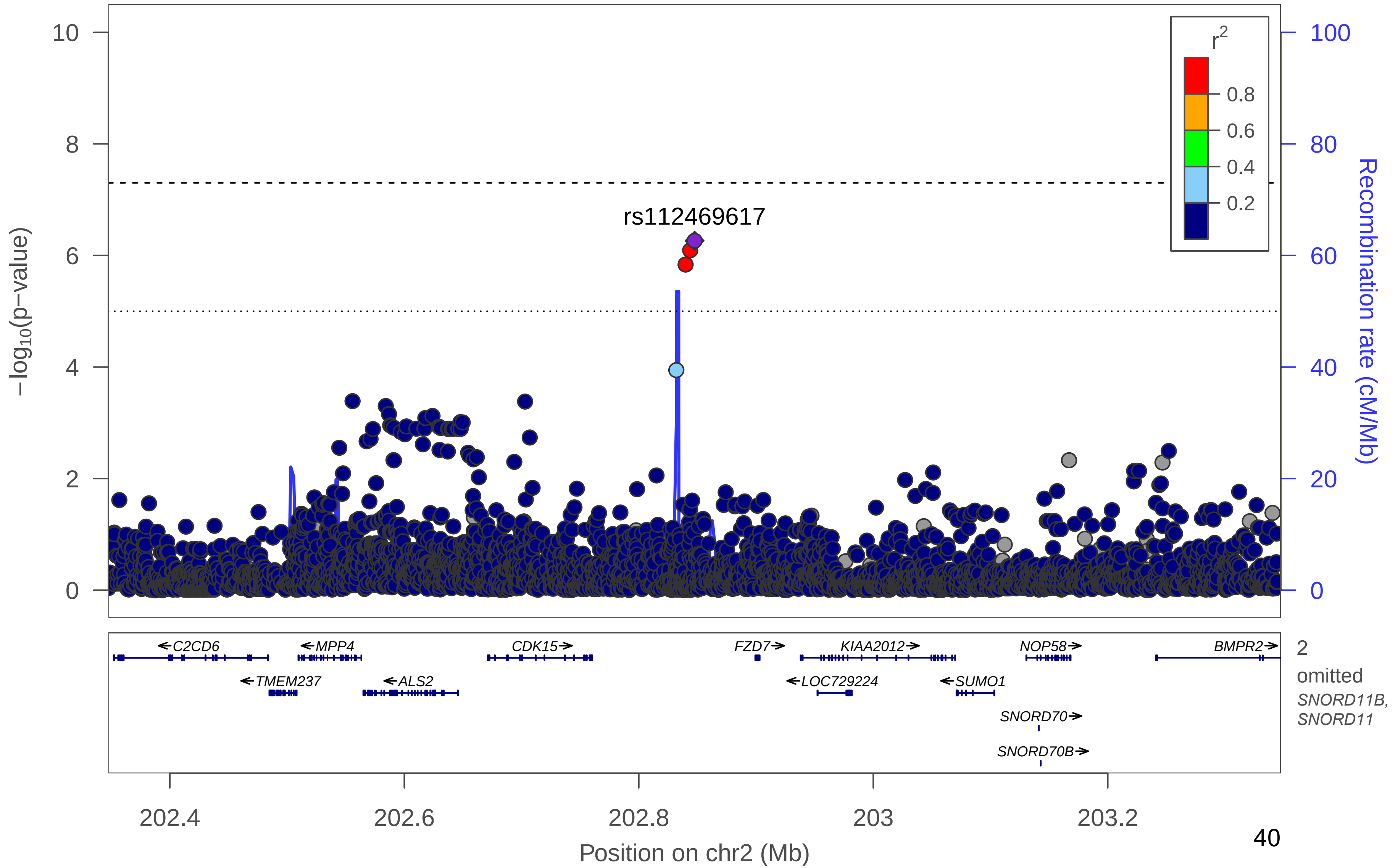

b) WCadjBMI Women

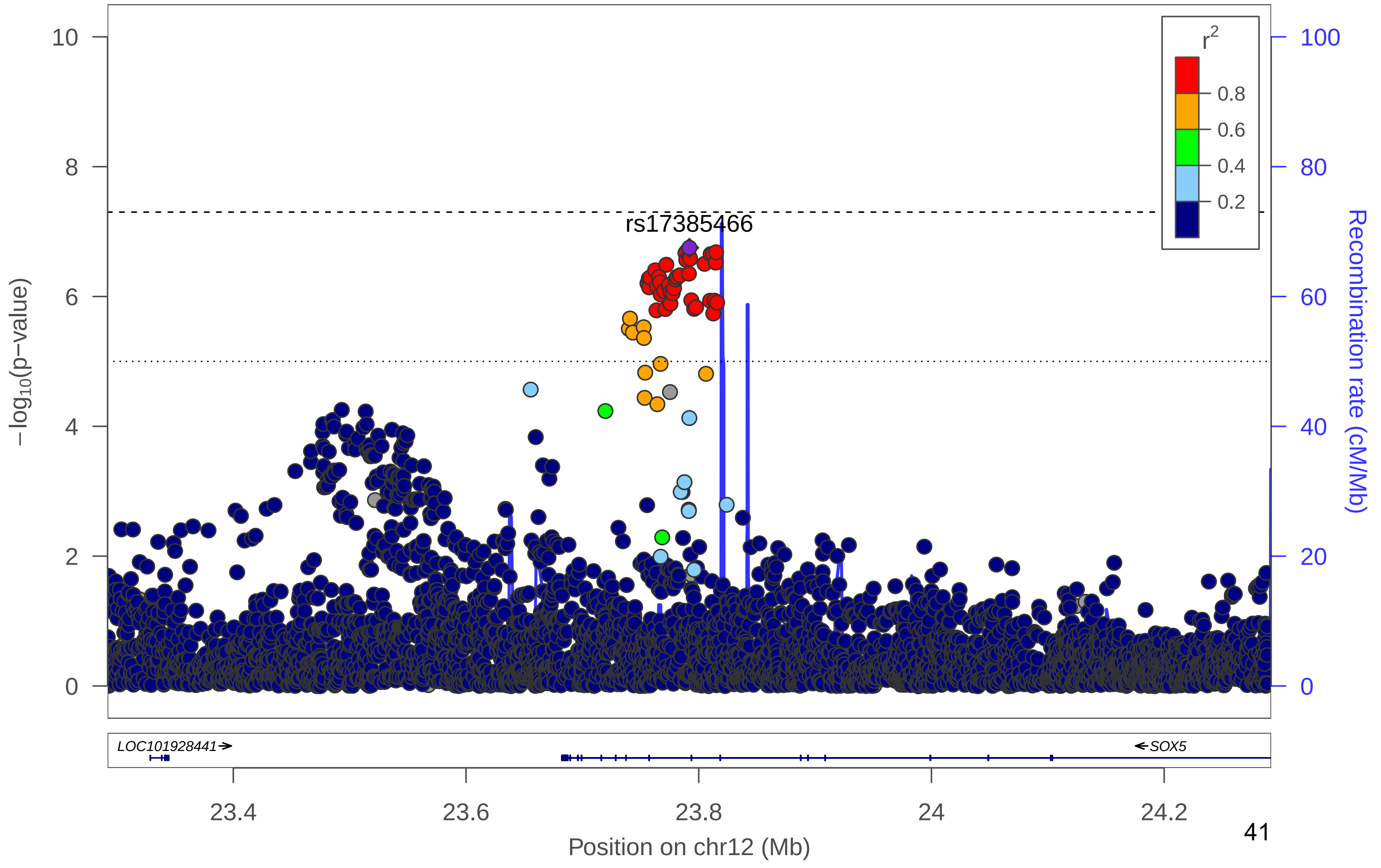

Supplementary Figure 12. Manhattan plot. Manhattan plot of the men-only analysis for WCadjBMI. All suggestively significant (*P*<1x10^{-6}) variants are highlighted in orange if they are >500 Kb from any previously-reported WCadjBMI associated variants. Previously reported loci (+/- 500 Kb) are highlighted in blue if any variant in the locus reached suggestive significance. All suggestively significant loci that meet our criteria for replication are annotated with the closest gene. †Replicated in African American meta-analysis. ‡ Replicated in Hispanic/Latino meta-analysis. ¥ Replicated in European American meta-analysis.

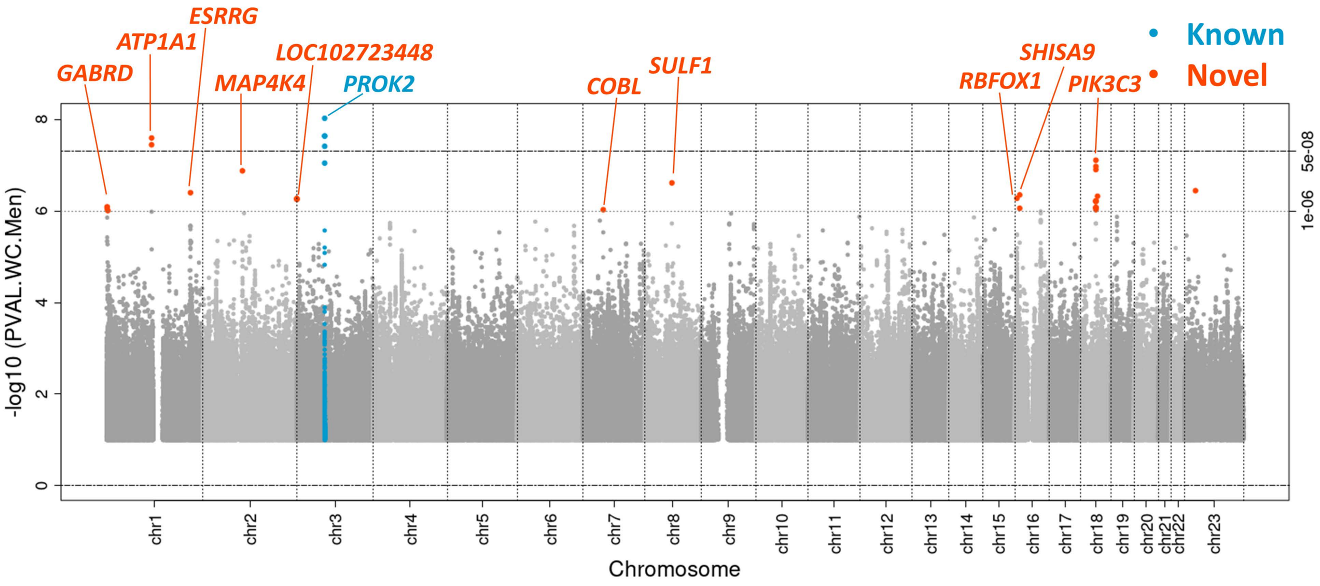

Supplementary Figure 13. Locus Zoom Plots. Regional association plots for suggestively significant loci in the HCHS/SOL WCadjBMI men-only analysis. The plots appear in chromosome:position order. Dot color reflects R^2 calculated from the 1000 Genomes AMR reference dataset. Point symbols represent variant functional classifications: a) rs74346221, *GABRD*; b) rs72693785, *ATP1A1*; c) rs11583298, *ESRRG*; d) rs76842062, *MAP4K4*; e) rs141365360, *LOC102723448*; f) rs6809759, *PROK2*; g) rs76941364, *COBL*; h) rs139139519, *SULF1*; i) rs35569658, *RBFOX1*; j) rs148280037, *SHISA9*; k) rs143565319, *PIK3C3*.

a) WCadjBMI Men

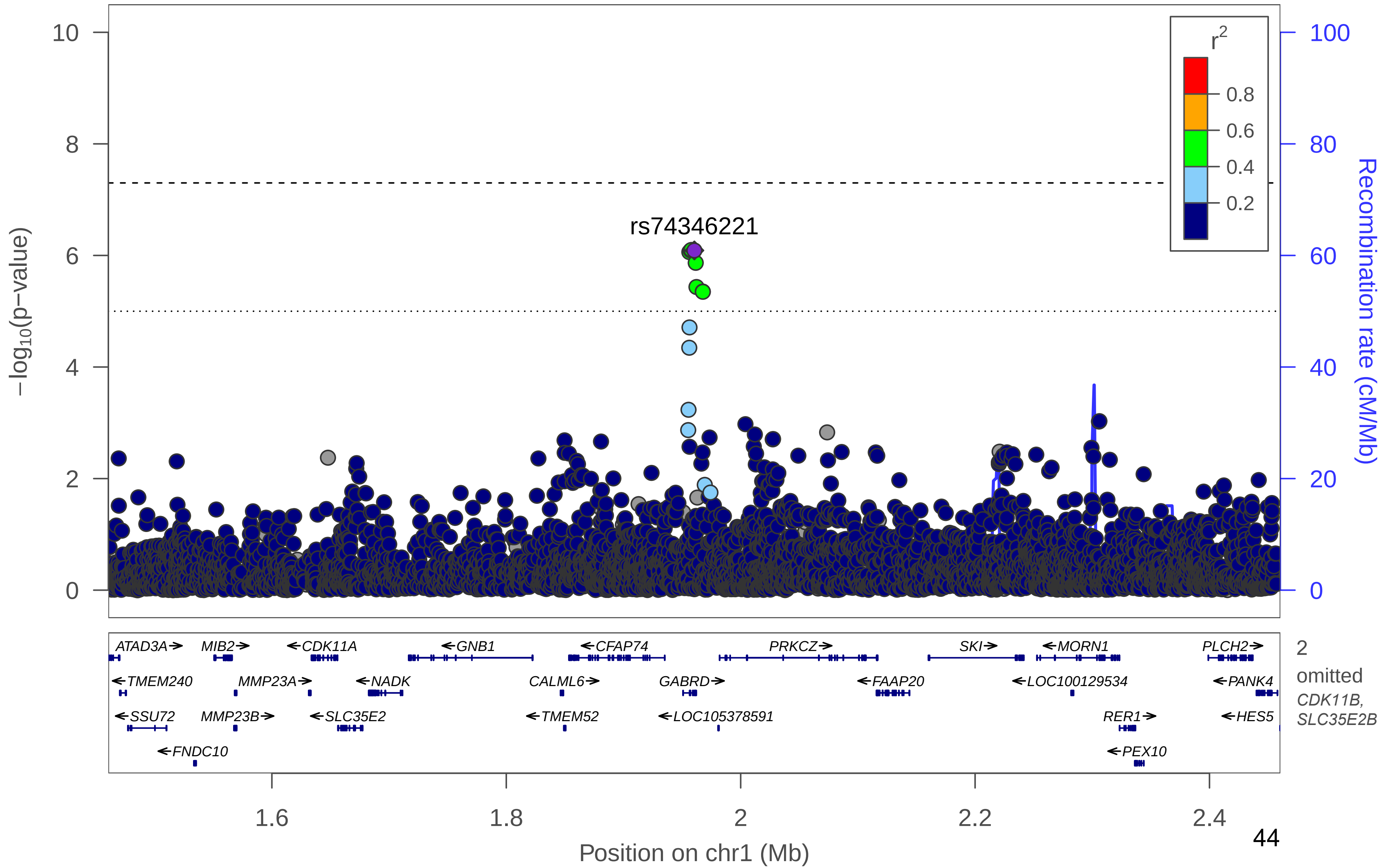

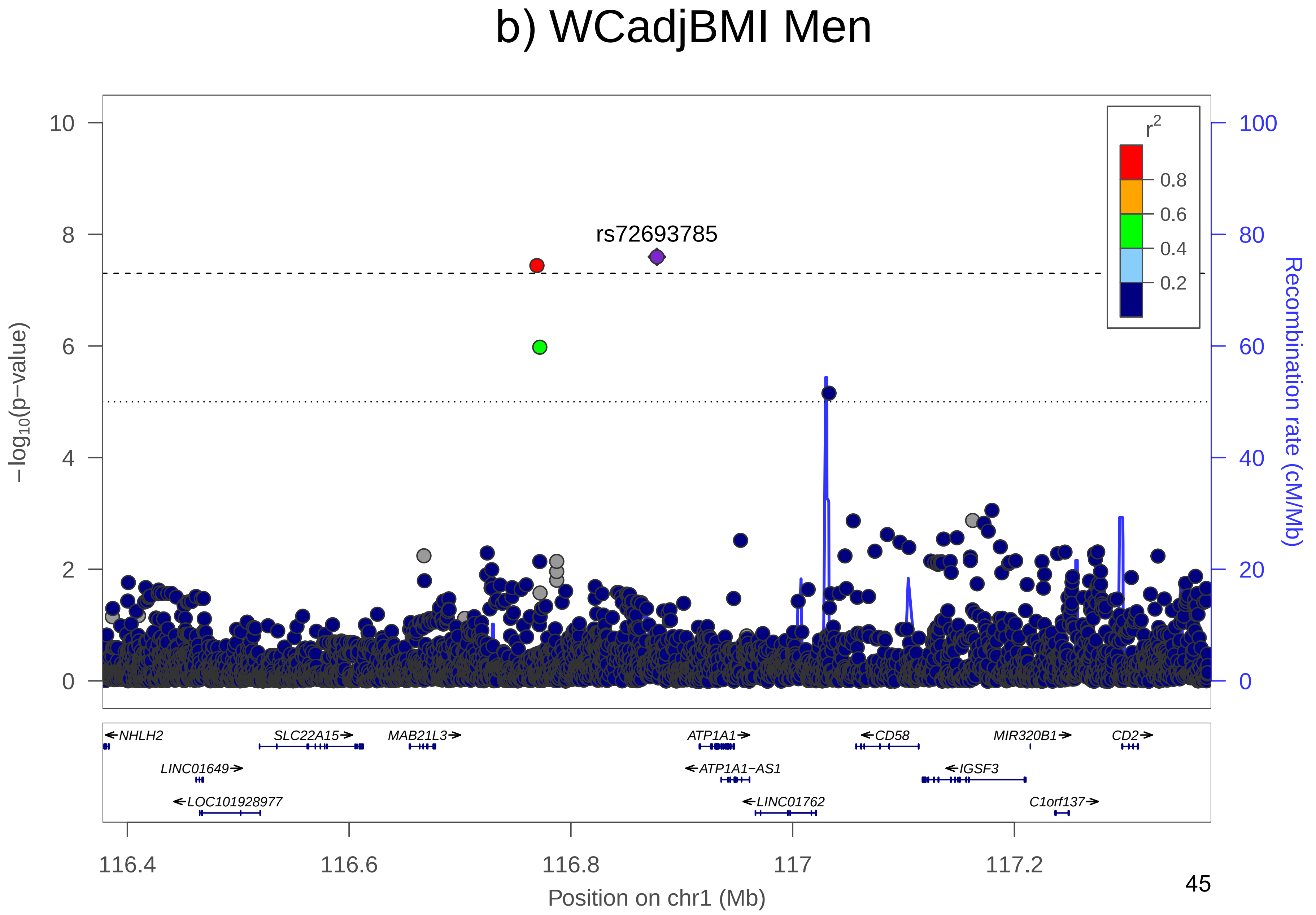
b) WCadjBMI Men
rs72693785
r2
0.8
0.6
0.4
0.2
−log10(p−value)
Recombination rate (cM/Mb)
NHLH2
SLC22A15
MAB21L3
ATP1A1
CD58
MIR320B1
CD2
LINC01649
ATP1A1−AS1
IGSF3
LOC101928977
LINC01762
C1orf137
116.4
116.6
116.8
117
117.2
Position on chr1 (Mb)

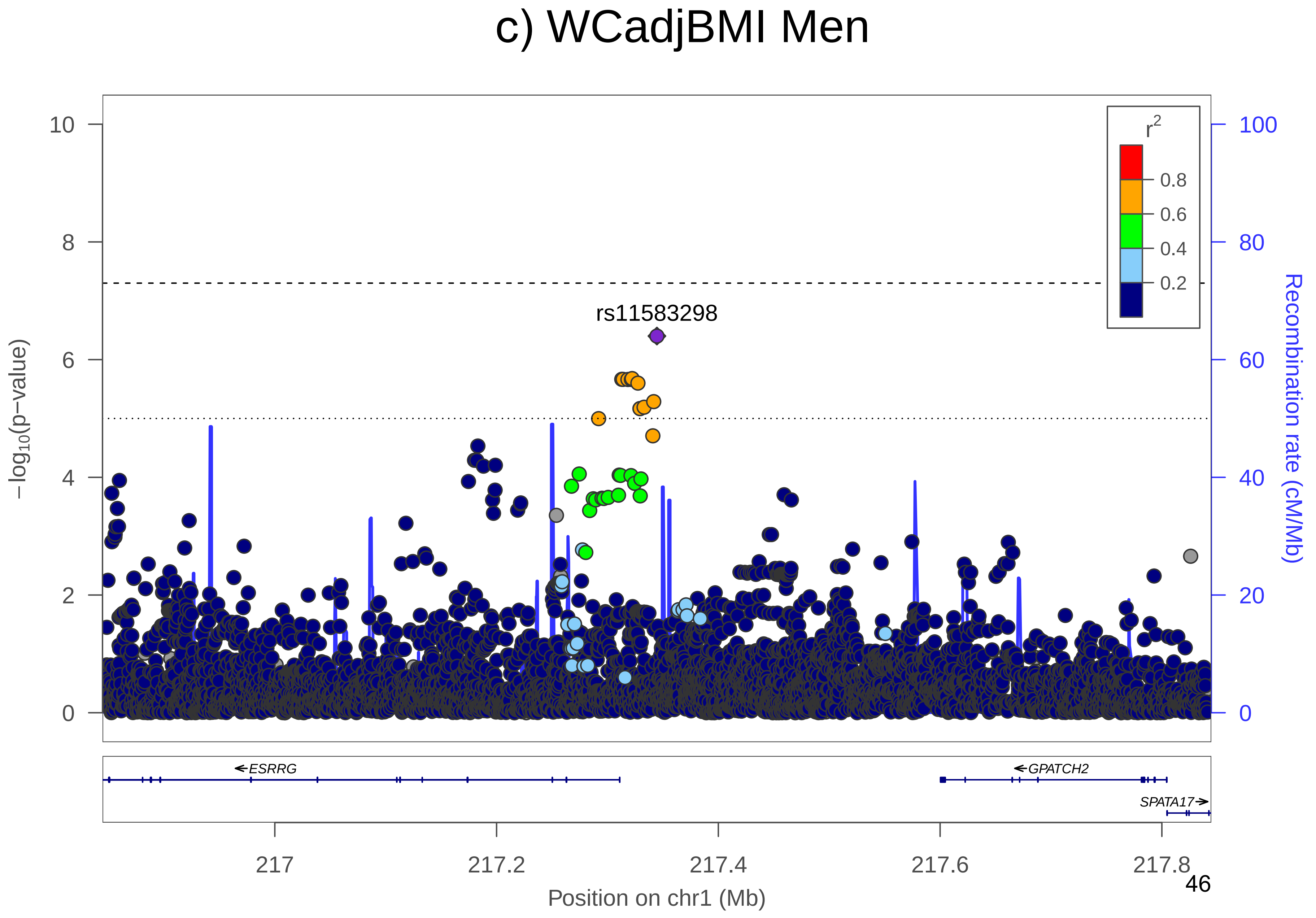
c) WCadjBMI Men
rs11583298
r2
0.8
0.6
0.4
0.2
−log10(p−value)
Recombination rate (cM/Mb)
ESRRG
GPATCH2
SPATA17
217
217.2
217.4
217.6
217.8
Position on chr1 (Mb)

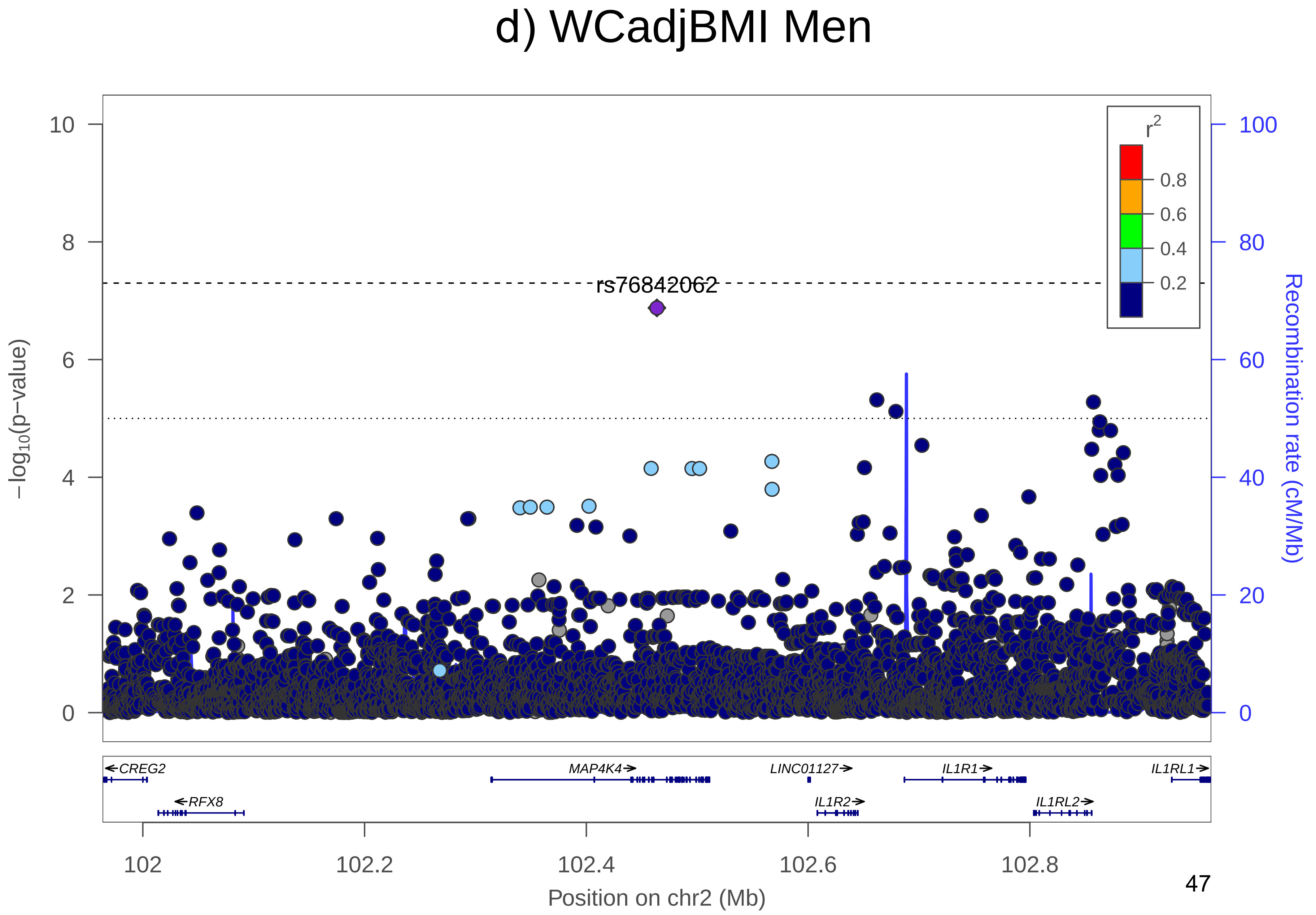

d) WCadjBMI Men
rs76842062
r2
0.8
0.6
0.4
0.2
−log10(p−value)
Recombination rate (cM/Mb)
10
8
6
4
2
0
100
80
60
40
20
0
CREG2
RFX8
MAP4K4
LINC01127
IL1R2
IL1R1
IL1RL2
IL1RL1
102
102.2
102.4
102.6
102.8
Position on chr2 (Mb)

e) WCadjBMI Men

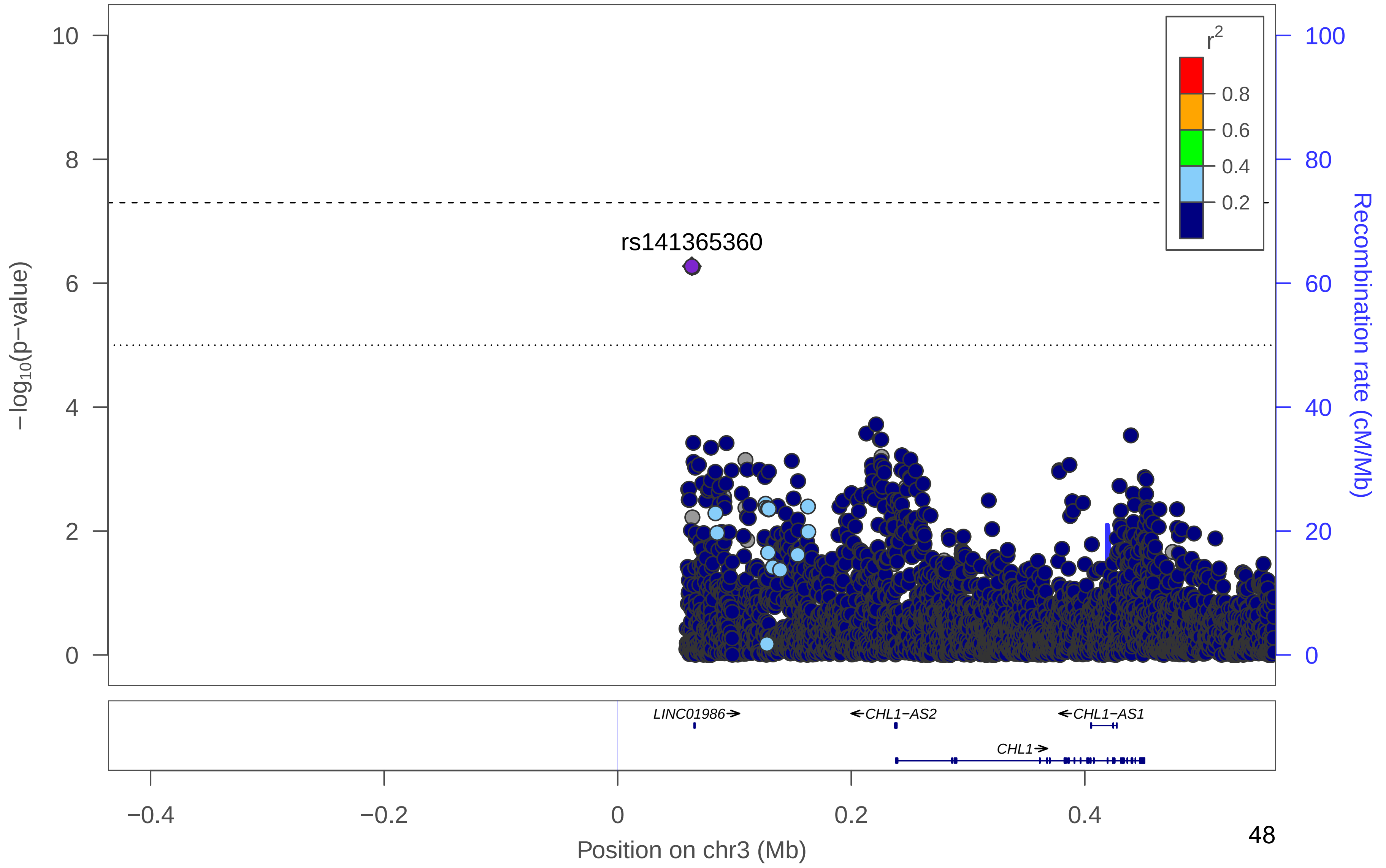

f) WCadjBMI Men

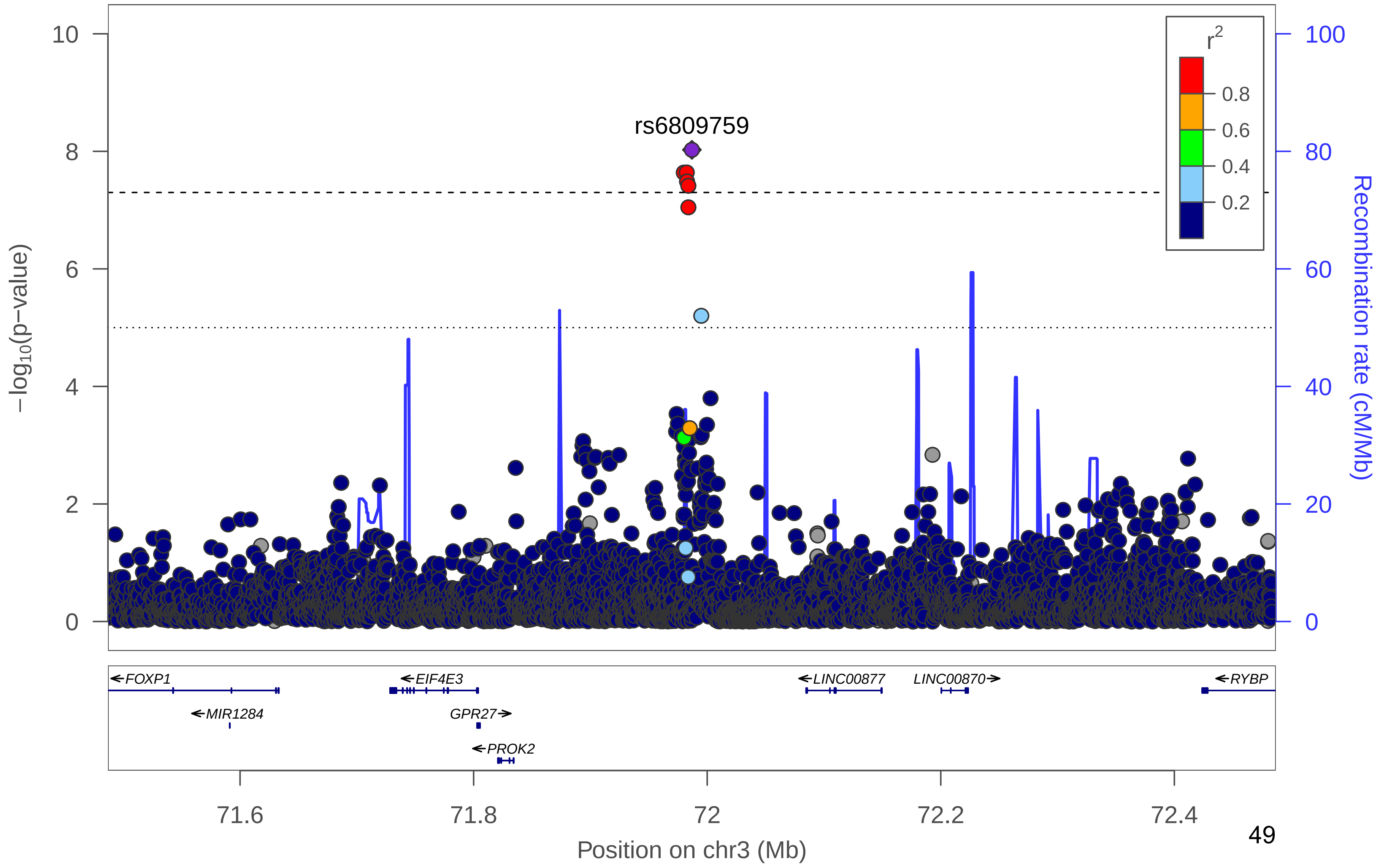

g) WCadjBMI Men

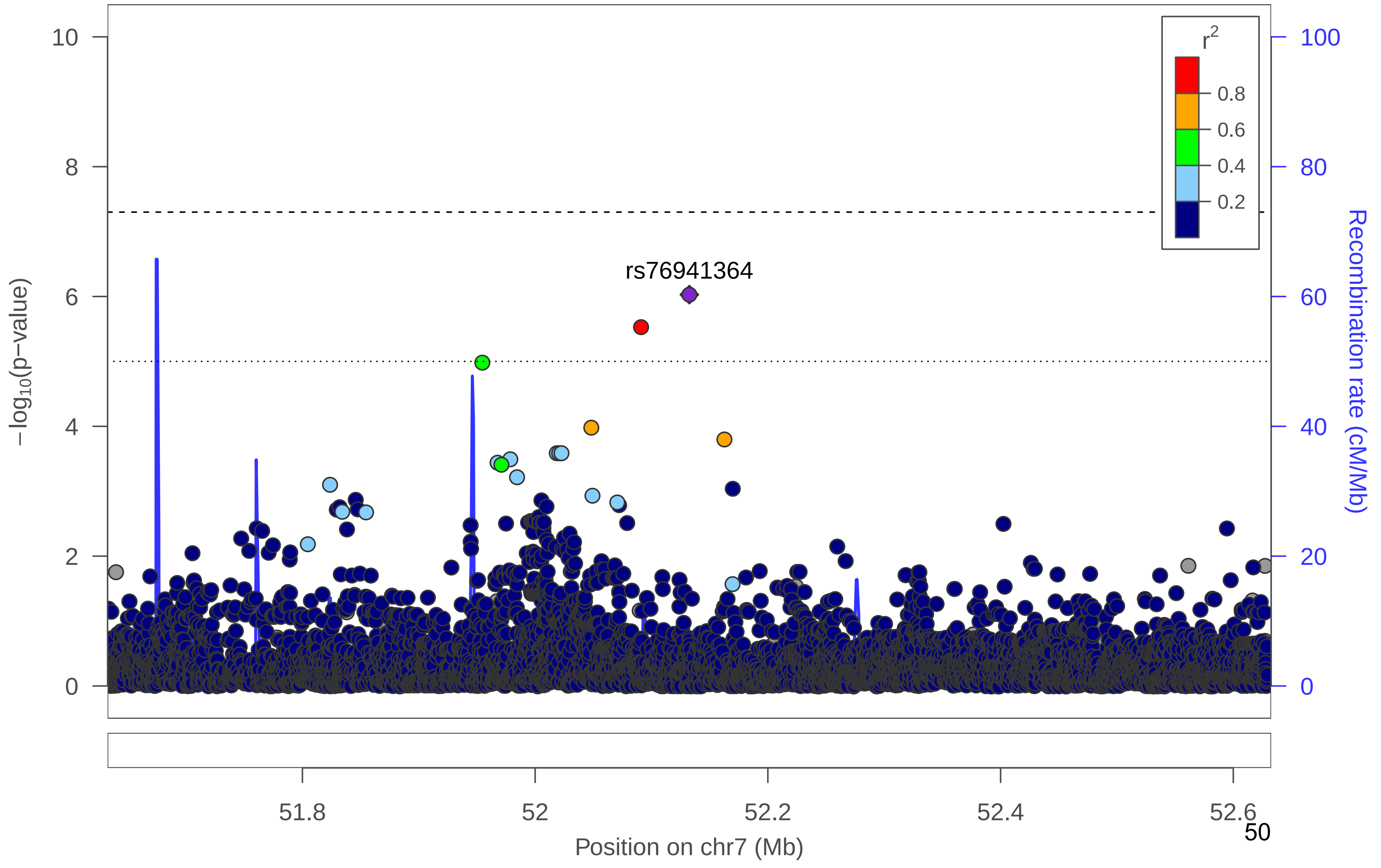

h) WCadjBMI Men

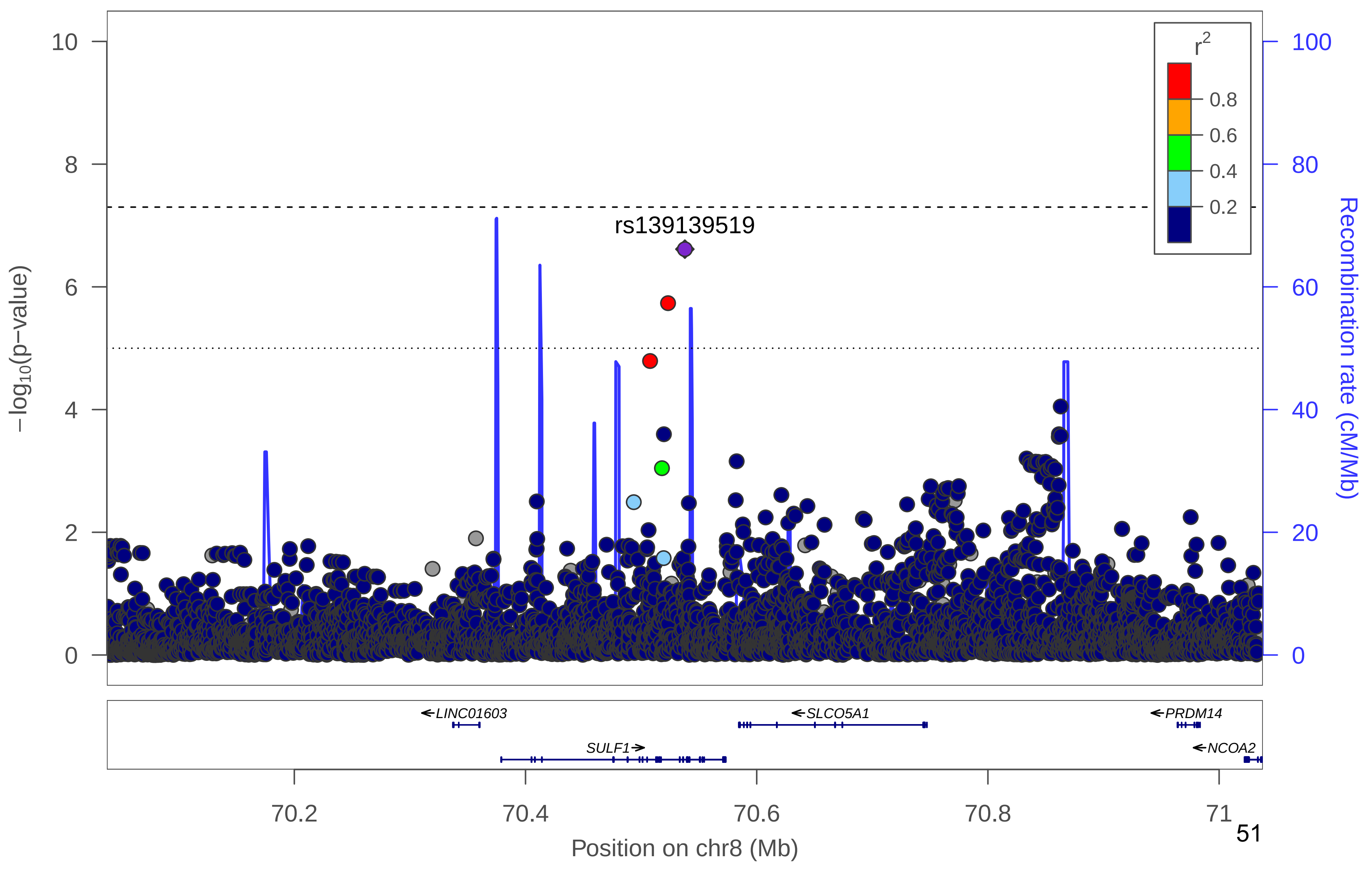

i) WCadjBMI Men

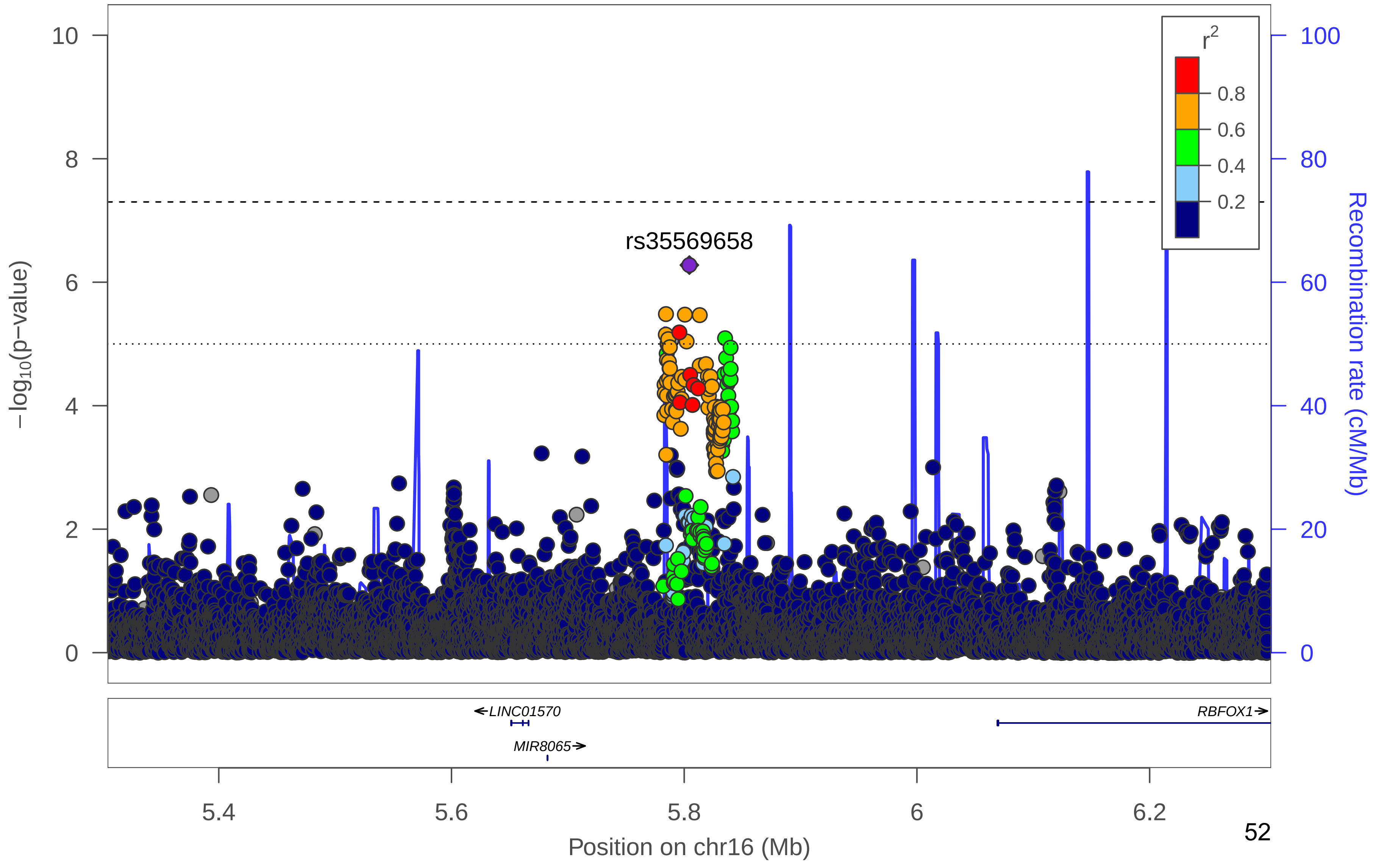

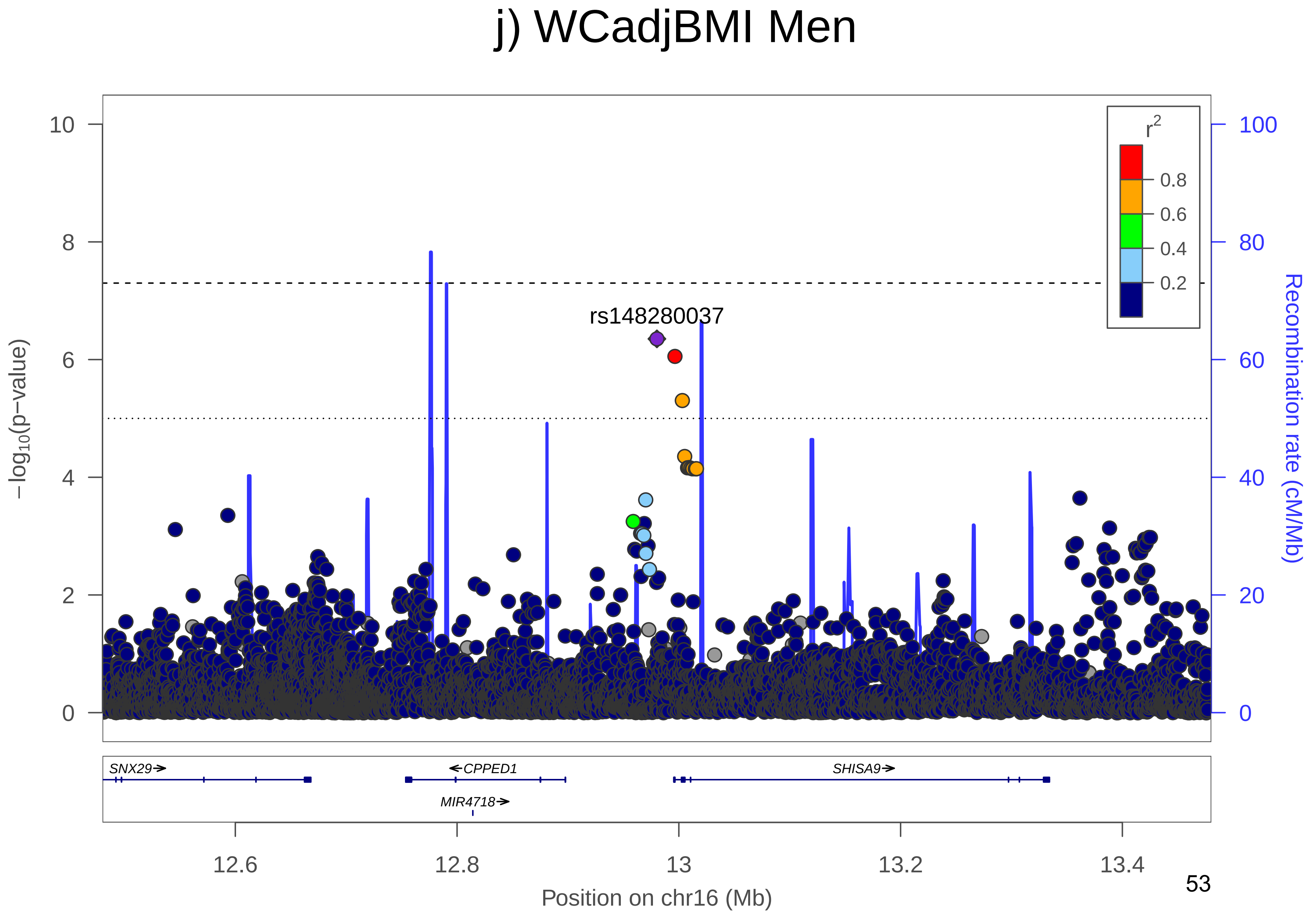
j) WCadjBMI Men
rs148280037
r²
0.8
0.6
0.4
0.2
−log10(p−value)
Recombination rate (cM/Mb)
10
8
6
4
2
0
100
80
60
40
20
0
SNX29
CPPED1
SHISA9
MIR4718
12.6
12.8
13
13.2
13.4
Position on chr16 (Mb)

k) WCadjBMI Men

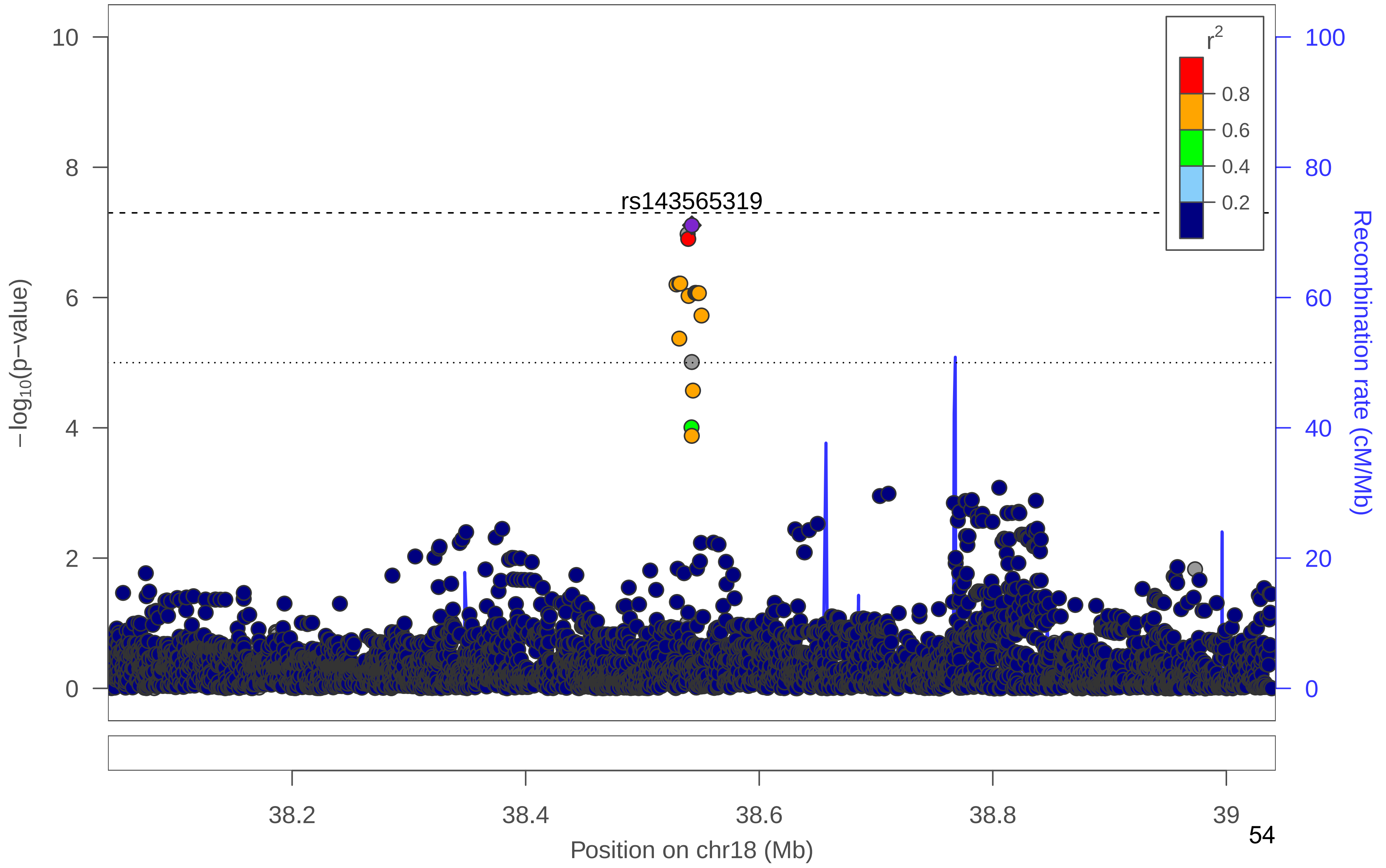

Supplementary Figure 14. QQ Plots. QQ Plots for WCadjBMI, including sexes-combined (black), women-only (orange), men-only (blue).

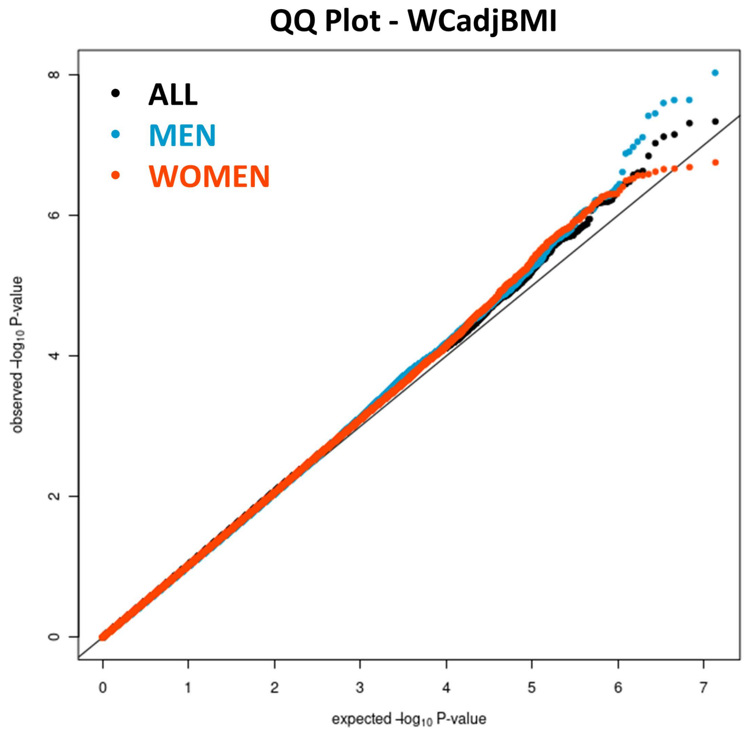

Supplementary Figure 15. Manhattan plot. Manhattan plot of the sexes-combined analysis for HIPadjBMI. All suggestively significant (*P*<1x10^{-6}) variants are highlighted in orange if they are >500 Kb from any previously-reported HIPadjBMI associated variants. Previously reported loci (+/- 500 Kb) are highlighted in blue if any variant in the locus reached suggestive significance. All suggestively significant loci that meet our criteria for replication are annotated with the closest gene. †Replicated in African American meta-analysis. ‡ Replicated in Hispanic/Latino meta-analysis. ¥ Replicated in European American meta-analysis.

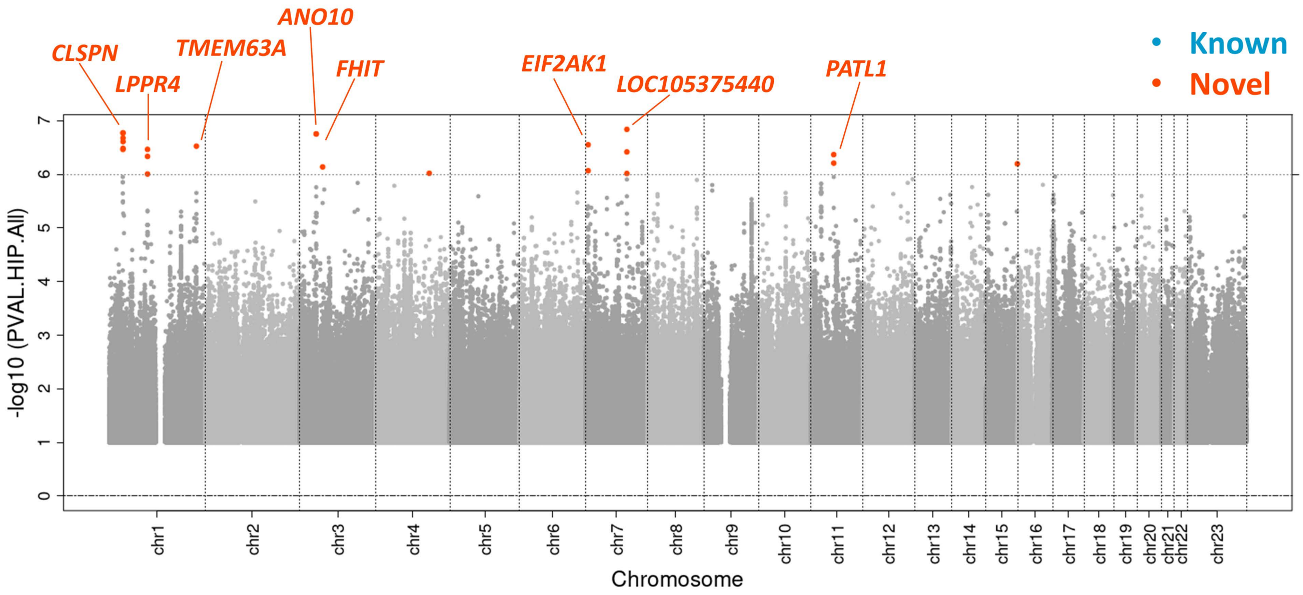

Supplementary Figure 16. Locus Zoom Plots: HIPadjBMI. Regional association plots for suggestively significant loci in the HCHS/SOL HIPadjBMI sexes-combined analysis. The plots appear in chromosome:position order. Dot color reflects R^2 calculated from the 1000 Genomes AMR reference dataset. Point symbols represent variant functional classifications: a) rs144655586, *CLSPN*; b) rs712900, *LPPR4*; c) rs115546449, *TMEM63A*; d) rs145815581, *ANO10*; e) rs72886347, *FHIT*; f) rs17136358, *EIF2AK1*; g) rs117683919, *LOC105375440*; h) rs143542634, *PATL1*.

a) HIPadjBMI All

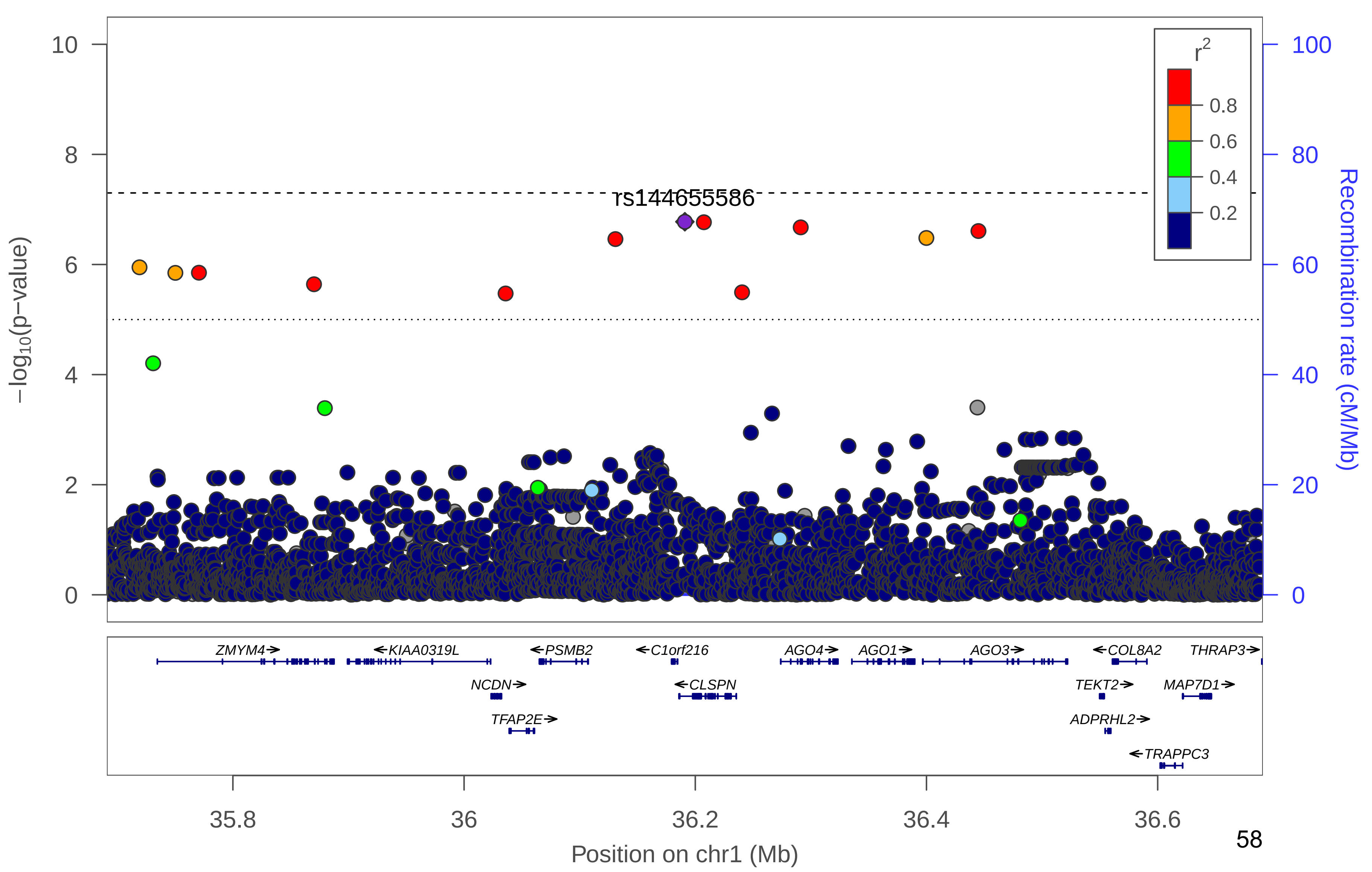

b) HIPadjBMI All

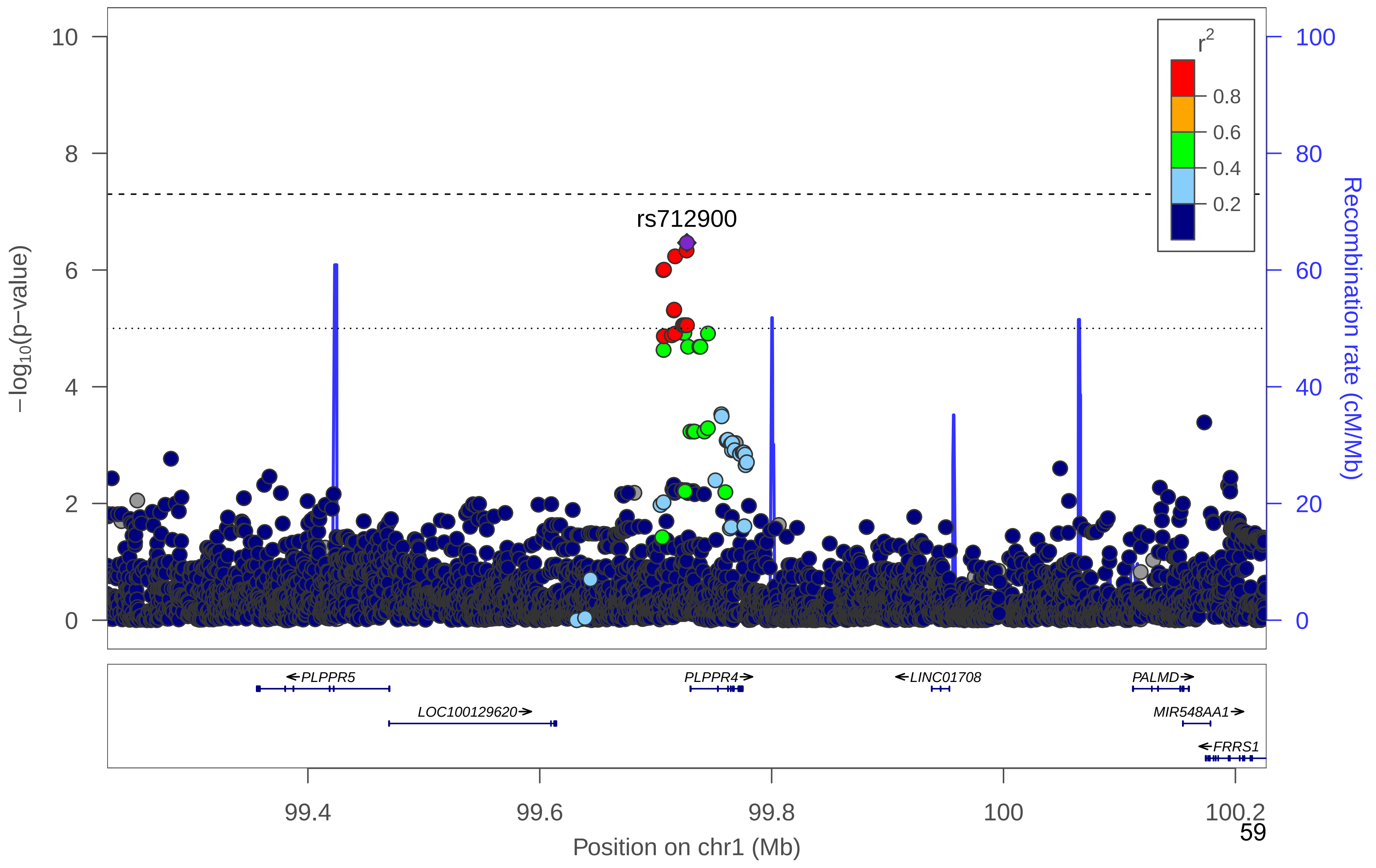

c) HIPadjBMI All

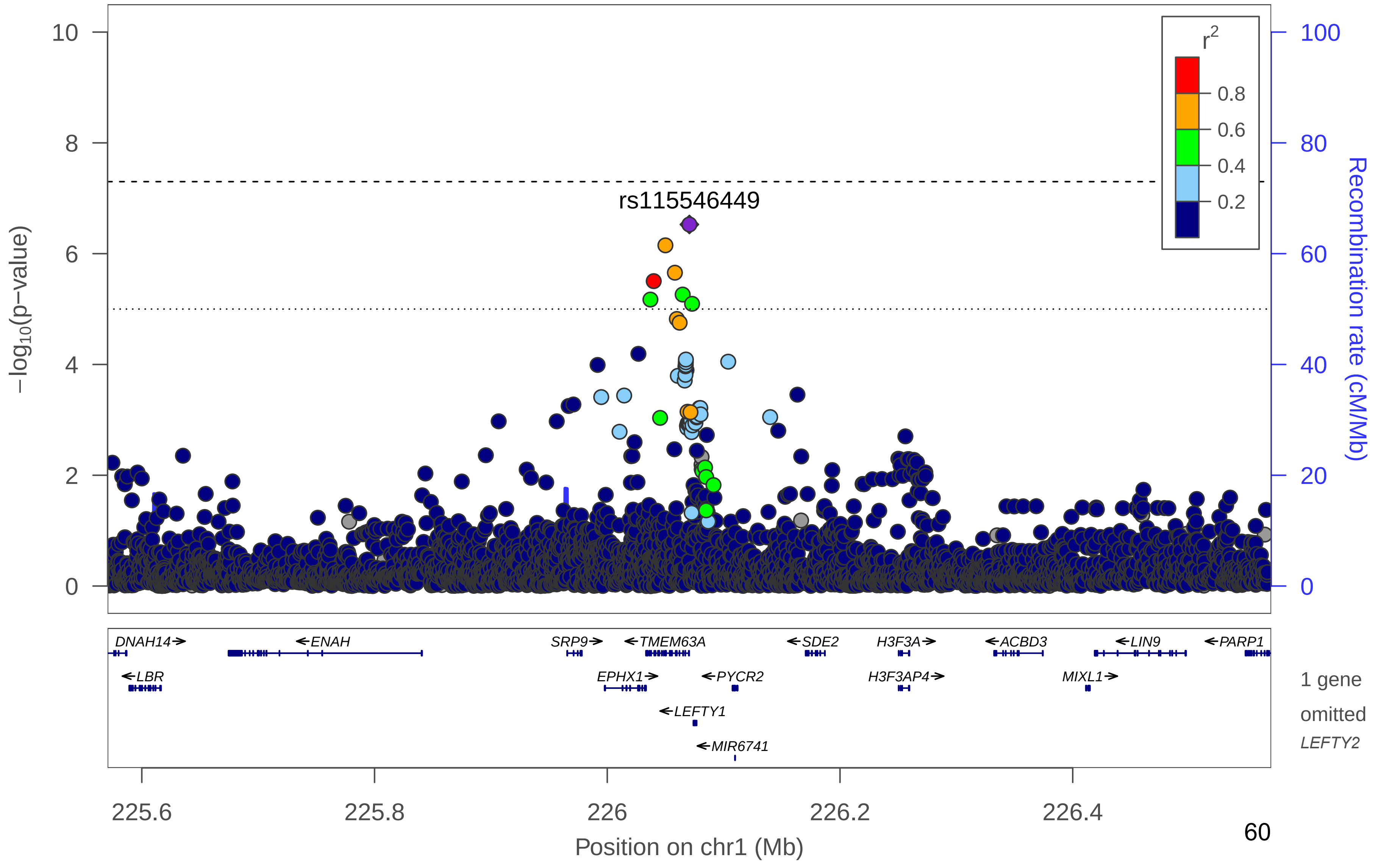

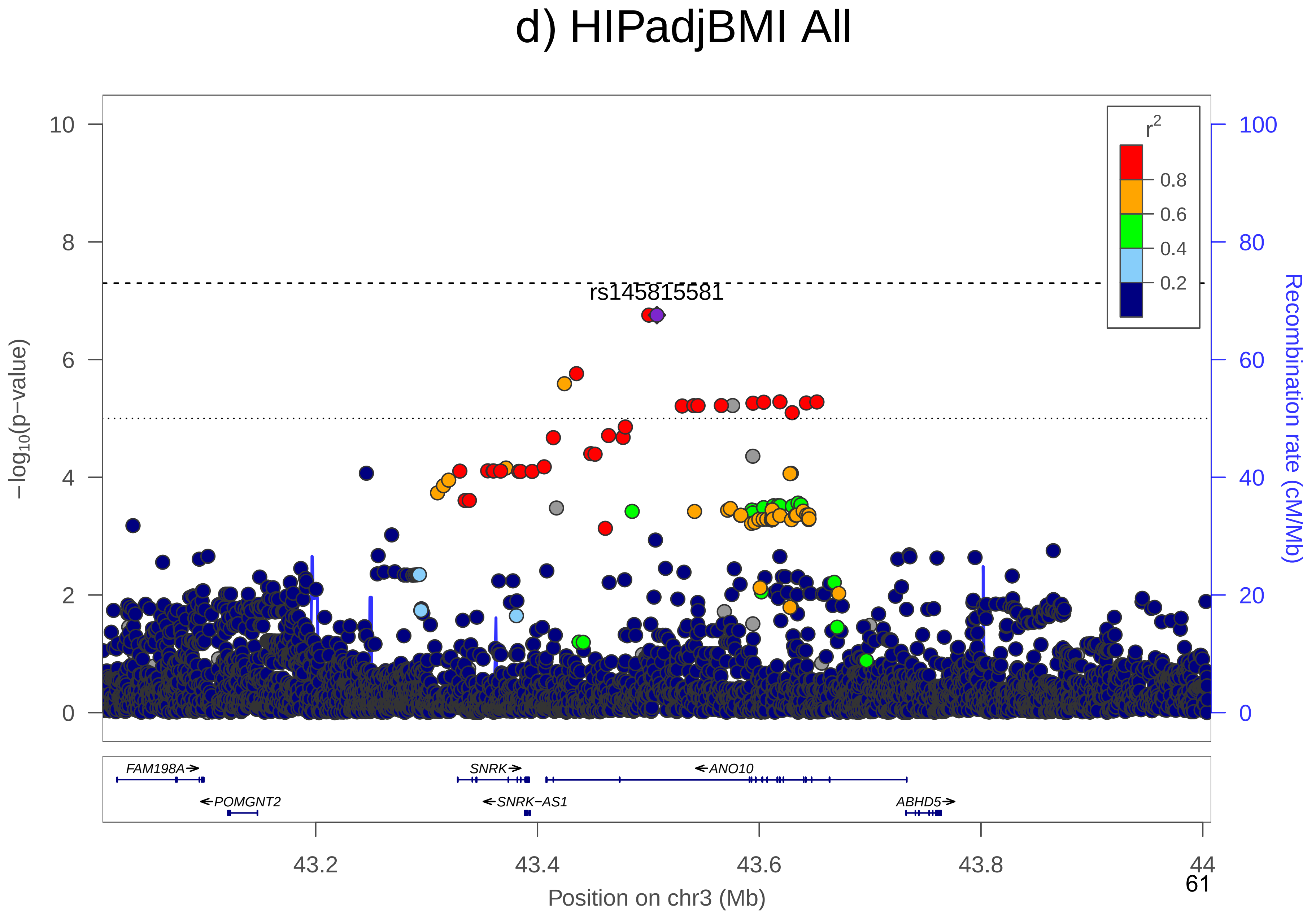

d) HIPadjBMI All
rs145815581
−log10(p−value)
Recombination rate (cM/Mb)
r2
0.8
0.6
0.4
0.2
FAM198A
POMGNT2
SNRK
SNRK−AS1
ANO10
ABHD5
Position on chr3 (Mb)
43.2
43.4
43.6
43.8
44

e) HIPadjBMI All

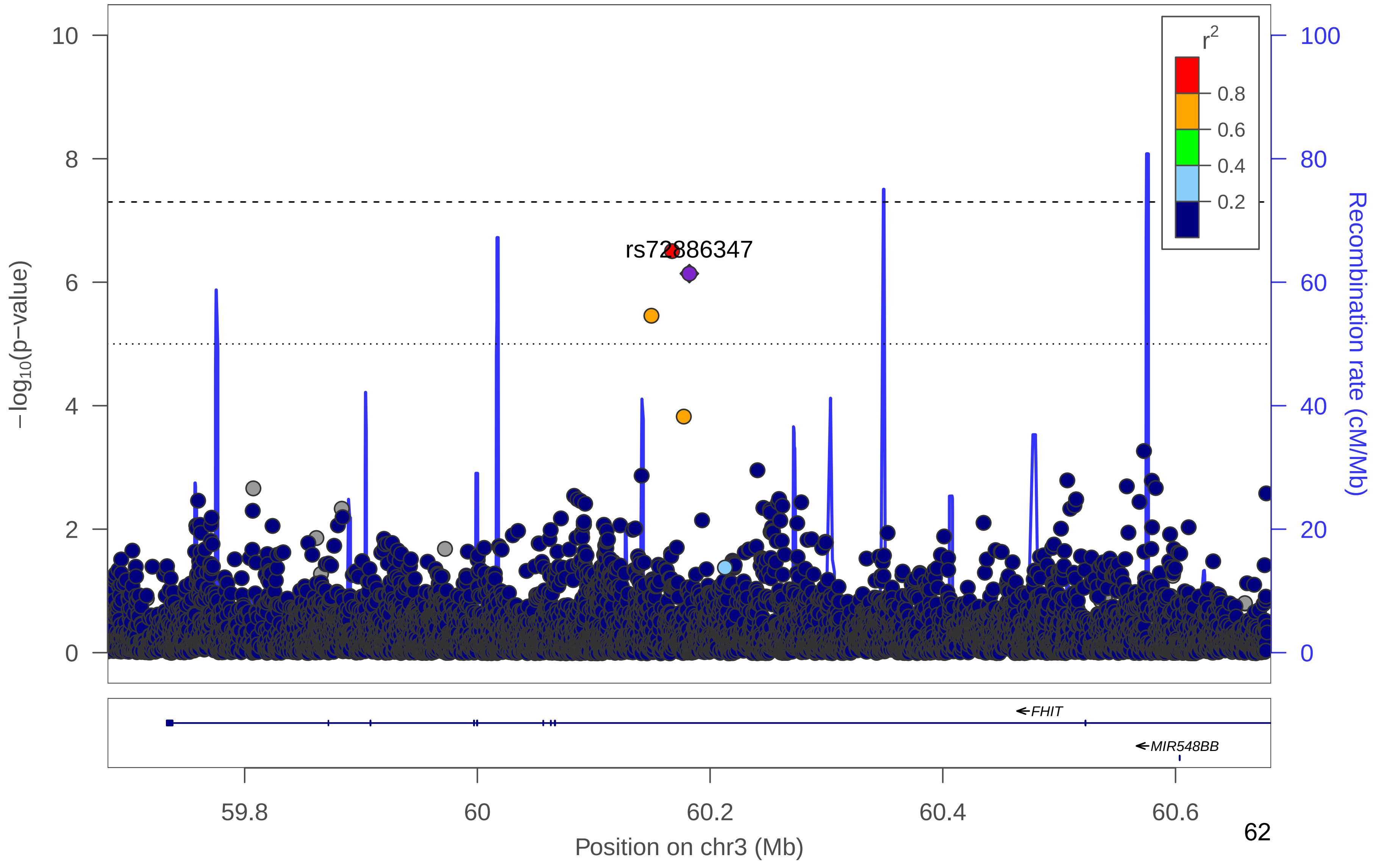

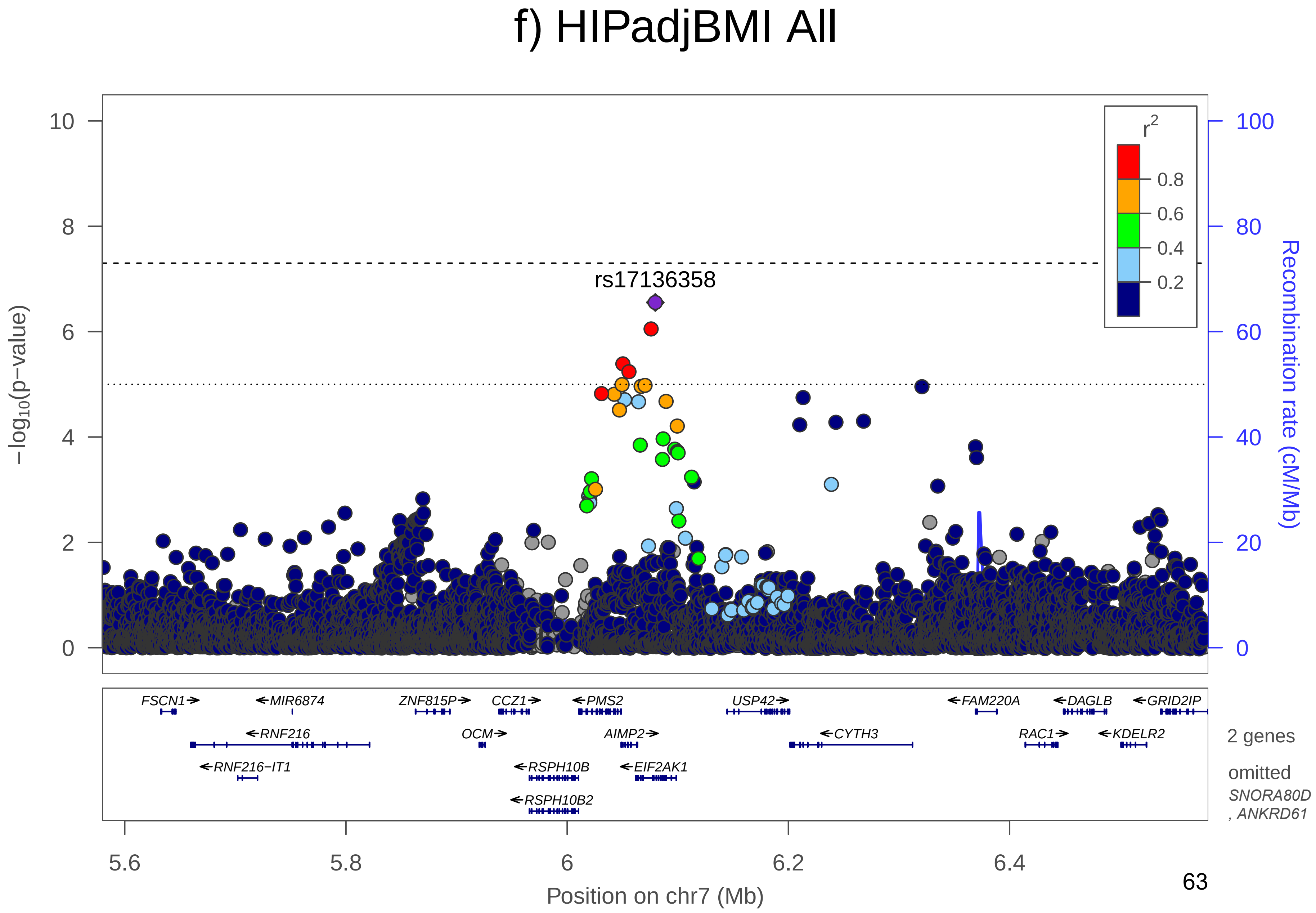

f) HIPadjBMI All
rs17136358
−log10(p−value)
Recombination rate (cM/Mb)
r2
0.8
0.6
0.4
0.2
FSCN1
MIR6874
ZNF815P
CCZ1
PMS2
USP42
FAM220A
DAGLB
GRID2IP
RNF216
OCM
AIMP2
CYTH3
RAC1
KDELR2
RNF216−IT1
RSPH10B
EIF2AK1
RSPH10B2
2 genes
omitted
SNORA80D
, ANKRD61
5.6
5.8
6
6.2
6.4
Position on chr7 (Mb)

g) HIPadjBMI All

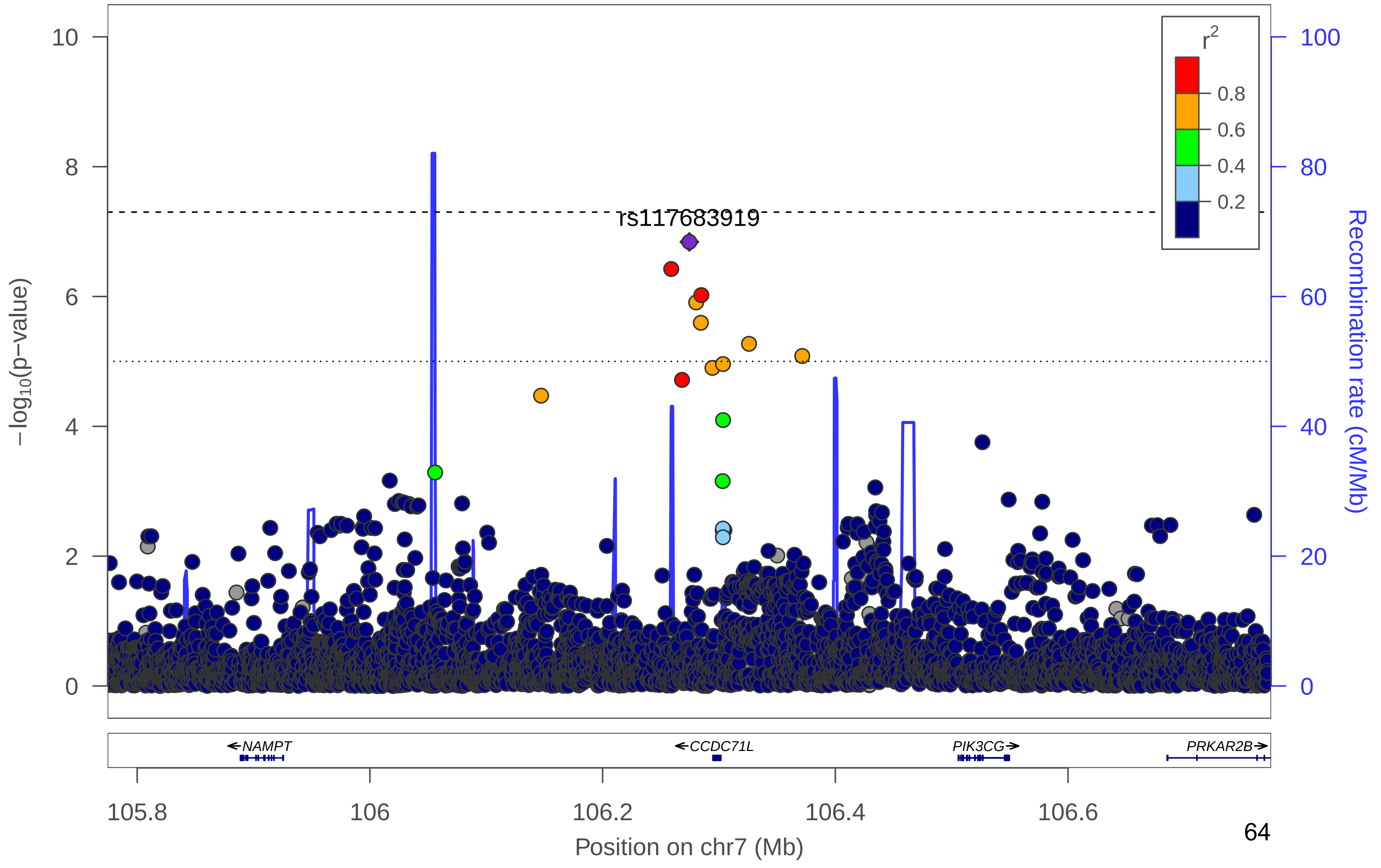

h) HIPadjBMI All

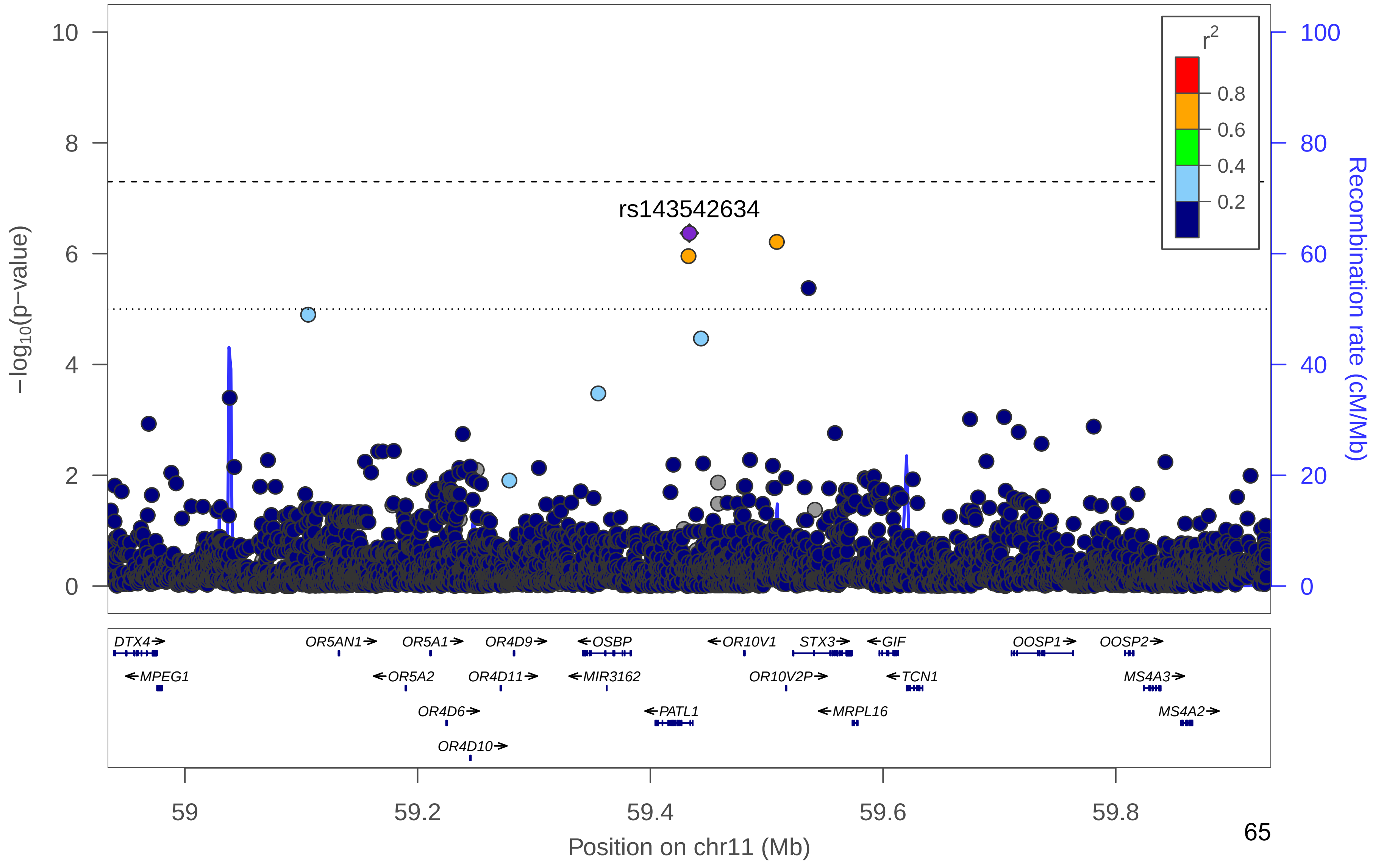

Supplementary Figure 17. Manhattan plot. Manhattan plot of the women-only analysis for HIPadjBMI. All suggestively significant (P<1x10^{-6}) variants are highlighted in orange if they are >500 Kb from any previously-reported HIPadjBMI associated variants. Previously reported loci (+/- 500 Kb) are highlighted in blue if any variant in the locus reached suggestive significance. All suggestively significant loci that meet our criteria for replication are annotated with the closest gene. †Replicated in African American meta-analysis. ‡ Replicated in Hispanic/Latino meta-analysis. ¥ Replicated in European American meta-analysis.

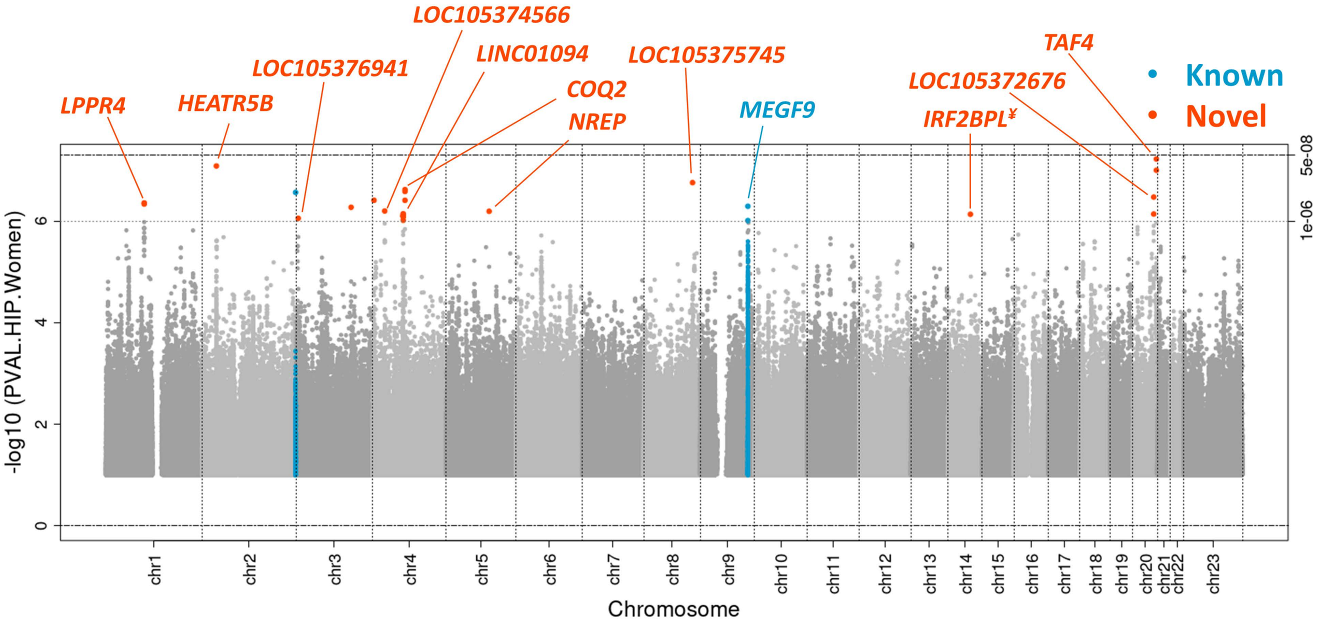

Supplementary Figure 18. Locus Zoom Plots. Regional association plots for suggestively significant loci in the HCHS/SOL HIPadjBMI women-only analysis. The plots appear in chromosome:position order. Dot color reflects R^2 calculated from the 1000 Genomes AMR reference dataset. Point symbols represent variant functional classifications: a) rs72978809, *LPPR4*; b) rs12478843, *HEATR5B*; c) rs115331260, *LOC105376941*; d) rs7662640, *LOC105374566*; e) rs6814739, *LINC01094*; f) rs11099588, *COQ2*; g) rs6860625, *NREP*; h) rs77186623, *LOC105375745*; i) rs10818474, *MEGF9*; j) rs28692724, *IRF2BPL*; k) rs6092086, *LOC105372676*; l) rs9631175, *TAF4*.

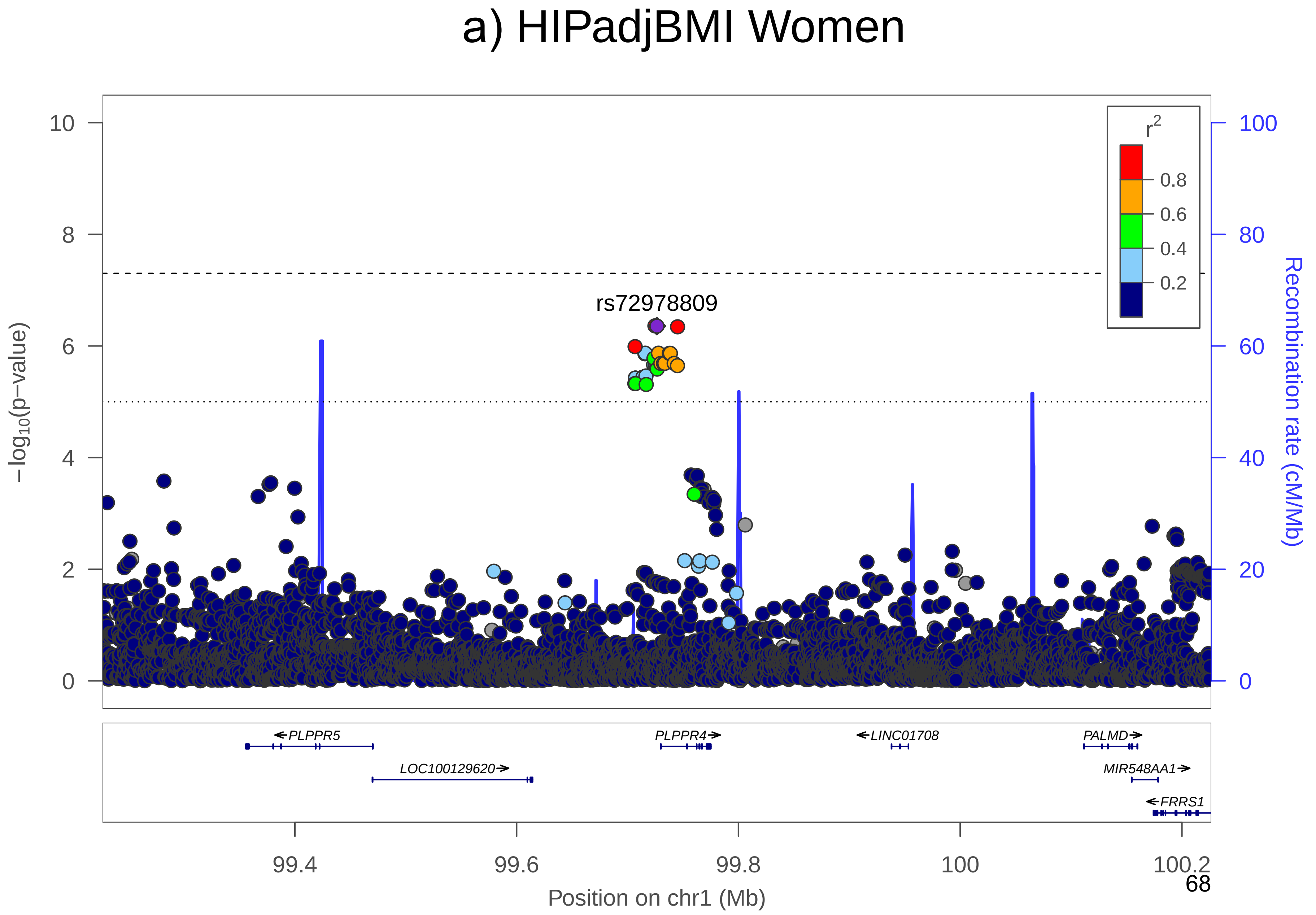

a) HIPadjBMI Women
rs72978809
−log10(p−value)
Recombination rate (cM/Mb)
r2
0.8
0.6
0.4
0.2
PLPPR5
LOC100129620
PLPPR4
LINC01708
PALMD
MIR548AA1
FRRS1
99.4
99.6
99.8
100
100.2
Position on chr1 (Mb)

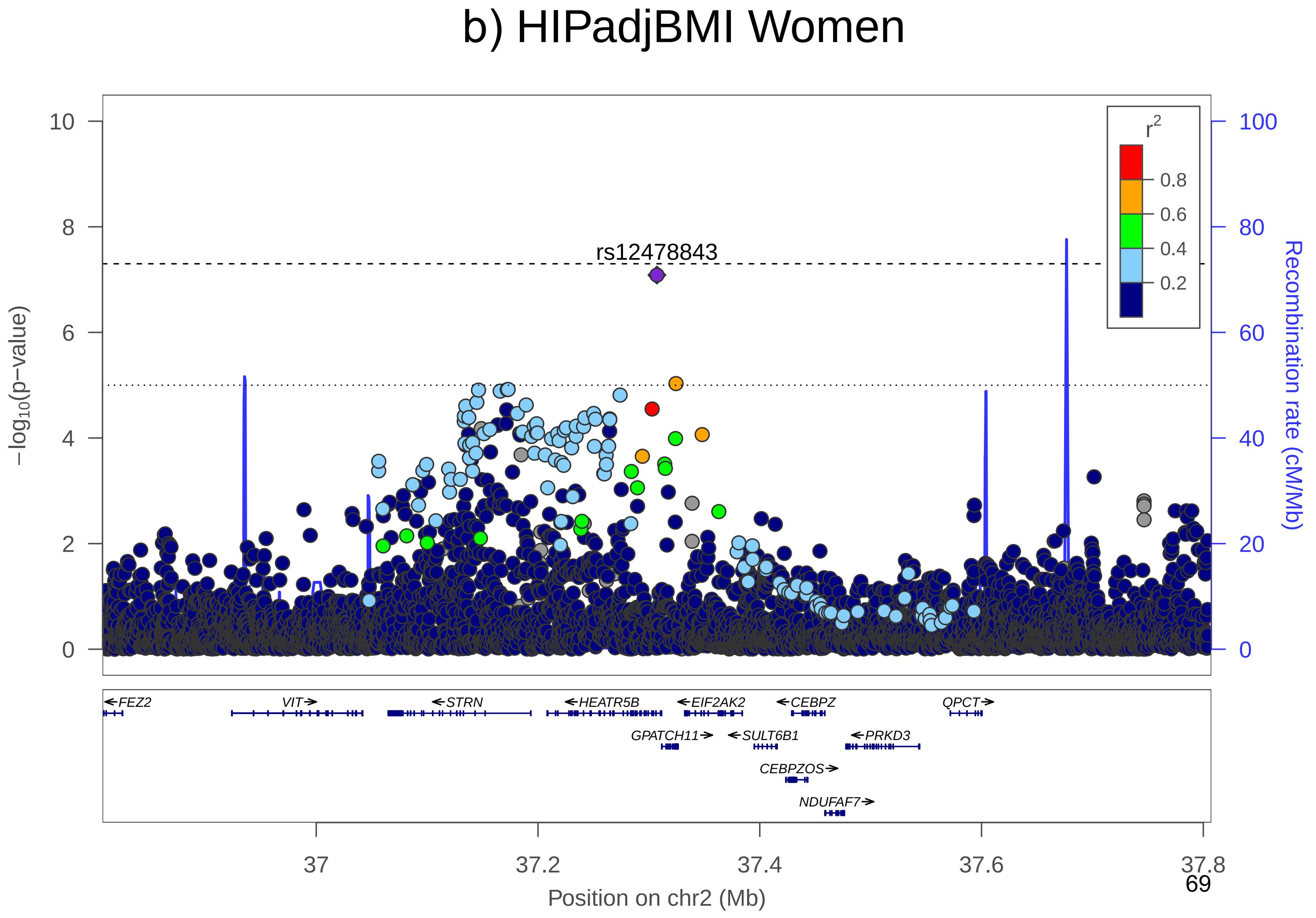
b) HIPadjBMI Women
rs12478843
−log10(p−value)
Recombination rate (cM/Mb)
r2
0.8
0.6
0.4
0.2
10
8
6
4
2
0
100
80
60
40
20
0
FEZ2
VIT
STRN
HEATR5B
EIF2AK2
CEBPZ
QPCT
GPATCH11
SULT6B1
PRKD3
CEBPZOS
NDUFAF7
37
37.2
37.4
37.6
37.8
Position on chr2 (Mb)

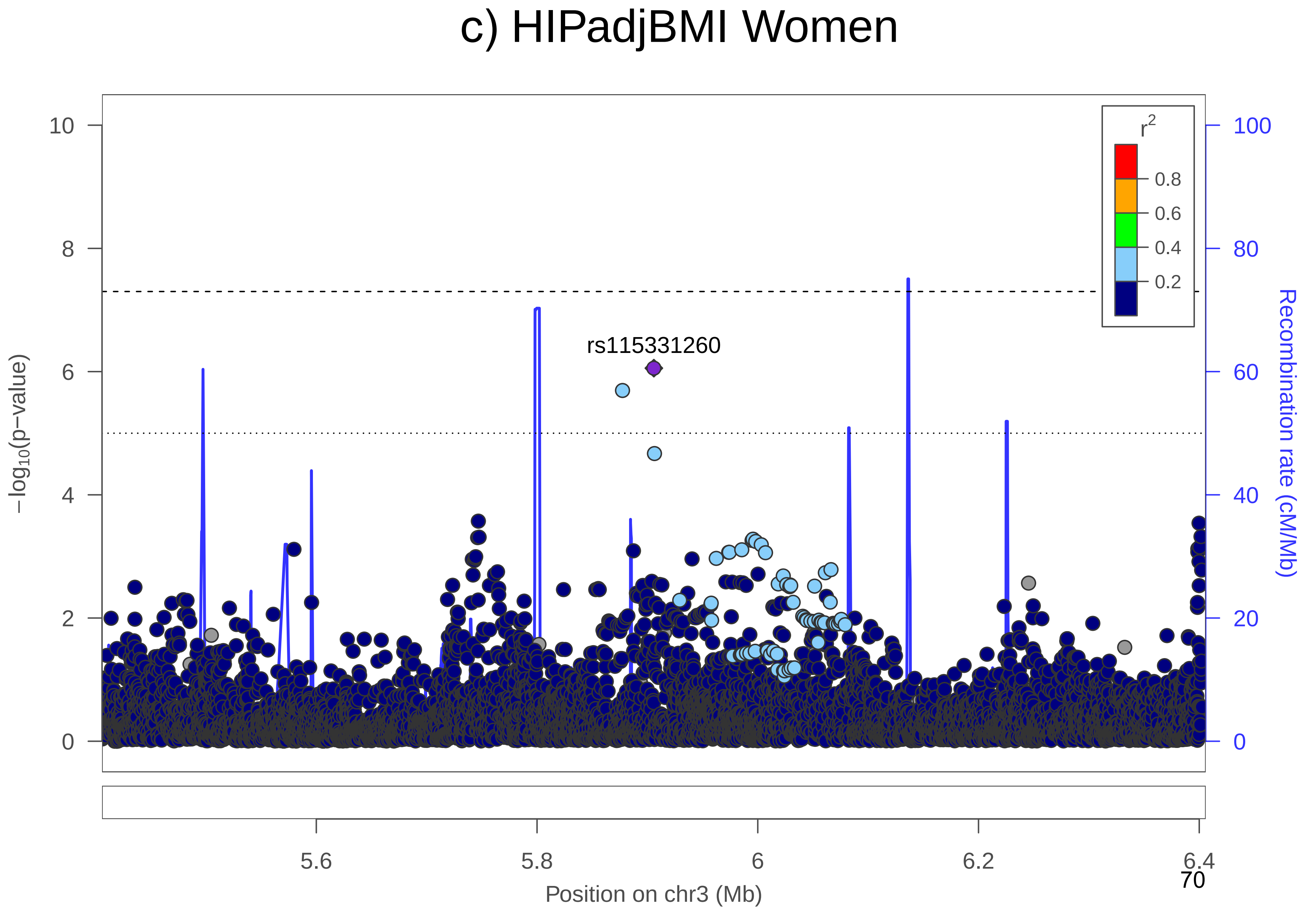
c) HIPadjBMI Women
rs115331260
r²
0.8
0.6
0.4
0.2
−log10(p−value)
10
8
6
4
2
0
100
80
60
40
20
0
Recombination rate (cM/Mb)
5.6
5.8
6
6.2
6.4
Position on chr3 (Mb)

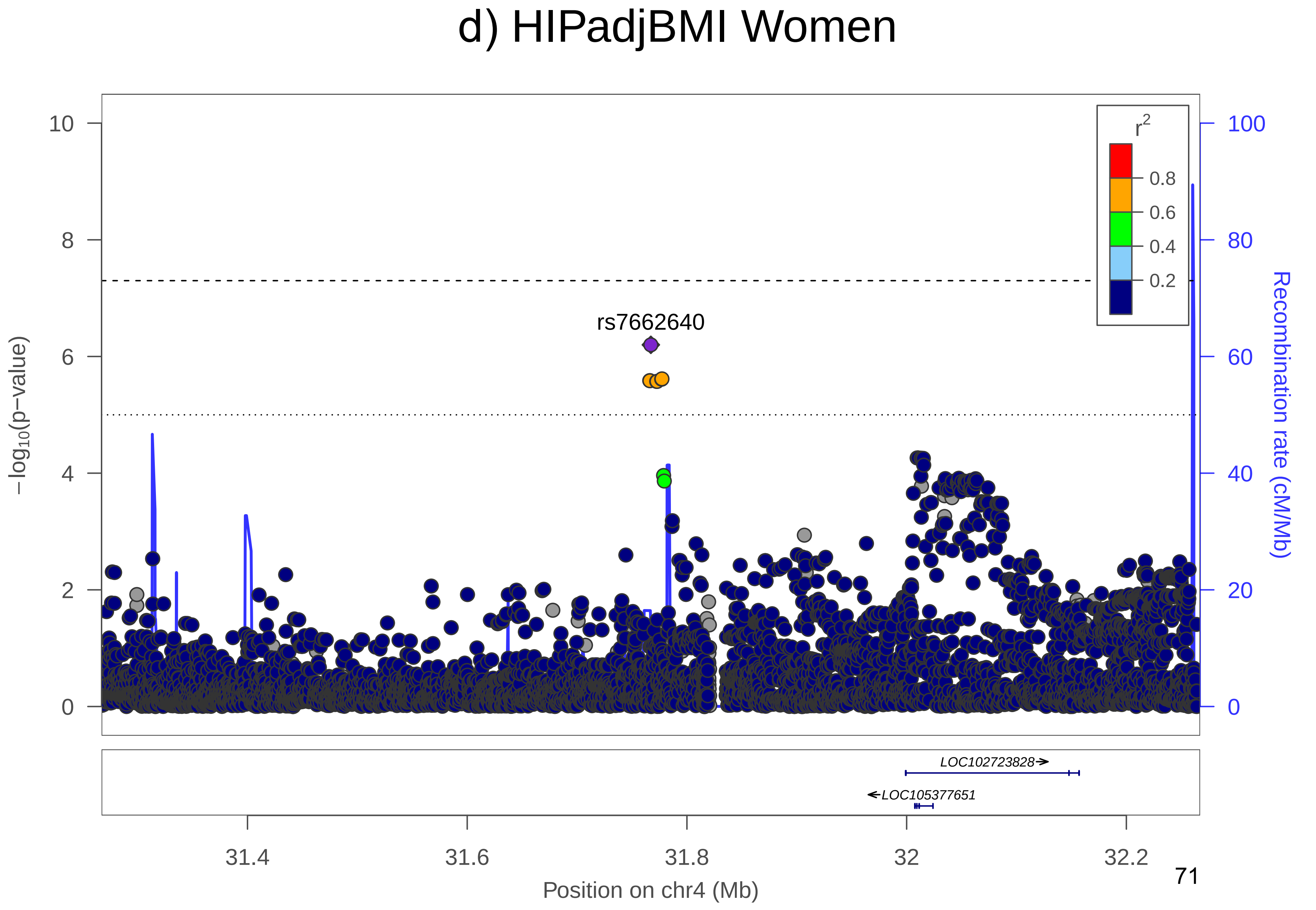
d) HIPadjBMI Women
−log10(p−value)
Recombination rate (cM/Mb)
Position on chr4 (Mb)
rs7662640
r2
0.8
0.6
0.4
0.2
LOC102723828
LOC105377651
31.4
31.6
31.8
32
32.2
0
2
4
6
8
10
0
20
40
60
80
100

e) HIPadjBMI Women

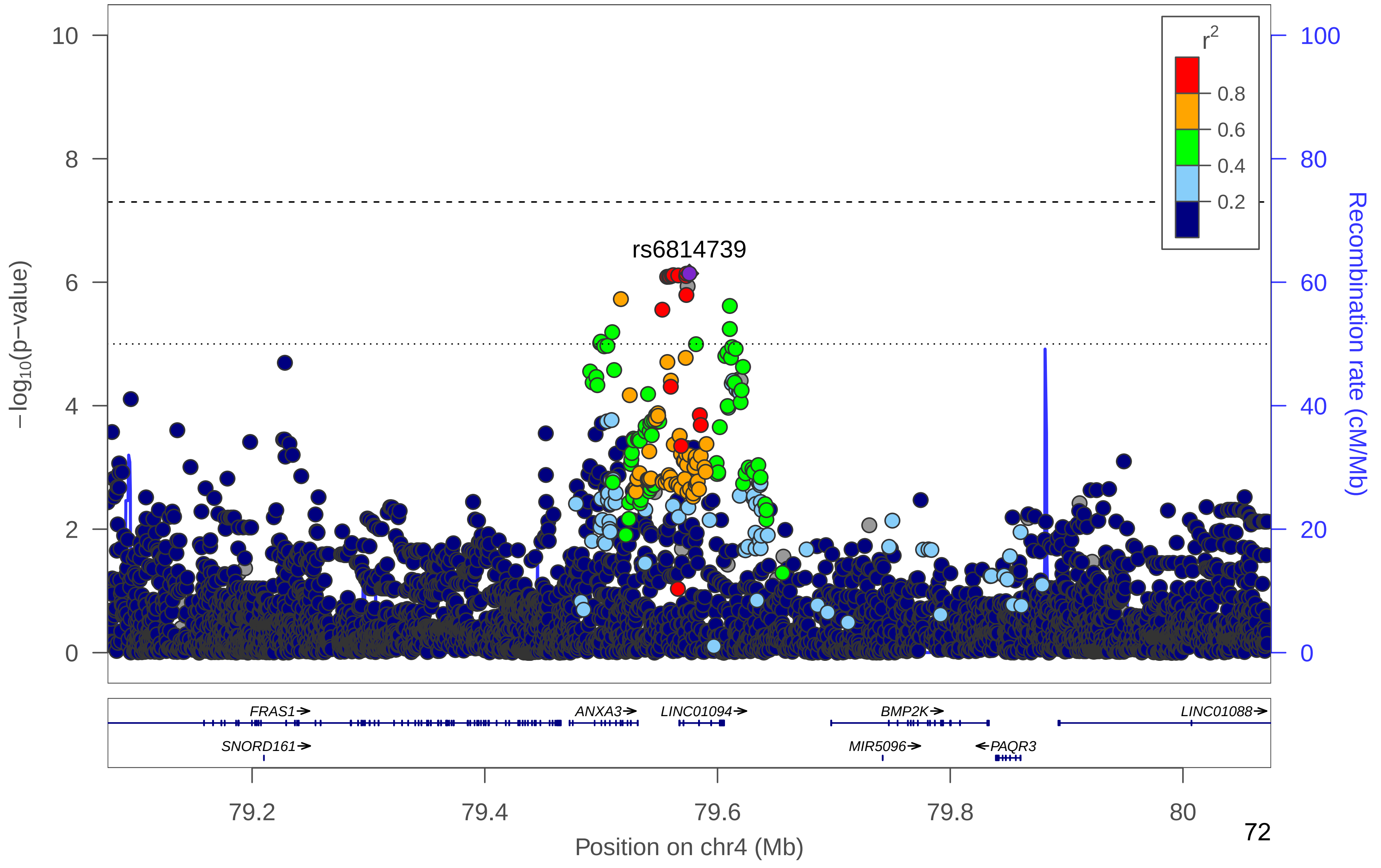

f) HIPadjBMI Women

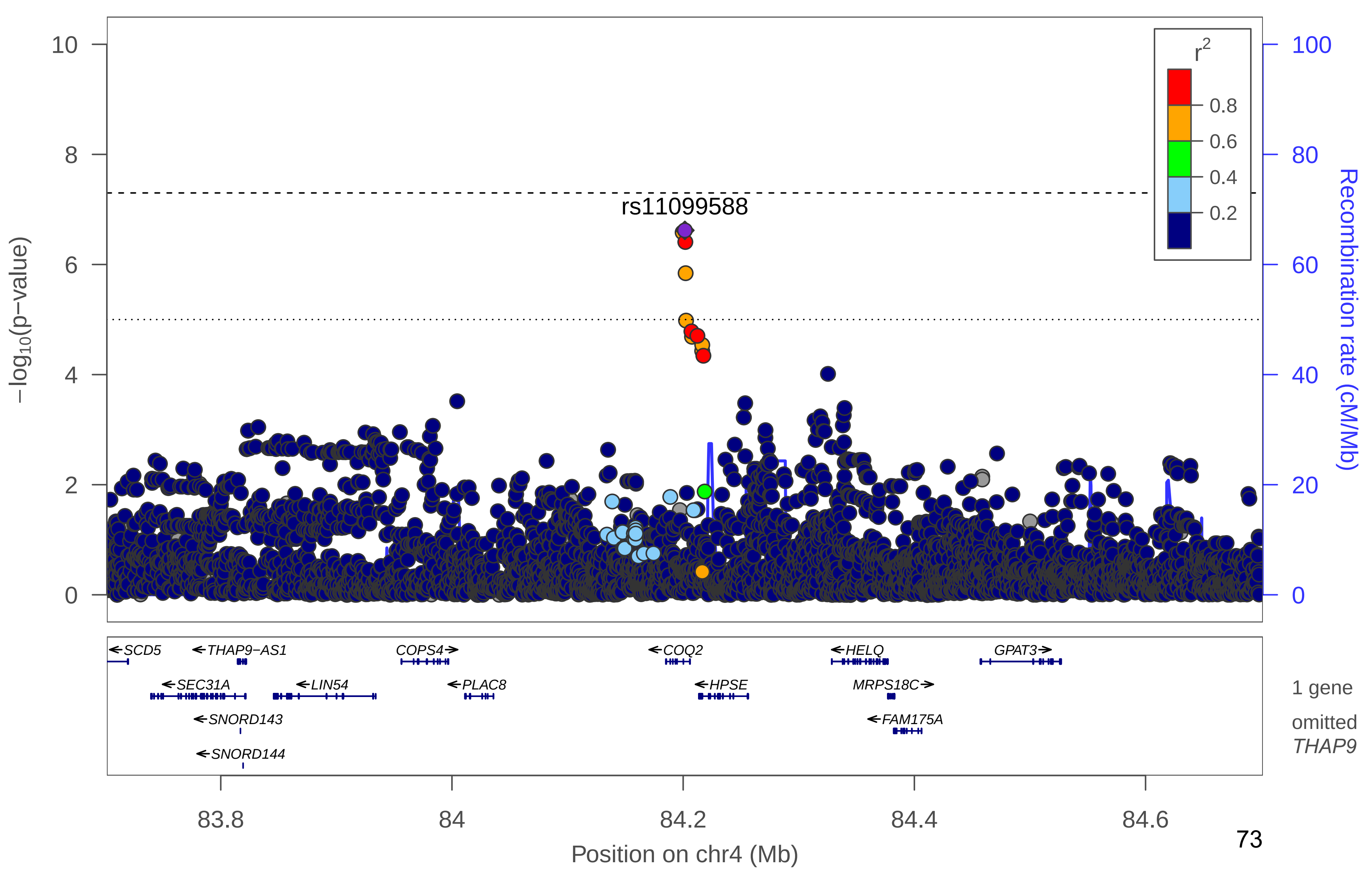

g) HIPadjBMI Women

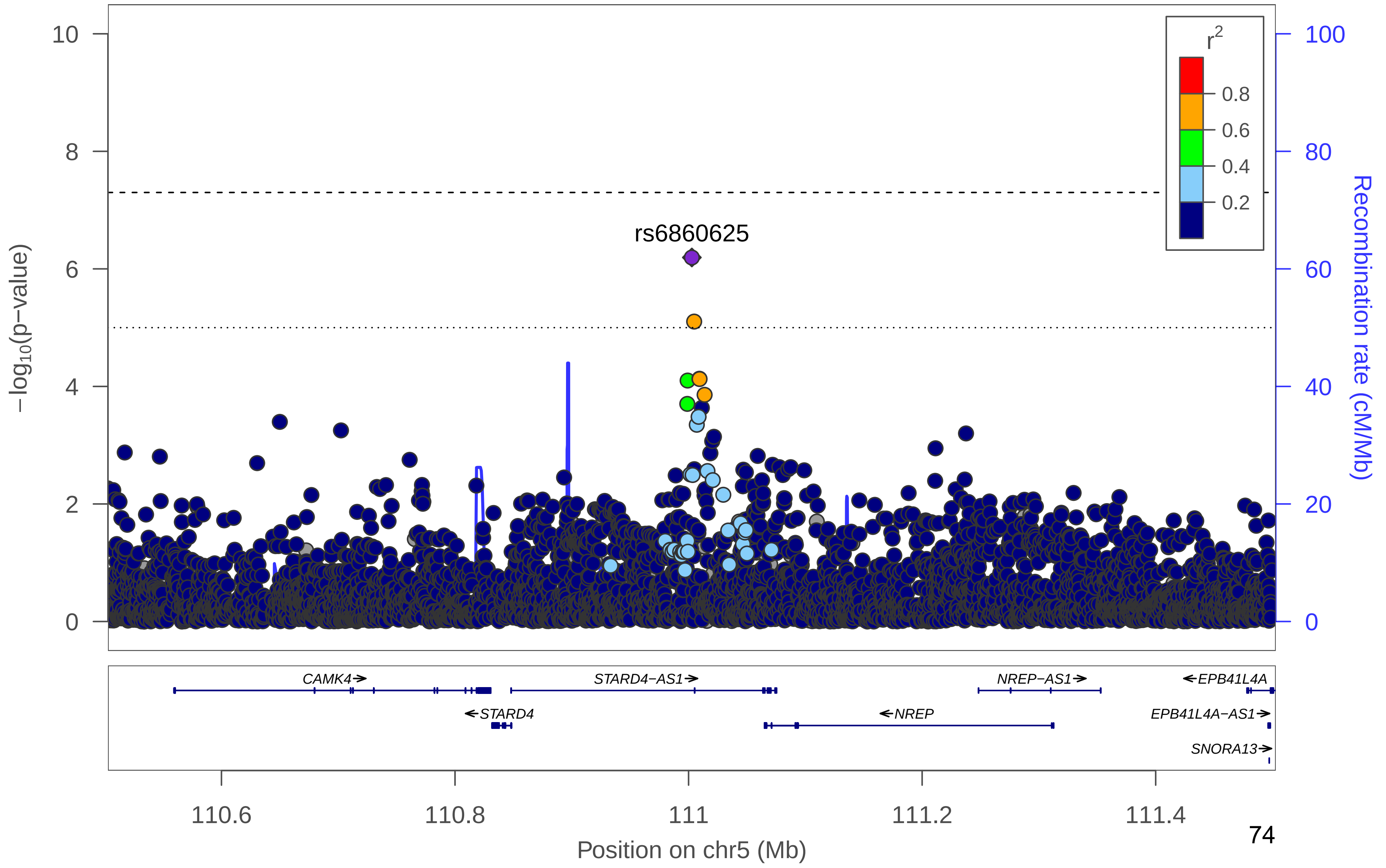

h) HIPadjBMI Women

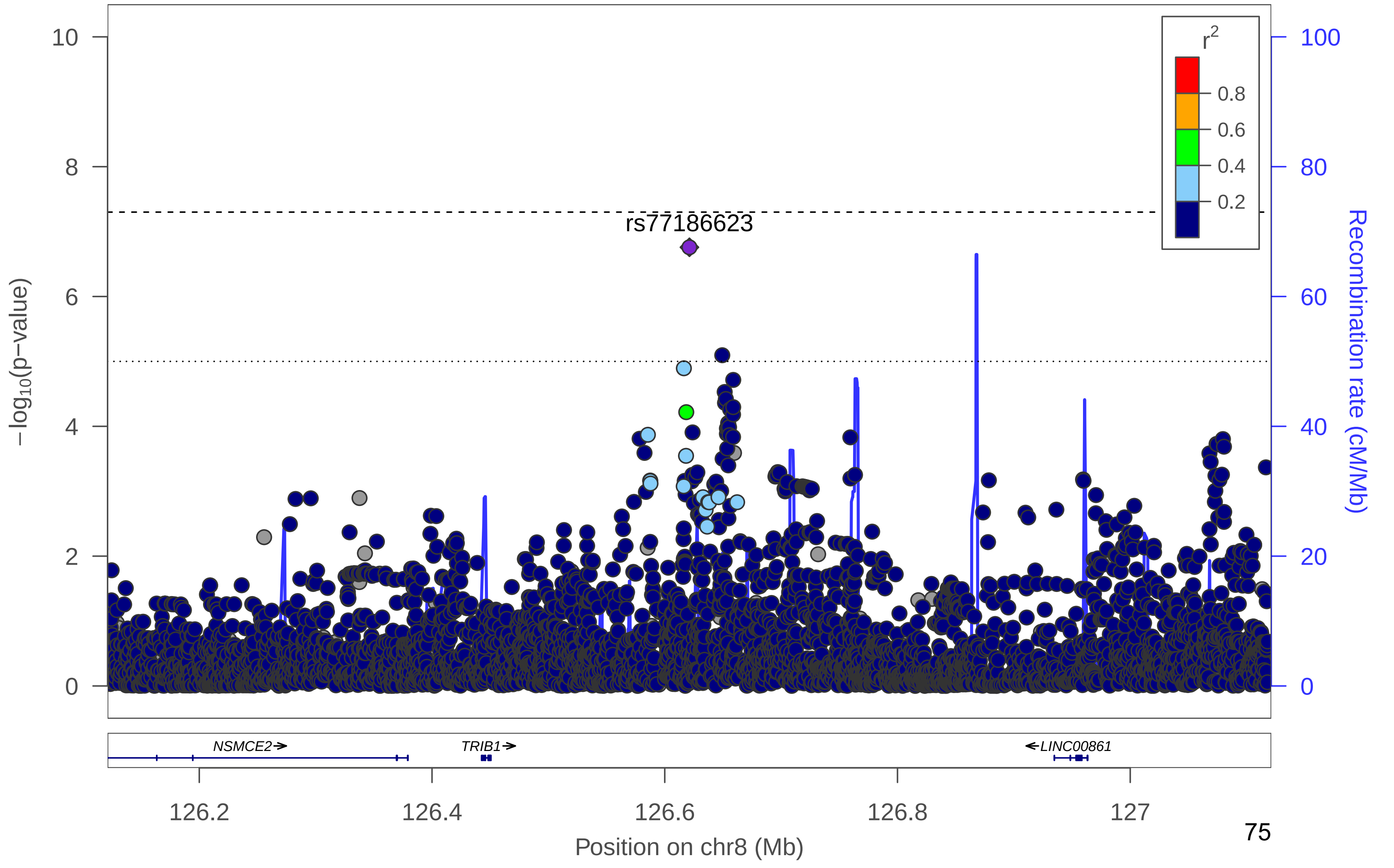

i) HIPadjBMI Women

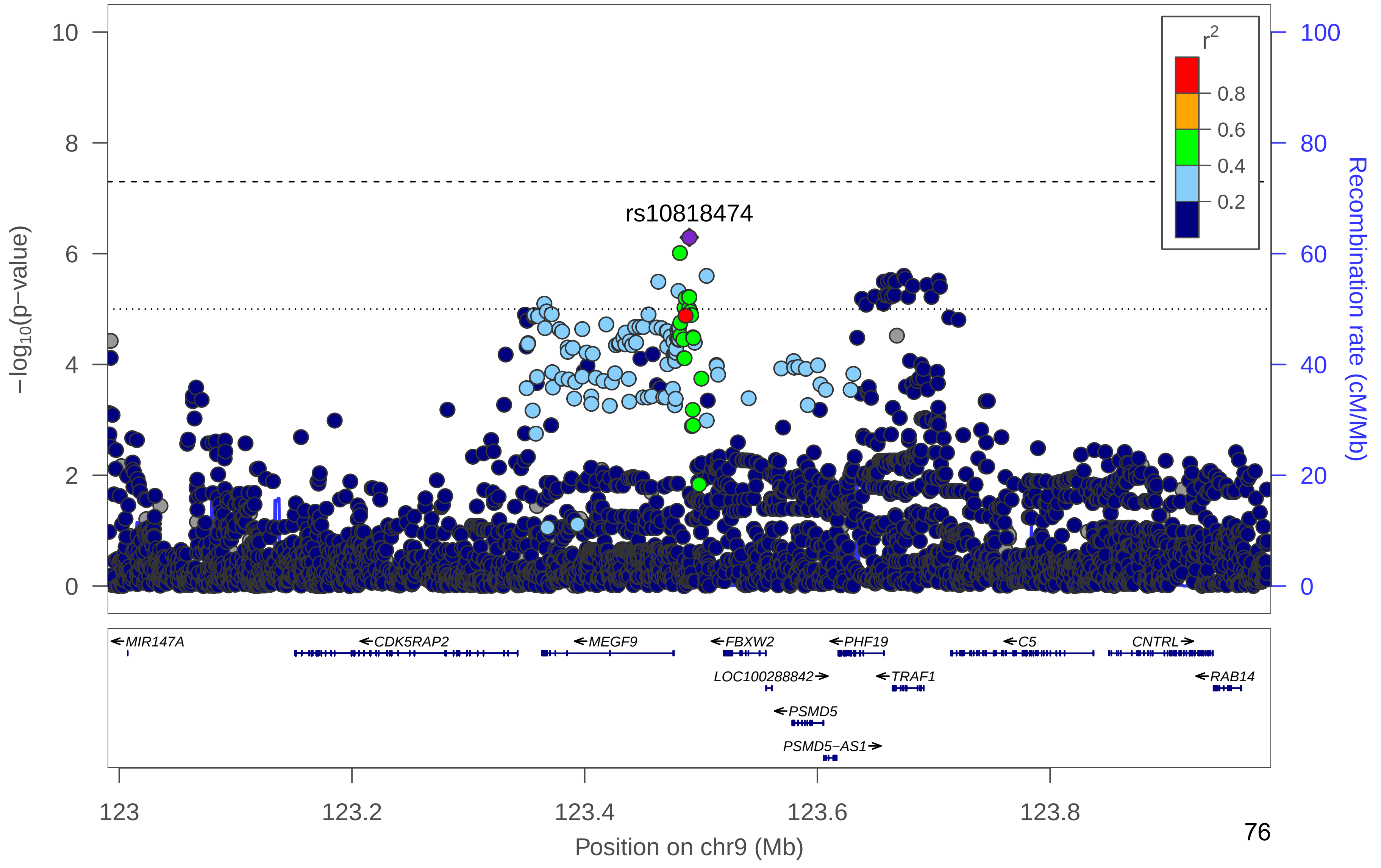

j) HIPadjBMI Women

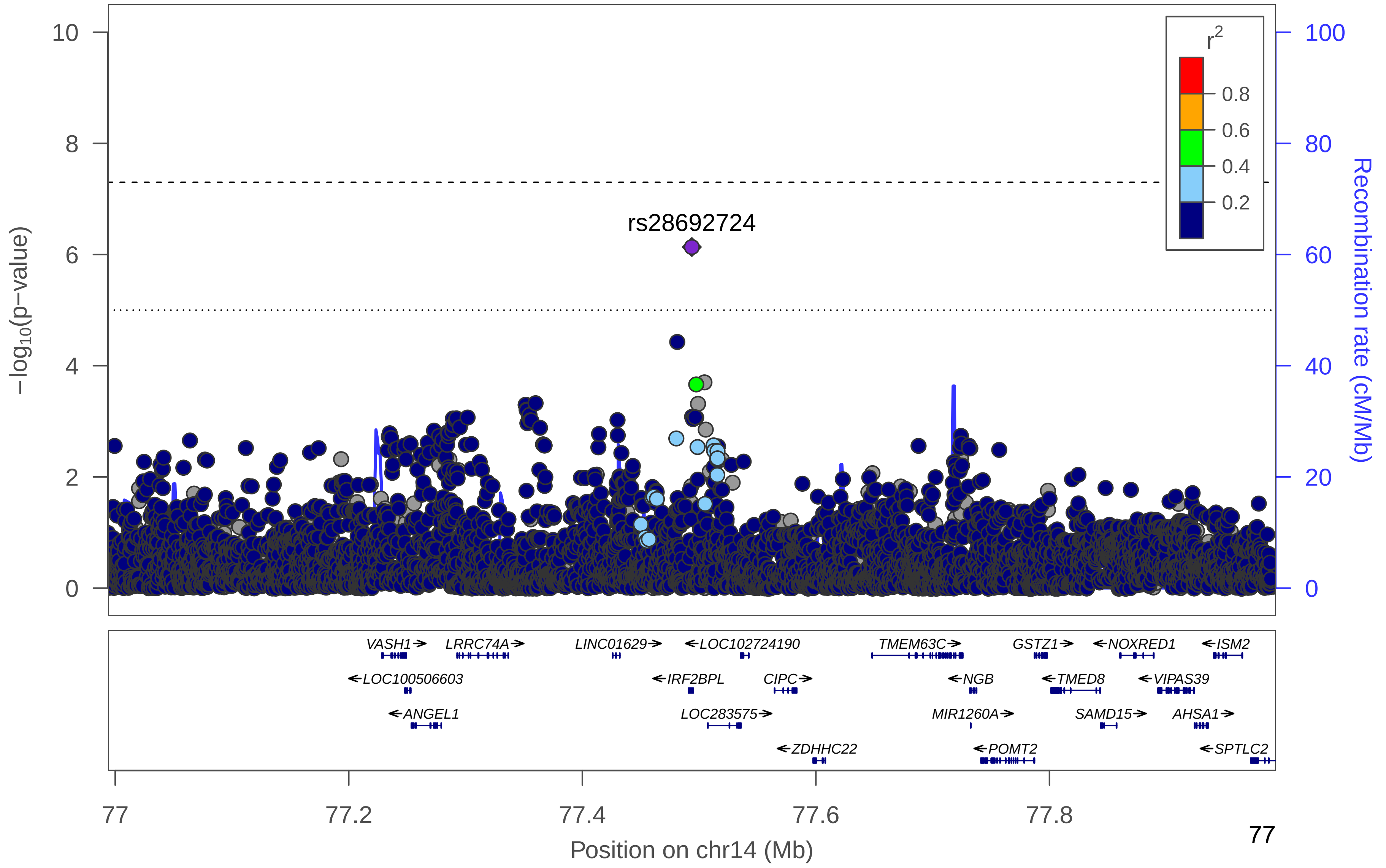

k) HIPadjBMI Women

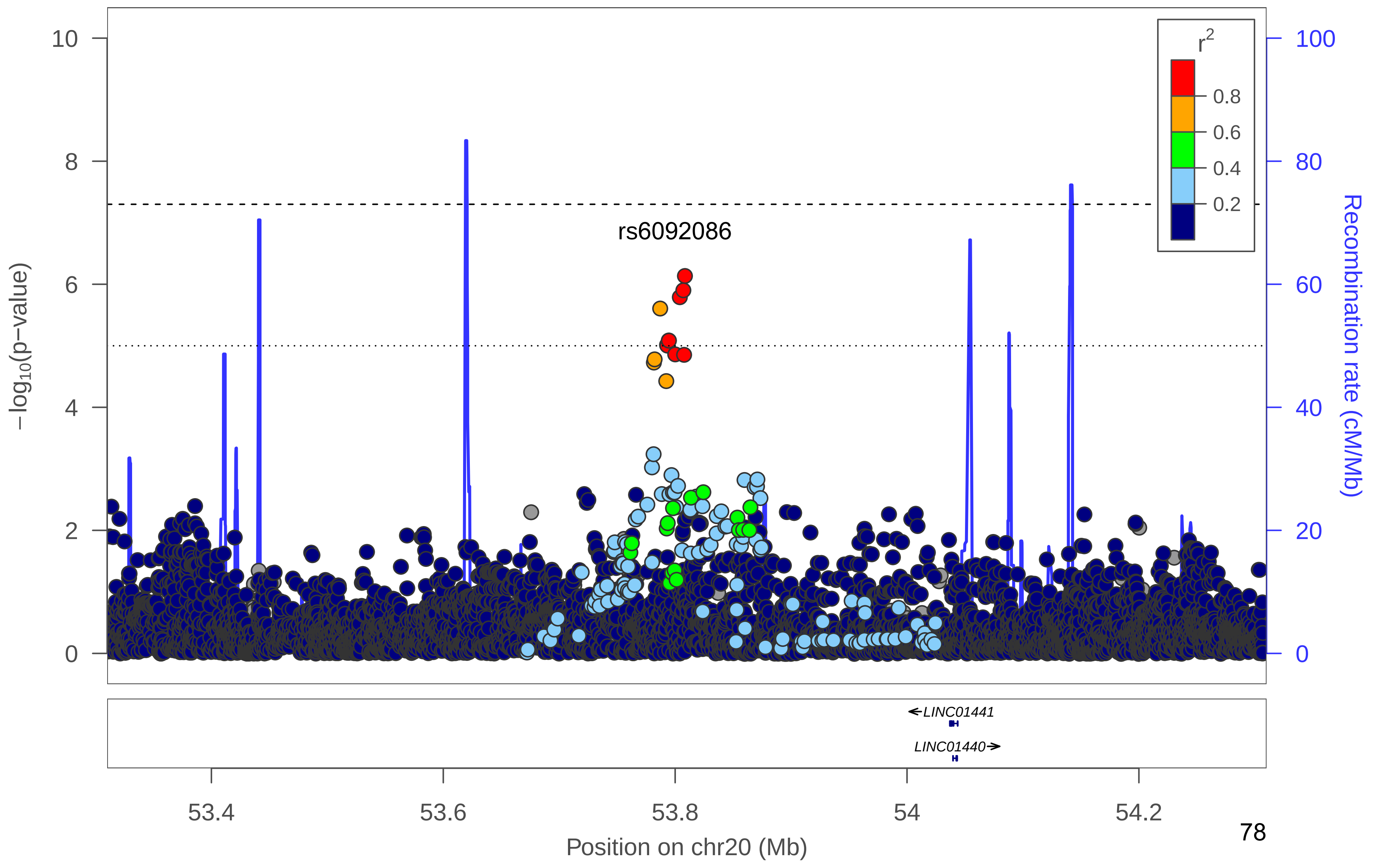

l) HIPadjBMI Women

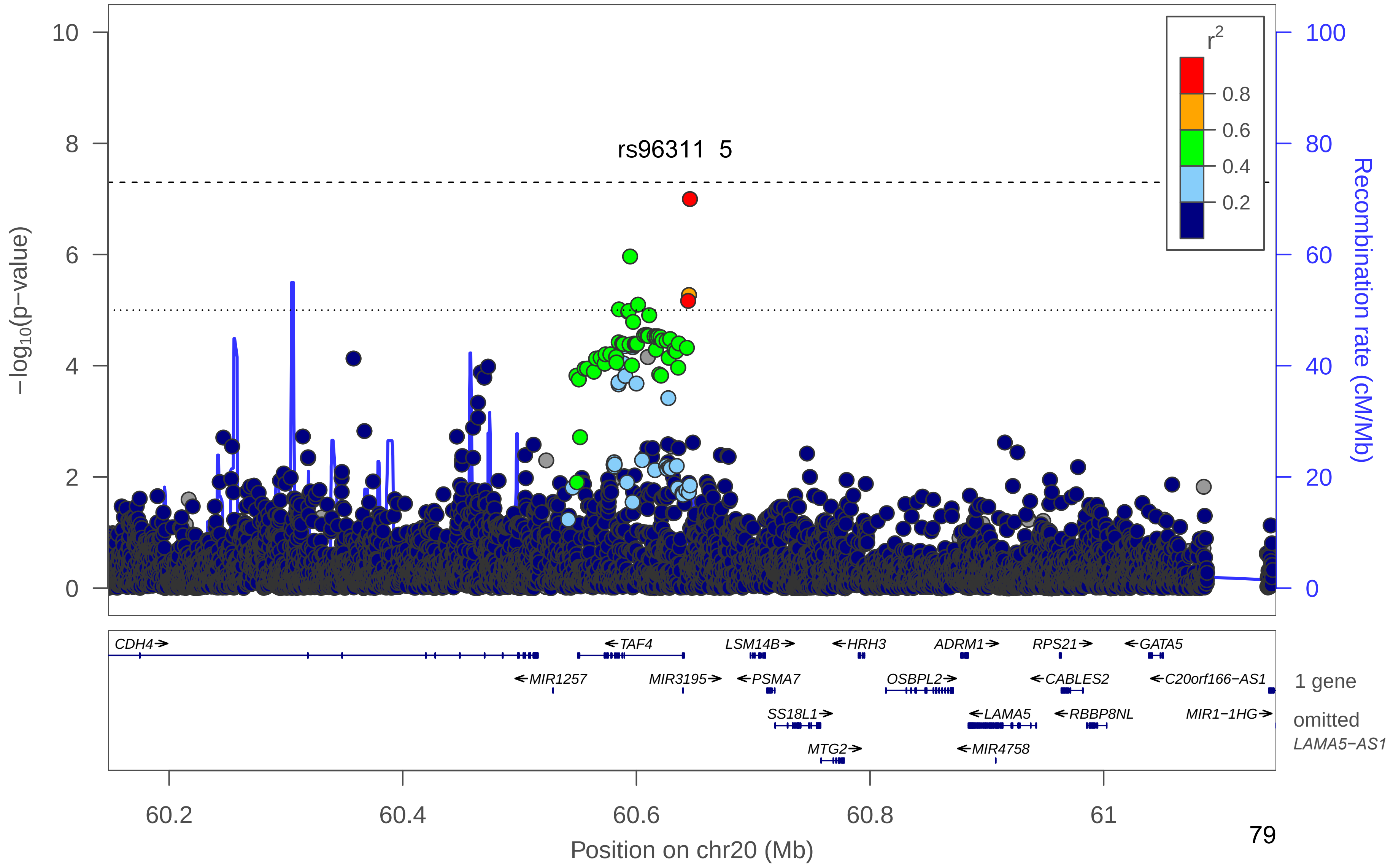

Supplementary Figure 19. Manhattan plot. Manhattan plot of the men-only analysis for HIPadjBMI. All suggestively significant (P<1x10^{-6}) variants are highlighted in orange if they are >500 Kb from any previously-reported HIPadjBMI associated variants. Previously reported loci (+/- 500 Kb) are highlighted in blue if any variant in the locus reached suggestive significance. All suggestively significant loci that meet our criteria for replication are annotated with the closest gene. †Replicated in African American meta-analysis. ‡ Replicated in Hispanic/Latino meta-analysis. ¥ Replicated in European American meta-analysis.

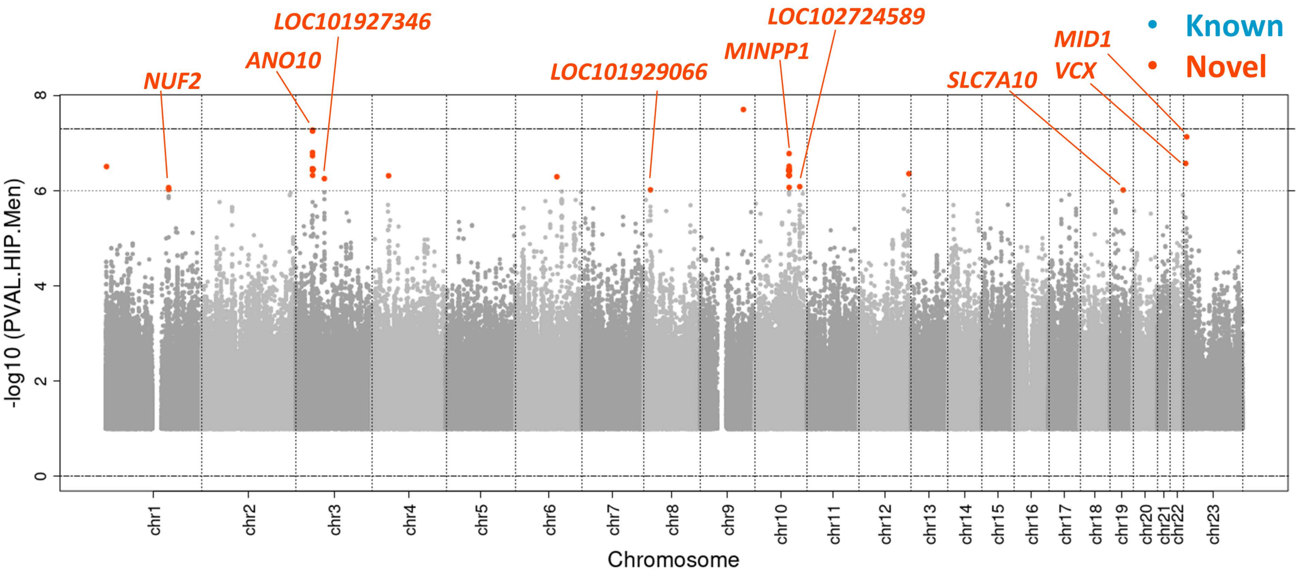

Supplementary Figure 20. Locus Zoom Plots. Regional association plots for suggestively significant loci in the HCHS/SOL HIPadjBMI men-only analysis. The plots appear in chromosome:position order. Dot color reflects R^2 calculated from the 1000 Genomes AMR reference dataset. Point symbols represent variant functional classifications: a) rs114865909, *NUF2*; b) rs149681500, *ANO10*; c) rs3915213, *LOC101927346*; d) rs12677587, *LOC101929066*; e) rs56405004, *MINPP1*; f) rs968849, *LOC102724589*; g) rs76469489, *SLC7A10*; h) rs7063750, *VCX*; i) rs112519383, *MID1*.

a) HIPadjBMI Men

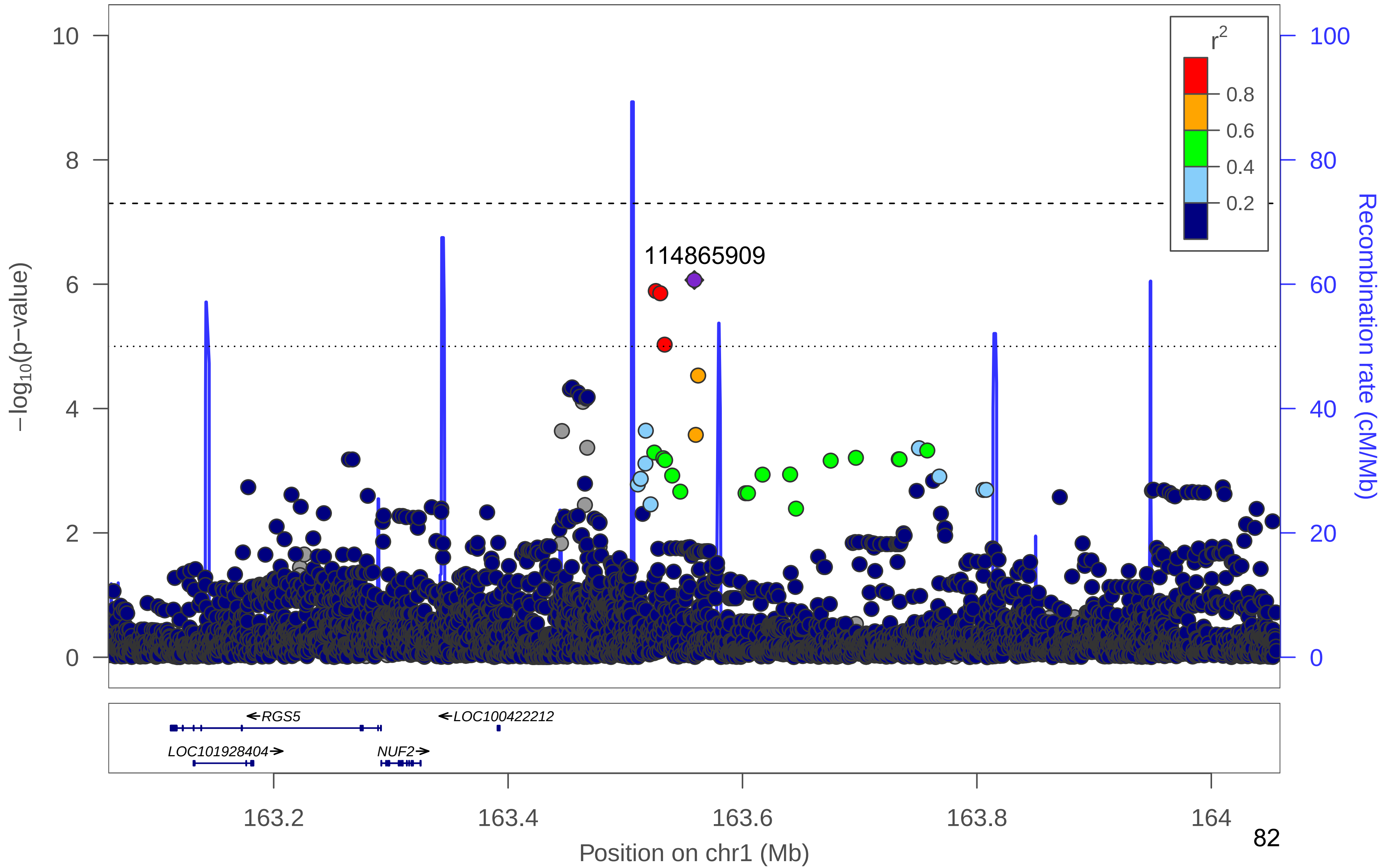

b) HIPadjBMI Men

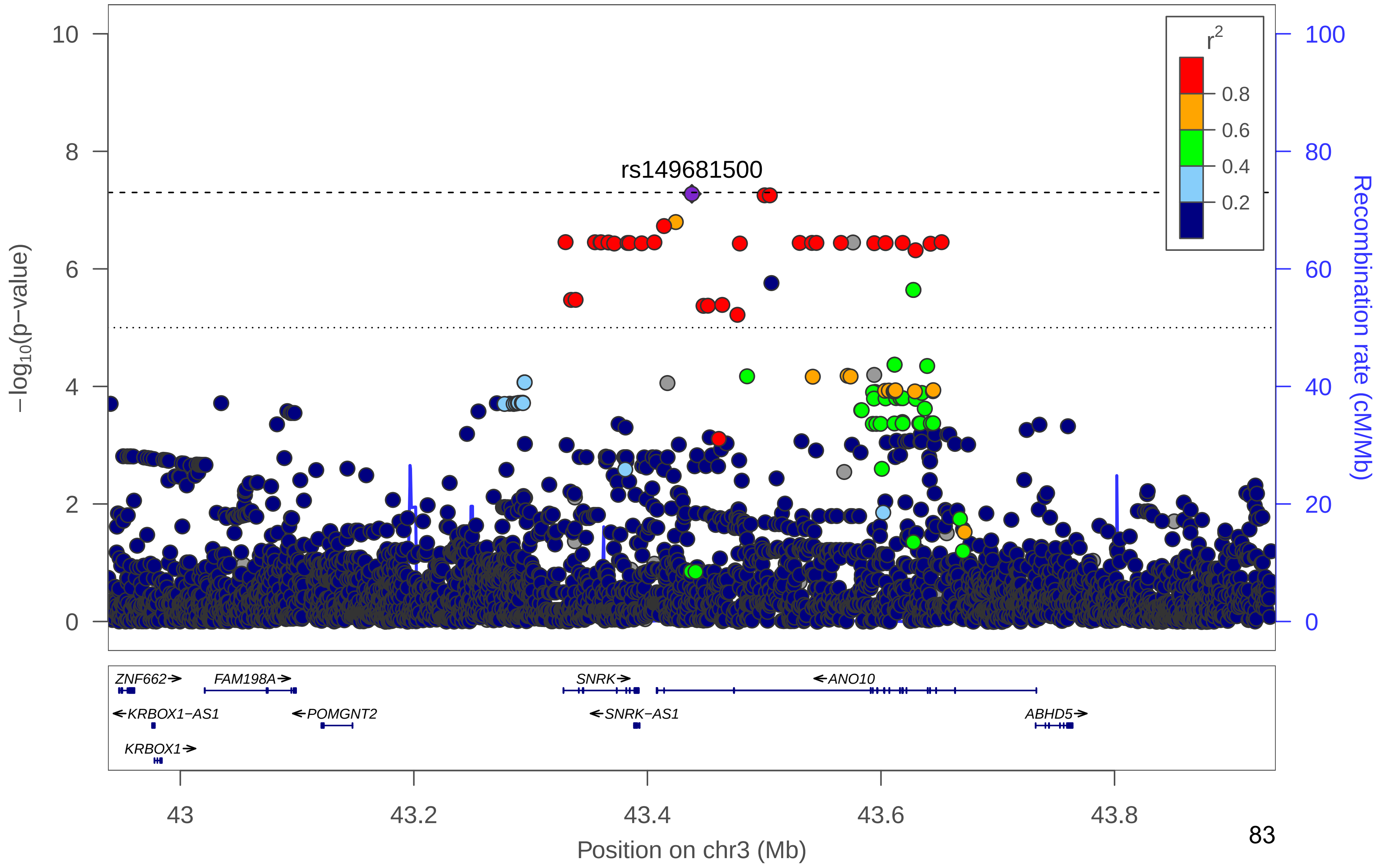

c) HIPadjBMI Men

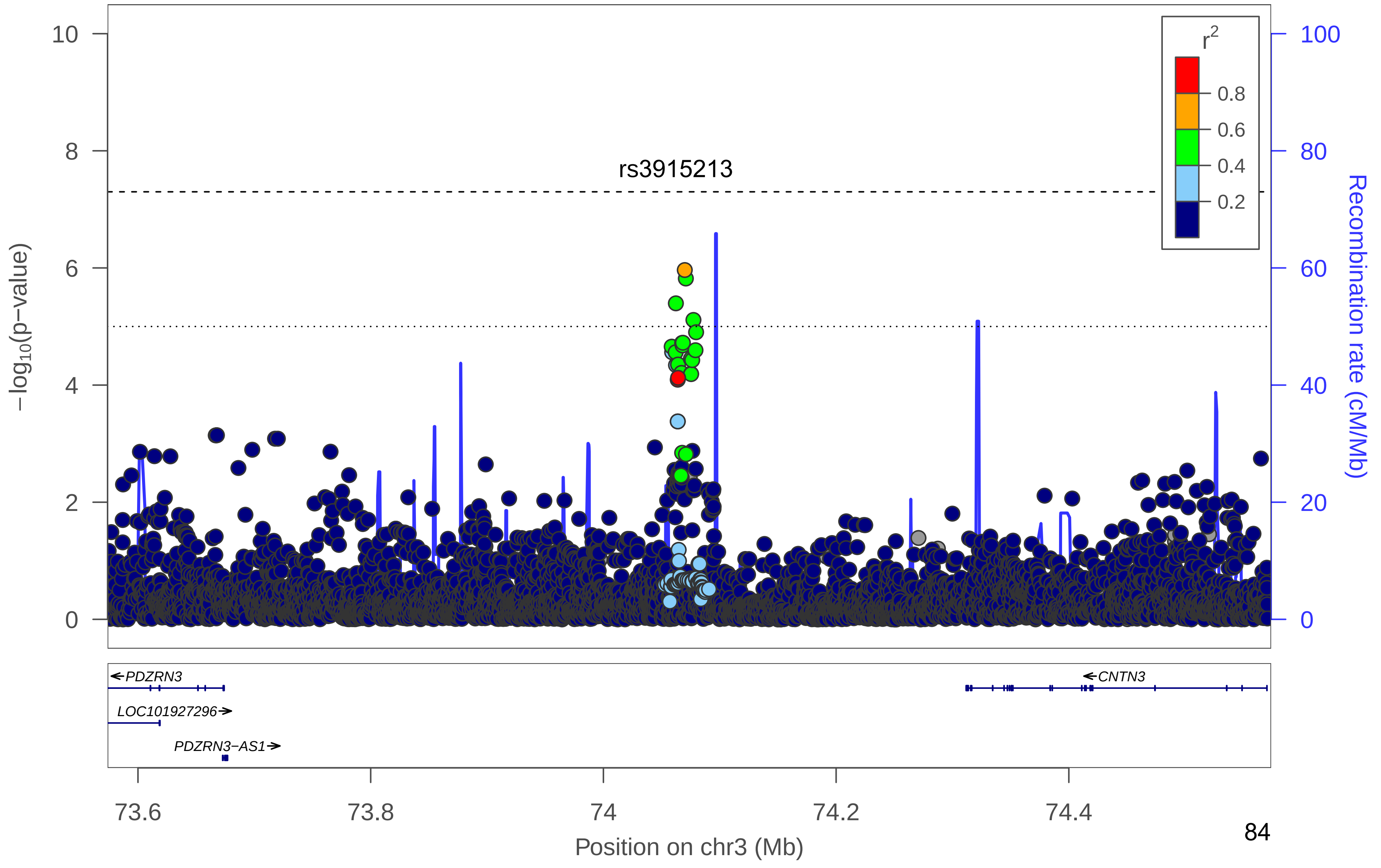

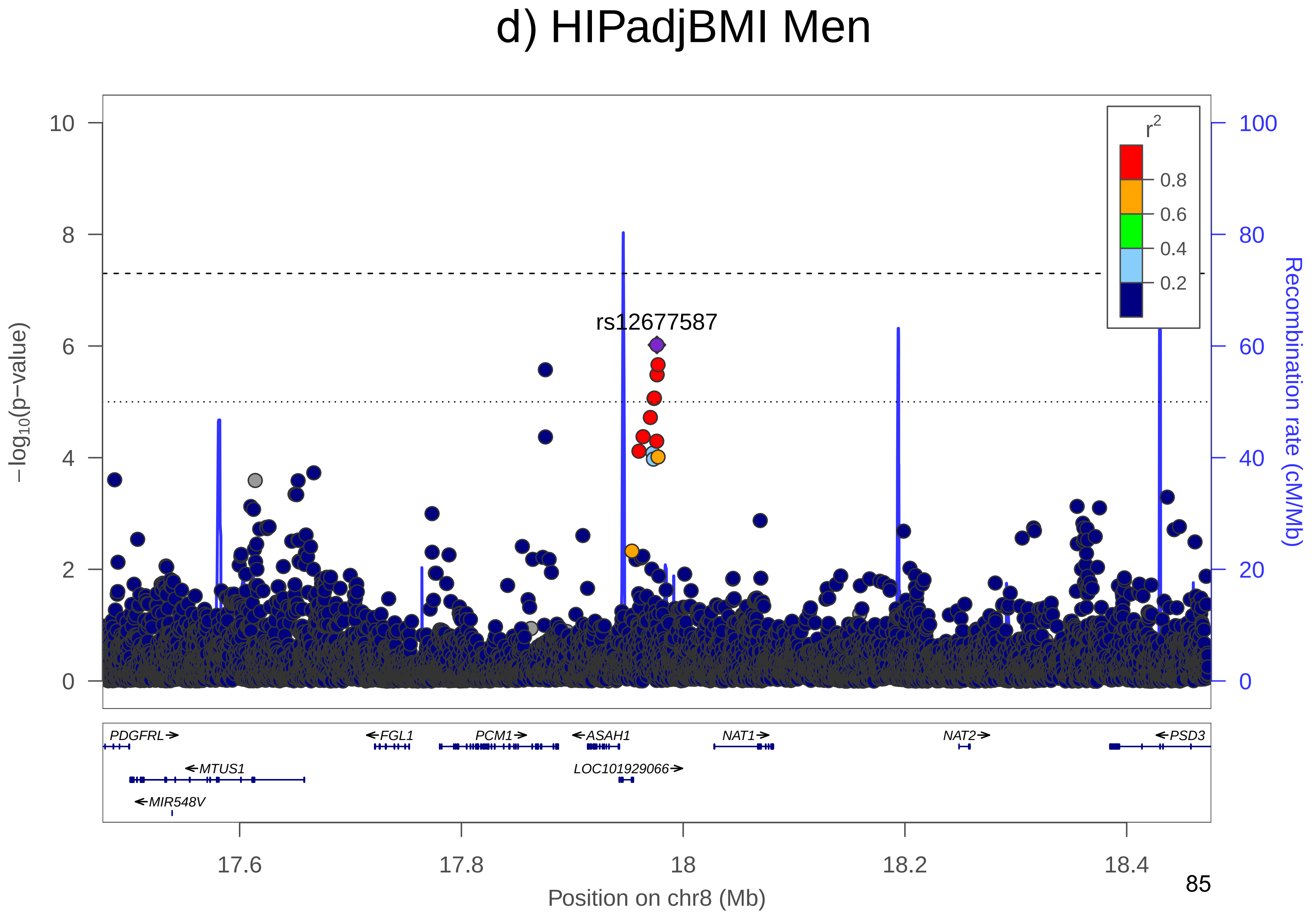

d) HIPadjBMI Men
rs12677587
−log10(p−value)
Recombination rate (cM/Mb)
r2
0.8
0.6
0.4
0.2
0
2
4
6
8
10
0
20
40
60
80
100
PDGFRL
FGL1
PCM1
ASAH1
NAT1
NAT2
PSD3
MTUS1
LOC101929066
MIR548V
17.6
17.8
18
18.2
18.4
Position on chr8 (Mb)

e) HIPadjBMI Men

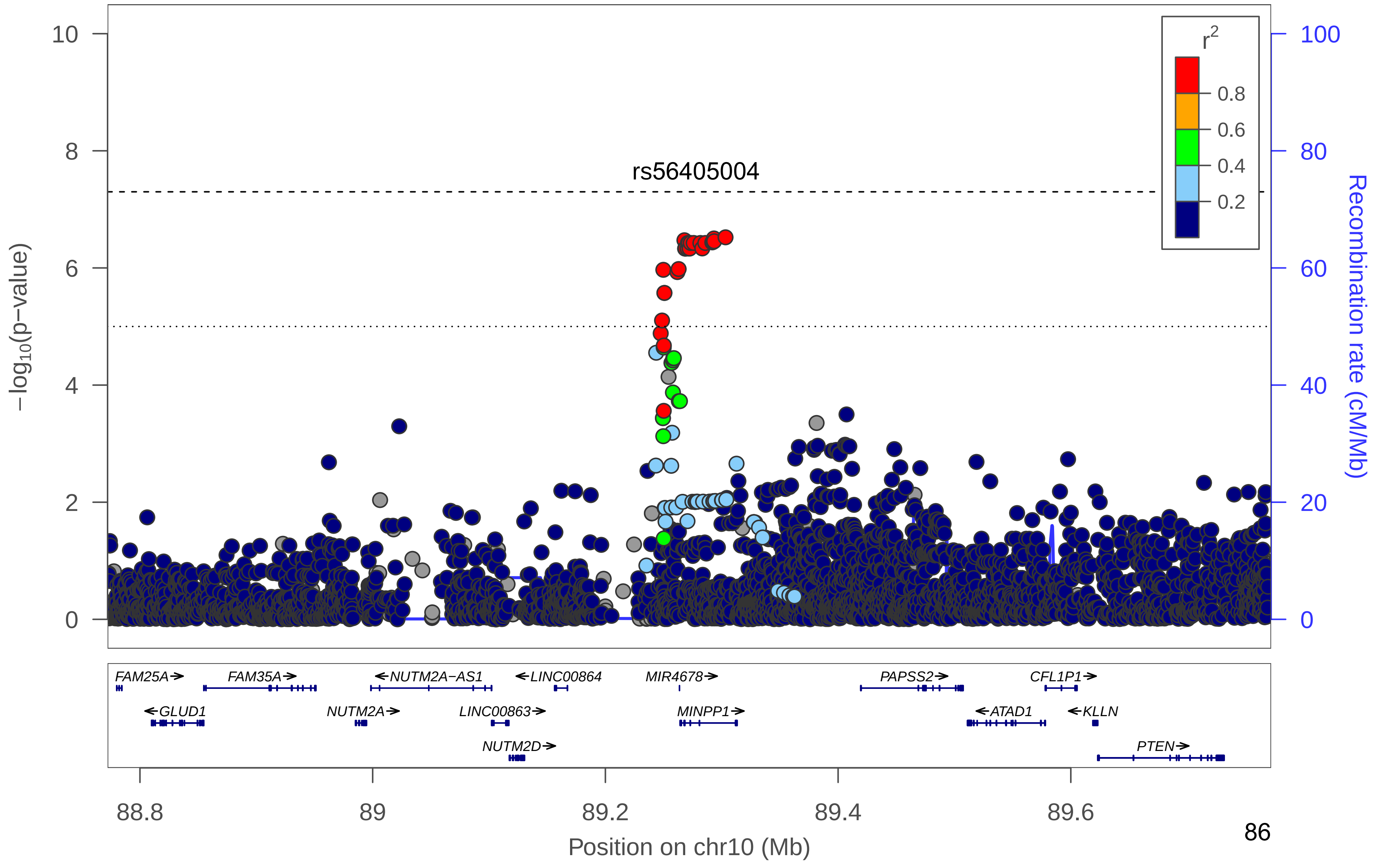

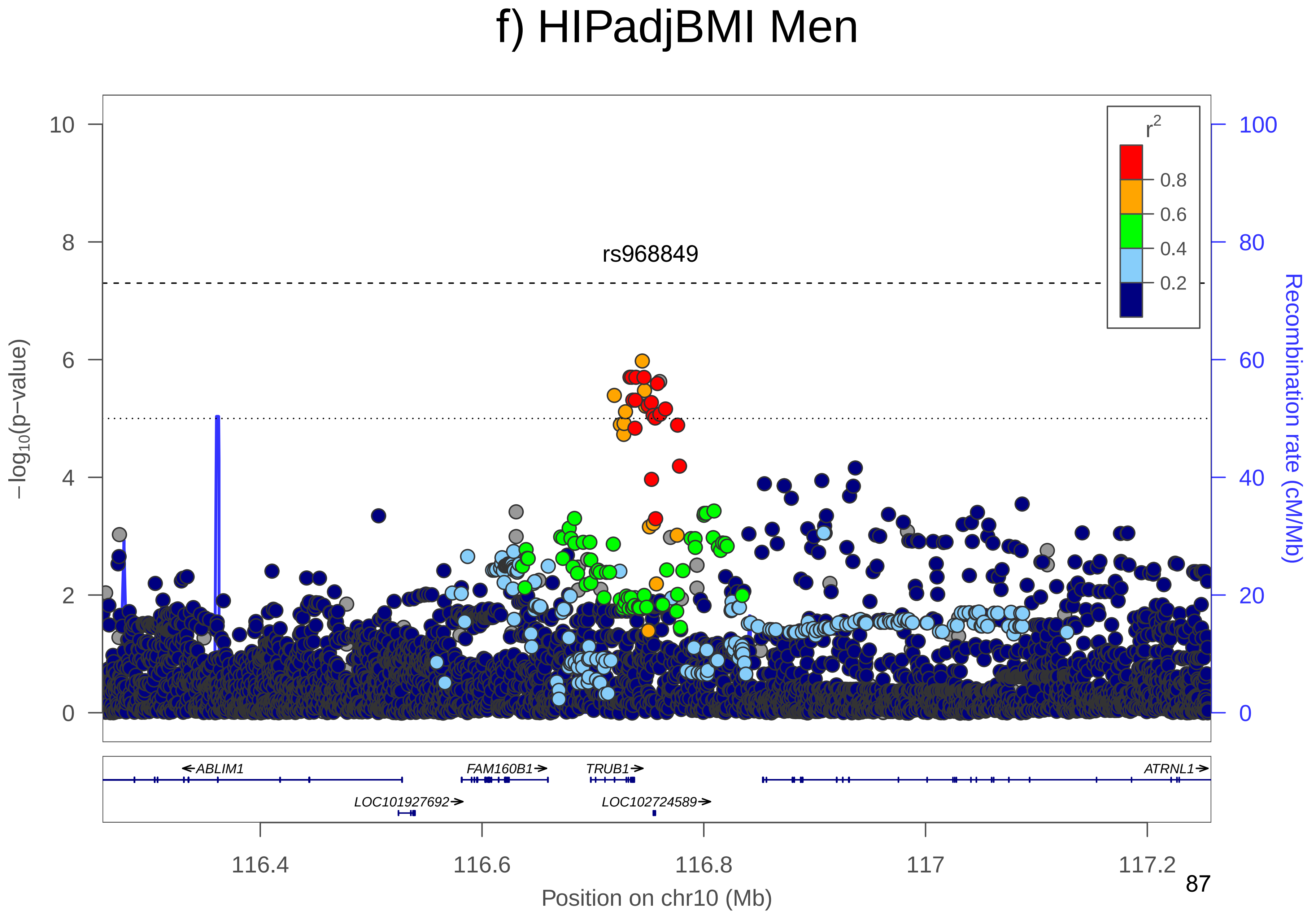

f) HIPadjBMI Men
rs968849
r2
0.8
0.6
0.4
0.2
10
8
6
4
2
0
−log10(p−value)
100
80
60
40
20
0
Recombination rate (cM/Mb)
ABLIM1
FAM160B1
TRUB1
ATRNL1
LOC101927692
LOC102724589
116.4
116.6
116.8
117
117.2
Position on chr10 (Mb)

g) HIPadjBMI Men

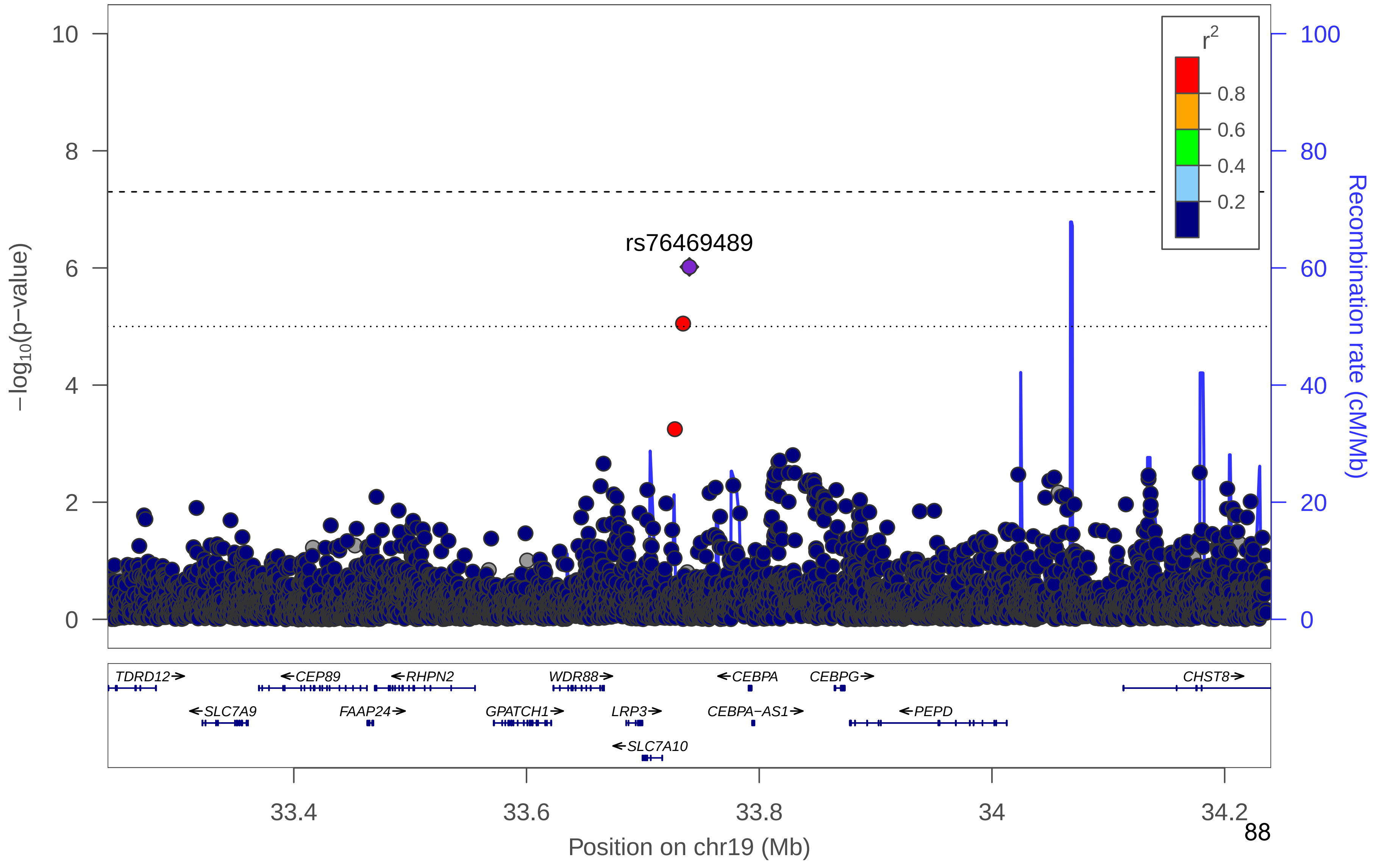

h) HIPadjBMI Men

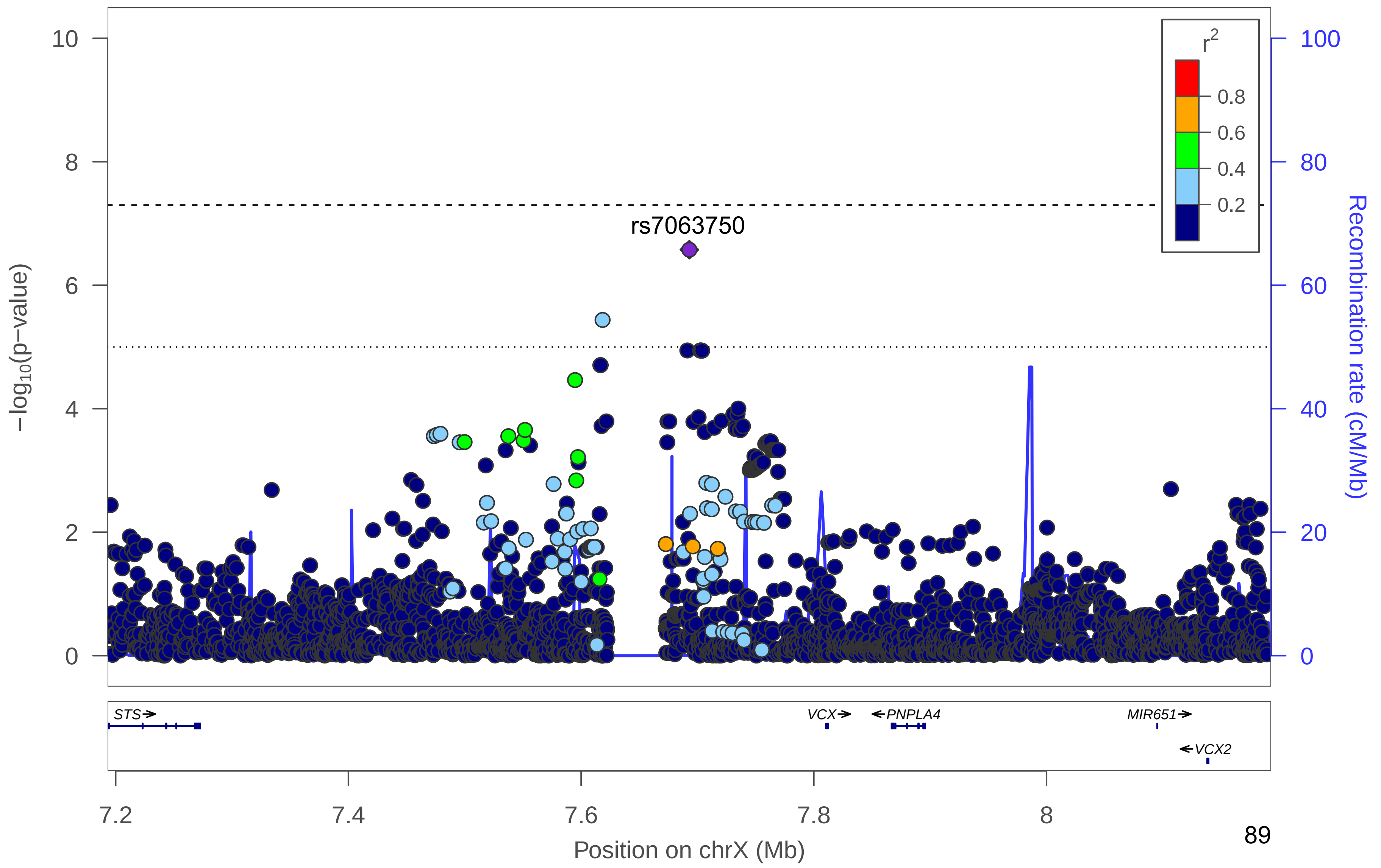

i) HIPadjBMI Men

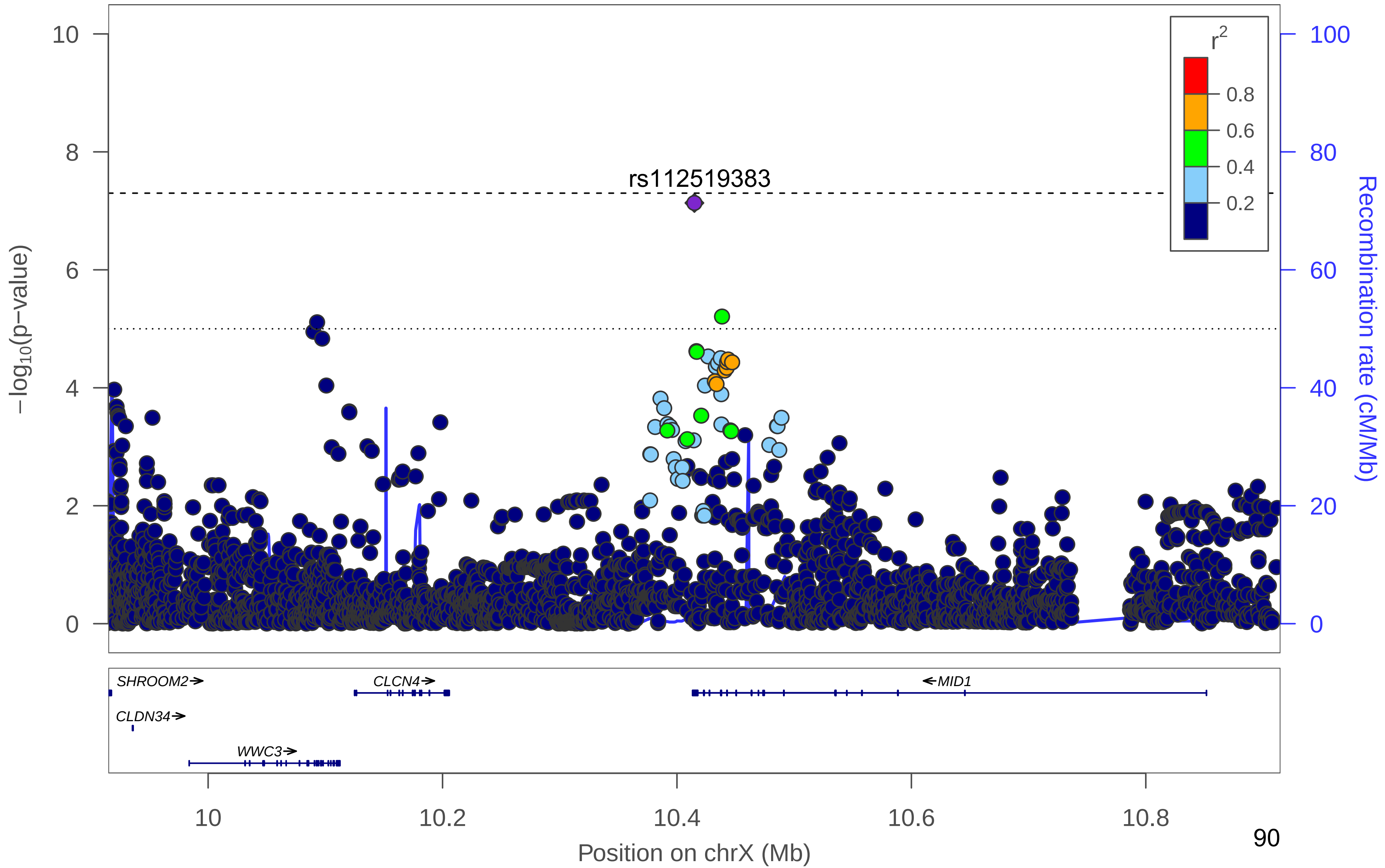

Supplementary Figure 21. QQ Plots. QQ Plots for HIPadjBMI, including sexes-combined (black), women-only (orange), men-only (blue).

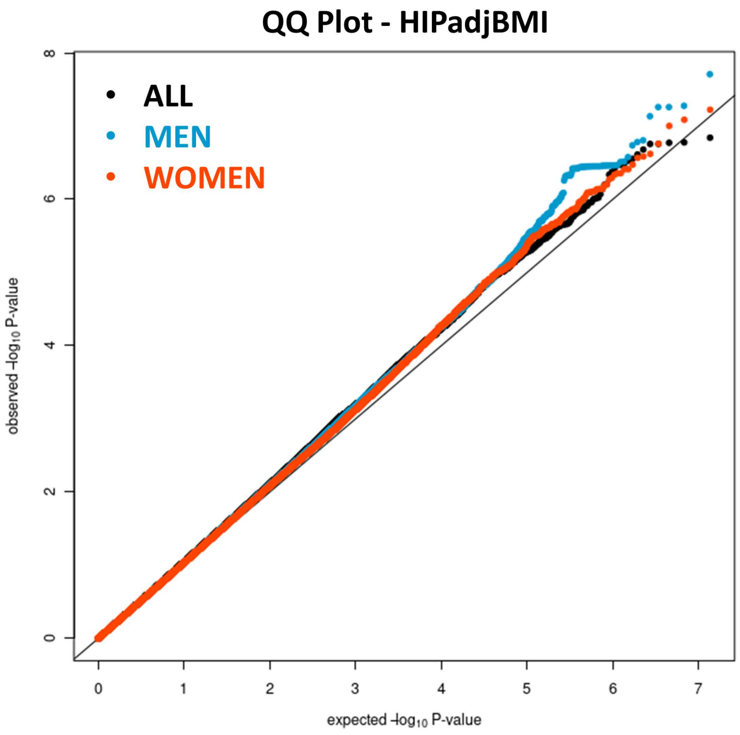

Supplementary Figure 22. Generalization of Known WHRadjBMI Loci. Comparison of the effect size estimates of known loci associated with WHRadjBMI in the sexes-combined analysis for GIANT (Shungin et al. 2015), all-ancestry GWAS, and the HCHS/SOL. Estimates are shown for all lead-gen SNPS with r-value < 0.1. Lead-gen SNPs are the SNPs with smallest r-value in a known locus. Loci were defined as regions of 1MB. Generalized SNPs (r-value<0.05) are highlighted in blue and non-generalized SNPs are highlighted in red.

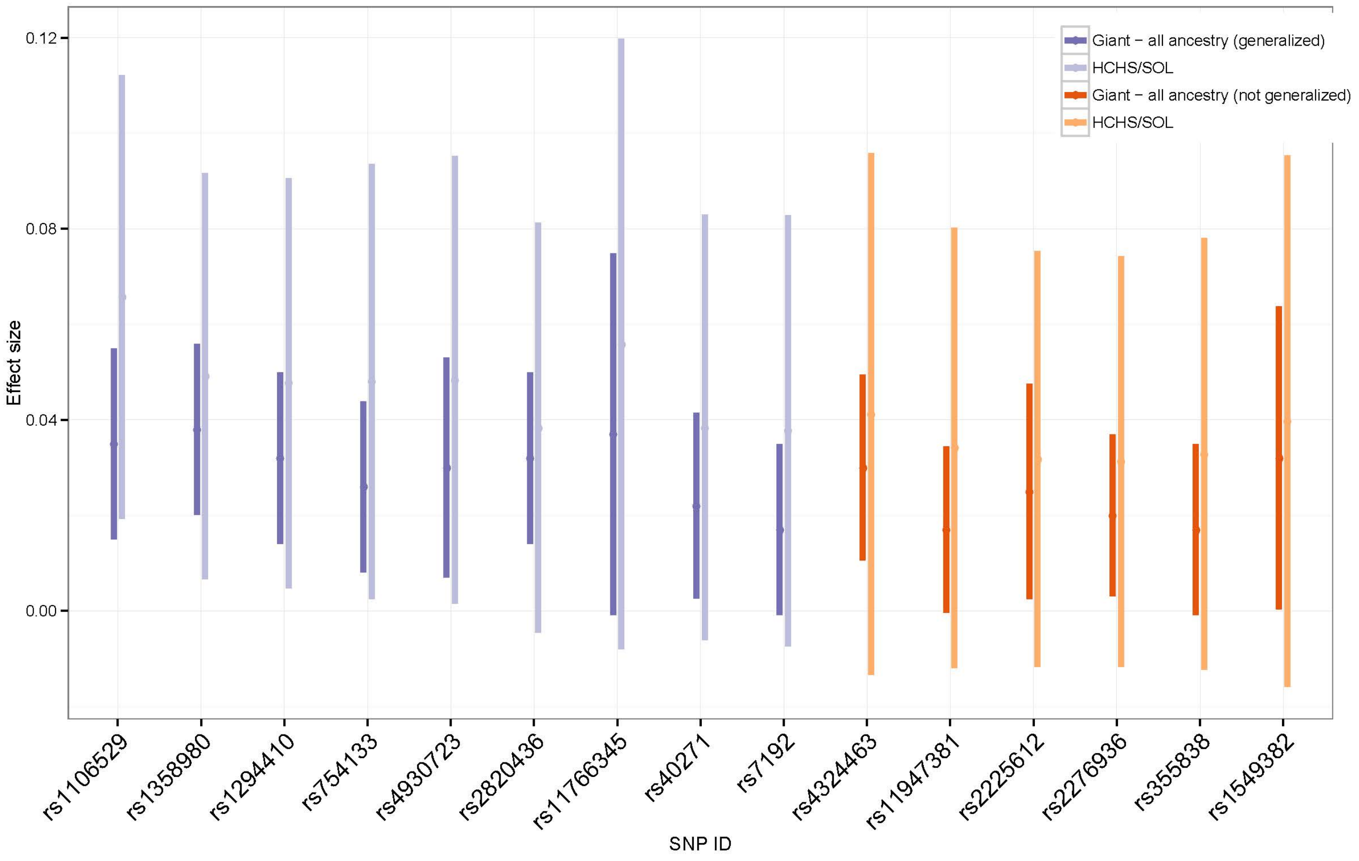

Supplementary Figure 23. Generalization of Known WHRadjBMI Loci. Comparison of the effect size estimates of known loci associated with WHRadjBMI in the women-only analysis for GIANT (Shungin et al. 2015), all-ancestry GWAS, and the HCHS/SOL women-only analysis. Estimates are shown for all lead-gen SNPS with r-value < 0.1. Lead-gen SNPs are the SNPs with smallest r-value in a known locus. Loci were defined as regions of 1MB. Generalized SNPs (r-value<0.05) are highlighted in blue and non-generalized SNPs are highlighted in red.

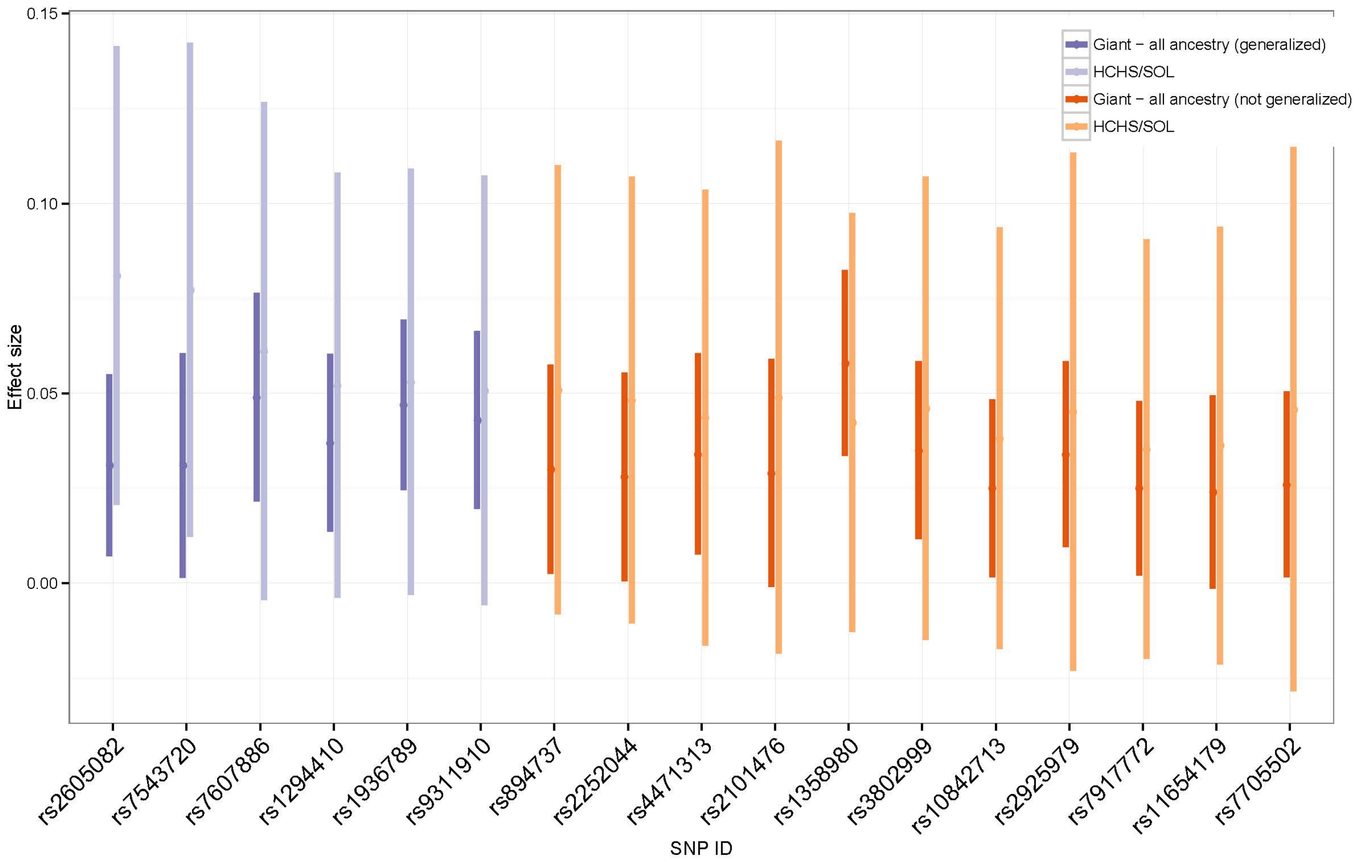

Supplementary Figure 24. Generalization of Known WHRadjBMI Loci. Comparison of the effect size estimates of known loci associated with WHRadjBMI in the men-only analysis for GIANT (Shungin et al. 2015), all-ancestry GWAS, and the HCHS/SOL men-only analysis. Estimates are shown for all lead-gen SNPS with r-value < 0.1. Lead-gen SNPs are the SNPs with smallest r-value in a known locus. Loci were defined as regions of 1MB. Generalized SNPs (r-value<0.05) are highlighted in blue and non-generalized SNPs are highlighted in red.

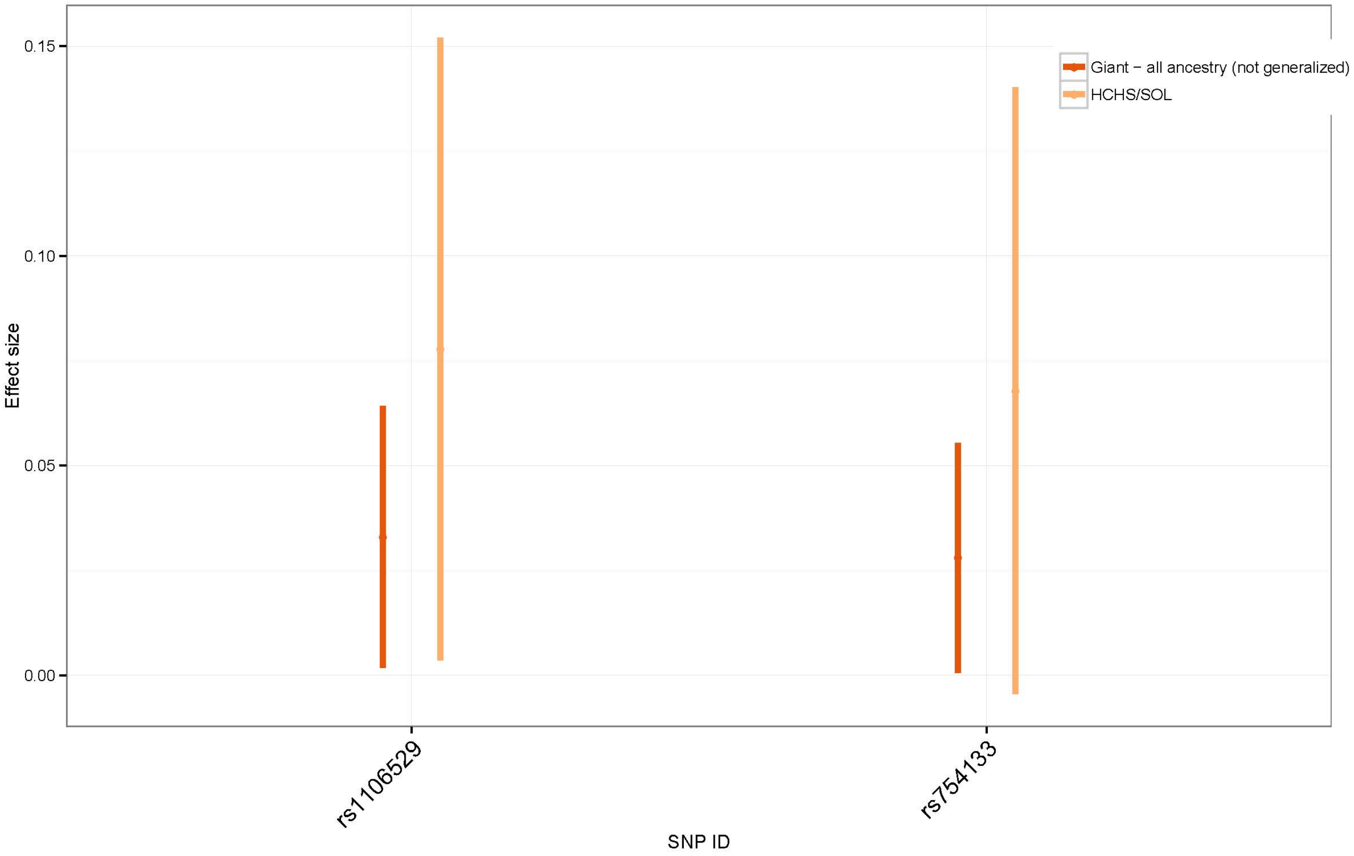

Supplementary Figure 25. Generalization of Known WCadjBMI Loci. Comparison of the effect size estimates of known loci associated with WCadjBMI in the sexes-combined analysis for GIANT (Shungin et al. 2015), all-ancestry GWAS, and the HCHS/SOL sexes-combined analysis. Estimates are shown for all lead-gen SNPS with r-value < 0.1. Lead-gen SNPs are the SNPs with smallest r-value in a known locus. Loci were defined as regions of 1MB. Generalized SNPs (r-value<0.05) are highlighted in blue and non-generalized SNPs are highlighted in red.

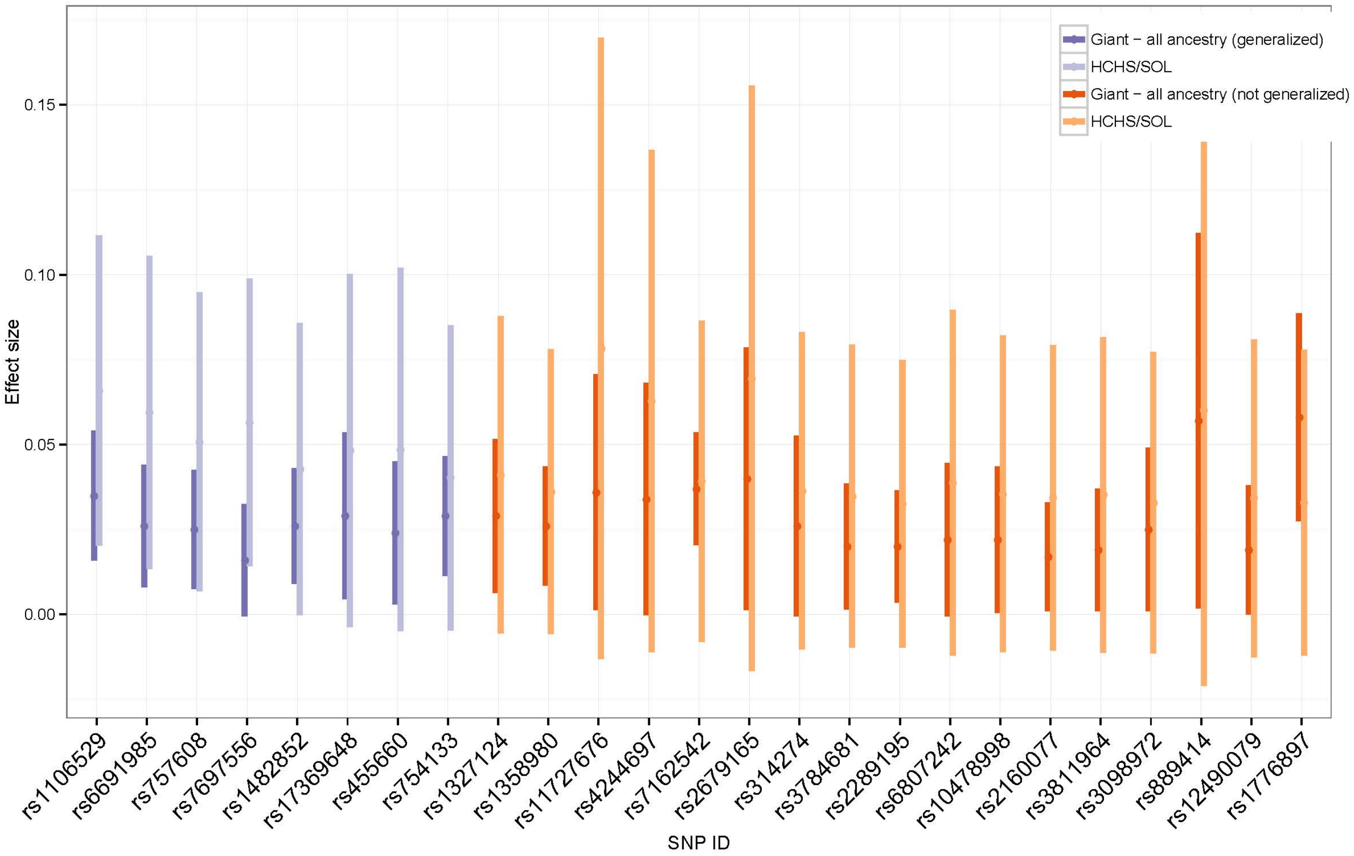

Supplementary Figure 26. Generalization of Known WCadjBMI Loci. Comparison of the effect size estimates of known loci associated with WCadjBMI in the women-only analysis for GIANT (Shungin et al. 2015), all-ancestry GWAS, and the HCHS/SOL women-only analysis. Estimates are shown for all lead-gen SNPS with r-value < 0.1. Lead-gen SNPs are the SNPs with smallest r-value in a known locus. Loci were defined as regions of 1MB. Generalized SNPs (r-value<0.05) are highlighted in blue and non-generalized SNPs are highlighted in red.

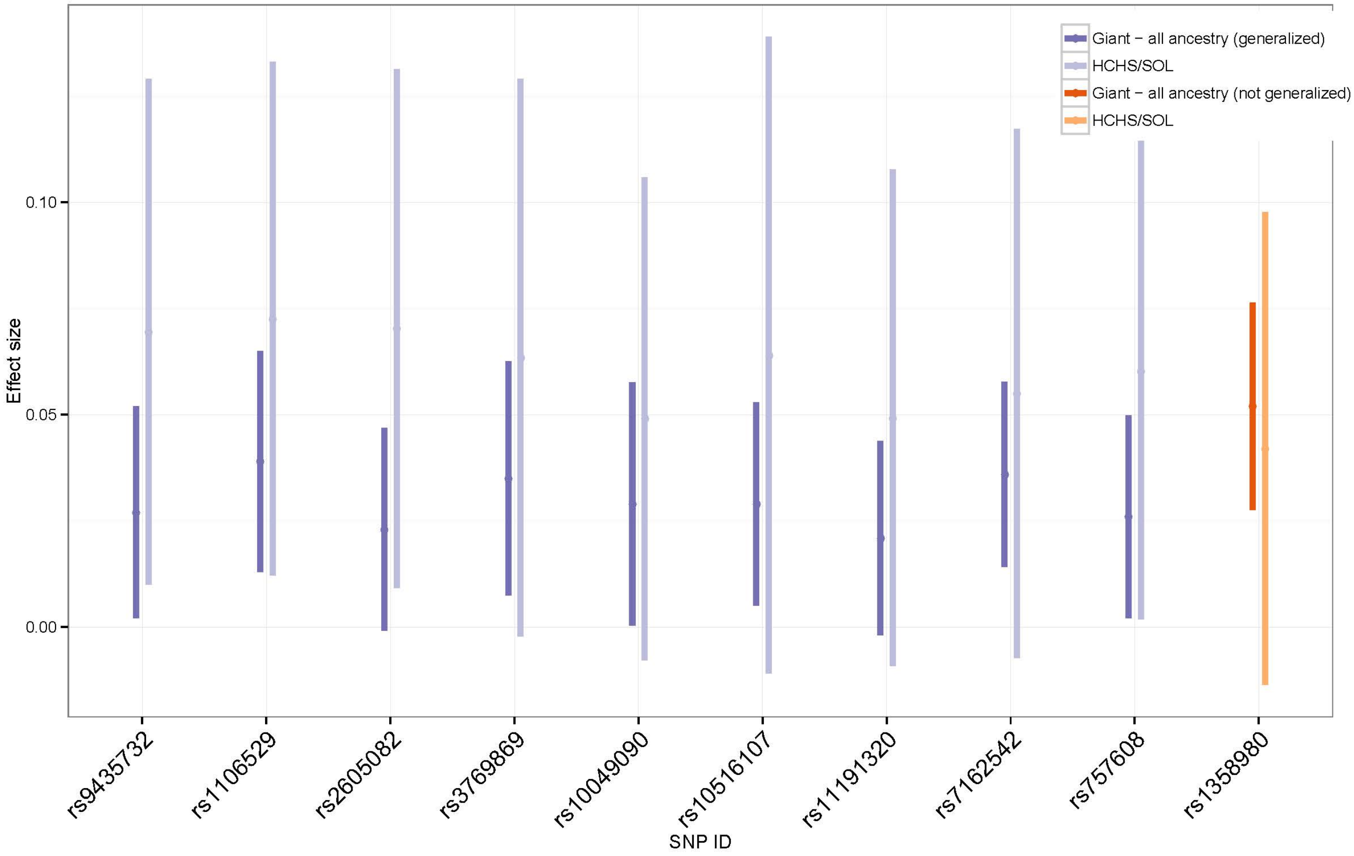

Supplementary Figure 27. Generalization of Known WCadjBMI Loci. Comparison of the effect size estimates of known loci associated with WCadjBMI in the men-only analysis for GIANT (Shungin et al. 2015), all-ancestry GWAS, and the HCHS/SOL men-only analysis. Estimates are shown for all lead-gen SNPS with r-value < 0.1. Lead-gen SNPs are the SNPs with smallest r-value in a known locus. Loci were defined as regions of 1MB. Generalized SNPs (r-value<0.05) are highlighted in blue and non-generalized SNPs are highlighted in red.

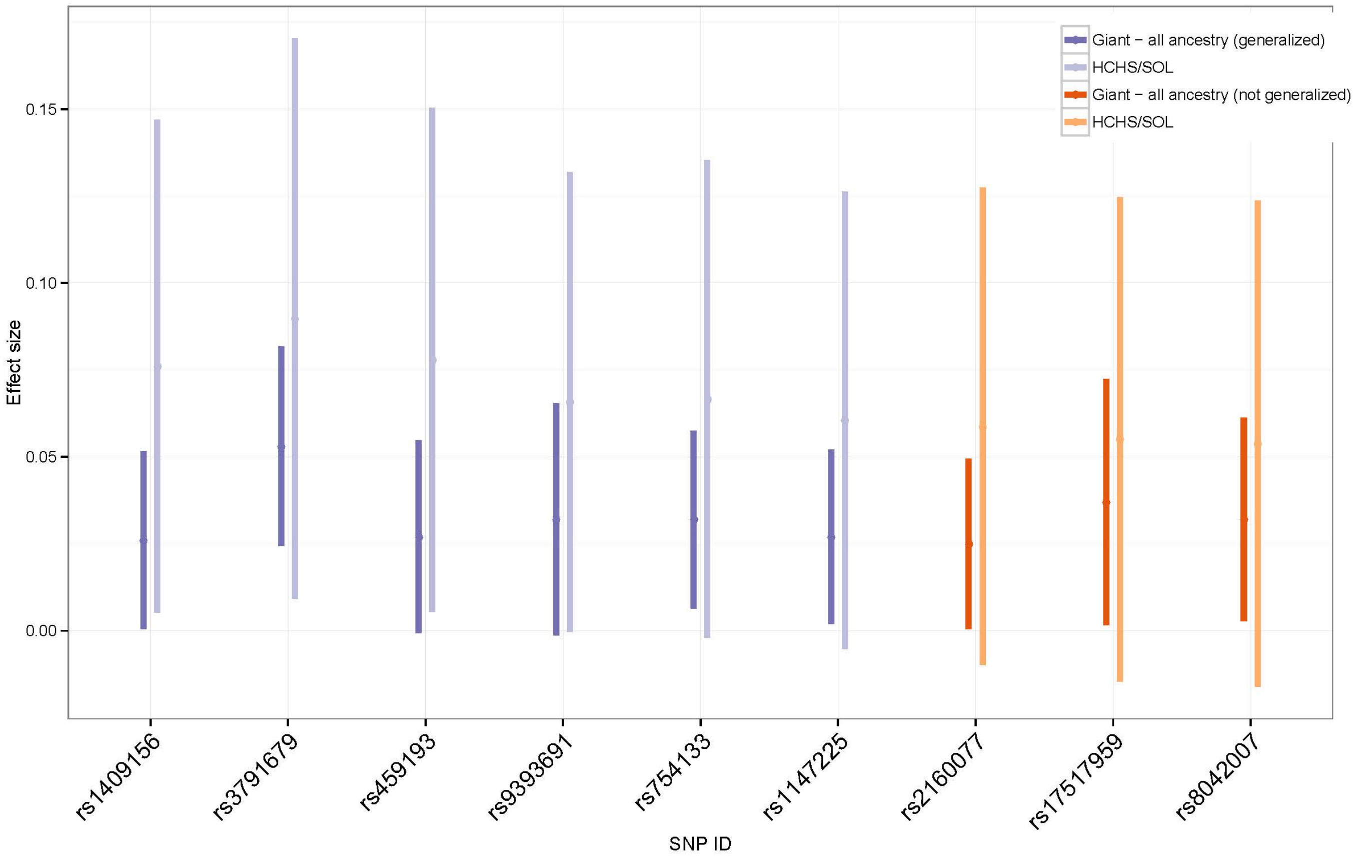

Supplementary Figure 28. Generalization of Known HIPadjBMI Loci. Comparison of the effect size estimates of known loci associated with HIPadjBMI in the sexes-combined analysis for GIANT (Shungin et al. 2015), all-ancestry GWAS, and the HCHS/SOL sexes-combined analysis. Estimates are shown for all lead-gen SNPS with r-value < 0.1. Lead-gen SNPs are the SNPs with smallest r-value in a known locus. Loci were defined as regions of 1MB. Generalized SNPs (r-value<0.05) are highlighted in blue and non-generalized SNPs are highlighted in red.

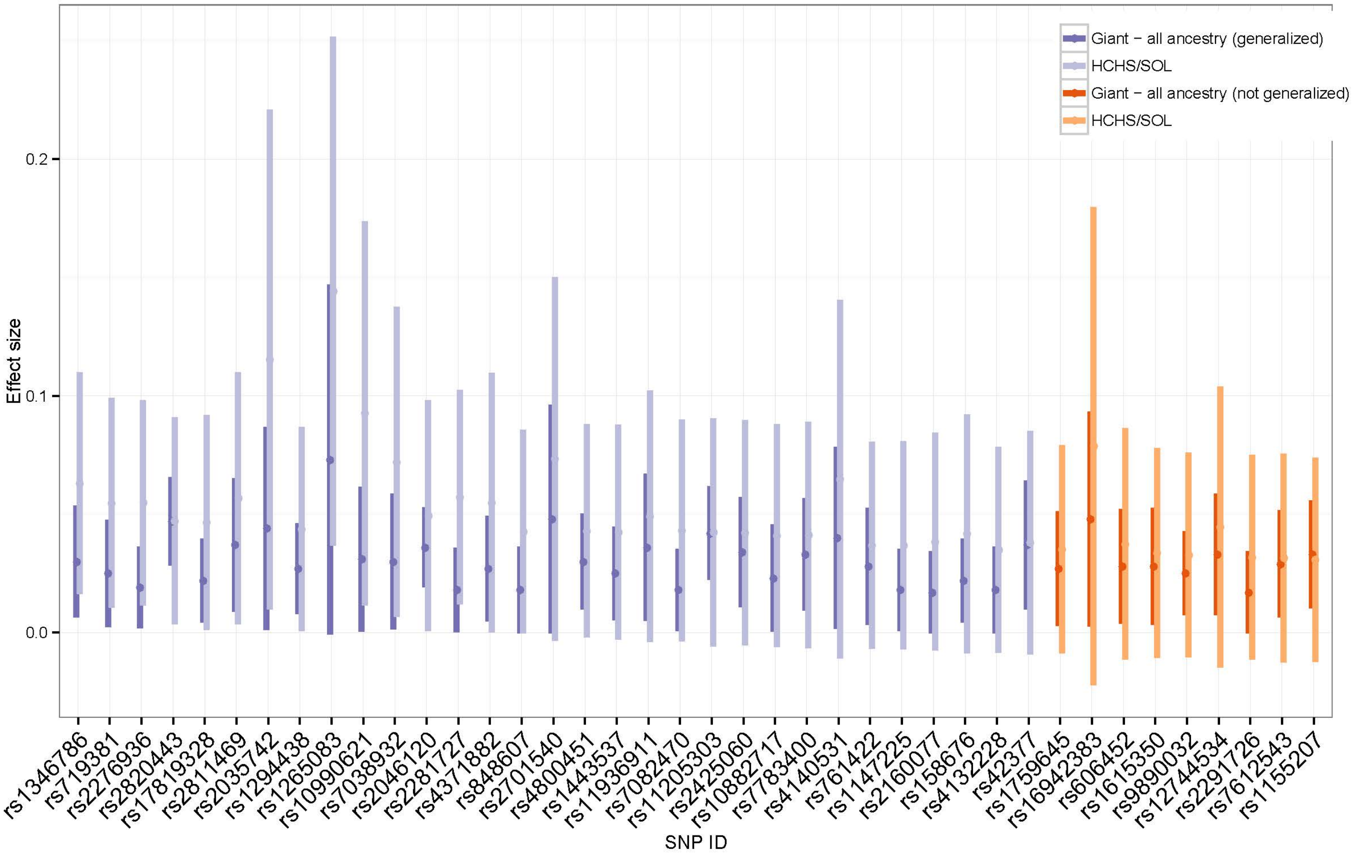

Supplementary Figure 29. Generalization of Known HIPadjBMI Loci. Comparison of the effect size estimates of known loci associated with HIPadjBMI in the women-only analysis for GIANT (Shungin et al. 2015), all-ancestry GWAS, and the HCHS/SOL women-only analysis. Estimates are shown for all lead-gen SNPS with r-value < 0.1. Lead-gen SNPs are the SNPs with smallest r-value in a known locus. Loci were defined as regions of 1MB. Generalized SNPs (r-value<0.05) are highlighted in blue and non-generalized SNPs are highlighted in red.

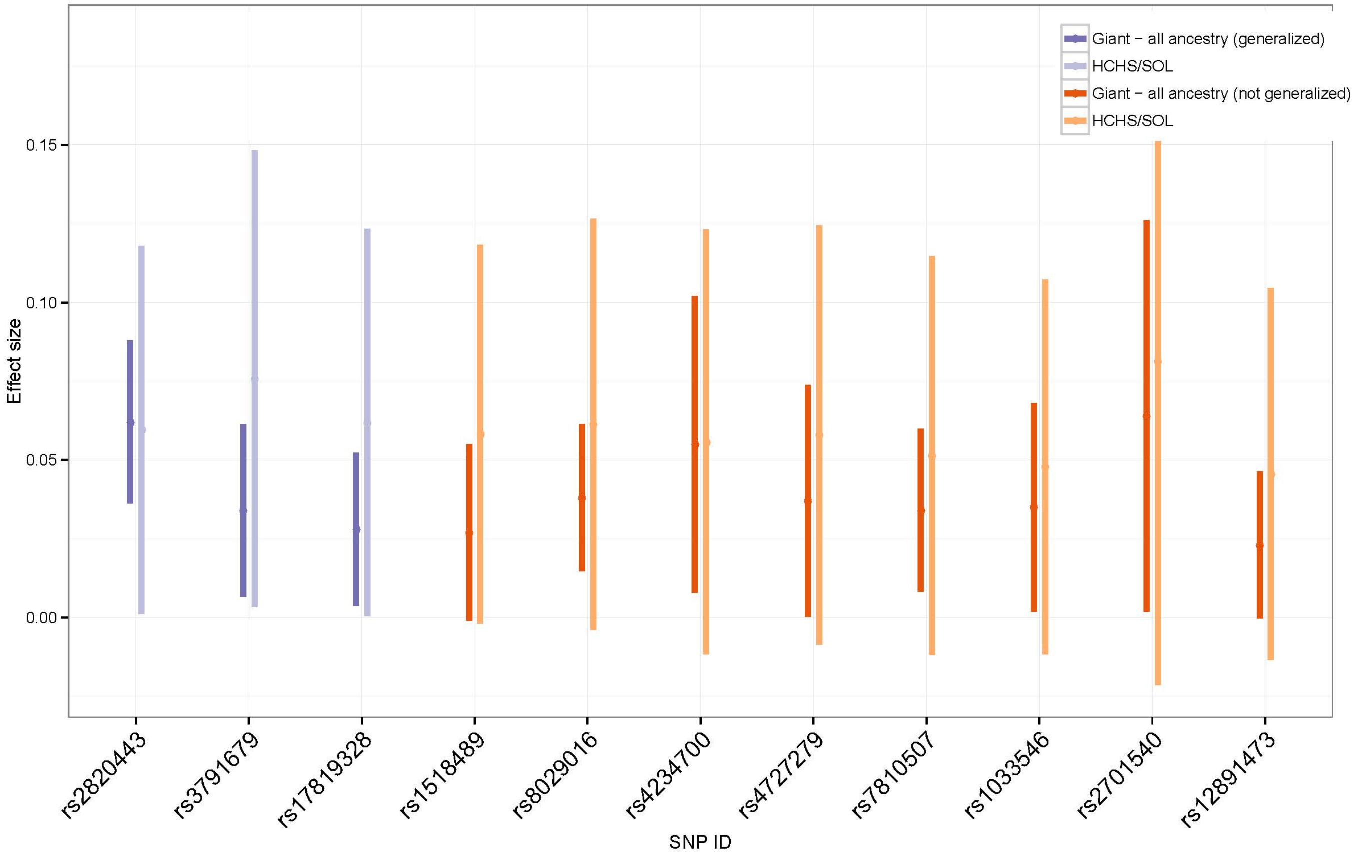

Supplementary Figure 30. Generalization of Known HIPadjBMI Loci. Comparison of the effect size estimates of known loci associated with HIPadjBMI in the men-only analysis for GIANT (Shungin et al. 2015), all-ancestry GWAS, and the HCHS/SOL men-only analysis. Estimates are shown for all lead-gen SNPS with r-value < 0.1. Lead-gen SNPs are the SNPs with smallest r-value in a known locus. Loci were defined as regions of 1MB. Generalized SNPs (r-value<0.05) are highlighted in blue and non-generalized SNPs are highlighted in red.

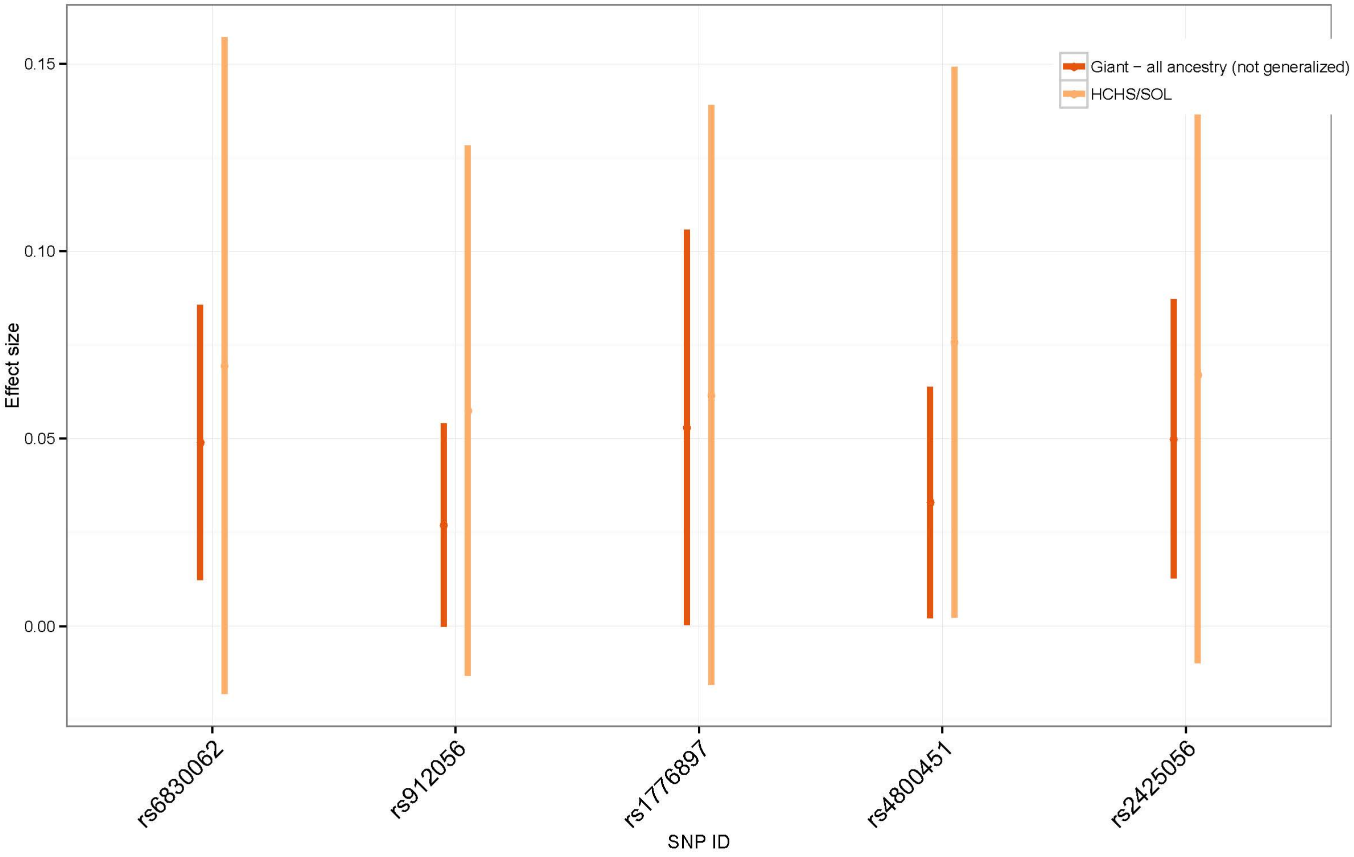

Supplementary Table 1. Study-specifc descriptive statistics for discovery and replication cohorts. Abbreviations: WC - waist circumference; HIP - hip circumference; WHR - waist-to-hip ratio; BMI - body mass index.

							MEN												WOMEN												Analysis software				
							WC			HIP			WHR			BMI			WC			HIP			WHR			BMI							
Study name	Study Abbreviation	Study design	Call rate	Study-specific exclusions	For HL, % ancestry background for study sample (Mainland, Caribbean)	Measured/ Self-reported	N	Mean	SD	N	Mean	SD	N	Mean	SD	N	Mean	SD	N	Mean	SD	N	Mean	SD	N	Mean	SD	N	Mean	SD	Analysis software - phenotype	Analysis software – SNP associations	Genotyping platform	Imputation software	References for Study Design
**Hispanic/Latino**																																			
Genetics of Latinos Diabetic Retinopathy	GOLDR	families recruited through diabete patients with eye disease	>=90%	Family QC, Gender mismatch	98.13% Mainland, 1.87% Caribbean	measured	219	103.90	14.58	54	103.06	13.44	54	0.98	0.06	223	31.03	6.25	371	107.32	14.32	84	110.38	15.72	84	0.95	0.05	374	32.63	7.02	SAS 9.4	R (GWAF)	Omni Express	SHAPEIT, Minimac3	PMCID: 3343221
Hispanic Community Health Study / Study of Latinos	HCHS/SOL	Population based cohort	>=98%	Gender mismatch; identity issues; PCA outliers	55.51% Mainland, 44.49% Caribbean	measured	5,200	99.10	13.41	5,200	103.58	10.08	5,200	0.96	0.07	5,200	29.07	5.32	7,272	97.76	14.36	7,272	108.42	13.14	7,272	0.90	0.07	7,272	30.34	6.48	R 3.2.0	R (GENESIS)	HumanOmni2.5-8v1-1 + custom content	IMPUTE2	PMIDs: 20609343, 20609344
Mexican–American Hypertension Study	HTN	families recruited through hypertensive probands.	>=95%	Family QC, Gender mismatch, Bad Concordance OMNI1 and 1S	90.43% Mainland, 9.57% Caribbean	measured	253	95.04	14.73	248	101.71	9.76	248	0.94	0.07	312	28.91	5.24	362	89.70	14.48	358	106.21	11.77	357	0.85	0.07	451	29.52	6.06	SAS 9.4	R (GWAF)	Omni Express + 1s	SHAPEIT, IMPUTEv2.3.0	PMID: 11136689
Mexican-American Coronary Artery Disease	MACAD	CAD proband, spouse of proband, and adult offspring and spouses of offspring	>=95%	Family QC, Gender mismatch, Bad Concordance OMNI1 and 1S	94.84 Mainland, 5.16% Caribbean	measured	311	96.53	11.40	311	103.41	8.92	311	0.93	0.06	346	28.92	4.52	417	90.46	13.19	416	105.69	11.89	416	0.86	0.07	457	29.00	5.58	SAS 9.4	R (GWAF)	Omni Express + 1s	SHAPEIT, IMPUTEv2.3.0	PMID: 14693718
Multi-Ethnic Study of Atherosclerosis	MESA	Population based	>=95%	Gender mismatch; PCA outliers	%67.8 Mexico ; 32.2% Caribbean	measured	720	100.92	11.43	720	102.67	8.83	720	0.98	0.05	720	28.80	4.35	777	100.87	14.72	777	108.13	11.93	777	0.93	0.07	777	30.17	5.78	SAS	SNPTEST2	AFFY6.0	IMPUTE2	PMID: 12397006; Am J Epidemiol. 2002 Nov 1;156(9):871-8
Mexico-City Diabetes study	Mexico-City Cases	Case/Control	>=99%	Related individuals; PCA outliers; Gender misn	100% Mexico	measured	360	99.03	12.42	360	101.51	10.02	360	0.97	0.07	360	28.59	5.15	534	95.82	12.14	534	106.83	12.07	534	0.90	0.06	535	30.40	5.48	SPSS	SNPTEST	Affymetrix Axiom LAT	SHAPEIT; IMPUTE2	PMID: 26780889
1982 Pelotas Birth Cohort	PELOTAS	Prospective, population-ba	≥95%	Samples excluded if there were sex mismatches (heterozygosity threshold: 0.02), heterozygosity rate outside the range of median ± 1.5 x IQR, missingness >3% and cryptic relatedness (kinship>0.1, as described in PMID:).	NA	measured	1,362	89.42	11.87	1,362	102.76	9.28	1,362	0.87	0.06	1,351	27.10	5.08	1,436	80.87	12.17	1,435	105.95	10.88	1,435	0.76	0.07	1,432	26.82	6.10	R 3.2.0	R 3.2.0	HumanOmni2.5-8v1 (Illumina)	IMPUTE2	PMID: 16373375; PMID: 25733577
Starr County Health Studies	STARR - Cases	Case/Control	0.9995	None	31% Native American	measured	274	106.43	13.22	273	105.54	10.29	273	1.01	0.06	328	30.17	5.39	413	112.14	15.57	413	114.09	14.26	413	0.98	0.07	489	32.84	6.80	Excel	SNPTEST	Affymetrix Genome-Wide Human SNP Array 6.0	IMPUTE2	PMIDs: 8640221, 21647700
Starr County Health Studies	STARR - Contr	Case/Control	0.9995	None	31% Native American	measured	223	100.86	12.88	223	106.11	9.30	223	0.95	0.06	223	29.37	5.27	556	94.79	15.08	555	110.13	13.26	553	0.86	0.07	558	30.45	6.58	Excel	SNPTEST	Affymetrix Genome-Wide Human SNP Array 6.0	IMPUTE2	PMIDs: 8640221, 21647700
Women's Health Initiative	WHI	Prospective cohort study	>=95%	first-degree relatives	Unknown	measured	NA	NA	NA	NA	NA	NA	NA	NA	NA	NA	NA	NA	3,420	86.46	12.21	3,420	105.75	11.01	3,420	0.82	0.07	3,420	28.85	5.68	STATA (v14)	PLINK/ Probable	AFFY6.0	MACH v1.0.16	PMID: 9492970
**African American**																																			
Atherosclerosis Risk in Communities Study	ARIC	Population based	>=90%	first-degree relatives ; ancestry outliers ; gender mismatch; identity issues; excessive heterozygosity; missing height or weight	N/A	measured	1,056	97.45	11.88	1,056	103.25	8.70	1,056	0.94	0.05	1,056	27.91	4.59	1,773	99.64	15.22	1,773	109.84	11.31	1,773	0.91	0.08	1,773	30.40	6.00	STATA (v14)	PLINK/FAST	Affymetrix 6.0	MACH v1.0.16	PMID: 2646917
Multi-Ethnic Study of Atherosclerosis	MESA	Population based	>=95%	Gender mismatch; PCA outliers	N/A	measured	758	100.79	12.82	758	106.03	9.76	758	0.95	0.06	758	28.76	4.74	891	101.63	16.10	891	112.94	13.07	891	0.90	0.08	891	31.32	6.41	SAS	SNPTEST2	AFFY6.0	IMPUTE2	PMID: 12397006; Am J Epidemiol. 2002 Nov 1;156(9):871-8
Women's Health Initiative	WHI	Prospective cohort study	>=95%	first-degree relatives	N/A	mreasured	NA	NA	NA	NA	NA	NA	NA	NA	NA	NA	NA	NA	8,022	91.56	13.56	8,022	111.26	13.05	8,022	0.82	0.07	8,022	31.03	6.53	STATA (v14)	PLINK/Probable	AFFY6.0	MACH v1.0.16	PMID: 9492970
**European American**																																			
Atherosclerosis Risk in Communities Study	ARIC	Population based	>=90%	first-degree relatives ; ancestry outliers ; gender mismatch; identity issues; excessive heterozygosity; missing height or weight	N/A	measured	4,281	99.62	10.21	4,281	102.71	7.25	4,281	0.97	0.05	4,281	27.42	3.89	4,795	92.75	14.34	4,795	103.92	10.31	4,795	0.89	0.08	4,795	26.47	5.27	STATA (v14)	PLINK/FAST	Affymetrix 6.0	MACH v1.0.16	PMID: 2646917

Supplementary Table 2. Association and replication results for all loci that reached suggestive significance (*P* <1x10^{-6}) in the discovery phase for WHRadjBMI. Abbreviations: CHR- chromosome number; POS- position (build GRCh38); EAF- effect allele frequency; SE- standard error.

							SOL ONLY					WITHOUT SOL																							
												EUR¥						AAⱡ						HL‡						ALLMETA					
dnSNPID	Nearest Gene	CHR	POS (GRCh38)	Known Locus (PMID)	Effect Allele	Other Allele	EAF	Beta	SE	P	N	EAF	Beta	SE	P	ISQ	N	EAF	Beta	SE	P	ISQ	N	EAF	Beta	SE	P	ISQ	N	EAF	Beta	SE	P	ISQ	N
**MEN**																																			
rs12032174	*RYR2*	1	237692868	-	T	C	0.648	0.005	0.001	4.5E-07	5,200	0.627	-0.002	0.001	1.2E-01	-	4,165	0.606	-0.001	0.002	5.5E-01	0	1,813	0.617	0.001	0.001	4.6E-01	0	4,030	0.619	-0.001	0.001	4.6E-01	0	10,008
rs10475310	*PLEKHG4B*	5	23024	-	C	G	0.748	-0.006	0.001	**2.0E-08**	5,200	-	-	-	-	-	-	0.754	-4.E-04	0.003	8.9E-01	0	758	0.749	0.001	0.001	4.4E-01	32.3	4,030	0.750	0.001	0.001	5.0E-01	24.9	4,788
rs16977373	*RIT2*	18	43189620	-	A	C	0.962	-0.013	0.003	6.6E-07	5,200	0.997	0.007	0.010	4.8E-01	-	4,165	0.793	0.001	0.002	5.5E-01	5.5	1,812	0.959	1.E-04	0.004	9.8E-01	0	2,735	0.839	0.001	0.002	5.1E-01	0	8,712
rs721424	*CFAP61*	20	20358666	-	T	G	0.484	-0.005	0.001	8.2E-07	5,200	0.618	-0.001	0.001	5.4E-01	-	4,165	0.256	1.E-04	0.002	9.8E-01	50.1	1,813	0.493	0.002	0.001	6.0E-02	29.8	4,030	0.517	0.001	0.001	3.7E-01	33.5	10,008
rs148213302	*STS*	23	7416464	-	T	C	0.995	0.025	0.005	3.7E-07	5,189	-	-	-	-	-	-	0.987	-0.012	0.007	1.2E-01	0	758	-	-	-	-	-	-	0.987	-0.012	0.007	1.2E-01	0	758
**WOMEN**																																			
rs77377042	*MARCKSL1*	1	32343334	-	T	C	0.024	0.018	0.004	8.0E-07	7,472	-	-	-	-	-	-	0.127	0.002	0.002	1.7E-01	72.5	10,684	0.021	0.006	0.004	1.8E-01	0	6,998	0.113	0.003	0.002	7.7E-02	35	17,682
rs75120960	*EPHA5*	4	66367753	-	A	G	0.98	-0.02	0.004	1.9E-07	7,472	-	-	-	-	-	-	0.882	-0.002	0.002	2.9E-01	49	10,683	0.983	-0.001	0.005	9.0E-01	69.6	5,616	0.891	-0.002	0.002	2.9E-01	52.6	16,299
rs16922424	*FAM110B*	8	57618850	-	T	C	0.982	0.021	0.004	4.9E-07	7471	-	-	-	-	-	-	0.892	-0.002	0.002	2.2E-01	0	10,684	0.984	0.004	0.005	4.4E-01	26.6	5,616	0.900	-0.002	0.002	3.4E-01	0	16,300
rs79478137	*SLC22A18AS*	11	2891739	rs116895106 (28552196)*	T	C	0.015	-0.023	0.004	2.0E-07	7,472	0.009	4.E-04	0.011	9.7E-01	-	4,678	0.084	0.001	0.002	7.7E-01	9.7	10,684	0.017	-0.012	0.005	3.1E-02	0	6,582	0.074	-0.001	0.002	6.2E-01	10.5	21,944
rs113818604	*NTM*	11	131960980	-	A	G	0.013	-0.027	0.005	5.5E-08	7,471	0.023	0.001	0.006	9.0E-01	-	4,678	0.004	0.003	0.009	7.6E-01	0	9,792	0.016	-0.003	0.005	4.6E-01	0	6,786	0.017	-0.001	0.003	7.3E-01	0	21,256
rs115981023	*TAOK3*	12	118313300	-	A	G	0.009	0.029	0.006	8.9E-07	7,472	0.003	-0.005	0.017	8.0E-01	-	4,678	0.049	0.002	0.002	3.9E-01	0	10,684	0.011	0.014	0.007	3.6E-02	71.5	5,616	0.044	0.003	0.002	1.4E-01	41.4	20,978
rs146900844	*ZNF207*	17	32378136	-	A	G	0.005	-0.041	0.008	6.3E-07	7,472	0.008	0.015	0.012	1.9E-01	-	4,678	-	-	-	-	-	-	0.007	-0.001	0.013	9.6E-01	0	1,419	0.008	0.008	0.009	3.5E-01	0	6,097
rs61305557	*C19orf67*	19	14081134	-	A	G	0.029	-0.018	0.004	4.8E-07	7,472	-	-	-	-	-	-	0.121	0.008	0.005	1.5E-01	0	1,771	0.030	0.002	0.006	7.6E-01	37.7	2,612	0.083	0.005	0.004	1.9E-01	19.1	4,383
**ALL**																																			
rs13301996	*CDK5RAP2*	9	120570806	-	T	G	0.808	0.005	0.001	5.7E-07	12,672	0.794	5.E-04	0.001	7.6E-01	-	8,845	0.872	0.004	0.001	1.1E-02	0	12,496	0.811	0.001	0.001	1.9E-01	0	12,341	0.823	0.002	0.001	1.7E-02	0	33,682
rs115981023	*TAOK3*	12	118313300	-	A	G	0.008	0.023	0.004	2.1E-07	12,672	0.003	0.002	0.012	8.7E-01	-	8,845	0.049	0.001	0.002	6.3E-01	0	12,496	0.009	0.010	0.005	5.8E-02	64.5	8,456	0.043	0.002	0.002	2.5E-01	38.4	29,797
rs185566196	*KIAA0391*	14	35152593	rs12435790 (30239722)	T	C	0.011	0.019	0.004	2.6E-07	12,672	0.003	0.003	0.017	8.8E-01	-	8,844	0.090	0.003	0.002	1.2E-01	0	12,496	0.017	-0.004	0.004	3.3E-01	44.3	9,142	0.078	0.002	0.002	2.9E-01	22.3	30,482
rs116612483	*CDH4*	20	60751325	-	A	G	0.006	0.027	0.005	6.0E-07	12,672	-	-	-	-	-	-	0.029	-0.007	0.003	1.5E-02	0	12,496	0.005	-0.007	0.007	3.2E-01	63.6	7,680	0.025	-0.007	0.003	8.9E-03	17.4	20,176

							SOL ONLY					WITH SOL																							
												EUR¥						AAⱡ						HL‡						ALLMETA					
dnSNPID	Nearest Gene	CHR	POS (GRCh38)	Known Locus (PMID)	Effect Allele	Other Allele	EAF	Beta	SE	P	N	EAF	Beta	SE	P	ISQ	N	EAF	Beta	SE	P	ISQ	N	EAF	Beta	SE	P	ISQ	N	EAF	Beta	SE	P	ISQ	N
**MEN**																																			
rs12032174	*RYR2*	1	237692868	-	T	C	0.648	0.005	0.001	4.5E-07	5,200	0.639	0.002	0.001	5.1E-03	94.9	9,365	0.638	0.004	0.001	4.6E-05	78.7	7,013	0.634	0.003	0.001	2.3E-05	33.6	9,230	0.629	0.001	0.001	1.7E-02	59	15,208
rs10475310	*PLEKHG4B*	5	23024	-	C	G	0.748	-0.006	0.001	**2.0E-08**	5,200	-	-	-	-	-	-	0.749	-0.006	0.001	**8.5E-09**	65	5,958	0.748	-0.003	0.001	2.5E-05	70.6	9,230	0.749	-0.003	0.001	3.8E-05	68.2	9,988
rs16977373	*RIT2*	18	43189620	-	A	C	0.962	-0.013	0.003	6.6E-07	5,200	0.965	-0.011	0.003	8.1E-05	73.3	9,365	0.847	-0.003	0.002	5.0E-02	87.8	7,012	0.961	-0.008	0.002	9.1E-04	60.9	7,935	0.870	-0.003	0.002	1.0E-01	65.8	13,912
rs721424	*CFAP61*	20	20358666	-	T	G	0.484	-0.005	0.001	8.2E-07	5,200	0.541	-0.003	0.001	2.8E-05	87.3	9,365	0.443	-0.004	0.001	6.2E-06	69.6	7,013	0.488	-0.002	0.001	1.5E-02	73.6	9,230	0.505	-0.001	0.001	2.3E-02	67.7	15,208
rs148213302	*STS*	23	7416464	-	T	C	0.995	0.025	0.005	3.7E-07	5,189	-	-	-	-	-	-	0.993	0.014	0.004	1.1E-03	94	5,947	-	-	-	-	-	-	0.993	0.014	0.004	1.1E-03	94	5,947
**WOMEN**																																			
rs77377042	*MARCKSL1*	1	32343334	-	T	C	0.024	0.018	0.004	8.0E-07	7,472	-	-	-	-	-	-	0.112	0.005	0.002	3.0E-03	85.4	18,156	0.023	0.012	0.003	2.9E-05	33.6	14,470	0.101	0.005	0.001	1.1E-03	64.1	25,154
rs75120960	*EPHA5*	4	66367753	-	A	G	0.98	-0.02	0.004	1.9E-07	7,472	-	-	-	-	-	-	0.895	-0.004	0.002	4.9E-03	86.4	18,155	0.981	-0.012	0.003	7.4E-05	81.1	13,088	0.902	-0.004	0.001	6.2E-03	79.4	23,771
rs16922424	*FAM110B*	8	57618850	-	T	C	0.982	0.021	0.004	4.9E-07	7471	-	-	-	-	-	-	0.905	0.001	0.002	4.0E-01	89.7	18,155	0.983	0.015	0.003	3.4E-06	67.8	13,087	0.911	0.002	0.002	3.1E-01	81.3	23,771
rs79478137	*SLC22A18AS*	11	2891739	rs116895106 (28552196)*	T	C	0.015	-0.023	0.004	2.0E-07	7,472	0.014	-0.020	0.004	6.7E-08	74.5	12,150	0.070	-0.005	0.002	1.6E-02	89.9	18,156	0.016	-0.019	0.003	**3.6E-09**	2.8	14,054	0.063	-0.005	0.002	3.4E-03	73.3	29,416
rs113818604	*NTM*	11	131960980	-	A	G	0.013	-0.027	0.005	5.5E-08	7,471	0.018	-0.015	0.004	7.6E-05	92.6	12,149	0.011	-0.020	0.004	5.1E-06	76.7	17,263	0.015	-0.014	0.003	2.7E-05	63.8	14,257	0.016	-0.009	0.003	1.1E-03	62.1	28,727
rs115981023	*TAOK3*	12	118313300	-	A	G	0.009	0.029	0.006	8.9E-07	7,472	0.008	0.025	0.006	7.3E-06	69.9	12,150	0.044	0.006	0.002	1.0E-02	83	18,156	0.010	0.022	0.005	5.7E-07	69.4	13,088	0.040	0.006	0.002	2.4E-03	73.6	28,450
rs146900844	*ZNF207*	17	32378136	-	A	G	0.005	-0.041	0.008	6.3E-07	7,472	0.006	-0.023	0.007	4.3E-04	93.6	12,150	-	-	-	-	-	-	0.006	-0.030	0.007	1.0E-05	85.2	8,891	0.006	-0.019	0.006	1.5E-03	88.8	13,569
rs61305557	*C19orf67*	19	14081134	-	A	G	0.029	-0.018	0.004	4.8E-07	7,472	-	-	-	-	-	-	0.062	-0.009	0.003	6.3E-03	93.3	9,243	0.029	-0.012	0.003	2.7E-04	70.9	10,084	0.055	-0.007	0.003	2.2E-02	80.4	11,855
**ALL**																																			
rs13301996	*CDK5RAP2*	9	120570806	-	T	G	0.808	0.005	0.001	5.7E-07	12,672	0.804	0.004	0.001	1.6E-05	84.5	21,517	0.830	0.005	0.001	**2.9E-08**	0	25,168	0.809	0.003	0.001	6.1E-06	19.5	25,013	0.818	0.003	0.001	1.1E-06	13.1	46,354
rs115981023	*TAOK3*	12	118313300	-	A	G	0.008	0.023	0.004	2.1E-07	12,672	0.008	0.021	0.004	**3.6E-08**	63.8	21,517	0.040	0.006	0.002	2.0E-03	87.6	25,168	0.009	0.018	0.003	**1.0E-08**	67.4	21,128	0.036	0.006	0.002	4.2E-04	76	42,469
rs185566196	*KIAA0391*	14	35152593	rs12435790 (30239722)	T	C	0.011	0.019	0.004	2.6E-07	12,672	0.011	0.018	0.004	3.2E-06	0	21,516	0.077	0.005	0.002	9.1E-04	79.8	25,168	0.014	0.008	0.003	6.9E-03	78.6	21,814	0.069	0.004	0.002	6.2E-03	66	43,154
rs116612483	*CDH4*	20	60751325	-	A	G	0.006	0.027	0.005	6.0E-07	12,672	-	-	-	-	-	-	0.023	0.002	0.003	4.7E-01	91.4	25,168	0.006	0.015	0.004	1.6E-04	85.9	20,352	0.021	0.001	0.002	7.5E-01	85.7	32,848

ⱡAfrican American (AA) replication samples included : Atherosclerosis Risk in Communities(ARIC), Multi-Ethnic Study of Atherosclerosis(MESA), Women's Health Initiative(WHI)

‡Hispanic Latino (HL) replication samples included : Genetics of Latinos Diabetic Retinopathy(GOLDR), Hispanic Community Health Study / Study of Latinos(HCHS/SOL), Mexican–American Hypertension Study(HTN), Mexican-American Coronary Artery Disease(MACAD), Multi-Ethnic Study of Atherosclerosis(MESA), Mexico-City, 1982 Pelotas Birth Cohort(PELOTAS), Starr County Health Studies(STARR), Women's Health Initiative(WHI)

¥European American (EUR) replication samples included : Atherosclerosis Risk in Communities(ARIC)

* Not genome-wide significant in original publication

Supplementary Table 3. Association and replication results for all loci that reached suggestive significance ($P <1x10^{-6}$) in the discovery phase for WCadjBMI. Abbreviations: CHR- chromosome number; POS- position (build GRCh38); EAF- effect allele frequency; SE- standard error.

							SOL ONLY					WITHOUT SOL																							
												EUR[¥]						AA[ǂ]						HL[‡]						ALLMETA					
dnSNPID	Nearest Gene	CHR	POS (GRCh38)	Known Locus (PMID)	Effect Allele	Other Allele	EAF	Beta	SE	P	N	EAF	Beta	SE	P	ISQ	N	EAF	Beta	SE	P	ISQ	N	EAF	Beta	SE	P	ISQ	N	EAF	Beta	SE	P	ISQ	N
MEN																																			
rs74346221	*GABRD*	1	2029024	-	T	G	0.019	-2.023	0.410	8.1E-07	5,202	0.002	2.847	4.337	5.1E-01	-	4,165	0.083	-2.883	1.361	**3.4E-02**	0	1,054	0.017	0.240	0.663	7.2E-01	0	1,344	0.029	-0.360	0.596	5.5E-01	76.5	2,398
rs72693785	*ATP1A1*	1	116334965	-	T	C	0.006	-4.088	0.734	**2.5E-08**	5,203	0.008	-0.806	1.660	6.3E-01	-	4,165	-	-	-	-	-	-	-	-	-	-	-	-	0.008	-0.806	1.660	6.3E-01	0	4,165
rs11583298	*ESRRG*	1	217171261	-	T	C	0.822	-0.711	0.140	4.0E-07	5,203	0.899	-0.477	0.378	2.1E-01	-	4,166	0.977	-1.496	0.820	6.8E-02	0	1,813	0.816	0.076	0.155	6.2E-01	0	4,206	0.833	-0.048	0.142	7.3E-01	8.7	10,185
rs76842062	*MAP4K4*	2	101847192	-	T	G	0.995	3.799	0.720	1.3E-07	5,203	-	-	-	-	-	-	0.946	0.452	0.531	3.9E-01	0	1,813	-	-	-	-	-	-	0.984	1.393	1.088	2.0E-01	0	1,812
rs141365360	*LOC102723448*	3	21826	-	A	G	0.007	3.154	0.629	5.3E-07	5,203	-	-	-	-	-	-	0.012	-1.676	1.672	3.2E-01	0	1,813	0.012	0.749	0.743	3.1E-01	0	1,344	0.035	0.382	0.488	4.3E-01	0	2,102
rs6809759	*PROK2*	3	71937742	rs7628338, rs6549455 (28448500); rs12330322 (25673412, 28443625, 28448500)	A	G	0.497	-0.586	0.102	**9.4E-09**	5,203	0.637	0.117	0.235	6.2E-01	-	4,166	0.394	-0.014	0.239	9.6E-01	0	1,812	0.507	0.033	0.111	7.7E-01	0	4,206	0.510	0.039	0.092	6.8E-01	0	10,184
rs76941364	*COBL*	7	52064843	-	A	G	0.989	2.443	0.498	9.4E-07	5,203	0.999	7.885	3.328	**1.8E-02**	-	4,165	0.984	1.393	1.088	2.0E-01	0	1,812	0.990	0.059	0.679	9.3E-01	0	2,064	0.963	0.421	0.415	3.1E-01	31.6	8,042
rs139139519	*SULF1*	8	69625608	-	A	G	0.012	-2.365	0.458	2.4E-07	5,203	0.012	-0.941	1.113	4.0E-01	-	4,166	0.052	0.105	0.647	8.7E-01	0	758	0.016	0.132	0.550	8.1E-01	0	2,375	0.015	-0.207	0.473	6.6E-01	0	8,354
rs35569658	*RBFOX1*	16	5754433	-	C	G	0.313	-0.562	0.112	5.3E-07	5,203	0.234	-0.167	0.272	5.4E-01	-	4,166	0.148	0.025	0.344	9.4E-01	3.8	1,813	0.341	-0.123	0.167	4.6E-01	32.2	2,862	0.288	-0.111	0.132	4.0E-01	13.5	8,841
rs148280037	*SHISA9*	16	12886370	-	T	C	0.984	2.081	0.412	4.5E-07	5,203	0.989	-0.648	1.114	5.6E-01	-	4,166	0.984	0.809	1.117	4.7E-01	0	1,813	0.986	0.053	0.581	9.3E-01	52.6	2,338	0.986	0.062	0.468	8.9E-01	10.2	8,317
rs143565319	*PIK3C3*	18	40962346	-	T	C	0.989	2.669	0.497	7.8E-08	5,203	-	-	-	-	-	-	0.932	-0.614	0.475	2.0E-01	18.3	1,813	0.987	0.391	0.637	5.4E-01	0	2,064	0.951	-0.255	0.381	5.0E-01	0	3,877
WOMEN																																			
rs112469617	*FZD7*	2	201982887	-	T	C	0.009	-2.977	0.594	5.5E-07	7,471	-	-	-	-	-	-	0.034	0.085	0.397	8.3E-01	69.8	10,683	0.005	-1.154	1.295	3.7E-01	0	1,420	0.031	-0.022	0.380	9.5E-01	59.7	12,103
rs17385466	*SOX5*	12	23639004	-	T	C	0.923	1.089	0.209	1.8E-07	7,471	0.883	-0.945	0.466	**4.3E-02**	-	4,678	0.974	0.507	0.366	1.7E-01	0	10,684	0.901	0.087	0.170	6.1E-01	57.7	8,613	0.911	0.053	0.146	7.2E-01	53.1	23,975
ALL																																			
rs77993329	*ACTRT2*	1	3001535	-	A	G	0.020	-1.457	0.288	4.1E-07	12,673	0.005	-0.540	1.349	6.9E-01	-	8,845	0.095	-0.144	0.191	4.5E-01	66.9	12,497	0.013	-0.140	0.093	1.3E-01	66.2	9,803	0.029	-0.142	0.084	8.9E-02	57	31,145
rs138819965	*LINC00299*	2	8304328	-	A	G	0.994	2.706	0.525	2.5E-07	12,674	-	-	-	-	-	-	0.964	-0.095	0.274	7.3E-01	0	12,497	0.995	-0.042	0.632	9.5E-01	0	7,681	0.969	-0.087	0.251	7.3E-01	0	20,178
rs77319470	*ADAMTS3*	4	72638360	rs7697556 (28443625); rs10518107 (26785701); rs16848284 (28448500)	T	G	0.371	0.406	0.082	8.6E-07	12,674	0.417	0.146	0.204	4.7E-01	-	8,844	0.504	-0.198	0.110	7.3E-02	45.3	12,496	0.344	0.012	0.017	4.8E-01	56.5	12,819	0.348	0.008	0.016	6.2E-01	54.1	34,159
rs921999	*FGF5*	4	80296998	-	A	C	0.965	-1.104	0.222	6.7E-07	12,674	0.999	1.464	3.837	7.0E-01	-	8,844	0.829	0.054	0.142	7.0E-01	80.5	12,496	0.989	0.056	0.084	5.0E-01	0	12,819	0.947	0.056	0.072	4.3E-01	6.5	34,159
rs112404395	*AP3B1*	5	77964563	-	C	G	0.991	-2.220	0.412	7.1E-08	12,674	0.985	0.412	0.991	6.8E-01	-	8,844	0.997	-3.848	4.427	3.8E-01	0	2,825	0.992	0.025	0.142	8.6E-01	0	4,390	0.992	0.029	0.140	8.4E-01	0	16,059
rs77264633	*CCDC171*	9	15823920	-	A	G	0.962	-1.118	0.205	**4.6E-08**	12,674	0.928	0.161	0.385	6.8E-01	-	8,844	0.973	0.620	0.386	1.1E-01	0	12,497	0.975	-0.006	0.049	9.0E-01	44.3	12,819	0.974	0.006	0.048	9.0E-01	35.9	34,160
rs184067184	*PHF21A*	11	46189280	-	A	C	0.006	-3.612	0.702	2.7E-07	12,674	-	-	-	-	-	-	-	-	-	-	-	-	0.010	0.282	1.287	8.3E-01	0	1,497	0.010	0.282	1.287	8.3E-01	0	1,497
rs3168072	*FADS2*	11	61864038	-	A	T	0.725	0.514	0.102	5.3E-07	12,674	0.975	2.013	0.632	**1.5E-03**	-	8,845	0.977	-0.233	0.363	5.2E-01	0	12,497	0.711	0.001	0.017	9.6E-01	0	12,819	0.711	0.002	0.017	9.2E-01	22.1	34,161
rs60260780	*WSB2*	12	118041899	-	T	C	0.013	1.780	0.350	3.7E-07	12,674	0.025	0.676	0.779	3.9E-01	-	8,844	0.103	-0.062	0.178	7.3E-01	0	12,496	0.021	-0.037	0.065	5.7E-01	9.3	10,490	0.031	-0.036	0.061	5.6E-01	0	31,830

							SOL ONLY					WITH SOL																							
												EUR[¥]						AA[ǂ]						HL[‡]						ALLMETA					
dnSNPID	Nearest Gene	CHR	POS (GRCh38)	Known Locus (PMID)	Effect Allele	Other Allele	EAF	Beta	SE	P	N	EAF	Beta	SE	P	ISQ	N	EAF	Beta	SE	P	ISQ	N	EAF	Beta	SE	P	ISQ	N	EAF	Beta	SE	P	ISQ	N
MEN																																			
rs74346221	*GABRD*	1	2029024	-	T	G	0.019	-2.023	0.410	8.14E-07	5,202	0.024	-2.095	0.393	9.5E-08	0	6,256	0.024	-2.095	0.393	9.5E-08	0	6,256	0.018	-1.398	0.349	6.2E-05	88.1	6,546	0.022	-1.489	0.338	1.1E-05	79	7,600
rs72693785	*ATP1A1*	1	116334965	-	T	C	0.006	-4.088	0.734	**2.5E-08**	5,203	0.006	-3.551	0.671	1.2E-07	69.4	9,368	-	-	-	-	-	-	-	-	-	-	-	-	0.006	-3.551	0.671	1.2E-07	69.4	9,368
rs11583298	*ESRRG*	1	217171261	-	T	C	0.822	-0.711	0.140	4.0E-07	5,203	0.831	-0.683	0.131	2.0E-07	0	9,369	0.826	-0.733	0.138	1.1E-07	0	7,016	0.819	-0.358	0.104	5.7E-04	57.4	9,409	0.827	-0.383	0.100	1.2E-04	48.1	15,388
rs76842062	*MAP4K4*	2	101847192	-	T	G	0.995	3.799	0.720	1.3E-07	5,203	-	-	-	-	-	-	0.969	1.511	0.363	3.2E-05	73.9	7,016	-	-	-	-	-	-	0.992	3.066	0.601	3.3E-07	44	7,015
rs141365360	*LOC102723448*	3	21826	-	A	G	0.007	3.154	0.629	5.3E-07	5,203	-	-	-	-	-	-	0.012	-2.317	0.442	1.6E-07	0	7,016	0.009	2.151	0.480	7.5E-06	83.6	6,547	0.024	1.423	0.386	2.2E-04	84.1	7,305
rs6809759	*PROK2*	3	71937742	rs7628338, rs6549455 (28448500); rs12330322 (25673412, 28443625, 28448500)	A	G	0.497	-0.586	0.102	**9.4E-09**	5,203	0.519	-0.474	0.094	4.0E-07	86.7	9,369	0.481	-0.498	0.094	1.1E-07	59.2	7,015	0.502	-0.302	0.075	5.7E-05	54.4	9,409	0.504	-0.243	0.069	3.9E-04	49.3	15,387
rs76941364	*COBL*	7	52064843	-	A	G	0.989	2.443	0.498	9.4E-07	5,203	0.989	2.562	0.493	2.0E-07	61.8	9,368	0.992	3.066	0.601	3.3E-07	44	7,015	0.989	1.609	0.402	6.1E-05	76.1	7,267	0.974	1.249	0.319	8.9E-05	67.9	13,245
rs139139519	*SULF1*	8	69625608	-	A	G	0.012	-2.365	0.458	2.4E-07	5,203	0.012	-2.159	0.424	3.5E-07	28.6	9,369	0.029	1.671	0.451	2.1E-04	91.3	5,961	0.014	-1.343	0.352	1.4E-04	77.2	7,578	0.013	-1.321	0.329	6.0E-05	55	13,557
rs35569658	*RBFOX1*	16	5754433	-	C	G	0.313	-0.562	0.112	5.3E-07	5,203	0.302	-0.505	0.104	1.1E-06	44.6	9,369	0.297	-0.506	0.107	2.0E-06	45.5	7,016	0.322	-0.426	0.093	4.7E-06	47	8,065	0.302	-0.373	0.085	1.2E-05	40.1	14,044
rs148280037	*SHISA9*	16	12886370	-	T	C	0.984	2.081	0.412	4.5E-07	5,203	0.985	1.753	0.386	5.7E-06	81.1	9,369	0.984	1.929	0.387	6.1E-07	0	7,016	0.985	1.402	0.336	3.0E-05	75.7	7,541	0.985	1.199	0.309	1.1E-04	62.6	13,520
rs143565319	*PIK3C3*	18	40962346	-	T	C	0.989	2.669	0.497	7.8E-08	5,203	-	-	-	-	-	-	0.959	0.953	0.343	5.5E-03	91.7	7,016	0.988	1.807	0.392	4.0E-06	74.8	7,267	0.965	0.826	0.302	6.3E-03	83.8	9,080
WOMEN																																			
rs112469617	*FZD7*	2	201982887	-	T	C	0.009	-2.977	0.594	5.5E-07	7,471	-	-	-	-	-	-	0.026	-0.862	0.330	9.0E-03	24.961	18,154	0.008	-2.660	0.540	8.4E-07	38.9	8,891	0.025	-0.880	0.320	6.0E-03	84	19,574
rs17385466	*SOX5*	12	23639004	-	T	C	0.923	1.089	0.209	1.8E-07	7,471	0.916	0.749	0.191	8.6E-05	93.7	12,149	0.936	0.946	0.181	1.9E-07	2.216	18,155	0.910	0.485	0.132	2.3E-04	71.5	16,084	0.915	0.393	0.120	1.0E-03	68.4	31,446

ALL																																			
rs77993329	*ACTRT2*	1	3001535	-	A	G	0.020	-1.457	0.288	4.1E-07	12,673	0.019	-1.417	0.282	4.9E-07	0	21,518	0.072	-0.546	0.159	6.1E-04	85.3	25,170	0.013	-0.265	0.089	2.8E-03	82.2	22,476	0.028	-0.245	0.080	2.3E-03	75.1	43,818
rs138819965	*LINC00299*	2	8304328	-	A	G	0.994	2.706	0.525	2.5E-07	12,674	-	-	-	-	-	-	0.970	0.504	0.243	3.8E-02	87	25,171	0.994	1.585	0.404	8.7E-05	74.3	20,355	0.974	0.434	0.227	5.6E-02	75.2	32,852
rs77319470	*ADAMTS3*	4	72638360	rs7697556 (28443625); rs10518107 (26785701); rs16848284 (28448500)	T	G	0.371	0.406	0.082	8.6E-07	12,674	0.377	0.370	0.076	1.2E-06	28.9	21,518	0.418	0.191	0.066	3.7E-03	86.9	25,170	0.345	0.027	0.016	9.5E-02	76.7	25,493	0.349	0.023	0.016	1.5E-01	72.6	46,833
rs921999	*FGF5*	4	80296998	-	A	C	0.965	-1.104	0.222	6.7E-07	12,674	0.965	-1.095	0.222	7.7E-07	0	21,518	0.868	-0.280	0.119	1.9E-02	89.9	25,170	0.986	-0.088	0.078	2.6E-01	63.6	25,493	0.949	-0.054	0.069	4.3E-01	63.7	46,833
rs112404395	*AP3B1*	5	77964563	-	C	G	0.991	-2.220	0.412	7.1E-08	12,674	0.990	-1.832	0.380	1.5E-06	83.4	21,518	0.991	-2.234	0.410	5.2E-08	0	15,499	0.992	-0.213	0.134	1.1E-01	89.4	17,064	0.992	-0.205	0.133	1.2E-01	83	28,733
rs77264633	*CCDC171*	9	15823920	-	A	G	0.962	-1.118	0.205	**4.6E-08**	12,674	0.954	-0.836	0.181	3.9E-06	88.4	21,518	0.964	-0.736	0.181	4.8E-05	82.5	25,171	0.974	-0.067	0.048	1.6E-01	77.3	25,493	0.973	-0.053	0.047	2.6E-01	71.3	46,834
rs184067184	*PHF21A*	11	46189280	-	A	C	0.006	-3.612	0.702	2.7E-07	12,674	-	-	-	-	-	-	-	-	-	-	-	-	0.007	-2.719	0.616	1.0E-05	85.8	14,171	0.007	-2.719	0.616	1.0E-05	85.8	14,171
rs3168072	*FADS2*	11	61864038	-	A	T	0.725	0.514	0.102	5.3E-07	12,674	0.731	0.552	0.101	**4.2E-08**	81.7	21,519	0.744	0.459	0.098	2.9E-06	33	25,171	0.711	0.014	0.016	3.9E-01	67	25,493	0.712	0.015	0.016	3.6E-01	66.1	46,835
rs60260780	*WSB2*	12	118041899	-	T	C	0.013	1.780	0.350	3.7E-07	12,674	0.015	1.595	0.319	5.9E-07	40.1	21,518	0.085	0.318	0.159	4.5E-02	87.4	25,170	0.021	0.023	0.064	7.2E-01	78.6	23,164	0.030	0.018	0.060	7.7E-01	68.9	44,504

ƗAfrican American (AA) replication samples included : Atherosclerosis Risk in Communities(ARIC), Multi-Ethnic Study of Atherosclerosis(MESA), Women's Health Initiative(WHI)

‡Hispanic Latino (HL) replication samples included : Genetics of Latinos Diabetic Retinopathy(GOLDR), Hispanic Community Health Study / Study of Latinos(HCHS/SOL), Mexican–American Hypertension Study(HTN), Mexican-American Coronary Artery Disease(MACAD), Multi-Ethnic Study of Atherosclerosis(MESA), Mexico-City, 1982 Pelotas Birth Cohort(PELOTAS), Starr County Health Studies(STARR), Women's Health Initiative(WHI)

¥European American (EUR) replication samples included : Atherosclerosis Risk in Communities(ARIC)

Supplementary Table 4. Association and replication results for all loci that reached suggestive significance ($P<1x10^{-6}$) in the discovery phase for HIPadjBMI. Abbreviations: CHR- chromosome number; POS- position (build GRCh38); EAF- effect allele frequency; SE- standard error.

							SOL ONLY (Log10)					WITHOUT SOL																			
												EUR[¥]					AA[†]					HL[‡]					ALLMETA				
dbSNPID	Nearest Gene	CHR	POS (GRCh38)	Known Locus (PMID)	Effect Allele	Other Allele	EAF	Beta	SE	P	N	EAF	Beta	SE	P	N	EAF	Z	P	ISQ	N	EAF	Z	P	ISQ	N	EAF	Z	P	ISQ	N
**MEN**																															
rs114865909	*NUF2*	1	163589134	-	T	C	0.994	-0.012	0.002	8.6E-07	5,197	-	-	-	-	-	0.953	-0.974	3.3E-01	78	1,812	0.993	-0.579	5.6E-01	0	1,344	0.970	-1.116	2.7E-01	56.5	3,156
rs149681500	*ANO10*	3	43396572	-	T	C	0.007	-0.012	0.002	5.3E-08	5,197	-	-	-	-	-	0.036	-0.273	7.9E-01	0	1,812	0.006	-1.529	1.3E-01	0	1,344	0.023	-1.204	2.3E-01	0	3,156
rs3915213	*LOC101927346*	3	74024720	-	T	C	0.779	-0.002	0.000	5.6E-07	5,197	0.704	0.028	0.175	8.7E-01	4,165	0.683	0.128	9.0E-01	24.2	1,812	0.772	0.464	6.4E-01	14.3	4,027	0.728	0.453	6.5E-01	0	10,004
rs12677587	*LOC101929066*	8	18118812	-	C	G	0.012	-0.009	0.002	9.6E-07	5,197	0.002	0.009	0.964	9.9E-01	4,280	0.068	-0.053	9.6E-01	23.4	1,814	0.015	0.082	9.3E-01	0	2,064	0.020	0.023	9.8E-01	0	8,158
rs56405004	*MINPP1*	10	87512407	-	T	C	0.018	0.008	0.001	1.7E-07	5,197	-	-	-	-	-	0.092	0.036	9.7E-01	0	1,812	0.012	-2.292	2.2E-02	67.1	2,064	0.050	-1.648	9.9E-02	53.9	3,876
rs968849	*LOC102724589*	10	114997972	-	A	G	0.195	-0.002	0.000	8.2E-07	5,197	0.345	0.081	0.166	6.3E-01	4,166	0.195	-0.138	8.9E-01	0	1,813	0.205	0.627	5.3E-01	11	4,027	0.261	0.654	5.1E-01	0	10,006
rs76469489	*SLC7A10*	19	33249049	-	C	G	0.013	-0.009	0.002	9.6E-07	5,197	0.036	0.052	0.488	9.2E-01	4,166	0.008	-0.482	6.3E-01	0	1,054	0.018	0.068	9.5E-01	0	2,873	0.026	-0.057	9.5E-01	0	8,093
rs7063750	*VCX*	23	7724974	-	T	G	0.039	0.004	0.001	2.7E-07	5,186	-	-	-	-	-	0.160	-1.128	2.6E-01	0	758	0.027	-1.770	7.7E-02	0	720	0.095	-2.043	4.1E-02	0	1,478
rs112519383	*MID1*	23	10446834	-	A	G	0.988	-0.007	0.001	7.4E-08	5,186	-	-	-	-	-	0.914	-0.135	8.9E-01	0	758	0.987	0.546	5.9E-01	0	720	0.950	0.284	7.8E-01	0	1,478
**WOMEN**																															
rs72978809	*LPPR4*	1	99260939	-	A	G	0.994	0.011	0.002	4.4E-07	7,462	-	-	-	-	-	0.959	-1.051	2.9E-01	18.6	10,683	0.996	0.323	7.5E-01	70.4	4,839	0.971	-0.691	4.9E-01	39	15,522
rs12478843	*HEATR5B*	2	37080089	-	A	G	0.248	-0.002	0.000	8.2E-08	7,462	0.150	-0.288	0.303	3.4E-01	4,679	0.056	0.898	3.7E-01	0	10,683	0.256	0.476	6.3E-01	2.9	8,315	0.145	0.464	6.4E-01	0	23,677
rs115331260	*LOC105376941*	3	5864170	-	A	G	0.985	0.008	0.002	8.8E-07	7,462	0.942	0.006	0.517	9.9E-01	4,678	0.990	-0.133	8.9E-01	0	10,683	0.979	-0.525	6.0E-01	58.1	7,120	0.976	-0.382	7.0E-01	28.3	22,481
rs7662640	*LOC105374566*	4	31765535	-	T	C	0.012	0.008	0.002	6.3E-07	7,462	0.021	0.642	0.837	4.4E-01	4,678	0.009	-0.673	5.0E-01	0	1,771	0.014	-1.391	1.6E-01	42.3	7,873	0.016	-0.829	4.1E-01	37.6	14,322
rs6814739	*LINC01094*	4	78654647	-	T	C	0.569	-0.002	0.000	7.2E-07	7,462	0.750	-0.062	0.247	8.0E-01	4,679	0.258	-0.776	4.4E-01	0	10,684	0.577	-1.507	1.3E-01	27.3	8,315	0.467	-1.526	1.3E-01	6.9	23,678
rs11099588	*COQ2*	4	83280326	-	T	C	0.057	0.004	0.001	2.4E-07	7,462	0.032	0.560	0.644	3.8E-01	4,678	0.232	0.768	4.4E-01	0	10,683	0.162	-0.621	5.4E-01	58.8	8,231	0.168	0.537	5.9E-01	44.8	23,592
rs6860625	*NREP*	5	111667124	-	A	G	0.813	-0.002	0.000	6.4E-07	7,462	0.848	-0.172	0.303	5.7E-01	4,679	0.633	-0.188	8.5E-01	59.5	10,683	0.831	-1.523	1.3E-01	0	8,315	0.745	-1.281	2.0E-01	0	23,677
rs77186623	*LOC105375745*	8	125608952	-	A	C	0.022	-0.006	0.001	1.7E-07	7,461	-	-	-	-	-	0.082	-0.320	7.5E-01	38.4	10,683	0.013	-0.972	3.3E-01	0	5,616	0.058	-0.830	4.1E-01	0	16,299
rs10818474	*MEGF9*	9	120727686	rs7044106 (25673412)	T	C	0.809	-0.002	0.000	5.1E-07	7,462	0.753	0.014	0.100	8.9E-01	4,773	0.571	-1.176	2.4E-01	0	10,682	0.742	0.479	6.3E-01	0	7,345	0.664	-0.468	6.4E-01	0	22,800
rs28692724	*IRF2BPL*	14	77027445	-	T	C	0.425	0.002	0.000	7.3E-07	7,462	0.303	0.789	0.305	**9.6E-03**	4,678	0.260	-0.286	7.8E-01	68.9	10,683	0.391	-0.143	8.9E-01	0	3,260	0.294	1.021	3.1E-01	39.9	18,621
rs6092086	*LOC105372676*	20	55193568	-	A	G	0.922	-0.003	0.001	3.4E-07	7,462	0.989	1.144	1.042	2.7E-01	4,679	0.653	-0.031	9.8E-01	0	10,684	0.941	0.642	5.2E-01	21.2	8,231	0.820	0.848	4.0E-01	0.1	23,594
rs9631175	*TAF4*	20	62072648	-	A	C	0.793	0.002	0.000	6.0E-08	7462	0.881	-0.161	0.370	6.6E-01	4,679	0.755	1.676	9.4E-02	0	10,683	0.807	0.957	3.4E-01	26.8	8,315	0.798	1.499	1.3E-01	13.1	23,677
**ALL**																															
rs144655586	*CLSPN*	1	35725381	-	T	C	0.012	0.007	0.001	1.7E-07	12,658	-	-	-	-	-	-	-	-	-	-	0.009	0.738	4.6E-01	29.2	4,490	0.009	0.738	4.6E-01	29.2	4,490
rs712900	*LPPR4*	1	99261313	-	T	C	0.014	-0.006	0.001	3.4E-07	12,659	0.003	1.482	1.534	3.3E-01	8,845	0.096	2.159	3.1E-02	0	12,497	0.012	-0.447	6.6E-01	0	7,680	0.045	1.720	8.5E-02	0	29,022
rs115546449	*TMEM63A*	1	225882871	-	A	G	0.026	-0.004	0.001	3.0E-07	12,659	0.004	-0.040	1.365	9.8E-01	8,844	0.122	-0.428	6.7E-01	0	12,497	0.021	0.225	8.2E-01	14.3	9,791	0.057	-0.160	8.7E-01	0	31,132
rs145815581	*ANO10*	3	43466242	-	A	G	0.007	-0.008	0.002	1.8E-07	12,659	-	-	-	-	-	0.041	0.615	5.4E-01	32.4	12,497	0.005	-0.057	9.5E-01	0	7,680	0.027	0.448	6.5E-01	0	20,177
rs72886347	*FHIT*	3	60196461	-	T	C	0.993	0.008	0.002	7.2E-07	12,659	-	-	-	-	-	0.964	-1.609	1.1E-01	74.9	12,497	0.994	-1.119	2.6E-01	23.8	7,680	0.975	-1.957	5.0E-02	52.9	20,177
rs17136358	*EIF2AK1*	7	6040031	-	T	C	0.963	-0.003	0.001	2.8E-07	12,659	0.939	-0.369	0.286	2.0E-01	8,844	0.942	0.281	7.8E-01	0	12,497	0.966	-0.118	9.1E-01	17.1	12,342	0.950	-0.561	5.8E-01	1.1	33,683
rs117683919	*LOC105375440*	7	106634111	-	A	G	0.006	-0.009	0.002	1.4E-07	12,659	0.010	-0.020	0.747	9.8E-01	8,844	0.004	-0.852	4.0E-01	0	4,474	0.009	1.630	1.0E-01	23.7	10,871	0.008	0.710	4.8E-01	19.5	24,189
rs143542634	*PATL1*	11	59666070	-	A	G	0.006	0.009	0.002	4.3E-07	12,659	0.009	0.999	0.863	2.5E-01	8,845	0.004	1.031	3.0E-01	0	4,474	0.011	0.300	7.6E-01	0	3,541	0.008	1.507	1.3E-01	0	16,860

							SOL ONLY (Log10)					WITH SOL																			
												EUR[¥]					AA[†]					HL[‡]					ALLMETA				
dbSNPID	Nearest Gene	CHR	POS (GRCh38)	Known Locus (PMID)	Effect Allele	Other Allele	EAF	Beta	SE	P	N	EAF	Z	P	ISQ	N	EAF	Z	P	ISQ	N	EAF	Z	P	ISQ	N	EAF	Z	P	ISQ	N
**MEN**																															
rs114865909	*NUF2*	1	163589134	-	T	C	0.994	-0.012	0.002	8.6E-07	5,197	-	-	-	-	-	0.983	-4.733	2.2E-06	72.7	7,009	0.994	-4.650	3.3E-06	66	6,541	0.985	-4.568	4.9E-06	67.4	8,353
rs149681500	*ANO10*	3	43396572	-	T	C	0.007	-0.012	0.002	5.3E-08	5,197	-	-	-	-	-	0.014	-4.824	1.4E-06	68.9	7,009	0.007	-5.543	**3.0E-08**	17.9	6,541	0.013	-5.032	4.9E-07	55.4	8,353
rs3915213	*LOC101927346*	3	74024720	-	T	C	0.779	-0.002	0.000	5.6E-07	5,197	0.746	-3.622	2.9E-04	91.6	9,362	0.754	-4.246	2.2E-05	76.1	7,009	0.776	-3.451	5.6E-04	60.4	9,224	0.745	-2.560	1.0E-02	59.2	15,201
rs12677587	*LOC101929066*	8	18118812	-	C	G	0.012	-0.009	0.002	9.6E-07	5,197	0.007	-3.623	2.9E-04	90.8	9,477	0.026	-4.246	2.2E-05	72.6	7,011	0.013	-4.102	4.1E-05	72.6	7,261	0.017	-3.039	2.4E-03	69.1	13,355
rs56405004	*MINPP1*	10	87512407	-	T	C	0.018	0.008	0.001	1.7E-07	5,197	-	-	-	-	-	0.037	4.526	6.0E-06	74.5	7,009	0.016	3.207	1.3E-03	92.1	7,261	0.032	2.885	3.9E-03	85.9	9,073
rs968849	*LOC102724589*	10	114997972	-	A	G	0.195	-0.002	0.000	8.2E-07	5,197	0.262	-3.348	8.1E-04	92.5	9,363	0.195	-4.316	1.6E-05	67.7	7,010	0.199	-3.287	1.0E-03	60.7	9,224	0.239	-2.353	1.9E-02	58.5	15,203
rs76469489	*SLC7A10*	19	33249049	-	C	G	0.013	-0.009	0.002	9.6E-07	5,197	0.023	-3.579	3.4E-04	91.1	9,363	0.012	-4.666	3.1E-06	59.5	6,251	0.015	-3.892	1.0E-04	58.6	8,070	0.021	-3.109	1.9E-03	61	13,290
rs7063750	*VCX*	23	7724974	-	T	G	0.039	0.004	0.001	2.7E-07	5,186	-	-	-	-	-	0.054	4.404	1.1E-05	88	5,944	0.038	4.204	2.6E-05	91.6	5,906	0.051	3.577	3.5E-04	88.9	6,664
rs112519383	*MID1*	23	10446834	-	A	G	0.988	-0.007	0.001	7.4E-08	5,186	-	-	-	-	-	0.979	-5.076	3.9E-07	69	5,944	0.988	-4.853	1.2E-06	82.5	5,906	0.980	-4.614	3.9E-06	75	6,664
**WOMEN**																															
rs72978809	*LPPR4*	1	99260939	-	A	G	0.994	0.011	0.002	4.4E-07	7,462	-	-	-	-	-	0.974	2.433	1.5E-02	87	18,145	0.995	4.137	3.5E-05	83.2	12,301	0.978	2.310	2.1E-02	81.6	22,984
rs12478843	*HEATR5B*	2	37080089	-	A	G	0.248	-0.002	0.000	8.2E-08	7,462	0.210	-4.793	1.6E-06	85	12,141	0.135	-2.750	6.0E-03	87.4	18,145	0.252	-3.343	8.3E-04	63.1	15,777	0.169	-2.221	2.6E-02	62.1	31,139
rs115331260	*LOC105376941*	3	5864170	-	A	G	0.985	0.008	0.002	8.8E-07	7,462	0.968	3.862	1.1E-04	89.2	12,140	0.988	3.051	2.3E-03	80.5	18,145	0.982	3.150	1.6E-03	77.3	14,582	0.979	2.123	3.4E-02	69.1	29,943
rs7662640	*LOC105374566*	4	31765535	-	T	C	0.012	0.008	0.002	6.3E-07	7,462	0.016	4.382	1.2E-05	83.9	12,140	0.011	4.184	2.9E-05	87.1	9,233	0.013	2.478	1.3E-02	75.6	15,335	0.014	2.243	2.5E-02	71.3	21,784
rs6814739	*LINC01094*	4	78654647	-	T	C	0.569	-0.002	0.000	7.2E-07	7,462	0.639	-4.040	5.3E-05	87.9	12,141	0.386	-3.773	1.6E-04	74.8	18,146	0.573	-4.502	6.7E-06	47.2	15,777	0.492	-3.756	1.7E-04	47.6	31,140
rs11099588	*COQ2*	4	83280326	-	T	C	0.057	0.004	0.001	2.4E-07	7,462	0.047	4.589	4.5E-06	84.3	12,140	0.160	3.902	9.5E-05	76.8	18,145	0.112	3.112	1.9E-03	75.5	15,693	0.141	3.000	2.7E-03	67.3	31,054
rs6860625	*NREP*	5	111667124	-	A	G	0.813	-0.002	0.000	6.4E-07	7,462	0.827	-4.255	2.1E-05	85.7	12,141	0.707	-3.337	8.5E-04	83.9	18,145	0.823	-4.530	5.9E-06	22.7	15,777	0.761	-3.554	3.8E-04	46.4	31,139
rs77186623	*LOC105375745*	8	125608952	-	A	C	0.022	-0.006	0.001	1.7E-07	7,461	-	-	-	-	-	0.058	-3.596	3.2E-04	83.1	18,144	0.018	-4.584	4.6E-06	59.6	13,077	0.047	-3.615	3.0E-04	67.9	23,760

rs10818474	*MEGF9*	9	120727686	rs7044106 (25673412)	T	C	0.809	-0.002	0.000	5.1E-07	7,462	0.787	-3.834	1.3E-04	90.5	12,235	0.669	-4.123	3.7E-05	71.3	18,144	0.776	-3.228	1.3E-03	62	14,807	0.700	-2.900	3.7E-03	52.6	30,262
rs28692724	*IRF2BPL*	14	77027445	-	T	C	0.425	0.002	0.000	7.3E-07	7,462	0.378	5.490	**4.0E-08**	8.3	12,140	0.328	2.956	3.1E-03	86.6	18,145	0.415	4.053	5.1E-05	45.8	10,722	0.331	3.512	4.5E-04	66.1	26,083
rs6092086	*LOC105372676*	20	55193568	-	A	G	0.922	-0.003	0.001	3.4E-07	7,462	0.948	-3.318	9.1E-04	93.8	12,141	0.764	-3.296	9.8E-04	81.4	18,146	0.932	-3.053	2.3E-03	67	15,693	0.845	-1.762	7.8E-02	63.5	31,056
rs9631175	*TAF4*	20	62072648	-	A	C	0.793	0.002	0.000	6.0E-08	7462	0.827	3.978	7.0E-05	92.7	12,141	0.770	4.762	1.9E-06	71.4	18,145	0.800	4.422	9.8E-06	56.6	15,777	0.797	3.960	7.5E-05	54.7	31,139
**ALL**																															
rs144655586	*CLSPN*	1	35725381	-	T	C	0.012	0.007	0.001	1.7E-07	12,658	-	-	-	-	-	-	-	-	-	-	0.011	4.873	1.1E-06	57.1	17,148	0.011	4.873	1.1E-06	57.1	17,148
rs712900	*LPPR4*	1	99261313	-	T	C	0.014	-0.006	0.001	3.4E-07	12,659	0.009	-3.293	9.9E-04	93.8	21,504	0.055	-2.096	3.6E-02	88.9	25,156	0.013	-4.298	1.7E-05	66	20,339	0.036	-1.375	1.7E-01	78	41,681
rs115546449	*TMEM63A*	1	225882871	-	A	G	0.026	-0.004	0.001	3.0E-07	12,659	0.017	-3.951	7.8E-05	90.6	21,503	0.074	-3.937	8.3E-05	73.9	25,156	0.024	-3.700	2.2E-04	67.5	22,450	0.048	-2.891	3.8E-03	59.2	43,791
rs145815581	*ANO10*	3	43466242	-	A	G	0.007	-0.008	0.002	1.8E-07	12,659	-	-	-	-	-	0.024	-3.273	1.1E-03	84.9	25,156	0.006	-4.157	3.2E-05	71.1	20,339	0.020	-2.892	3.8E-03	73.5	32,836
rs72886347	*FHIT*	3	60196461	-	T	C	0.993	0.008	0.002	7.2E-07	12,659	-	-	-	-	-	0.979	2.381	1.7E-02	89.8	25,156	0.993	3.221	1.3E-03	83.4	20,339	0.982	1.542	1.2E-01	83.6	32,836
rs17136358	*EIF2AK1*	7	6040031	-	T	C	0.963	-0.003	0.001	2.8E-07	12,659	0.953	-4.768	1.9E-06	81.2	21,503	0.953	-3.446	5.7E-04	80.6	25,156	0.964	-3.738	1.9E-04	57	25,001	0.954	-3.163	1.6E-03	53.1	46,342
rs117683919	*LOC105375440*	7	106634111	-	A	G	0.006	-0.009	0.002	1.4E-07	12,659	0.008	-4.053	5.1E-05	91.1	21,503	0.005	-4.957	7.2E-07	53.1	17,133	0.007	-2.750	6.0E-03	77.1	23,530	0.008	-2.508	1.2E-02	69.8	36,848
rs143542634	*PATL1*	11	59666070	-	A	G	0.006	0.009	0.002	4.3E-07	12,659	0.007	4.622	3.8E-06	82	21,504	0.005	4.873	1.1E-06	37.4	17,133	0.007	4.610	4.0E-06	54.7	16,200	0.007	4.450	8.6E-06	41.7	29,519

ƗAfrican American (AA) replication samples included : Atherosclerosis Risk in Communities(ARIC), Multi-Ethnic Study of Atherosclerosis(MESA), Women's Health Initiative(WHI)

‡Hispanic Latino (HL) replication samples included : Genetics of Latinos Diabetic Retinopathy(GOLDR), Hispanic Community Health Study / Study of Latinos(HCHS/SOL), Mexican–American Hypertension Study(HTN), Mexican-American Coronary Artery Disease(MACAD), Multi-Ethnic Study of Atherosclerosis(MESA), Mexico-City, 1982 Pelotas Birth Cohort(PELOTAS), Starr County Health Studies(STARR), Women's Health Initiative(WHI).

¥European American (EA) replication samples included : Atherosclerosis Risk in Communities(ARIC)

Supplementary Table 5. Summary of association results in SOL subgroup analyses for suggestively significant loci (P<1E-6) associated with WHRadjBMI. EAF-estimated allele frequency, CHR- chromosome, POS- position (build GRCh38), SE- standard error, ISQ- I squared heterogeneity. EAF for reference population obtained from 1000 Genomes Project Phase 3.

	Nearest		POS	Effect	Other	Combined					Caribbean					Mainland							EAF		
**dbSNPID**	**Gene**	**CHR**	**(GRCh38)**	**Allele**	**Allele**	**N**	**EAF**	**BETA**	**SE**	**P**	**N**	**EAF**	**BETA**	**SE**	**P**	**N**	**EAF**	**BETA**	**SE**	**P**	**ISQ**	**P_{diff}**	**AFR**	**EUR**	**AMR**
**MEN**																									
rs12032174	*RYR2*	1	237692868	C	T	5,200	0.352	-0.005	0.001	4.5E-07	2,395	0.353	-0.005	0.002	5.9E-04	2,793	0.352	-0.005	0.001	4.5E-04	64.72	3.8E-01	0.401	0.383	0.370
rs10475310	*PLEKHG4B*	5	23024	C	G	5,200	0.748	-0.006	0.001	**2.0E-08**	2,395	0.747	-0.006	0.002	1.8E-04	2,793	0.748	-0.006	0.002	4.9E-05	0	3.9E-01	0.781	0.751	0.733
rs16977373	*RIT2*	18	43189620	A	C	5,200	0.962	-0.013	0.003	6.6E-07	2,395	0.939	-0.015	0.003	9.7E-06	2,793	0.981	-0.010	0.005	3.9E-02	83.56	2.7E-01	0.752	0.997	0.984
rs721424	*CFAP61*	20	20358666	G	T	5,200	0.516	0.005	0.001	8.2E-07	2,395	0.520	0.005	0.002	2.4E-03	2,793	0.513	0.005	0.001	2.3E-04	0	4.0E-01	0.816	0.421	0.548
rs148213302	*STS*	X	7416464	T	C	5,189	0.995	0.025	0.005	3.7E-07	2,391	0.992	0.030	0.006	6.5E-07	2,786	0.997	0.009	0.009	3.0E-01	0	5.5E-02	0.981	1.000	1.000
**WOMEN**																									
rs77377042	*MARCKSL1*	1	32343334	C	T	7,472	0.976	-0.018	0.004	8.0E-07	3,238	0.961	-0.021	0.005	3.7E-06	4,220	0.987	-0.011	0.006	8.2E-02	81.79	1.7E-01	0.831	1.000	0.988
rs75120960	*EPHA5*	4	66367753	A	G	7,472	0.980	-0.020	0.004	1.9E-07	3,238	0.966	-0.018	0.005	1.6E-04	4,220	0.991	-0.027	0.008	3.9E-04	0	2.5E-01	0.860	1.000	0.987
rs16922424	*FAM110B*	8	57618850	T	C	7,471	0.982	0.021	0.004	4.9E-07	3,237	0.969	0.017	0.005	1.1E-03	4,220	0.992	0.034	0.008	2.6E-05	24.99	8.8E-02	0.868	1.000	0.987
rs79478137	*SLC22A18AS*	11	2891739	C	T	7,472	0.985	0.023	0.004	2.0E-07	3,238	0.975	0.025	0.006	8.9E-06	4,220	0.992	0.024	0.008	2.7E-03	0	4.0E-01	0.905	0.989	0.986
rs113818604	*NTM*	11	131960980	G	A	7,471	0.987	0.027	0.005	**5.5E-08**	3,238	0.987	0.014	0.008	8.1E-02	4,219	0.986	0.034	0.006	**1.6E-08**	78.48	6.1E-02	0.998	0.977	0.991
rs115981023	*TAOK3*	12	118313300	G	A	7,472	0.991	-0.029	0.006	8.9E-07	3,238	0.984	-0.030	0.007	2.7E-05	4,220	0.997	-0.027	0.012	2.5E-02	0	3.9E-01	0.937	0.998	0.999
rs146900844	*ZNF207*	17	32378136	G	A	7,472	0.995	0.041	0.008	6.3E-07	3,238	0.993	0.042	0.012	3.7E-04	4,220	0.996	0.040	0.012	6.6E-04	39.45	4.0E-01	1.000	0.994	0.996
rs61305557	*C19orf67*	19	14081134	G	A	7,472	0.971	0.018	0.004	4.8E-07	3,238	0.946	0.021	0.004	1.2E-06	4,220	0.990	0.005	0.008	4.8E-01	72.14	8.6E-02	0.818	0.999	0.978
**COMBINED**																									
rs13301996	*CDK5RAP2*	9	120570806	T	G	12,672	0.808	0.005	0.001	5.7E-07	5,633	0.789	0.006	0.002	2.5E-04	7,013	0.822	0.003	0.001	2.0E-02	37.02	1.8E-01	0.886	0.809	0.794
rs115981023	*TAOK3*	12	118313300	G	A	12,672	0.992	-0.023	0.004	2.1E-07	5,633	0.985	-0.019	0.006	8.9E-04	7,013	0.997	-0.030	0.010	2.6E-03	0	2.6E-01	0.937	0.998	0.999
rs185566196	*KIAA0391*	14	35152593	C	T	12,672	0.989	-0.019	0.004	2.6E-07	5,633	0.979	-0.025	0.005	5.3E-07	7,013	0.996	-0.019	0.009	3.6E-02	0	3.5E-01	0.956	0.993	0.996
rs116612483	*CDH4*	20	60751325	G	A	12,672	0.994	-0.027	0.005	6.0E-07	5,633	0.989	-0.025	0.007	2.6E-04	7,013	0.998	-0.011	0.016	5.0E-01	0	2.9E-01	0.950	1.000	0.999

Supplementary Table 6. Summary of association results in SOL subgroup analyses for suggestively significant loci (P<1E-6) associated with WCadjBMI. EAF-estimated allele frequency, CHR- chromosome, POS- position (build GRCh38), SE- standard error, ISQ- I squared heterogeneity. EAF for reference population obtained from 1000 Genomes Project Phase 3.

dbSNPID	Nearest Gene	CHR	POS (GRCh38)	Effect Allele	Other Allele	Combined					Caribbean					Mainland					ISQ	P_{diff}	EAF		
						N	EAF	BETA	SE	PVAL	N	EAF	BETA	SE	PVAL	N	EAF	BETA	SE	PVAL			AFR	EUR	AMR
**MEN**																									
rs74346221	*GABRD*	1	2029024	T	G	5,202	0.019	-2.023	0.41	8.1E-07	2,397	0.031	-1.811	0.490	2.2E-04	2,793	0.009	-2.565	0.779	9.9E-04	0	2.9E-01	0.121	0.000	0.009
rs72693785	*ATP1A1*	1	116334965	T	C	5,203	0.006	-4.088	0.734	**2.5E-08**	2,398	0.009	-4.557	0.858	1.1E-07	2,793	0.003	-3.336	1.516	2.8E-02	0	3.1E-01	0.002	0.009	0.001
rs11583298	*ESRRG*	1	217171261	T	C	5,203	0.822	-0.711	0.14	4.0E-07	2,398	0.910	-0.644	0.270	1.7E-02	2,793	0.747	-0.711	0.161	1.0E-05	0	3.9E-01	0.998	0.920	0.790
rs76842062	*MAP4K4*	2	101847192	T	G	5,203	0.995	3.799	0.72	1.3E-07	2,398	0.990	3.052	0.805	1.5E-04	2,793	0.998	6.881	1.688	4.6E-05	0	**4.9E-02**	0.962	0.999	0.996
rs141365360	*LOC102723448*	3	21826	A	G	5,203	0.007	3.154	0.629	5.3E-07	2,398	0.011	3.437	0.756	5.6E-06	2,793	0.004	2.159	1.153	6.1E-02	0	2.6E-01	0.056	0.000	0.004
rs6809759	*PROK2*	3	71937742	A	G	5,203	0.497	-0.586	0.102	**9.4E-09**	2,398	0.514	-0.461	0.155	2.9E-03	2,793	0.482	-0.673	0.135	6.5E-07	0	2.3E-01	0.358	0.601	0.504
rs76941364	*COBL*	7	52064843	A	G	5,203	0.989	2.443	0.498	9.4E-07	2,398	0.983	1.753	0.597	3.3E-03	2,793	0.995	4.303	0.922	3.0E-06	0	**2.7E-02**	0.950	0.999	0.994
rs139139519	*SULF1*	8	69625608	A	G	5,203	0.012	-2.365	0.458	2.4E-07	2,398	0.018	-2.668	0.573	3.2E-06	2,793	0.008	-1.747	0.775	2.4E-02	0	2.5E-01	0.018	0.011	0.009
rs35569658	*RBFOX1*	16	5754433	C	G	5,203	0.313	-0.562	0.112	5.3E-07	2,398	0.250	-0.560	0.180	1.8E-03	2,793	0.367	-0.536	0.142	1.6E-04	0	4.0E-01	0.138	0.249	0.353
rs148280037	*SHISA9*	16	12886370	T	C	5,203	0.984	2.081	0.412	4.5E-07	2,398	0.978	2.358	0.527	7.5E-06	2,793	0.989	1.638	0.674	1.5E-02	0	2.8E-01	0.979	0.987	0.994
rs143565319	*PIK3C3*	18	40962346	T	C	5,203	0.989	2.669	0.497	7.8E-08	2,398	0.983	2.656	0.583	5.3E-06	2,793	0.995	2.111	1.001	3.5E-02	0	3.6E-01	0.925	1.000	0.996
**WOMEN**																									
rs112469617	*FZD7*	2	201982887	T	C	7,471	0.009	-2.977	0.594	5.5E-07	3,237	0.014	-3.047	0.744	4.3E-05	4,220	0.006	-2.895	0.984	3.3E-03	0	4.0E-01	0.064	0.000	0.007
rs17385466	*SOX5*	12	23639004	T	C	7,471	0.923	1.089	0.209	1.8E-07	3,237	0.899	1.286	0.283	5.6E-06	4,220	0.941	0.869	0.308	4.8E-03	0	2.4E-01	0.995	0.886	0.916
**ALL**																									
rs77993329	*ACTRT2*	1	3001535	A	G	12,673	0.02	-1.457	0.288	4.1E-07	5,634	0.031	-1.610	0.371	1.4E-05	7,013	0.011	-1.523	0.528	3.9E-03	0	4.0E-01	0.091	0.000	0.019
rs138819965	*LINC00299*	2	8304328	A	G	12,674	0.994	2.706	0.525	2.5E-07	5,635	0.989	2.781	0.624	8.2E-06	7,013	0.998	1.670	1.230	1.7E-01	0	2.9E-01	0.953	1.000	0.999
rs77319470	*ADAMTS3*	4	72638360	T	G	12,674	0.371	0.406	0.082	8.6E-07	5,635	0.409	0.434	0.130	8.3E-04	7,013	0.341	0.329	0.115	4.1E-03	0	3.3E-01	0.580	0.391	0.359
rs921999	*FGF5*	4	80296998	A	C	12,674	0.965	-1.104	0.222	6.7E-07	5,635	0.939	-0.850	0.275	2.0E-03	7,013	0.986	-1.160	0.456	1.1E-02	0	3.4E-01	0.791	1.000	0.980
rs112404395	*AP3B1*	5	77964563	C	G	12,674	0.991	-2.22	0.412	7.1E-08	5,635	0.989	-2.344	0.602	9.9E-05	7,013	0.992	-2.164	0.627	5.6E-04	0	3.9E-01	0.999	0.979	0.993
rs77264633	*CCDC171*	9	15823920	A	G	12,674	0.962	-1.118	0.205	**4.6E-08**	5,635	0.955	-1.132	0.302	1.8E-04	7,013	0.967	-1.242	0.304	4.4E-05	0	3.9E-01	0.995	0.931	0.963
rs184067184	*PHF21A*	11	46189280	A	C	12,674	0.006	-3.612	0.702	2.7E-07	5,635	0.008	-3.988	0.833	1.7E-06	7,013	0.004	-2.871	1.720	9.5E-02	0	3.4E-01	0.000	0.000	0.004
rs3168072	*FADS2*	11	61864038	A	T	12,674	0.725	0.514	0.102	5.3E-07	5,635	0.885	0.417	0.207	4.4E-02	7,013	0.597	0.455	0.120	1.6E-04	0	3.9E-01	0.990	0.967	0.627
rs60260780	*WSB2*	12	118041899	T	C	12,674	0.013	1.78	0.35	3.7E-07	5,635	0.023	2.070	0.427	1.2E-06	7,013	0.005	0.620	0.759	4.1E-01	63.92	1.0E-01	0.117	0.001	0.006

Supplementary Table 7. Summary of association results in SOL subgroup analyses for suggestively significant loci (P<1E-6) associated with HIPadjBMI. EAF-estimated allele frequency, CHR- chromosome, POS-position (build GRCh38), SE- standard error, ISQ- I squared heterogeneity. EAF for reference population obtained from 1000 Genomes Project Phase 3.

dbSNPID	Nearest Gene	CHR	POS (GRCh38)	Effect Allele	Other Allele	Combined					Caribbean					Mainland					ISQ	P_{diff}	EAF		
						N	EAF	BETA	SE	PVAL	N	EAF	BETA	SE	PVAL	N	EAF	BETA	SE	PVAL			AFR	EUR	AMR
**MEN**																									
rs114865909	*NUF2*	1	163589134	T	C	5,197	0.994	-0.012	0.002	8.6E-07	2,394	0.989	-0.012	0.003	1.5E-05	2,791	0.998	-0.010	0.006	9.8E-02	7.27	3.7E-01	0.950	0.999	0.999
rs149681500	*ANO10*	3	43396572	T	C	5,197	0.007	-0.012	0.002	5.3E-08	2,394	0.013	-0.014	0.003	9.6E-08	2,791	0.002	-0.002	0.005	7.7E-01	80.06	**4.4E-02**	0.051	0.000	0.004
rs3915213	*LOC101927346*	3	74024720	T	C	5,197	0.779	-0.002	0.000	5.6E-07	2,394	0.712	-0.002	0.001	2.0E-04	2,791	0.836	-0.002	0.001	1.5E-03	0.00	3.8E-01	0.665	0.698	0.831
rs12677587	*LOC101929066*	8	18118812	C	G	5,197	0.012	-0.009	0.002	9.6E-07	2,394	0.020	-0.009	0.002	2.7E-05	2,791	0.005	-0.008	0.004	3.2E-02	0.00	3.7E-01	0.058	0.002	0.010
rs56405004	*MINPP1*	10	87512407	T	C	5,197	0.018	0.008	0.001	1.7E-07	2,394	0.027	0.007	0.002	5.1E-05	2,791	0.010	0.008	0.002	7.5E-04	0.00	3.9E-01	0.108	0.002	0.010
rs968849	*LOC102724589*	10	114997972	A	G	5,197	0.195	-0.002	0.000	8.2E-07	2,394	0.237	-0.002	0.001	1.2E-03	2,791	0.158	-0.002	0.001	4.0E-04	0.00	3.9E-01	0.161	0.313	0.154
rs76469489	*SLC7A10*	19	33249049	C	G	5,197	0.013	-0.009	0.002	9.6E-07	2,394	0.015	-0.009	0.002	2.7E-04	2,791	0.010	-0.008	0.003	1.5E-03	0.00	3.9E-01	0.002	0.035	0.016
rs7063750	*VCX*	X	7724974	T	G	5,186	0.039	0.004	0.001	2.7E-07	2,390	0.068	0.004	0.001	2.1E-05	2,784	0.014	0.004	0.002	4.6E-03	0.00	3.7E-01	0.628	0.086	0.120
rs112519383	*MID1*	X	10446834	A	G	5,186	0.988	-0.007	0.001	7.4E-08	2,390	0.980	-0.007	0.001	2.5E-06	2,784	0.995	-0.006	0.003	3.0E-02	0.00	3.5E-01	0.883	0.999	0.990
**WOMEN**																									
rs72978809	*LPPR4*	1	99260939	A	G	7,462	0.994	0.011	0.002	4.4E-07	3,232	0.990	0.012	0.003	1.4E-05	4,216	0.997	0.010	0.004	1.5E-02	0.00	3.8E-01	0.956	1.000	0.996
rs12478843	*HEATR5B*	2	37080089	A	G	7,462	0.248	-0.002	0.000	8.2E-08	3,232	0.154	-0.002	0.001	6.0E-03	4,216	0.320	-0.002	0.000	6.5E-06	1.68	3.9E-01	0.023	0.137	0.268
rs115331260	*LOC105376941*	3	5864170	A	G	7,462	0.985	0.008	0.002	8.8E-07	3,232	0.982	0.009	0.002	4.0E-05	4,216	0.987	0.005	0.002	1.9E-02	0.00	1.9E-01	0.003	0.037	0.004
rs7662640	*LOC105374566*	4	31765535	T	C	7,462	0.012	0.008	0.002	6.3E-07	3,232	0.012	0.009	0.003	5.8E-04	4,216	0.012	0.007	0.002	4.4E-04	0.00	3.7E-01	0.011	0.024	0.012
rs6814739	*LINC01094*	4	78654647	T	C	7,462	0.569	-0.002	0.000	7.2E-07	3,232	0.594	-0.002	0.001	2.8E-04	4,216	0.550	-0.002	0.000	1.7E-04	0.00	3.5E-01	0.154	0.766	0.566
rs11099588	*COQ2*	4	83280326	T	C	7,462	0.057	0.004	0.001	2.4E-07	3,232	0.090	0.005	0.001	1.9E-06	4,216	0.032	0.003	0.001	3.8E-02	64.14	2.0E-01	0.281	0.033	0.036
rs6860625	*NREP*	5	111667124	A	G	7,462	0.813	-0.002	0.000	6.4E-07	3,232	0.789	-0.004	0.001	1.3E-07	4,216	0.831	-0.001	0.001	6.4E-02	28.03	**9.5E-03**	0.593	0.869	0.839
rs77186623	*LOC105375745*	8	125608952	A	C	7,461	0.022	-0.006	0.001	1.7E-07	3,231	0.041	-0.008	0.001	**3.1E-08**	4,216	0.008	-0.001	0.003	5.7E-01	55.31	**4.2E-02**	0.115	0.000	0.017
rs10818474	*MEGF9*	9	120727686	T	C	7,462	0.809	-0.002	0.000	5.1E-07	3,232	0.743	-0.002	0.001	3.1E-03	4,216	0.860	-0.003	0.001	4.2E-06	0.00	1.6E-01	0.548	0.764	0.817
rs28692724*	*IRF2BPL*	14	77027445	T	C	7,462	0.425	0.002	0.000	7.3E-07	3,232	0.377	0.002	0.001	4.0E-05	4,216	0.462	0.002	0.001	8.7E-04	0.00	2.3E-01	0.160	0.384	0.310
rs6092086	*LOC105372676*	20	55193568	A	G	7,462	0.922	-0.003	0.001	3.4E-07	3,232	0.871	-0.003	0.001	4.0E-05	4,216	0.962	-0.003	0.001	6.4E-03	0.00	4.0E-01	0.562	0.989	0.944
rs9631175	*TAF4*	20	62072648	A	C	7,462	0.793	0.002	0.000	6.0E-08	3,232	0.834	0.002	0.001	1.3E-02	4,216	0.762	0.003	0.001	2.1E-06	38.00	2.8E-01	0.701	0.888	0.785
**ALL**																									
rs144655586	*CLSPN*	1	35725381	T	C	12,658	0.012	0.007	0.001	1.7E-07	5,625	0.012	0.010	0.002	9.1E-06	7,007	0.011	0.006	0.002	3.5E-03	49.55	1.5E-01	0.001	0.014	0.006
rs712900	*LPPR4*	1	99261313	T	C	12,659	0.014	-0.006	0.001	3.4E-07	5,626	0.022	-0.005	0.001	1.3E-04	7,007	0.007	-0.006	0.002	3.2E-03	0.00	3.8E-01	0.098	0.001	0.007
rs115546449	*TMEM63A*	1	225882871	A	G	12,659	0.026	-0.004	0.001	3.0E-07	5,626	0.047	-0.004	0.001	1.4E-04	7,007	0.010	-0.005	0.002	4.0E-03	0.00	3.2E-01	0.136	0.001	0.014
rs145815581	*ANO10*	3	43466242	A	G	12,659	0.007	-0.008	0.002	1.8E-07	5,626	0.013	-0.009	0.002	2.3E-07	7,007	0.003	-0.002	0.003	5.9E-01	76.32	**4.8E-02**	0.051	0.000	0.004
rs72886347	*FHIT*	3	60196461	T	C	12,659	0.993	0.008	0.002	7.2E-07	5,626	0.989	0.010	0.002	4.5E-07	7,007	0.997	0.006	0.003	6.0E-02	15.13	2.2E-01	0.940	1.000	0.994
rs17136358	*EIF2AK1*	7	6040031	T	C	12,659	0.963	-0.003	0.001	2.8E-07	5,626	0.951	-0.003	0.001	2.6E-03	7,007	0.972	-0.004	0.001	4.9E-04	0.00	3.4E-01	0.958	0.953	0.967
rs117683919	*LOC105375440*	7	106634111	A	G	12,659	0.006	-0.009	0.002	1.4E-07	5,626	0.009	-0.011	0.002	4.0E-07	7,007	0.004	-0.007	0.003	1.5E-02	0.00	1.9E-01	0.005	0.014	0.003
rs143542634	*PATL1*	11	59666070	A	G	12,659	0.006	0.009	0.002	4.3E-07	5,626	0.006	0.011	0.003	6.7E-05	7,007	0.007	0.008	0.002	2.1E-04	0.00	2.9E-01	0.001	0.006	0.004

Supplementary Table 8. Local ancestry results for all suggestively significant loci across all traits.

rsID	Nearest Gene	CHR	POS (GRCh38)	Sex	Effect Allele	Other Allele	EAF		
							African	Native American	European
**WHRadjBMI**									
rs13301996	*CDK5RAP2*	9	120570806	All	T	G	0.9029	0.8890	0.7414
rs115981023	*TAOK3*	12	118313300	All	G	A	0.9431	1.0000	0.9996
rs185566196	*KIAA0391*	14	35152593	All	NA	NA	NA	NA	NA
rs116612483	*CDH4*	20	60751325	All	G	A	0.9631	1.0000	0.9999
rs12032174	*RYR2*	1	237692868	Men	C	T	0.4060	0.3051	0.3693
rs10475310	*PLEKHG4B*	5	23024	Men	NA	NA	NA	NA	NA
rs16977373	*RIT2*	18	43189620	Men	A	C	0.7718	1.0000	0.9911
rs721424	*CFAP61*	20	20358666	Men	G	T	0.8124	0.5079	0.4454
rs148213302	*STS*	23	7416464	Men	NA	NA	NA	NA	NA
rs77377042	*MARCKSL1*	1	32343334	Women	C	T	0.8421	1.0000	0.9994
rs75120960	*EPHA5*	4	66367753	Women	A	G	0.8609	1.0000	0.9994
rs16922424	*FAM110B*	8	57618850	Women	T	C	0.8762	1.0000	0.9994
rs79478137	*SLC22A18AS*	11	2891739	Women	C	T	0.9232	0.9989	0.9946
rs113818604	*NTM*	11	131960980	Women	G	A	1.0000	1.0000	0.9809
rs115981023	*TAOK3*	12	118313300	Women	G	A	0.9431	1.0000	0.9996
rs146900844	*ZNF207*	17	32378136	Women	G	A	1.0000	1.0000	0.9942
rs61305557	*C19orf67*	19	14081134	Women	NA	NA	NA	NA	NA
**WCadjBMI**									
rs77993329	*ACTRT2*	1	3001535	All	G	A	0.9126	0.9996	0.9904
rs138819965	*LINC00299*	2	8304328	All	A	G	0.9598	1.0000	1.0000
rs77319470	*ADAMTS3*	4	72638360	All	G	T	0.5050	0.7252	0.6110
rs921999	*FGF5*	4	80296998	All	A	C	0.7737	0.9998	0.9941
rs112404395	*AP3B1*	5	77964563	All	C	G	1.0000	1.0000	0.9835
rs77264633	*CCDC171*	9	15823920	All	A	G	1.0000	0.9985	0.9318
rs184067184	*PHF21A*	11	46189280	All	C	A	1.0000	0.9946	1.0000
rs3168072	*FADS2*	11	61864038	All	A	T	0.9884	0.1949	0.9755
rs60260780	*WSB2*	12	118041899	All	C	T	0.9112	0.9999	0.9998
rs74346221	*GABRD*	1	2029024	Men	NA	NA	NA	NA	NA
rs72693785	*ATP1A1*	1	116334965	Men	C	T	1.0000	1.0000	0.9943
rs11583298	*ESRRG*	1	217171261	Men	T	C	1.0000	0.5320	0.9309
rs76842062	*MAP4K4*	2	101847192	Men	T	G	0.9775	1.0000	0.9997
rs141365360	*LOC102723448*	3	21826	Men	G	A	0.9512	0.9999	0.9996
rs6809759	*PROK2*	3	71937742	Men	G	A	0.6679	0.6342	0.3908
rs76941364	*COBL*	7	52064843	Men	A	G	0.9472	1.0000	0.9971
rs139139519	*SULF1*	8	69625608	Men	G	A	0.9803	1.0000	0.9820
rs35569658	*RBFOX1*	16	5754433	Men	G	C	0.8982	0.5073	0.7564
rs148280037	*SHISA9*	16	12886370	Men	T	C	0.9780	0.9997	0.9825
rs143565319	*PIK3C3*	18	40962346	Men	T	C	0.9216	1.0000	0.9998
rs112469617	*FZD7*	2	201982887	Women	C	T	0.9471	1.0000	1.0000
rs17385466	*SOX5*	12	23639004	Women	T	C	0.9990	0.9998	0.8576
**HIPadjBMI**									
rs144655586	*CLSPN*	1	35725381	All	C	T	0.9976	1.0000	0.9914
rs712900	*LPPR4*	1	99261313	All	C	T	0.8977	1.0000	0.9998
rs115546449	*TMEM63A*	1	225882871	All	G	A	0.8206	1.0000	0.9983
rs145815581	*ANO10*	3	43466242	All	G	A	0.9531	1.0000	1.0000

rs72886347	*FHIT*	3	60184786	All	T	C	0.9608	1.0000	0.9998
rs17136358	*EIF2AK1*	7	6040031	All	T	C	0.9423	0.9995	0.9486
rs117683919	*LOC105375440*	7	106634111	All	G	A	0.9944	1.0000	0.9909
rs143542634	*PATL1*	11	59666070	All	G	A	1.0000	1.0000	0.9905
rs114865909	*NUF2*	1	163589134	Men	T	C	0.9550	1.0000	0.9996
rs149681500	*ANO10*	3	43396572	Men	C	T	0.9518	1.0000	1.0000
rs3915213	*LOC101927346*	3	74024720	Men	T	C	0.6733	0.9898	0.6870
rs12677587	*LOC101929066*	8	18118812	Men	G	C	0.9245	0.9997	0.9972
rs56405004	*MINPP1*	10	87512407	Men	C	T	0.8830	0.9997	0.9953
rs968849	*LOC102724589*	10	114997972	Men	G	A	0.8553	0.9888	0.6894
rs76469489	*SLC7A10*	19	33249049	Men	G	C	1.0000	0.9991	0.9775
rs7063750	*VCX*	23	7724974	Men	NA	NA	NA	NA	NA
rs112519383	*MID1*	23	10446834	Men	NA	NA	NA	NA	NA
rs72978809	*LPPR4*	1	99260939	Women	A	G	0.9607	1.0000	0.9998
rs12478843	*HEATR5B*	2	37080089	Women	G	A	0.9869	0.4895	0.8482
rs115331260	*LOC105376941*	3	5864170	Women	A	G	1.0000	1.0000	0.9843
rs7662640	*LOC105374566*	4	31765535	Women	C	T	0.9930	0.9924	0.9864
rs6814739	*LINC01094*	4	78654647	Women	C	T	0.8707	0.6437	0.1988
rs11099588	*COQ2*	4	83280326	Women	T	C	0.2944	0.0006	0.0308
rs6860625	*NREP*	5	111667124	Women	A	G	0.5807	0.8582	0.8526
rs77186623	*LOC105375745*	8	125608952	Women	C	A	0.8587	1.0000	0.9965
rs10818474	*MEGF9*	9	120727686	Women	C	T	0.4459	0.0034	0.2387
rs28692724	*IRF2BPL*	14	77027445	Women	C	T	0.8296	0.4506	0.6436
rs6092086	*LOC105372676*	20	55193568	Women	A	G	0.5493	0.9995	0.9692
rs9631175	*TAF4*	20	62072648	Women	A	C	0.7289	0.6444	0.8909

Supplementary Table 9. Top variants for each generalized locus for WHRadjBMI in SOL across known loci found in GIANT GWAS

						GIANT estimates				HCHS/SOL estimates					Meta-Analysis estimates			
dbSNPID	Region	Chr	Position (build GRCh38)	Effect Allele	Other Allele	Effect Allele Frequency	Beta	SE	P-value	Effect Allele Frequency	Beta	SE	P-value	Generalization r-value	Beta	SE	P-value	Locus #
**WOMEN**																		
rs7543720	*LOC101929147*	1	119163343	A	G	0.325	-0.031	0.006	1.00E-07	0.239	-0.077	0.019	5.98E-05	0.035	-0.035	0.006	3.48E-10	**1**
rs2605082	*LOC102723886*	1	219446196	G	A	0.305	0.031	0.005	4.90E-11	0.291	0.081	0.018	5.97E-06	0.007	0.034	0.005	5.08E-14	**2**
rs3769869	*COBLL1*	2	164686689	A	G	0.783	0.051	0.006	1.80E-20	0.769	0.072	0.019	1.71E-04	0.038	0.053	0.005	2.56E-23	**3**
rs9311910	*ADAMTS9-AS2*	3	64721593	G	A	0.550	0.043	0.005	6.30E-21	0.417	0.051	0.017	2.42E-03	0.047	0.044	0.004	9.49E-23	**4**
rs1294410	*LOC101928004*	6	6738519	C	T	0.625	0.037	0.005	6.00E-16	0.529	0.052	0.017	1.66E-03	0.038	0.038	0.004	8.58E-18	**5**
rs2800703	*LOC105377989*	6	127105653	G	T	0.333	0.048	0.005	2.20E-22	0.419	0.054	0.017	1.36E-03	0.038	0.048	0.005	6.99E-25	**6**
**ALL**																		
rs1106529	*TBX15*	1	118988874	G	A	0.275	-0.035	0.004	1.80E-19	0.315	-0.066	0.014	1.59E-06	0.002	-0.037	0.004	2.64E-23	**1**
rs2605095	*LOC107985272*	1	219468108	T	A	0.317	-0.039	0.005	8.00E-18	0.442	-0.038	0.013	2.47E-03	0.027	-0.039	0.004	4.31E-20	**2**
rs40271	*intergenic*	5	56500492	C	T	0.225	0.022	0.004	3.80E-09	0.362	0.038	0.013	3.51E-03	0.034	0.023	0.004	1.86E-10	**7**
rs1294410	*LOC101928004*	6	6738519	C	T	0.625	0.032	0.004	7.70E-20	0.524	0.048	0.013	1.67E-04	0.010	0.033	0.003	9.69E-23	**5**
rs7192	*HLA-DRA*	6	32443869	G	T	0.583	0.017	0.004	5.40E-07	0.670	0.038	0.013	4.62E-03	0.041	0.018	0.003	6.04E-08	**8**
rs1358980	*LOC105375070*	6	43796814	T	C	0.450	0.038	0.004	1.30E-26	0.480	0.049	0.013	8.84E-05	0.008	0.039	0.003	1.15E-30	**9**
rs11766345	*LAMB1*	7	107971205	G	T	0.875	0.037	0.007	7.10E-07	0.871	0.056	0.019	3.05E-03	0.031	0.040	0.007	9.64E-09	**10**
rs894737	*HOXC4*	12	54024359	C	A	0.342	0.029	0.004	2.40E-11	0.323	0.048	0.013	3.44E-04	0.012	0.031	0.004	1.62E-13	**11**
rs4930723	*CCDC92*	12	123939053	G	C	0.617	0.030	0.005	1.70E-11	0.702	0.048	0.014	4.74E-04	0.015	0.032	0.004	1.16E-13	**12**

Abbreviations: Chr, chromosome; SE, standard error

Supplementary Table 10. Top variants for each generalized locus for WCadjBMI in SOL across known loci found in GIANT GWAS

						GIANT estimates				HCHS/SOL estimates					Meta-Analysis estimates			
dbSNPID	Region	Chr	Position (build GRCh38)	Effect Allele	Other Allele	Effect Allele Frequency	Beta	SE	P-value	Effect Allele Frequency	Beta	SE	P-value	Generalization r-value	Beta	SE	P-value	Locus #
**MEN**																		
rs1409156	*TBX15*	1	118750684	G	A	0.342	0.026	0.005	1.30E-07	0.325	0.076	0.021	3.95E-04	0.034	0.028	0.005	2.50E-09	1
rs3791679	*EFEMP1*	2	55723261	A	G	0.725	0.053	0.006	6.20E-22	0.796	0.090	0.024	2.40E-04	0.034	0.055	0.005	1.83E-24	2
rs1147225	*NPR3*	5	32823835	C	T	0.642	0.027	0.005	1.50E-08	0.576	0.060	0.020	2.43E-03	0.050	0.029	0.005	6.48E-10	3
rs459193	*C5orf67*	5	56546681	A	G	0.217	0.027	0.005	4.50E-07	0.277	0.078	0.022	3.88E-04	0.034	0.030	0.005	7.28E-09	4
rs9358927	*LOC10192874*	6	26438243	T	G	0.583	0.027	0.005	1.50E-08	0.591	0.067	0.020	7.79E-04	0.034	0.029	0.005	4.05E-10	5
rs754133	*HOXC4*	12	52311403	A	G	0.333	0.032	0.005	8.80E-11	0.326	0.067	0.021	1.36E-03	0.037	0.034	0.005	1.33E-12	6
**WOMEN**																		
rs6691985	*CROCC*	1	16963416	T	C	0.225	-0.024	0.005	2.80E-07	0.339	-0.074	0.018	3.54E-05	0.025	-0.027	0.005	2.15E-09	7
rs1106529	*TBX15*	1	118790397	G	A	0.275	-0.039	0.005	1.00E-14	0.312	-0.073	0.018	4.11E-05	0.025	-0.041	0.005	6.62E-18	1
rs2605082	*LOC10272388*	1	217512819	G	A	0.305	0.023	0.005	7.00E-07	0.292	0.070	0.018	8.32E-05	0.027	0.026	0.004	5.78E-09	8
rs3769869	*COBLL1*	2	164394935	A	G	0.783	0.035	0.005	2.90E-11	0.769	0.063	0.019	9.55E-04	0.027	0.037	0.005	4.34E-13	9
rs10049090	*LINC02029*	3	158562607	G	A	0.600	0.029	0.006	1.00E-07	0.531	0.049	0.017	3.20E-03	0.027	0.031	0.005	3.00E-09	10
rs7705502	*CPEB4*	5	173826418	A	G	0.292	0.029	0.005	4.40E-10	0.167	0.064	0.022	3.46E-03	0.027	0.030	0.005	1.29E-11	11
rs10748827	*SUFU*	10	102598569	G	T	0.700	0.022	0.004	5.80E-07	0.652	0.050	0.017	3.55E-03	0.027	0.024	0.004	2.57E-08	12
rs11853983	*ADAMTSL3*	15	81961473	A	G	0.725	0.031	0.006	2.30E-08	0.782	0.064	0.020	1.28E-03	0.027	0.033	0.005	5.60E-10	13
rs757608	Intergenic	17	58774698	A	G	0.300	0.026	0.005	1.40E-08	0.364	0.060	0.017	4.20E-04	0.027	0.028	0.004	1.83E-10	14
**ALL**																		
rs6691985	*CROCC*	1	16963416	T	C	0.225	-0.026	0.004	3.90E-13	0.337	-0.060	0.014	1.71E-05	0.008	-0.028	0.003	6.92E-16	7
rs1106529	*TBX15*	1	118790397	G	A	0.275	-0.035	0.004	1.00E-20	0.315	-0.066	0.014	1.58E-06	0.002	-0.037	0.004	3.08E-24	1
rs17369648	Intergenic	2	55708944	C	T	0.7	0.029	0.005	4.20E-09	0.788	0.048	0.016	1.97E-03	0.042	0.031	0.005	4.91E-11	2
rs10049090	*LINC02029*	3	158562607	G	A	0.6	0.030	0.005	2.60E-11	0.532	0.043	0.013	8.81E-04	0.033	0.031	0.004	1.46E-13	10
rs7697556	Intergenic	4	72868460	C	T	0.4917	-0.016	0.003	9.00E-07	0.557	-0.057	0.013	8.30E-06	0.026	-0.019	0.003	6.08E-09	15
rs455660	*C5orf67*	5	56556818	T	C	0.1333	0.024	0.004	3.70E-09	0.202	0.049	0.016	2.46E-03	0.046	0.026	0.004	3.07E-10	4
rs754133	*HOXC4*	12	52311403	A	G	0.3333	0.029	0.004	4.00E-17	0.322	0.040	0.013	2.84E-03	0.048	0.030	0.003	1.79E-18	6
rs7214743	Intergenic	17	58775473	A	G	0.2917	0.024	0.005	1.30E-07	0.363	0.051	0.013	1.12E-04	0.022	0.027	0.004	5.79E-10	14

Abbreviations: Chr, chromosome; SE, standard error

Supplementary Table 11. Top variants for each generalized locus for hip ratio adjusted for body mass index in SOL across known loci found in GIANT GWAS

						GIANT estimates				HCHS/SOL estimates					Meta-Analysis estimates			
dbSNPID	Region	Chr	Position (build GRCh38)	Effect Allele	Other Allele	Effect Allele Frequency	Beta	SE	P-value	Effect Allele Frequency	Beta	SE	P-value	Generalization r-value	Beta	SE	P-value	Locus #
**WOMEN**																		
rs2820443	Regulatory Region	1	217646790	C	T	0.300	0.062	0.005	4.30E-35	0.402	0.060	0.017	3.175E-04	0.047	0.062	0.005	3.88E-38	1
rs1346786	*EFEMP1*	2	55734702	C	T	0.667	0.030	0.006	2.60E-07	0.691	0.081	0.018	6.000E-06	0.047	0.035	0.006	4.12E-10	2
rs17819328	*PPARG*	3	12422843	G	T	0.450	-0.028	0.005	1.60E-09	0.375	-0.062	0.017	3.839E-04	0.047	-0.030	0.005	2.44E-11	3
**ALL**																		
rs761422	*MFAP2*	1	16975285	A	G	0.440	-0.028	0.005	1.40E-08	0.523	-0.037	0.013	4.161E-03	0.040	-0.029	0.005	3.90E-10	4
rs11205303	*MTMR11*	1	149934520	C	T	0.358	0.042	0.004	6.40E-26	0.271	0.042	0.014	2.792E-03	0.037	0.042	0.004	9.52E-28	5
rs2494196	Intergenic	1	217655862	A	C	0.250	0.051	0.005	7.60E-23	0.422	0.049	0.013	1.533E-04	0.018	0.051	0.005	1.12E-26	1
rs848607	*CRIM1*	2	36396553	A	G	0.367	-0.018	0.004	9.60E-07	0.455	-0.043	0.013	7.675E-04	0.021	-0.020	0.004	1.99E-08	6
rs1346786	*EFEMP1*	2	55734702	C	T	0.667	0.030	0.005	5.40E-10	0.690	0.063	0.014	4.500E-06	0.013	0.034	0.005	1.24E-13	2
rs9872031	*PPARG*	3	12429962	A	G	0.442	-0.024	0.005	1.70E-07	0.377	-0.047	0.013	3.930E-04	0.018	-0.026	0.004	1.14E-09	3
rs2811469	*LXND1*	3	131045304	G	A	0.708	-0.037	0.006	5.30E-11	0.788	-0.057	0.016	2.904E-04	0.018	-0.039	0.005	2.15E-13	7
rs11936911	*LCORL*	4	17556143	G	A	0.158	-0.036	0.006	1.40E-08	0.209	-0.049	0.016	1.688E-03	0.032	-0.038	0.006	9.57E-11	8
rs7670141		4	72863854	A	G	0.517	-0.026	0.005	7.10E-09	0.604	-0.056	0.013	1.740E-05	0.015	-0.029	0.004	6.67E-12	9
rs1443537		4	81448750	A	C	0.242	0.025	0.004	4.70E-10	0.332	0.042	0.013	1.539E-03	0.030	0.026	0.004	5.37E-12	10
rs3775380	*FAM13A*	4	89037680	G	A	0.492	-0.018	0.004	2.50E-07	0.491	-0.057	0.013	6.970E-06	0.018	-0.021	0.003	7.72E-10	11
rs2035742	*HHIP*	4	144875668	A	G	0.958	0.044	0.009	3.40E-07	0.957	0.115	0.031	2.055E-04	0.018	0.049	0.008	4.32E-09	12
rs1147225	*NPR3*	5	32823835	C	T	0.642	0.018	0.004	3.50E-07	0.577	0.037	0.013	4.326E-03	0.041	0.019	0.003	1.13E-08	13
rs1294438	*LOC101928004*	6	6696825	C	T	0.667	-0.027	0.004	4.70E-12	0.512	-0.044	0.013	5.751E-04	0.018	-0.028	0.004	2.37E-14	14
rs4371882	*BMP6*	6	7737935	A	G	0.833	-0.027	0.005	3.30E-09	0.809	-0.055	0.016	6.764E-04	0.020	-0.029	0.004	2.20E-11	15
rs1265083	*CCHCR1*	6	31251549	T	G	0.008	-0.073	0.015	9.20E-07	0.042	-0.144	0.032	5.100E-06	0.018	-0.086	0.014	2.13E-10	16
rs4140531	*ADGRG6*	6	142405651	G	A	0.933	0.040	0.008	3.50E-07	0.910	0.065	0.022	3.673E-03	0.040	0.043	0.007	6.63E-09	17
rs7783400	*GNA12*	7	2821326	A	C	0.275	-0.033	0.005	8.30E-12	0.280	-0.041	0.014	3.479E-03	0.040	-0.034	0.005	9.34E-14	18
rs42377	*CDK6*	7	92452294	A	G	0.371	0.037	0.006	1.40E-11	0.297	0.038	0.014	6.272E-03	0.050	0.037	0.005	3.84E-13	19
rs10990768	*SLC35D2*	9	95413062	C	T	0.858	-0.031	0.006	5.50E-07	0.924	-0.096	0.024	5.700E-05	0.018	-0.035	0.006	4.84E-09	20
rs7082470	*JMJD1C*	10	63187272	G	A	0.517	-0.018	0.004	4.90E-07	0.679	-0.043	0.014	1.742E-03	0.032	-0.020	0.003	8.66E-09	21
rs10882717	*ENTPD1-AS1*	10	96081177	G	C	0.692	0.023	0.005	6.10E-07	0.699	0.041	0.014	3.133E-03	0.038	0.025	0.004	1.38E-08	22
rs2701540	*MACROD1*	11	63858211	T	A	0.042	-0.048	0.010	9.90E-07	0.086	-0.073	0.023	1.169E-03	0.028	-0.052	0.009	7.24E-09	23
rs2160077	Regulatory Region	14	91031819	G	A	0.608	0.017	0.004	8.90E-07	0.677	0.038	0.014	4.611E-03	0.042	0.018	0.003	6.26E-08	24
rs8029016	*ADAMTSL3*	15	82085127	C	T	0.475	0.036	0.003	1.80E-25	0.664	0.052	0.014	2.424E-04	0.018	0.037	0.003	7.14E-29	25
rs2281727	*SMG6*	17	2161401	G	A	0.333	-0.018	0.004	5.30E-07	0.361	-0.057	0.013	1.830E-05	0.018	-0.021	0.003	2.78E-09	26
rs143499	*SMG6*	17	2175780	T	C	0.325	-0.018	0.004	9.10E-07	0.365	-0.057	0.013	1.750E-05	0.018	-0.021	0.003	2.75E-09	27
rs4800451	*CABLES1*	18	21390842	T	C	0.683	0.030	0.004	9.70E-14	0.634	0.043	0.013	1.178E-03	0.028	0.031	0.004	1.88E-15	28
rs158676	*CDK5RAP1*	20	32850250	G	A	0.283	-0.022	0.004	2.10E-09	0.246	-0.042	0.015	4.904E-03	0.044	-0.023	0.003	4.04E-11	29
rs2425060	*UQCC1*	20	34790312	T	C	0.675	-0.034	0.005	5.50E-13	0.710	-0.042	0.014	2.659E-03	0.037	-0.035	0.004	5.54E-15	30

Abbreviations: Chr, chromosome; SE, standard error

Supplementary Table 12. P-values from testing genetic scores for each of the traits and populations

Strata	p-value<1e-8	1e-8<p-value<1e-7	1e-7<p-value<1e-6
**WHRadjBMI**			
MEN	9.00E-03	4.55E-02	3.02E-01
WOMEN	5.95E-06	3.85E-03	8.36E-03
ALL	2.00E-06	5.78E-03	2.87E-01
**WCadjBMI**			
MEN	1.17E-02	8.20E-05	4.33E-06
WOMEN	2.74E-04	5.46E-01	1.62E-02
ALL	9.41E-12	1.24E-03	9.68E-03
**HIPadjBMI**			
MEN	1.46E-10	2.14E-03	9.17E-02
WOMEN	1.89E-11	2.36E-05	2.95E-02
ALL	3.81E-07	6.46E-03	4.45E-05

Supplementary Table 13. GWAS lookups of replication variants from current study in Phenoscanner. Only significant SNP-phenotype associations shown ($P<0.05/7631=6.55x10^{-5}$).

Discovery Trait	dbSNPID	CHR	POS (GRCh38)	Effect Allele	Other Allele	GWAS Trait	PMID/ Source	Ancestry	Beta	SE	P	N	N (cases)	N (controls)
WHRadjBMI	rs79478137	11	2891739	C	T	Cause of death: multisystem degeneration	UKBB	European	-0.024	0.004	**4.8E-11**	7637	8	7629
WHRadjBMI	rs79478137	11	2891739	C	T	Cause of death: tongue, unspecified	UKBB	European	-0.023	0.004	**4.8E-08**	7637	11	7626
WHRadjBMI	rs79478137	11	2891739	C	T	Home area population density: postcode not linkable	UKBB	European	-0.001	0.000	**1.4E-07**	333997	12	333985
WCadjBMI	rs3168072	11	61864038	A	T	Cause of death: other specified respiratory disorders	UKBB	European	-0.006	0.001	**1.7E-06**	7637	4	7633

Supplementary Table 14. eQTL lookups of replicated variants from current study in Phenoscanner. Only significant SNP-Gene expression associations shown ($P<0.05/88=5.68x10^{-4}$).

Discovery Trait	dbSNPID	CHR	POS (GRCh38)	Nearest Gene	Effect Allele	Other Allele	PMID/ Source	Ancestry	Tissue	Gene	Beta	SE	P	N	Dataset
WHRadjBMI	rs13301996	9	120570806	*CDK5RAP2*	G	T	25954001	Unspecified	Adipose subcutaneous	*MEGF9*	0.293	0.055	**1.9E-07**	385	GTEx-V7_eQTL_EUR_2017
WHRadjBMI	rs13301996	9	120570806	*CDK5RAP2*	G	T	25954001	Unspecified	Adipose subcutaneous	*AHCYP2*	0.351	0.084	**4.1E-05**	385	GTEx-V7_eQTL_EUR_2017
WHRadjBMI	rs13301996	9	120570806	*CDK5RAP2*	G	T	25954001	Unspecified	Adipose subcutaneous	*MEGF9*	0.254	0.067	**1.7E-04**	298	GTEx-V6p_eQTL_EUR_2016
WHRadjBMI	rs13301996	9	120570806	*CDK5RAP2*	G	T	25954001	Unspecified	Adipose subcutaneous	*PSMD5-AS1*	0.289	0.082	**5.0E-04**	385	GTEx-V7_eQTL_EUR_2017
WHRadjBMI	rs13301996	9	120570806	*CDK5RAP2*	G	T	25954001	Unspecified	Artery aorta	*PSMD5-AS1*	0.363	0.103	**5.1E-04**	267	GTEx-V7_eQTL_EUR_2017
WHRadjBMI	rs13301996	9	120570806	*CDK5RAP2*	G	T	25954001	Unspecified	Artery coronary	-	0.593	0.150	**1.5E-04**	118	GTEx-V6p_eQTL_EUR_2016
WHRadjBMI	rs13301996	9	120570806	*CDK5RAP2*	G	T	25954001	Unspecified	Artery tibial	*PSMD5-AS1*	0.458	0.087	**2.5E-07**	388	GTEx-V7_eQTL_EUR_2017
WHRadjBMI	rs13301996	9	120570806	*CDK5RAP2*	G	T	25954001	Unspecified	Artery tibial	*PSMD5-AS1*	0.436	0.102	**3.0E-05**	285	GTEx-V6p_eQTL_EUR_2016
WHRadjBMI	rs13301996	9	120570806	*CDK5RAP2*	G	T	25954001	Unspecified	Brain cerebellum	*PSMD5-AS1*	0.564	0.152	**3.9E-04**	103	GTEx-V6p_eQTL_EUR_2016
WHRadjBMI	rs13301996	9	120570806	*CDK5RAP2*	G	T	25954001	Unspecified	Colon sigmoid	*PSMD5-AS1*	0.404	0.110	**3.1E-04**	203	GTEx-V7_eQTL_EUR_2017
WHRadjBMI	rs13301996	9	120570806	*CDK5RAP2*	G	T	25954001	Unspecified	Colon sigmoid	*PSMD5-AS1*	0.511	0.142	**5.1E-04**	124	GTEx-V6p_eQTL_EUR_2016
WHRadjBMI	rs13301996	9	120570806	*CDK5RAP2*	G	T	25954001	Unspecified	Colon sigmoid	*AHCYP2*	0.346	0.098	**5.5E-04**	203	GTEx-V7_eQTL_EUR_2017
WHRadjBMI	rs13301996	9	120570806	*CDK5RAP2*	G	T	25954001	Unspecified	Esophagus muscularis	*PSMD5-AS1*	0.407	0.091	**1.2E-05**	335	GTEx-V7_eQTL_EUR_2017
WHRadjBMI	rs13301996	9	120570806	*CDK5RAP2*	G	T	25954001	Unspecified	Esophagus muscularis	*PSMD5-AS1*	0.451	0.112	**7.8E-05**	218	GTEx-V6p_eQTL_EUR_2016
WHRadjBMI	rs13301996	9	120570806	*CDK5RAP2*	G	T	25954001	Unspecified	Esophagus muscularis	*AHCYP2*	0.309	0.088	**5.3E-04**	335	GTEx-V7_eQTL_EUR_2017
WHRadjBMI	rs13301996	9	120570806	*CDK5RAP2*	G	T	25954001	Unspecified	Liver	*PSMD5*	0.354	0.093	**2.2E-04**	153	GTEx-V7_eQTL_EUR_2017
WHRadjBMI	rs13301996	9	120570806	*CDK5RAP2*	G	T	25954001	Unspecified	Liver	*PSMD5-AS1*	0.361	0.100	**4.6E-04**	153	GTEx-V7_eQTL_EUR_2017
WHRadjBMI	rs13301996	9	120570806	*CDK5RAP2*	G	T	25954001	Unspecified	Lung	*PSMD5-AS1*	0.349	0.076	**6.7E-06**	383	GTEx-V7_eQTL_EUR_2017
WHRadjBMI	rs13301996	9	120570806	*CDK5RAP2*	G	T	25954001	Unspecified	Muscle skeletal	*PSMD5*	0.261	0.056	**4.1E-06**	491	GTEx-V7_eQTL_EUR_2017
WHRadjBMI	rs13301996	9	120570806	*CDK5RAP2*	G	T	25954001	Unspecified	Muscle skeletal	*PSMD5*	0.327	0.070	**4.5E-06**	361	GTEx-V6p_eQTL_EUR_2016
WHRadjBMI	rs13301996	9	120570806	*CDK5RAP2*	G	T	25954001	Unspecified	Muscle skeletal	*PSMD5-AS1*	0.302	0.072	**3.7E-05**	491	GTEx-V7_eQTL_EUR_2017
WHRadjBMI	rs13301996	9	120570806	*CDK5RAP2*	G	T	25954001	Unspecified	Muscle skeletal	*PSMD5-AS1*	0.320	0.090	**4.2E-04**	361	GTEx-V6p_eQTL_EUR_2016
WHRadjBMI	rs13301996	9	120570806	*CDK5RAP2*	G	T	25954001	Unspecified	Nerve tibial	*PSMD5-AS1*	0.461	0.091	**6.6E-07**	361	GTEx-V7_eQTL_EUR_2017
WHRadjBMI	rs13301996	9	120570806	*CDK5RAP2*	G	T	25954001	Unspecified	Nerve tibial	*PSMD5-AS1*	0.428	0.105	**6.2E-05**	256	GTEx-V6p_eQTL_EUR_2016
WHRadjBMI	rs13301996	9	120570806	*CDK5RAP2*	G	T	25954001	Unspecified	Skin not sun exposed suprapubic	*PSMD5-AS1*	0.333	0.083	**8.3E-05**	335	GTEx-V7_eQTL_EUR_2017
WHRadjBMI	rs13301996	9	120570806	*CDK5RAP2*	G	T	25954001	Unspecified	Skin sun exposed lower leg	*PSMD5-AS1*	0.328	0.082	**7.5E-05**	414	GTEx-V7_eQTL_EUR_2017
WHRadjBMI	rs13301996	9	120570806	*CDK5RAP2*	G	T	25954001	Unspecified	Skin sun exposed lower leg	*PSMD5-AS1*	0.387	0.096	**8.0E-05**	302	GTEx-V6p_eQTL_EUR_2016
WHRadjBMI	rs13301996	9	120570806	*CDK5RAP2*	G	T	25954001	Unspecified	Skin sun exposed lower leg	*MEGF9*	0.123	0.033	**2.8E-04**	302	GTEx-V6p_eQTL_EUR_2016
WHRadjBMI	rs13301996	9	120570806	*CDK5RAP2*	G	T	27863251	European	T cells	*MEGF9*	-0.437	0.126	**5.5E-04**	169	BLUEPRINT_eQTL_EUR_2016
WHRadjBMI	rs13301996	9	120570806	*CDK5RAP2*	G	T	25954001	Unspecified	Testis	*CDK5RAP2*	0.251	0.060	**4.8E-05**	225	GTEx-V7_eQTL_EUR_2017
WHRadjBMI	rs13301996	9	120570806	*CDK5RAP2*	G	T	25954001	Unspecified	Thyroid	*PSMD5-AS1*	0.395	0.082	**2.4E-06**	399	GTEx-V7_eQTL_EUR_2017
WHRadjBMI	rs13301996	9	120570806	*CDK5RAP2*	G	T	25954001	Unspecified	Thyroid	*PSMD5*	0.194	0.051	**1.8E-04**	399	GTEx-V7_eQTL_EUR_2017
WHRadjBMI	rs13301996	9	120570806	*CDK5RAP2*	G	T	25954001	Unspecified	Thyroid	*PSMD5-AS1*	0.365	0.101	**3.6E-04**	278	GTEx-V6p_eQTL_EUR_2016
WHRadjBMI	rs13301996	9	120570806	*CDK5RAP2*	G	T	eQTLGen	European	Whole blood	*MEGF9*	NA	NA	**1.8E-149**	30523	eQTLGen_eQTL_EUR_2018
WHRadjBMI	rs13301996	9	120570806	*CDK5RAP2*	G	T	eQTLGen	European	Whole blood	*RP11-27I1.2*	NA	NA	**8.5E-107**	16488	eQTLGen_eQTL_EUR_2018
WHRadjBMI	rs13301996	9	120570806	*CDK5RAP2*	G	T	eQTLGen	European	Whole blood	*CDK5RAP2*	NA	NA	**1.7E-61**	30737	eQTLGen_eQTL_EUR_2018
WHRadjBMI	rs13301996	9	120570806	*CDK5RAP2*	G	T	eQTLGen	European	Whole blood	*PSMD5*	NA	NA	**1.6E-10**	30737	eQTLGen_eQTL_EUR_2018
WHRadjBMI	rs13301996	9	120570806	*CDK5RAP2*	G	T	eQTLGen	European	Whole blood	*PHF19*	NA	NA	**1.0E-08**	29988	eQTLGen_eQTL_EUR_2018
WHRadjBMI	rs13301996	9	120570806	*CDK5RAP2*	G	T	eQTLGen	European	Whole blood	*GGTA1P*	NA	NA	**1.5E-07**	24529	eQTLGen_eQTL_EUR_2018
WHRadjBMI	rs13301996	9	120570806	*CDK5RAP2*	G	T	28122634	Mixed	Whole blood	*MEGF9*	-0.018	0.003	**2.6E-07**	5257	Joehanes-R_eQTL_EUR_2017
WHRadjBMI	rs13301996	9	120570806	*CDK5RAP2*	G	T	28122634	Mixed	Whole blood	*CYP4Z1;CYP4Z2P*	-0.030	0.007	**7.7E-06**	5257	Joehanes-R_eQTL_EUR_2017
WHRadjBMI	rs13301996	9	120570806	*CDK5RAP2*	G	T	28122634	Mixed	Whole blood	*RAB14*	0.015	0.003	**7.8E-06**	5257	Joehanes-R_eQTL_EUR_2017
WHRadjBMI	rs13301996	9	120570806	*CDK5RAP2*	G	T	eQTLGen	European	Whole blood	*RAB14*	NA	NA	**8.0E-06**	29988	eQTLGen_eQTL_EUR_2018

WHRadjBMI	rs13301996	9	120570806	*CDK5RAP2*	G	T	28122634	Mixed	Whole blood	*BPIFB3*	0.013	0.003	**3.9E-05**	5257	Joehanes-R_eQTL_EUR_2017
WHRadjBMI	rs13301996	9	120570806	*CDK5RAP2*	G	T	28122634	Mixed	Whole blood	*PSMD5*	0.018	0.004	**4.4E-05**	5257	Joehanes-R_eQTL_EUR_2017
WHRadjBMI	rs13301996	9	120570806	*CDK5RAP2*	G	T	28122634	Mixed	Whole blood	*RBM3;SLC38A5*	0.013	0.003	**5.1E-05**	5257	Joehanes-R_eQTL_EUR_2017
WHRadjBMI	rs13301996	9	120570806	*CDK5RAP2*	G	T	28122634	Mixed	Whole blood	*SIN3A*	0.010	0.002	**7.7E-05**	5257	Joehanes-R_eQTL_EUR_2017
WHRadjBMI	rs13301996	9	120570806	*CDK5RAP2*	G	T	25954001	Unspecified	Whole blood	*PSMD5-AS1*	0.270	0.068	**9.4E-05**	369	GTEx-V7_eQTL_EUR_2017
WHRadjBMI	rs13301996	9	120570806	*CDK5RAP2*	G	T	28122634	Mixed	Whole blood	*MRPL14*	-0.019	0.005	**9.9E-05**	5257	Joehanes-R_eQTL_EUR_2017
WCadjBMI	rs3168072	11	61864038	*FADS2*	A	T	25954001	Unspecified	Artery tibial	*INCENP*	-0.623	0.129	**2.5E-06**	285	GTEx-V6p_eQTL_EUR_2016
WCadjBMI	rs3168072	11	61864038	*FADS2*	A	T	25954001	Unspecified	Artery tibial	*INCENP*	-0.358	0.101	**4.6E-04**	388	GTEx-V7_eQTL_EUR_2017
WCadjBMI	rs3168072	11	61864038	*FADS2*	A	T	25954001	Unspecified	Brain caudate basal ganglia	*LRRN4CL*	1.155	0.325	**5.4E-04**	144	GTEx-V7_eQTL_EUR_2017
WCadjBMI	rs3168072	11	61864038	*FADS2*	A	T	25954001	Unspecified	Cells EBV-transformed lymphocytes	*SDHAF2*	0.865	0.229	**2.8E-04**	117	GTEx-V7_eQTL_EUR_2017
WCadjBMI	rs3168072	11	61864038	*FADS2*	A	T	25954001	Unspecified	Nerve tibial	*SDHAF2*	0.551	0.155	**4.6E-04**	256	GTEx-V6p_eQTL_EUR_2016
WCadjBMI	rs3168072	11	61864038	*FADS2*	A	T	25954001	Unspecified	Pancreas	*UBXN1*	0.411	0.110	**2.5E-04**	220	GTEx-V7_eQTL_EUR_2017
WCadjBMI	rs3168072	11	61864038	*FADS2*	A	T	25954001	Unspecified	Skin not sun exposed suprapubic	*TAF6L*	0.368	0.098	**2.1E-04**	335	GTEx-V7_eQTL_EUR_2017
WCadjBMI	rs3168072	11	61864038	*FADS2*	A	T	25954001	Unspecified	Skin sun exposed lower leg	*TMEM258*	-0.409	0.117	**5.6E-04**	302	GTEx-V6p_eQTL_EUR_2016
WCadjBMI	rs3168072	11	61864038	*FADS2*	A	T	25954001	Unspecified	Small intestine terminal ileum	*SDHAF2*	0.813	0.210	**1.9E-04**	122	GTEx-V7_eQTL_EUR_2017
WCadjBMI	rs3168072	11	61864038	*FADS2*	A	T	eQTLGen	European	Whole blood	*TMEM258*	NA	NA	**5.8E-11**	25068	eQTLGen_eQTL_EUR_2018
WCadjBMI	rs3168072	11	61864038	*FADS2*	A	T	28122634	Mixed	Whole blood	*C11orf10*	-0.053	0.009	**1.3E-09**	5257	Joehanes-R_eQTL_EUR_2017
WCadjBMI	rs3168072	11	61864038	*FADS2*	A	T	28122634	Mixed	Whole blood	*XG;XGPY2*	0.049	0.012	**2.9E-05**	5257	Joehanes-R_eQTL_EUR_2017
WCadjBMI	rs3168072	11	61864038	*FADS2*	A	T	eQTLGen	European	Whole blood	*FTH1*	NA	NA	**3.3E-05**	30638	eQTLGen_eQTL_EUR_2018
WCadjBMI	rs3168072	11	61864038	*FADS2*	A	T	28122634	Mixed	Whole blood	*APOA4*	-0.056	0.014	**4.7E-05**	5257	Joehanes-R_eQTL_EUR_2017
WCadjBMI	rs3168072	11	61864038	*FADS2*	A	T	eQTLGen	European	Whole blood	*MYRF*	NA	NA	**5.7E-05**	25068	eQTLGen_eQTL_EUR_2018
WCadjBMI	rs3168072	11	61864038	*FADS2*	A	T	28122634	Mixed	Whole blood	*MED21*	-0.046	0.011	**6.0E-05**	5257	Joehanes-R_eQTL_EUR_2017
WCadjBMI	rs3168072	11	61864038	*FADS2*	A	T	28122634	Mixed	Whole blood	*POU4F2*	0.040	0.010	**6.4E-05**	5257	Joehanes-R_eQTL_EUR_2017
WCadjBMI	rs3168072	11	61864038	*FADS2*	A	T	eQTLGen	European	Whole blood	*FADS1*	NA	NA	**5.0E-04**	31062	eQTLGen_eQTL_EUR_2018
HIPadjBMI	rs28692724	14	77027445	*IRF2BPL*	C	T	eQTLGen	European	Whole blood	*IRF2BPL*	NA	NA	**8.6E-23**	6544	eQTLGen_eQTL_EUR_2018
HIPadjBMI	rs28692724	14	77027445	*IRF2BPL*	C	T	27918533	European	Whole blood	*RP11-7F17.5*	NA	NA	**2.1E-10**	2116	BIOSQTL_eQTL_EUR_2017
HIPadjBMI	rs28692724	14	77027445	*IRF2BPL*	C	T	27918533	European	Whole blood	*RP11-7F17.5;RP11-7F17.3*	NA	NA	**1.4E-09**	2116	BIOSQTL_eQTL_EUR_2017
HIPadjBMI	rs28692724	14	77027445	*IRF2BPL*	C	T	27918533	European	Whole blood	*RP11-7F17.3*	NA	NA	**1.6E-09**	2116	BIOSQTL_eQTL_EUR_2017
HIPadjBMI	rs28692724	14	77027445	*IRF2BPL*	C	T	27918533	European	Whole blood	*RP11-7F17.7*	NA	NA	**2.2E-08**	2116	BIOSQTL_eQTL_EUR_2017
HIPadjBMI	rs28692724	14	77027445	*IRF2BPL*	C	T	27918533	European	Whole blood	*IRF2BPL*	NA	NA	**2.3E-07**	2116	BIOSQTL_eQTL_EUR_2017
HIPadjBMI	rs28692724	14	77027445	*IRF2BPL*	C	T	25954001	Unspecified	Colon sigmoid	*ANGEL1*	0.447	0.119	**2.7E-04**	124	GTEx-V6p_eQTL_EUR_2016

Supplementary Table 15. mQTL lookups of replicated variants from current study in Phenoscanner. Only significant SNP-metabolite associations shown ($P<0.05/488=P<1.02x10^{-4}$).

Discovery Trait	dbSNPID	CHR	POS (GRCh38)	Effect Allele	Other Allele	Metabolite	PMID/ Source	Ancestry	Beta	SE	P	N
WCadjBMI	rs3168072	11	61864038	A	T	Other polyunsaturated fatty acids than 18:2	27005778	European	0.235	0.034	**7.4E-12**	13549
WCadjBMI	rs3168072	11	61864038	A	T	CH2 groups in fatty acids	27005778	European	-0.169	0.032	**1.3E-07**	19021
WCadjBMI	rs3168072	11	61864038	A	T	Ratio of bis allylic bonds to double bonds in lipids	27005778	European	0.180	0.034	**1.8E-07**	13524
WCadjBMI	rs3168072	11	61864038	A	T	CH2 groups to double bonds ratio	27005778	European	-0.171	0.034	**5.7E-07**	13532
WCadjBMI	rs3168072	11	61864038	A	T	Ratio of bis allylic bonds to total fatty acids in lipids	27005778	European	0.171	0.035	**9.3E-07**	13171

Supplementary Table 16. Lookups of replicated variants and variants in high LD (R^2>0.8) HaploReg v4.1.

SOL SNP	Trait	CHR	POS (hg38)	R^2	D'	rsID	REF	ALT	AFR	AMR	ASN	EUR	Chromatin States	Chromatin States Imputed	Chromatin Marks	DNAse	Proteins	eQTL	Motifs	GENCODE ID	GENCODE Name	RefSeq ID	RefSeq Gene Name	dbSNP Functional Annotation	Enhancer Histone Marks
rs13301996	WHRadjBMI (Combined)	9	120569993	0.84	1	rs13299371	A	T	0.13	0.19	0.09	0.17	E017,6_EnhG;E049,6_EnhG;E068,6_EnhG;E069,6_EnhG;E090,6_EnhG;E108,6_EnhG;E118,6_EnhG;E121,7_Enh	E017,11_TxEnh3;E026,11_TxEnh3;E049,11_TxEnh3;E053,11_TxEnh3;E056,11_TxEnh3;E065,11_TxEnh3;E067,11_TxEnh3;E068,11_TxEnh3;E069,11_TxEnh3;E070,11_TxEnh3;E071,11_TxEnh3;E072,11_TxEnh3;E073,11_TxEnh3;E074,11_TxEnh3;E108,11_TxEnh3;E120,11_TxEnh3;E121,11_TxEnh3;E129,11_TxEnh3	E007,H3K9ac_Pro;E017,H3K9ac_Pro;E023,H3K9ac_Pro;E026,H3K9ac_Pro;E027,H3K9ac_Pro;E052,H3K9ac_Pro;E062,H3K9ac_Pro;E063,H3K9ac_Pro;E066,H3K9ac_Pro;E067,H3K9ac_Pro;E068,H3K9ac_Pro;E069,H3K9ac_Pro;E072,H3K9ac_Pro;E073,H3K9ac_Pro;E074,H3K9ac_Pro;E107,H3K9ac_Pro;E108,H3K9ac_Pro;E121,H3K9ac_Pro;E125,H3K9ac_Pro;E126,H3K9ac_Pro;E011,H3K27ac_Enh;E013,H3K27ac_Enh;E017,H3K27ac_Enh;E026,H3K27ac_Enh;E049,H3K27ac_Enh;E055,H3K27ac_Enh;E056,H3K27ac_Enh;E058,H3K27ac_Enh;E061,H3K27ac_Enh;E065,H3K27ac_Enh;E066,H3K27ac_Enh;E067,H3K27ac_Enh;E068,H3K27ac_Enh;E069,H3K27ac_Enh;E071,H3K27ac_Enh;E072,H3K27ac_Enh;E073,H3K27ac_Enh;E074,H3K27ac_Enh;E084,H3K27ac_Enh;E096,H3K27ac_Enh;E097,H3K27ac_Enh;E098,H3K27ac_Enh;E104,H3K27ac_Enh;E111,H3K27ac_Enh;E118,H3K27ac_Enh;E120,H3K27ac_Enh;E121,H3K27ac_Enh;E124,H3K27ac_Enh;E125,H3K27ac_Enh;E126,H3K27ac_Enh;E128,H3K27ac_Enh;E129,H3K27ac_Enh;E017,H3K4me1_Enh;E023,H3K4me1_Enh;E026,H3K4me1_Enh;E027,H3K4me1_Enh;E049,H3K4me1_Enh;E053,H3K4me1_Enh;E054,H3K4me1_Enh;E055,H3K4me1_Enh;E056,H3K4me1_Enh;E065,H3K4me1_Enh;E066,H3K4me1_Enh;E067,H3K4me1_Enh;E068,H3K4me1_Enh;E069,H3K4me1_Enh;E071,H3K4me1_Enh;E072,H3K4me1_Enh;E073,H3K4me1_Enh;E074,H3K4me1_Enh;E076,H3K4me1_Enh;E090,H3K4me1_Enh;E094,H3K4me1_Enh;E096,H3K4me1_Enh;E097,H3K4me1_Enh;E107,H3K4me1_Enh;E108,H3K4me1_Enh;E111,H3K4me1_Enh;E118,H3K4me1_Enh;E121,H3K4me1_Enh;E128,H3K4me1_Enh;E129,H3K4me1_Enh;E096,H3K4me3_Pro;E121,H3K4me3_Pro;E129,H3K4me3_Pro		.	GTEx2015_v6,Adipose_Subcutaneous,MEGF9,2.22717446123139e-05;GTEx2015_v6,Muscle_Skeletal,PSMD5,5.09639056923052e-06;Westra2013,Whole_Blood,CDK5RAP2,1.9918214956123527E-28;Westra2013,Whole_Blood,MEGF9,6.525126050211568E-31	Foxc1_2;PTF1-beta;SPIB	ENSG00000136861.12	CDK5RAP2	NM_018249	CDK5RAP2	Intronic	
**rs13301996**	WHRadjBMI (Combined)	9	120570806	1	1	**rs13301996**	T	G	0.13	0.22	0.09	0.18	E017,6_EnhG;E023,7_Enh;E026,7_Enh;E027,7_Enh;E049,7_Enh;E053,6_EnhG;E055,6_EnhG;E063,6_EnhG;E066,7_Enh;E067,6_EnhG;E068,6_EnhG;E084,6_EnhG;E089,6_EnhG;E090,6_EnhG;E107,6_EnhG;E108,7_Enh;E118,6_EnhG;E121,7_Enh	E006,11_TxEnh3;E017,11_TxEnh3;E023,15_EnhAF;E025,15_EnhAF;E026,14_EnhA2;E027,11_TxEnh3;E049,15_EnhAF;E052,11_TxEnh3;E053,11_TxEnh3;E054,11_TxEnh3;E055,11_TxEnh3;E063,11_TxEnh3;E065,17_EnhW2;E066,11_TxEnh3;E067,11_TxEnh3;E068,11_TxEnh3;E069,11_TxEnh3;E070,11_TxEnh3;E071,11_TxEnh3;E072,11_TxEnh3;E073,11_TxEnh3;E074,11_TxEnh3;E076,11_TxEnh3;E078,11_TxEnh3;E084,11_TxEnh3;E085,11_TxEnh3;E089,11_TxEnh3;E090,11_TxEnh3;E095,11_TxEnh3;E097,11_TxEnh3;E103,11_TxEnh3;E107,11_TxEnh3;E108,11_TxEnh3;E111,11_TxEnh3;E118,11_TxEnh3;E120,11_TxEnh3;E121,15_EnhAF;E125,11_TxEnh3;E126,11_TxEnh3;E128,11_TxEnh3;E129,15_EnhAF	E006,H3K4me1_Enh;E017,H3K4me1_Enh;E023,H3K4me1_Enh;E026,H3K4me1_Enh;E027,H3K4me1_Enh;E049,H3K4me1_Enh;E052,H3K4me1_Enh;E053,H3K4me1_Enh;E054,H3K4me1_Enh;E055,H3K4me1_Enh;E056,H3K4me1_Enh;E063,H3K4me1_Enh;E065,H3K4me1_Enh;E066,H3K4me1_Enh;E067,H3K4me1_Enh;E068,H3K4me1_Enh;E069,H3K4me1_Enh;E071,H3K4me1_Enh;E072,H3K4me1_Enh;E073,H3K4me1_Enh;E074,H3K4me1_Enh;E076,H3K4me1_Enh;E078,H3K4me1_Enh;E084,H3K4me1_Enh;E089,H3K4me1_Enh;E090,H3K4me1_Enh;E095,H3K4me1_Enh;E096,H3K4me1_Enh;E097,H3K4me1_Enh;E100,H3K4me1_Enh;E103,H3K4me1_Enh;E104,H3K4me1_Enh;E107,H3K4me1_Enh;E108,H3K4me1_Enh;E111,H3K4me1_Enh;E118,H3K4me1_Enh;E120,H3K4me1_Enh;E121,H3K4me1_Enh;E125,H3K4me1_Enh;E128,H3K4me1_Enh;E129,H3K4me1_Enh;E007,H3K9ac_Pro;E017,H3K9ac_Pro;E023,H3K9ac_Pro;E026,H3K9ac_Pro;E027,H3K9ac_Pro;E052,H3K9ac_Pro;E063,H3K9ac_Pro;E066,H3K9ac_Pro;E067,H3K9ac_Pro;E068,H3K9ac_Pro;E069,H3K9ac_Pro;E072,H3K9ac_Pro;E073,H3K9ac_Pro;E074,H3K9ac_Pro;E107,H3K9ac_Pro;E108,H3K9ac_Pro;E118,H3K9ac_Pro;E120,H3K9ac_Pro;E121,H3K9ac_Pro;E125,H3K9ac_Pro;E126,H3K9ac_Pro;E011,H3K27ac_Enh;E013,H3K27ac_Enh;E017,H3K27ac_Enh;E026,H3K27ac_Enh;E049,H3K27ac_Enh;E055,H3K27ac_Enh;E056,H3K27ac_Enh;E063,H3K27ac_Enh;E065,H3K27ac_Enh;E066,H3K27ac_Enh;E067,H3K27ac_Enh;E068,H3K27ac_Enh;E069,H3K27ac_Enh;E071,H3K27ac_Enh;E072,H3K27ac_Enh;E073,H3K27ac_Enh;E074,H3K27ac_Enh;E076,H3K27ac_Enh;E079,H3K27ac_Enh;E084,H3K27ac_Enh;E085,H3K27ac_Enh;E090,H3K27ac_Enh;E095,H3K27ac_Enh;E096,H3K27ac_Enh;E097,H3K27ac_Enh;E098,H3K27ac_Enh;E100,H3K27ac_Enh;E103,H3K27ac_Enh;E104,H3K27ac_Enh;E105,H3K27ac_Enh;E108,H3K27ac_Enh;E111,H3K27ac_Enh;E118,H3K27ac_Enh;E120,H3K27ac_Enh;E121,H3K27ac_Enh;E124,H3K27ac_Enh;E125,H3K27ac_Enh;E126,H3K27ac_Enh;E128,H3K27ac_Enh;E129,H3K27ac_Enh;E026,H3K4me3_Pro;E066,H3K4me3_Pro;E096,H3K4me3_Pro;E107,H3K4me3_Pro;E108,H3K4me3_Pro;E111,H3K4me3_Pro;E121,H3K4me3_Pro;E129,H3K4me3_Pro		.	GTEx2015_v6,Muscle_Skeletal,PSMD5,4.10599382929573e-06	AhR_1;Maf_known2;Zbtb3	ENSG00000136861.12	CDK5RAP2	NM_018249	CDK5RAP2	Intronic	
rs79478137	WHRadjBMI (Women)	11	2888469	1	1	rs80153297	C	T	0.09	0.02	0.01	0.0026	E001,12_EnhBiv;E003,12_EnhBiv;E005,7_Enh;E006,7_Enh;E009,7_Enh;E015,12_EnhBiv;E017,7_Enh;E020,12_EnhBiv;E022,7_Enh;E029,12_EnhBiv;E032,7_Enh;E034,7_Enh;E035,7_Enh;E036,7_Enh;E037,7_Enh;E038,7_Enh;E039,7_Enh;E040,7_Enh;E041,7_Enh;E042,7_Enh;E043,7_Enh;E044,7_Enh;E047,7_Enh;E048,7_Enh;E050,7_Enh;E051,7_Enh;E052,7_Enh;E053,7_Enh;E055,7_Enh;E056,7_Enh;E059,2_TssAFlnk;E063,7_Enh;E068,7_Enh;E070,12_EnhBiv;E071,7_Enh;E077,7_Enh;E080,7_Enh;E082,7_Enh;E085,7_Enh;E086,7_Enh;E088,7_Enh;E089,7_Enh;E090,7_Enh;E091,7_Enh;E092,7_Enh;E093,7_Enh;E097,7_Enh;E105,7_Enh;E106,7_Enh;E107,7_Enh;E108,7_Enh;E111,7_Enh;E113,7_Enh;E117,2_TssAFlnk;E118,7_Enh;E121,7_Enh;E123,7_Enh;E127,7_Enh	E005,16_EnhW1;E006,16_EnhW1;E007,23_PromBiv;E008,23_PromBiv;E009,19_DNase;E010,19_DNase;E013,19_DNase;E017,16_EnhW1;E018,23_PromBiv;E019,23_PromBiv;E020,23_PromBiv;E022,23_PromBiv;E023,19_DNase;E025,19_DNase;E026,19_DNase;E027,16_EnhW1;E028,19_DNase;E029,17_EnhW2;E030,17_EnhW2;E031,17_EnhW2;E032,16_EnhW1;E033,17_EnhW2;E034,14_EnhA2;E035,17_EnhW2;E036,16_EnhW1;E037,16_EnhW1;E038,16_EnhW1;E039,16_EnhW1;E040,17_EnhW2;E041,16_EnhW1;E042,16_EnhW1;E043,16_EnhW1;E044,16_EnhW1;E045,17_EnhW2;E046,14_EnhA2;E047,16_EnhW1;E048,16_EnhW1;E049,19_DNase;E050,14_EnhA2;E051,14_EnhA2;E052,19_DNase;E053,16_EnhW1;E054,16_EnhW1;E055,16_EnhW1;E056,14_EnhA2;E057,17_EnhW2;E058,19_DNase;E059,16_EnhW1;E061,17_EnhW2;E062,17_EnhW2;E063,16_EnhW1;E065,19_DNase;E066,19_DNase;E067,16_EnhW1;E068,16_EnhW1;E069,16_EnhW1;E070,16_EnhW1;E071,16_EnhW1;E072,16_EnhW1;E073,16_EnhW1;E074,16_EnhW1;E075,17_EnhW2;E076,17_EnhW2;E077,16_EnhW1;E078,17_EnhW2;E079,16_EnhW1;E080,14_EnhA2;E081,16_EnhW1;E082,16_EnhW1;E083,16_EnhW1;E084,17_EnhW2;E085,17_EnhW2;E086,16_EnhW1;E087,17_EnhW2;E088,14_EnhA2;E089,14_EnhA2;E090,14_EnhA2;E091,14_EnhA2;E092,14_EnhA2;E093,16_EnhW1;E094,17_EnhW2;E095,19_DNase;E096,17_EnhW2;E097,17_EnhW2;E098,16_EnhW1;E099,16_EnhW1;E100,17_EnhW2;E101,16_EnhW1;E102,16_EnhW1;E103,16_EnhW1;E104,19_DNase;E105,16_EnhW1;E106,16_EnhW1;E107,16_EnhW1;E108,16_EnhW1;E109,16_EnhW1;E110,17_EnhW2;E111,16_EnhW1;E112,16_EnhW1;E113,17_EnhW2;E114,	E001,H3K4me1_Enh;E003,H3K4me1_Enh;E005,H3K4me1_Enh;E006,H3K4me1_Enh;E007,H3K4me1_Enh;E009,H3K4me1_Enh;E011,H3K4me1_Enh;E013,H3K4me1_Enh;E014,H3K4me1_Enh;E015,H3K4me1_Enh;E017,H3K4me1_Enh;E018,H3K4me1_Enh;E019,H3K4me1_Enh;E020,H3K4me1_Enh;E021,H3K4me1_Enh;E022,H3K4me1_Enh;E023,H3K4me1_Enh;E024,H3K4me1_Enh;E025,H3K4me1_Enh;E027,H3K4me1_Enh;E030,H3K4me1_Enh;E032,H3K4me1_Enh;E034,H3K4me1_Enh;E035,H3K4me1_Enh;E036,H3K4me1_Enh;E037,H3K4me1_Enh;E038,H3K4me1_Enh;E039,H3K4me1_Enh;E040,H3K4me1_Enh;E041,H3K4me1_Enh;E042,H3K4me1_Enh;E043,H3K4me1_Enh;E044,H3K4me1_Enh;E045,H3K4me1_Enh;E046,H3K4me1_Enh;E047,H3K4me1_Enh;E048,H3K4me1_Enh;E050,H3K4me1_Enh;E051,H3K4me1_Enh;E053,H3K4me1_Enh;E054,H3K4me1_Enh;E055,H3K4me1_Enh;E056,H3K4me1_Enh;E057,H3K4me1_Enh;E058,H3K4me1_Enh;E059,H3K4me1_Enh;E061,H3K4me1_Enh;E063,H3K4me1_Enh;E066,H3K4me1_Enh;E067,H3K4me1_Enh;E068,H3K4me1_Enh;E069,H3K4me1_Enh;E070,H3K4me1_Enh;E071,H3K4me1_Enh;E072,H3K4me1_Enh;E073,H3K4me1_Enh;E074,H3K4me1_Enh;E075,H3K4me1_Enh;E077,H3K4me1_Enh;E078,H3K4me1_Enh;E079,H3K4me1_Enh;E080,H3K4me1_Enh;E081,H3K4me1_Enh;E082,H3K4me1_Enh;E083,H3K4me1_Enh;E084,H3K4me1_Enh;E085,H3K4me1_Enh;E088,H3K4me1_Enh;E089,H3K4me1_Enh;E090,H3K4me1_Enh;E091,H3K4me1_Enh;E092,H3K4me1_Enh;E093,H3K4me1_Enh;E094,H3K4me1_Enh;E097,H3K4me1_Enh;E098,H3K4me1_Enh;E099,H3K4me1_Enh;E101,H3K4me1_Enh;E102,H3K4me1_Enh;E103,H3K4me1_Enh;E105,H3K4me1_Enh;E106,H3K4me1_Enh;E107,H3K4me1_Enh;E108,H3K4me1_Enh;E109,H3K4me1_Enh;E110,H3K4me1_Enh;E111,H3K4me1_Enh;E112,H3K4me1_Enh;E113,H3K4me1_Enh;E117,H3K4me1_Enh;E118,H3K4me1_Enh;E121,H3K4me1_Enh;E123,H3K4me1_Enh;E124,H3K4me1_Enh;E125,H3K4me1_Enh;E127,H3K4me1_Enh;E129,H3K4me1_Enh;E005,H3K4me3_Pro;E018,H3K4me3_Pro;E019,H3K4me3_Pro;E020,H3K4me3_Pro;E022,H3K4me3_Pr;E038,H3K4me3_Pro;E039,H3K4me3_Pro;E042,H3K4me3_Pro;E044,H3K4me3_Pro;E047,H3K4me3_Pro;E051,H3K4me3_Pro;E055,H3K4me3_Pro;E056,H3K4me3_Pro;E059,H3K4me3_ProE069,H3K4me3_Pro;E070,H3K4me3_Pro;E074,H3K4me3_Pro;E076,H3K4me3_Pro;E077,H3K4me3_Pro;E078,H3K4me3_Pro;E082,H3K4me3_Pro;E088,H3K4me3_Pro;E101,H3K4me3_Pro;	E003;E004E005;E006;E007;E008;E017;E021;E022;E028;E029E032;E033;E034;E046;E050;E051;E055;E056;E057E059;E080;E081;E082;E083;E084;E085;E086;E088;E089;E090;E091;E092;E093;E094;E097;E098;E100;E109;E114;E116;E117;E118;E119;E120;E121;E122E123;E12	A549,CTCF,HudsonAlpha,EtOH_0.02pct;AG04449,CTCF,UW,None;AG04450,CTCF,UW,None;AG09309,CTCF,UW,None;AG09319,CTCF,UW,None;AG10803,CTCF,UW,None;AoAF,CTCF,UW,None;BJ,CTCF,UW,None;Caco-2,CTCF,UW,None;Fibrobl,CTCF,UT-A,None;G	.	BCL_disc9	ENSG00000254827.1	SLC22A18AS	NM_007105	SLC22A18AS	Nonsynonymous	SKIN, CRVX

rs79478137	WHRadjBMI (Women)	11	2888527	1	1	rs76505120	C	T	0.11	0.02	0	0.0013	E001,12_EnhBiv;E003,12_EnhBiv;E005,7_Enh;E006,7_Enh;E009,7_Enh;E015,12_EnhBiv;E017,7_Enh;E020,12_EnhBiv;E022,7_Enh;E029,12_EnhBiv;E032,7_Enh;E034,7_Enh;E035,7_Enh;E036,7_Enh;E037,7_Enh;E038,7_Enh;E039,7_Enh;E040,7_Enh;E041,7_Enh;E042,7_Enh;E043,7_Enh;E044,7_Enh;E047,7_Enh;E048,7_Enh;E050,7_Enh;E051,7_Enh;E052,7_Enh;E053,7_Enh;E055,7_Enh;E056,7_Enh;E059,2_TssAFlnk;E063,7_Enh;E068,7_Enh;E070,12_EnhBiv;E071,7_Enh;E077,7_Enh;E080,7_Enh;E082,7_Enh;E085,7_Enh;E086,7_Enh;E088,7_Enh;E089,7_Enh;E090,7_Enh;E091,7_Enh;E092,7_Enh;E093,7_Enh;E097,7_Enh;E105,7_Enh;E106,7_Enh;E107,7_Enh;E108,7_Enh;E111,7_Enh;E113,7_Enh;E117,2_TssAFlnk;E118,7_Enh;E121,7_Enh;E123,7_Enh;E127,7_Enh	E005,16_EnhW1;E006,16_EnhW1;E007,23_PromBiv;E008,23_PomBiv;E009,19_DNase;E010,19_DNase;E013,19_DNase;E017,16_EnhW1;E018,23_PromBiv;E019,23_PromBiv;E020,23_PromBiv;E022,23_PromBiv;E023,19_DNase;E025,19_DNase;E026,19_DNase;E027,16_EnhW1;E028,19_DNase;E029,17_EnhW2;E030,17_EnhW2;E031,17_EnhW2;E032,16_EnhW1;E033,17_EnhW2;E034,14_EnhA2;E035,17_EnhW2;E036,16_EnhW1;E037,16_EnhW1;E038,16_EnhW1;E039,16_EnhW1;E040,17_EnhW2;E041,16_EnhW1;E042,16_EnhW1;E043,16_EnhW1;E044,16_EnhW1;E045,17_EnhW2;E046,14_EnhA2;E047,16_EnhW1;E048,16_EnhW1;E049,19_DNase;E050,14_EnhA2;E051,14_EnhA2;E052,19_DNase;E053,16_EnhW1;E054,16_EnhW1;E055,16_EnhW1;E056,14_EnhA2;E057,17_EnhW2;E058,19_DNase;E059,16_EnhW1;E061,17_EnhW2;E062,17_EnhW2;E063,16_EnhW1;E065,19_DNase;E066,19_DNase;E067,16_EnhW1;E068,16_EnhW1;E069,16_EnhW1;E070,16_EnhW1;E071,16_EnhW1;E072,16_EnhW1;E073,16_EnhW1;E074,16_EnhW1;E075,17_EnhW2;E076,17_EnhW2;E077,16_EnhW1;E078,17_EnhW2;E079,16_EnhW1;E080,14_EnhA2;E081,16_EnhW1;E082,16_EnhW1;E083,16_EnhW1;E084,17_EnhW2;E085,17_EnhW2;E086,16_EnhW1;E087,17_EnhW2;E088,14_EnhA2;E089,14_EnhA2;E090,14_EnhA2;E091,14_EnhA2;E092,14_EnhA2;E093,16_EnhW1;E094,17_EnhW2;E095,19_DNase;E096,17_EnhW2;E097,17_EnhW2;E098,16_EnhW1;E099,16_EnhW1;E100,17_EnhW2;E101,16_EnhW1;E102,16_EnhW1;E103,16_EnhW1;E104,19_DNase;E105,16_EnhW1;E106,16_EnhW1;E107,16_EnhW1;E108,16_EnhW1;E109,16_EnhW1;E110,17_EnhW2;E111,16_EnhW1;E112,16_EnhW1;E113,17_EnhW2;E114,	E001,H3K4me1_Enh;E005,H3K4me1_Enh;E006,H3K4me1_Enh;E007,H3K4me1_Enh;E009,H3K4me1_Enh;E011,H3K4me1_Enh;E013,H3K4me1_Enh;E014,H3K4me1_Enh;E015,H3K4me1_Enh;E018,H3K4me1_Enh;E019,H3K4me1_Enh;E020,H3K4me1_Enh;E021,H3K4me1_Enh;E022,H3K4me1_Enh;E023,H3K4me1_Enh;E024,H3K4me1_Enh;E025,H3K4me1_Enh;E027,H3K4me1_Enh;E030,H3K4me1_Enh;E032,H3K4me1_Enh;E034,H3K4me1_Enh;E035,H3K4me1_Enh;E036,H3K4me1_Enh;E037,H3K4me1_Enh;E038,H3K4me1_Enh;E039,H3K4me1_Enh;E040,H3K4me1_Enh;E041,H3K4me1_Enh;E042,H3K4me1_Enh;E043,H3K4me1_Enh;E044,H3K4me1_Enh;E045,H3K4me1_Enh;E046,H3K4me1_Enh;E047,H3K4me1_Enh;E048,H3K4me1_Enh;E050,H3K4me1_Enh;E051,H3K4me1_Enh;E053,H3K4me1_Enh;E054,H3K4me1_Enh;E055,H3K4me1_Enh;E056,H3K4me1_Enh;E057,H3K4me1_Enh;E058,H3K4me1_Enh;E059,H3K4me1_Enh;E061,H3K4me1_Enh;E063,H3K4me1_Enh;E066,H3K4me1_Enh;E067,H3K4me1_Enh;E068,H3K4me1_Enh;E069,H3K4me1_Enh;E070,H3K4me1_Enh;E071,H3K4me1_Enh;E073,H3K4me1_Enh;E074,H3K4me1_Enh;E075,H3K4me1_Enh;E077,H3K4me1_Enh;E078,H3K4me1_Enh;E079,H3K4me1_Enh;E080,H3K4me1_Enh;E081,H3K4me1_Enh;E082,H3K4me1_Enh;E083,H3K4me1_Enh;E084,H3K4me1_Enh;E085,H3K4me1_Enh;E088,H3K4me1_Enh;E089,H3K4me1_Enh;E090,H3K4me1_Enh;E091,H3K4me1_Enh;E092,H3K4me1_Enh;E093,H3K4me1_Enh;E094,H3K4me1_Enh;E098,H3K4me1_Enh;E099,H3K4me1_Enh;E101,H3K4me1_Enh;E102,H3K4me1_Enh;E103,H3K4me1_Enh;E105,H3K4me1_Enh;E106,H3K4me1_Enh;E107,H3K4me1_Enh;E108,H3K4me1_Enh;E109,H3K4me1_Enh;E110,H3K4me1_Enh;E111,H3K4me1_Enh;E112,H3K4me1_Enh;E117,H3K4me1_Enh;E118,H3K4me1_Enh;E121,H3K4me1_Enh;E123,H3K4me1_Enh;E124,H3K4me1_Enh;E125,H3K4me1_Enh;E127,H3K4me1_Enh;E005,H3K4me3_Pro;E020,H3K4me3_ProE022,H3K4me3_Pro;E038,H3K4me3_Pro;E039,H3K4me3_Pro;E042,H3K4me3_Pro;E044,H3K4me3_Pro;E047,H3K4me3_Pro;E051,H3K4me3_Pro;E055,H3K4me3_Pro;E056,H3K4me3_Pro;059,H3K4me3_Pro;E069,H3K4me3_Pro;E070,H3K4me3_Pro;E076,H3K4me3_Pro;E077,H3K4me3_Pro;E078,H3K4me3_Pro;E082,H3K4me3_Pro;E088,H3K4me3_Pro;E101,H3K4me3_Pro;103,H3K4me3_Pro;E105,H3K4me3_Pro;E107,H3K4me3_Pro;E109,H3K4me3_Pro;E117,H3K4me3_Pro;E006,H3K9ac_Pro;E014,H3K9ac_Pro;E017,H3K9ac_Pro;E027,H3K9ac_Pro;E062,H3K	E003;E004E005;E006;E007;E008;E017;E021;E022;E028;E029E032;E033;E034;E046;E050;E051;E055;E056;E057E059;E080;E081;E082;E083;E084;E085;E086;E088;E089;E090;E091;E092;E093;E094;E097;E098;E100E109;E114;E116;E117;E118;E119;E120;E121;E122E123;E12	A549,CTCF,HudsonAlpha,DEX_100nM;A549,CTCF,HudsonAlpha,EtOH_0.02pct;AG04449,CTCF,UW,None;AG04450,CTCF,UW,None;AG09309,CTCF,UW,None;AG09319,CTCF,UW,None;AG10803,CTCF,UW,None;AoAF,CTCF,UW,None;BJ,CTCF,UW,None;Caco-2,CTCF,UW,None;Fi	.	Sin3Ak-20_disc7	ENSG00000254827.1	SLC22A18AS	NM_007105	SLC22A18AS	Nonsynonymous	SKIN, CRVX
**rs79478137**	WHRadjBMI (Women)	11	2891739	1	1	**rs79478137**	C	T	0.1	0.02	0.01	0.01	E001,7_Enh;E002,7_Enh;E003,7_Enh;E004,7_Enh;E006,7_Enh;E007,7_Enh;E009,7_Enh;E010,7_Enh;E011,7_Enh;E012,7_Enh;E013,7_Enh;E014,7_Enh;E015,7_Enh;E016,7_Enh;E017,7_Enh;E018,1_TssA;E019,7_Enh;E020,7_Enh;E022,7_Enh;E024,7_Enh;E025,7_Enh;E027,7_Enh;E028,7_Enh;E031,7_Enh;E032,7_Enh;E033,7_Enh;E034,7_Enh;E035,7_Enh;E036,7_Enh;E037,7_Enh;E038,7_Enh;E039,2_TssAFlnk;E040,7_Enh;E041,7_Enh;E042,7_Enh;E043,7_Enh;E044,7_Enh;E045,7_Enh;E046,7_Enh;E047,2_TssAFlnk;E048,7_Enh;E050,7_Enh;E051,7_Enh;E053,7_Enh;E055,7_Enh;E056,7_Enh;E057,7_Enh;E058,7_Enh;E059,7_Enh;E061,7_Enh;E062,7_Enh;E063,7_Enh;E066,7_Enh;E070,7_Enh;E073,7_Enh;E075,2_TssAFlnk;E076,7_Enh;E077,7_Enh;E078,7_Enh;E079,7_Enh;E080,7_Enh;E081,7_Enh;E083,7_Enh;E084,7_Enh;E085,7_Enh;E088,7_Enh;E089,7_Enh;E090,7_Enh;	E001,14_EnhA2;E002,17_EnhW2;E003,15_EnhAF;E004,17_EnhW2;E005,17_EnhW2;E006,14_EnhA2;E007,14_EnhA2;E008,17_EnhW2;E009,15_EnhAF;E010,13_EnhA1;E011,17_EnhW2;E012,14_EnhA2;E013,15_EnhAF;E014,17_EnhW2;E015,17_EnhW2;E016,17_EnhW2;E017,14_EnhA2;E018,16_EnhW1;E019,16_EnhW1;E020,16_EnhW1;E021,17_EnhW2;E022,15_EnhAF;E023,17_EnhW2;E024,15_EnhAF;E025,16_EnhW1;E026,17_EnhW2;E027,13_EnhA1;E028,17_EnhW2;E029,15_EnhAF;E030,16_EnhW1;E031,14_EnhA2;E032,14_EnhA2;E033,13_EnhA1;E034,2_PromU;E035,13_EnhA1;E036,13_EnhA1;E037,2_PromU;E038,2_PromU;E039,2_PromU;E040,13_EnhA1;E041,2_PromU;E042,13_EnhA1;E043,2_PromU;E044,13_EnhA1;E045,14_EnhA2;E046,2_PromU;E047,2_PromU;E048,2_PromU;E049,17_EnhW2;E050,13_EnhA1;E051,13_EnhA1;E052,17_EnhW2;E053,15_EnhAF;E054,17_EnhW2;E055,13_EnhA1;E056,13_EnhA1;E057,13_EnhA1;E058,13_EnhA1;E059,15_EnhAF;E061,15_EnhAF;E062,14_EnhA2;E063,14_EnhA2;E065,17_EnhW2;E066,14_EnhA2;E067,17_EnhW2;E068,17_EnhW2;E069,17_EnhW2;E070,15_EnhAF;E071,17_EnhW2;E072,17_EnhW2;E073,17_EnhW2;E074,17_EnhW2;E075,13_EnhA1;E076,14_EnhA2;E077,13_EnhA1;E078,14_EnhA2;E079,17_EnhW2;E080,13_EnhA1;E081,13_EnhA1;E082,15_EnhAF;E083,13_EnhA1;E084,15_EnhAF;E085,13_EnhA1;E086,17_EnhW2;E087,17_EnhW2;E088,13_EnhA1;E089,13_EnhA1;E090,13_EnhA1;E091,13_EnhA1;E092,13_EnhA1;E093,2_PromU;E094,15_EnhAF;E095,17_EnhW2;E096,17_EnhW2;E097,15_EnhAF;E098,13_EnhA1;E099,14_EnhA2;E100,17_EnhW2;E101,17_EnhW2;E102,14_EnhA2;E103,15_EnhAF;E104,17_EnhW2;E105,14_EnhA2;E106,17_	E001,H3K4me1_Enh;E002,H3K4me1_Enh;E003,H3K4me1_Enh;E005,H3K4me1_Enh;E006,H3K4me1_Enh;E007,H3K4me1_Enh;E009,H3K4me1_Enh;E010,H3K4me1_Enh;E011,H3K4me1_Enh;E012,H3K4me1_Enh;E013,H3K4me1_Enh;E014,H3K4me1_Enh;E015,H3K4me1_Enh;E016,H3K4me1_Enh;E017,H3K4me1_Enh;E018,H3K4me1_Enh;E019,H3K4me1_Enh;E020,H3K4me1_Enh;E021,H3K4me1_Enh;E022,H3K4me1_Enh;E023,H3K4me1_Enh;E024,H3K4me1_Enh;E025,H3K4me1_Enh;E026,H3K4me1_Enh;E027,H3K4me1_Enh;E028,H3K4me1_Enh;E029,H3K4me1_Enh;E030,H3K4me1_Enh;E031,H3K4me1_Enh;E032,H3K4me1_Enh;E033,H3K4me1_Enh;E034,H3K4me1_Enh;E035,H3K4me1_Enh;E036,H3K4me1_Enh;E037,H3K4me1_Enh;E038,H3K4me1_Enh;E039,H3K4me1_Enh;E040,H3K4me1_Enh;E041,H3K4me1_Enh;E042,H3K4me1_Enh;E043,H3K4me1_Enh;E044,H3K4me1_Enh;E045,H3K4me1_Enh;E046,H3K4me1_Enh;E047,H3K4me1_Enh;E048,H3K4me1_Enh;E049,H3K4me1_Enh;E050,H3K4me1_Enh;E051,H3K4me1_Enh;E052,H3K4me1_Enh;E053,H3K4me1_Enh;E054,H3K4me1_Enh;E055,H3K4me1_Enh;E056,H3K4me1_Enh;E057,H3K4me1_Enh;E058,H3K4me1_Enh;E059,H3K4me1_Enh;E061,H3K4me1_Enh;E062,H3K4me1_Enh;E063,H3K4me1_Enh;E066,H3K4me1_Enh;E067,H3K4me1_Enh;E069,H3K4me1_Enh;E070,H3K4me1_Enh;E071,H3K4me1_Enh;E073,H3K4me1_Enh;E074,H3K4me1_Enh;E075,H3K4me1_Enh;E076,H3K4me1_Enh;E077,H3K4me1_Enh;E078,H3K4me1_Enh;E079,H3K4me1_Enh;E080,H3K4me1_Enh;E081,H3K4me1_Enh;E082,H3K4me1_Enh;E083,H3K4me1_Enh;E084,H3K4me1_Enh;E085,H3K4me1_Enh;E088,H3K4me1_Enh;E089,H3K4me1_Enh;E090,H3K4me1_Enh;E091,H3K4me1_Enh;E092,H3K4me1_Enh;E093,H3K4me1_Enh;E094,H3K4me1_Enh;E095,H3K4me1_Enh;E096,H3K4me1_Enh;E097,H3K4me1_Enh;E098,H3K4me1_Enh;E099,H3K4me1_Enh;E100,H3K4me1_Enh;E101,H3K4me1_Enh;E102,H3K4me1_Enh;E103,H3K4me1_Enh;E104,H3K4me1_Enh;E105,H3K4me1_Enh;E106,H3K4me1_Enh;E107,H3K4me1_Enh;E108,H3K4me1_Enh;E110,H3K4me1_Enh;E111,H3K4me1_Enh;E112,H3K4me1_Enh;E113,H3K4me1_Enh;E114,H3K4me1_Enh;E115,H3K4me1_Enh;E116,H3K4me1_Enh;E117,H3K4me1_Enh;E118,H3K4me1_Enh;E119,H3K4me1_Enh;E120,H3K4me1_Enh;E121,H3K4me1_Enh;E122,H3K4me1_Enh;E123,H3K4me1_Enh;E124,H3K4me1_Enh;E125,H3K4me1_Enh;E126,H3K4me1_Enh;E127,H3K4me1_Enh;E128,H3K4me1_Enh;E129,H3K4me1_Enh;E001,H3K9	E007;E051E089;E092;E093;E097;E100	.	.	.	ENSG00000254827.1	SLC22A18AS	NM_007105	SLC22A18AS	Intronic	IPSC, BLD, GI, CRVX
rs79478137	WHRadjBMI (Women)	11	2892257	1	1	rs114179983	A	T	0.09	0.02	0	0.0013	E001,1_TssA;E003,7_Enh;E005,7_Enh;E006,2_TssAFlnk;E007,7_Enh;E008,1_TssA;E009,2_TssAFlnk;E010,1_TssA;E012,2_TssAFlnk;E013,7_Enh;E014,2_TssAFlnk;E015,1_TssA;E016,1_TssA;E017,2_TssAFlnk;E018,1_TssA;E019,2_TssAFlnk;E020,7_Enh;E022,7_Enh;E023,2_TssAFlnk;E024,7_Enh;E025,1_TssA;E026,2_TssAFlnk;E027,2_TssAFlnk;E028,7_Enh;E029,2_TssAFlnk;E030,2_TssAFlnk;E031,2_TssAFlnk;E032,2_TssAFlnk;E033,7_Enh;E034,2_TssAFlnk;E035,2_TssAFlnk;E036,2_TssAFlnk;E037,2_TssAFlnk;E038,2_TssAFlnk;E039,2_TssAFlnk;E040,2_TssAFlnk;E041,2_TssAFlnk;E042,2_TssAFlnk;E043,2_TssAFlnk;E044,2_TssAFlnk;E045,2_TssAFlnk;E046,2_TssAFlnk;E047,2_TssAFlnk;E048,2_TssAFlnk;E049,2_TssAFlnk;E050,7_Enh;E051,2_TssAFlnk;E052,2_TssAFlnk;E053,2_TssAFlnk;E054,2_TssAFlnk;E055,2_TssAFlnk;E056,2_TssAFlnk;E057,2_TssAFlnk;E058,2_TssAFlnk;E059,	E001,2_PromU;E002,2_PromU;E003,2_PromU;E004,2_PromU;E005,2_PromU;E006,2_PromU;E007,2_PromU;E008,2_PromU;E009,2_PromU;E010,2_PromU;E011,2_PromU;E012,2_PromU;E013,2_PromU;E014,2_PromU;E015,2_PromU;E016,2_PromU;E017,2_PromU;E018,2_PromU;E019,2_PromU;E020,2_PromU;E021,2_PromU;E022,2_PromU;E023,2_PromU;E024,2_PromU;E025,2_PromU;E026,2_PromU;E027,2_PromU;E028,2_PromU;E029,2_PromU;E030,2_PromU;E031,2_PromU;E032,2_PromU;E033,2_PromU;E034,2_PromU;E035,2_PromU;E036,2_PromU;E037,2_PromU;E038,2_PromU;E039,2_PromU;E040,2_PromU;E041,2_PromU;E042,2_PromU;E043,2_PromU;E044,2_PromU;E045,2_PromU;E046,2_PromU;E047,2_PromU;E048,2_PromU;E049,2_PromU;E050,2_PromU;E051,2_PromU;E052,2_PromU;E053,2_PromU;E054,2_PromU;E055,2_PromU;E056,2_PromU;E057,2_PromU;E058,2_PromU;E059,2_PromU;E061,2_PromU;E062,2_PromU;E063,2_PromU;E065,2_PromU;E066,2_PromU;E067,2_PromU;E068,2_PromU;E069,2_PromU;E070,2_PromU;E071,2_PromU;E072,2_PromU;E073,2_PromU;E074,2_PromU;E075,2_PromU;E076,2_PromU;E077,2_PromU;E078,2_PromU;E079,2_PromU;E080,2_PromU;E081,2_PromU;E082,2_PromU;E083,2_PromU;E084,2_PromU;E085,2_PromU;E086,2_PromU;E087,2_PromU;E088,2_PromU;E089,2_PromU;E090,2_PromU;E091,2_PromU;E092,2_PromU;E093,2_PromU;E094,2_PromU;E095,2_PromU;E096,2_PromU;E097,2_PromU;E098,2_PromU;E099,2_PromU;E100,2_PromU;E101,2_PromU;E102,2_PromU;E103,2_PromU;E104,2_PromU;E105,2_PromU;E106,2_PromU;E107,2_PromU;E108,2_PromU;E109,2_PromU;E110,2_PromU;E111,2_PromU;E112,2_PromU;E113,2_P	E001,H3K4me1_Enh;E003,H3K4me1_Enh;E005,H3K4me1_Enh;E006,H3K4me1_Enh;E007,H3K4me1_Enh;E009,H3K4me1_Enh;E010,H3K4me1_Enh;E011,H3K4me1_Enh;E012,H3K4me1_Enh;E013,H3K4me1_Enh;E014,H3K4me1_Enh;E015,H3K4me1_Enh;E016,H3K4me1_Enh;E017,H3K4me1_Enh;E018,H3K4me1_Enh;E019,H3K4me1_Enh;E020,H3K4me1_Enh;E021,H3K4me1_Enh;E022,H3K4me1_Enh;E023,H3K4me1_Enh;E024,H3K4me1_Enh;E025,H3K4me1_Enh;E026,H3K4me1_Enh;E027,H3K4me1_Enh;E028,H3K4me1_Enh;E029,H3K4me1_Enh;E030,H3K4me1_Enh;E031,H3K4me1_Enh;E032,H3K4me1_Enh;E033,H3K4me1_Enh;E034,H3K4me1_Enh;E035,H3K4me1_Enh;E036,H3K4me1_Enh;E037,H3K4me1_Enh;E038,H3K4me1_Enh;E039,H3K4me1_Enh;E040,H3K4me1_Enh;E041,H3K4me1_Enh;E042,H3K4me1_Enh;E043,H3K4me1_Enh;E044,H3K4me1_Enh;E045,H3K4me1_Enh;E046,H3K4me1_Enh;E047,H3K4me1_Enh;E048,H3K4me1_Enh;E049,H3K4me1_Enh;E050,H3K4me1_Enh;E051,H3K4me1_Enh;E052,H3K4me1_Enh;E053,H3K4me1_Enh;E054,H3K4me1_Enh;E055,H3K4me1_Enh;E056,H3K4me1_Enh;E057,H3K4me1_Enh;E058,H3K4me1_Enh;E059,H3K4me1_Enh;E061,H3K4me1_Enh;E062,H3K4me1_Enh;E063,H3K4me1_Enh;E066,H3K4me1_Enh;E067,H3K4me1_Enh;E068,H3K4me1_Enh;E069,H3K4me1_Enh;E070,H3K4me1_Enh;E071,H3K4me1_Enh;E072,H3K4me1_Enh;E073,H3K4me1_Enh;E074,H3K4me1_Enh;E075,H3K4me1_Enh;E076,H3K4me1_Enh;E077,H3K4me1_Enh;E078,H3K4me1_Enh;E079,H3K4me1_Enh;E080,H3K4me1_Enh;E081,H3K4me1_Enh;E082,H3K4me1_Enh;E083,H3K4me1_Enh;E084,H3K4me1_Enh;E085,H3K4me1_Enh;E088,H3K4me1_Enh;E089,H3K4me1_Enh;E090,H3K4me1_Enh;E091,H3K4me1_Enh;E092,H3K4me1_Enh;E093,H3K4me1_Enh;E094,H3K4me1_Enh;E095,H3K4me1_Enh;E096,H3K4me1_Enh;E098,H3K4me1_Enh;E099,H3K4me1_Enh;E100,H3K4me1_Enh;E101,H3K4me1_Enh;E102,H3K4me1_Enh;E103,H3K4me1_Enh;E104,H3K4me1_Enh;E105,H3K4me1_Enh;E106,H3K4me1_Enh;E107,H3K4me1_Enh;E108,H3K4me1_Enh;E110,H3K4me1_Enh;E111,H3K4me1_Enh;E112,H3K4me1_Enh;E113,H3K4me1_Enh;E114,H3K4me1_Enh;E115,H3K4me1_Enh;E116,H3K4me1_Enh;E117,H3K4me1_Enh;E118,H3K4me1_Enh;E119,H3K4me1_Enh;E120,H3K4me1_Enh;E121,H3K4me1_Enh;E122,H3K4me1_Enh;E123,H3K4me1_Enh;E124,H3K4me1_Enh;E125,H3K4me1_Enh;E126,H3K4me1_Enh;E127,H3K4me1_Enh;E128,H3K4me1_Enh;E129,H3K4me1_Enh;E001,H3K4	E003;E004E005;E006;E007;E008;E017;E021;E022;E028;E029E032;E033;E034;E046;E050;E051;E055;E056;E057E059;E080;E081;E082;E083;E085;E086;E088;E089;E090;E091;E092;E093;E094;E097;E100;E109;E114;E116;E117;E118;E119;E121;E122;E123;E124;E127	A549,CTCF,HudsonAlpha,DEX_100nM;A549,CTCF,HudsonAlpha,EtOH_0.02pct;AG04449,CTCF,UW,None;AG04450,CTCF,UW,None;AG09309,CTCF,UW,None;AG09319,CTCF,UW,None;AG10803,CTCF,UW,None;AoAF,CTCF,UW,None;BJ,CTCF,UW,None;Caco-2,CTCF,UW,None;Fi	.	HDAC2_disc3	ENSG00000254827.1	SLC22A18AS	NM_007105	SLC22A18AS	Intronic	ESC, ESDR, LNG, IPSC, FAT, STRM, BRST, BLD, MUS, BRN, SKIN, GI, ADRL, PLCNT, THYM, HRT, OVRY, CRVX, LIV, VAS, BONE
rs79478137	WHRadjBMI (Women)	11	2894144	1	1	rs12282506	G	A	0.11	0.02	0	0.0013	E018,10_TssBiv	E007,19_DNase;E117,19_DNase	E006,H3K4me3_Pro;E007,H3K4me3_Pro;E008,H3K4me3_Pro;E009,H3K4me3_Pro;E018,H3K4me3_Pro;E019,H3K4me3_Pro;E028,H3K4me3_Pro;E037,H3K4me3_Pro;E047,H3K4me3_Pro;E056,H3K4me3_Pro;E057,H3K4me3_Pro;E059,H3K4me3_Pro;E075,H3K4me3_Pro;E096,H3K4me3_Pro;E103,H3K4me3_Pro;E113,H3K4me3_Pro;E117,H3K4me3_Pro;E006,H3K9ac_Pro;E007,H3K9ac_Pro;E049,H3K9ac_Pro;E062,H3K9ac_Pro;E069,H3K9ac_Pro;E075,H3K9ac_Pro;E083,H3K9ac_Pro;E101,H3K9ac_Pro;E103,H3K9ac_Pro;E117,H3K9ac_Pro;E014,H3K4me1_Enh;E022,H3K4me1_Enh;E024,H3K4me1_Enh;E034,H3K4me1_Enh;E036,H3K4me1_Enh;E041,H3K4me1_Enh;E042,H3K4me1_Enh;E045,H3K4me1_Enh;E046,H3K4me1_Enh;E047,H3K4me1_Enh;E056,H3K4me1_Enh;E059,H3K4me1_Enh;E075,H3K4me1_Enh;E077,H3K4me1_Enh;E089,H3K4me1_Enh;E101,H3K4me1_Enh;E105,H3K4me1_Enh;E110,H3K4me1_Enh;E115,H3K4me1_Enh;E117,H3K4me1_Enh;E029,H3K27ac_Enh;E034,H3K27ac_Enh;E039,H3K27ac_Enh;E041,H3K27ac_Enh;E043,H3K27ac_Enh;E044,H3K27ac_Enh;E045,H3K27ac_Enh;E050,H3K27ac_Enh;E056,H3K27ac_Enh;E059,H3K27ac_Enh;E073,H3K27ac_Enh;E074,H3K27ac_Enh;E093,H3K27ac_Enh;E111,H3K27ac_Enh;E112,H3K27ac_Enh;E117,H3K27ac_Enh;E124,H3K27ac_Enh	E007;E093E097;E114	.	.	.	ENSG00000254827.1	SLC22A18AS	NM_007105	SLC22A18AS	Intronic	IPSC

rs79478137	WHRadjBMI (Women)	11	2894333	0.8	1	rs114826751	G	C	0.1	0.03	0	0.004	E090,7_Enh		E007,H3K4me3_Pro;E009,H3K4me3_Pro;E019,H3K4me3_Pro;E028,H3K4me3_Pro;E059,H3K4me3_Pro;E096,H3K4me3_Pro;E103,H3K4me3_Pro;E113,H3K4me3_Pro;E117,H3K4me3_Pro;E024,H3K4me1_Enh;E034,H3K4me1_Enh;E036,H3K4me1_Enh;E042,H3K4me1_Enh;E046,H3K4me1_Enh;E047,H3K4me1_Enh;E055,H3K4me1_Enh;E056,H3K4me1_Enh;E059,H3K4me1_Enh;E075,H3K4me1_Enh;E077,H3K4me1_Enh;E089,H3K4me1_Enh;E105,H3K4me1_Enh;E110,H3K4me1_Enh;E117,H3K4me1_Enh;E029,H3K27ac_Enh;E034,H3K27ac_Enh;E039,H3K27ac_Enh;E041,H3K27ac_Enh;E043,H3K27ac_Enh;E044,H3K27ac_Enh;E045,H3K27ac_Enh;E056,H3K27ac_Enh;E061,H3K27ac_Enh;E073,H3K27ac_Enh;E093,H3K27ac_Enh;E111,H3K27ac_Enh;E112,H3K27ac_Enh;E124,H3K27ac_Enh;E049,H3K9ac_Pro;E062,H3K9ac_Pro;E075,H3K9ac_Pro;E083,H3K9ac_Pro;E103,H3K9ac_Pro;E117,H3K9ac_Pro		.	.	EBF_disc2;RXRA_known6	ENSG00000254827.1	SLC22A18AS	NM_007105	SLC22A18AS	Intronic	
rs79478137	WHRadjBMI (Women)	11	2894922	1	1	rs12269740	T	G	0.09	0.02	0	0.0013	E089,12_EnhBiv;E090,7_Enh		E006,H3K4me1_Enh;E034,H3K4me1_Enh;E047,H3K4me1_Enh;E055,H3K4me1_Enh;E056,H3K4me1_Enh;E059,H3K4me1_Enh;E077,H3K4me1_Enh;E089,H3K4me1_Enh;E105,H3K4me1_Enh;E117,H3K4me1_Enh;E007,H3K4me3_Pro;E009,H3K4me3_Pro;E103,H3K4me3_Pro;E113,H3K4me3_Pro;E117,H3K4me3_Pro;E007,H3K9ac_Pro;E017,H3K9ac_Pro;E049,H3K9ac_Pro;E062,H3K9ac_Pro;E075,H3K9ac_Pro;E083,H3K9ac_Pro;E103,H3K9ac_Pro;E117,H3K9ac_Pro;E124,H3K9ac_Pro;E029,H3K27ac_Enh;E034,H3K27ac_Enh;E039,H3K27ac_Enh;E041,H3K27ac_Enh;E043,H3K27ac_Enh;E044,H3K27ac_Enh;E045,H3K27ac_Enh;E056,H3K27ac_Enh;E061,H3K27ac_Enh;E073,H3K27ac_Enh;E093,H3K27ac_Enh;E124,H3K27ac_Enh	E032;E034;E088;E092	.	.	PLAG1	ENSG00000254827.1	SLC22A18AS	NM_007105	SLC22A18AS	Intronic	
rs79478137	WHRadjBMI (Women)	11	2896281	1	1	rs12276467	G	A	0.09	0.02	0	0.0013	E059,6_EnhG	E056,17_EnhW2;E059,17_EnhW2	E006,H3K4me1_Enh;E029,H3K4me1_Enh;E034,H3K4me1_Enh;E039,H3K4me1_Enh;E040,H3K4me1_Enh;E042,H3K4me1_Enh;E055,H3K4me1_Enh;E056,H3K4me1_Enh;E059,H3K4me1_Enh;E061,H3K4me1_Enh;E075,H3K4me1_Enh;E077,H3K4me1_Enh;E089,H3K4me1_Enh;E094,H3K4me1_Enh;E096,H3K4me1_Enh;E098,H3K4me1_Enh;E105,H3K4me1_Enh;E113,H3K4me1_Enh;E117,H3K4me1_Enh;E124,H3K4me1_Enh;E007,H3K9ac_Pro;E063,H3K9ac_Pro;E075,H3K9ac_Pro;E083,H3K9ac_Pro;E117,H3K9ac_Pro;E124,H3K9ac_Pro;E041,H3K27ac_Enh;E045,H3K27ac_Enh;E055,H3K27ac_Enh;E056,H3K27ac_Enh;E059,H3K27ac_Enh;E061,H3K27ac_Enh;E117,H3K27ac_Enh;E055,H3K4me3_Pro;E056,H3K4me3_Pro;E059,H3K4me3_Pro;E111,H3K4me3_Pro	E100	.	.	THAP1_disc1	ENSG00000254827.1	SLC22A18AS	NM_007105	SLC22A18AS	Intronic	
rs79478137	WHRadjBMI (Women)	11	2896432	1	1	rs12290113	C	T	0.09	0.02	0	0.0013	E056,12_EnhBiv;E059,7_Enh;E089,12_EnhBiv	E056,17_EnhW2;E059,17_EnhW2	E006,H3K4me1_Enh;E029,H3K4me1_Enh;E034,H3K4me1_Enh;E036,H3K4me1_Enh;E039,H3K4me1_Enh;E040,H3K4me1_Enh;E042,H3K4me1_Enh;E055,H3K4me1_Enh;E056,H3K4me1_Enh;E059,H3K4me1_Enh;E061,H3K4me1_Enh;E075,H3K4me1_Enh;E077,H3K4me1_Enh;E089,H3K4me1_Enh;E094,H3K4me1_Enh;E096,H3K4me1_Enh;E098,H3K4me1_Enh;E105,H3K4me1_Enh;E113,H3K4me1_Enh;E117,H3K4me1_Enh;E123,H3K4me1_Enh;E124,H3K4me1_Enh;E007,H3K9ac_Pro;E063,H3K9ac_Pro;E083,H3K9ac_Pro;E107,H3K9ac_Pro;E117,H3K9ac_Pro;E124,H3K9ac_Pro;E041,H3K27ac_Enh;E044,H3K27ac_Enh;E045,H3K27ac_Enh;E055,H3K27ac_Enh;E056,H3K27ac_Enh;E059,H3K27ac_Enh;E061,H3K27ac_Enh;E117,H3K27ac_Enh;E055,H3K4me3_Pro;E056,H3K4me3_Pro;E059,H3K4me3_Pro;E111,H3K4me3_Pro	E097;E100	.	.	EWSR1-FLI1;Sox_17;Znf143_known1	ENSG00000254827.1	SLC22A18AS	NM_007105	SLC22A18AS	Intronic	
rs3168072	WCadjBMI (Combined)	11	61860488	0.92	0.96	rs74771917	C	T	0.06	0.32	0.2	0.04	E027,7_Enh;E056,3_TxFlnk;E057,6_EnhG;E063,6_EnhG;E072,6_EnhG;E074,6_EnhG;E075,7_Enh;E083,7_Enh;E089,6_EnhG;E090,6_EnhG;E095,7_Enh;E098,7_Enh;E104,7_Enh;E105,7_Enh;E107,7_Enh;E108,7_Enh;E110,7_Enh;E113,6_EnhG;E117,7_Enh;E123,2_TssAFlnk;E127,6_EnhG	E027,11_TxEnh3;E055,11_TxEnh3;E056,11_TxEnh3;E057,11_TxEnh3;E058,11_TxEnh3;E059,11_TxEnh3;E061,11_TxEnh3;E065,19_DNase;E068,11_TxEnh3;E069,11_TxEnh3;E071,11_TxEnh3;E072,11_TxEnh3;E073,11_TxEnh3;E074,11_TxEnh3;E076,11_TxEnh3;E078,11_TxEnh3;E079,11_TxEnh3;E083,13_EnhA1;E089,11_TxEnh3;E092,11_TxEnh3;E094,11_TxEnh3;E095,11_TxEnh3;E096,11_TxEnh3;E097,11_TxEnh3;E098,11_TxEnh3;E100,11_TxEnh3;E104,11_TxEnh3;E105,11_TxEnh3;E107,11_TxEnh3;E109,11_TxEnh3;E110,11_TxEnh3;E111,11_TxEnh3;E113,11_TxEnh3;E114,11_TxEnh3;E117,11_TxEnh3;E123,13_EnhA1;E127,11_TxEnh3	E005,H3K4me1_Enh;E013,H3K4me1_Enh;E023,H3K4me1_Enh;E025,H3K4me1_Enh;E027,H3K4me1_Enh;E055,H3K4me1_Enh;E056,H3K4me1_Enh;E057,H3K4me1_Enh;E058,H3K4me1_Enh;E063,H3K4me1_Enh;E072,H3K4me1_Enh;E074,H3K4me1_Enh;E075,H3K4me1_Enh;E077,H3K4me1_Enh;E078,H3K4me1_Enh;E079,H3K4me1_Enh;E083,H3K4me1_Enh;E089,H3K4me1_Enh;E090,H3K4me1_Enh;E094,H3K4me1_Enh;E095,H3K4me1_Enh;E097,H3K4me1_Enh;E098,H3K4me1_Enh;E104,H3K4me1_Enh;E105,H3K4me1_Enh;E107,H3K4me1_Enh;E108,H3K4me1_Enh;E110,H3K4me1_Enh;E113,H3K4me1_Enh;E114,H3K4me1_Enh;E117,H3K4me1_Enh;E123,H3K4me1_Enh;E127,H3K4me1_Enh;E005,H3K4me3_Pro;E055,H3K4me3_Pro;E056,H3K4me3_Pro;E059,H3K4me3_Pro;E083,H3K4me3_Pro;E113,H3K4me3_Pro;E123,H3K4me3_Pro;E011,H3K27ac_Enh;E013,H3K27ac_Enh;E037,H3K27ac_Enh;E050,H3K27ac_Enh;E055,H3K27ac_Enh;E056,H3K27ac_Enh;E061,H3K27ac_Enh;E068,H3K27ac_Enh;E069,H3K27ac_Enh;E071,H3K27ac_Enh;E072,H3K27ac_Enh;E073,H3K27ac_Enh;E074,H3K27ac_Enh;E095,H3K27ac_Enh;E104,H3K27ac_Enh;E105,H3K27ac_Enh;E109,H3K27ac_Enh;E117,H3K27ac_Enh;E123,H3K27ac_Enh;E127,H3K27ac_Enh;E023,H3K9ac_Pro;E083,H3K9ac_Pro;E103,H3K9ac_Pro;E117,H3K9ac_Pro;E123,H3K9ac_Pro	E004;E022;E055;E056;E057;E083;E088;E089;E090;E093;E094;E097;E098;E100;E109;E114;E117;E123;E125;E126;E127;E128	K562,ELF1,HudsonAlpha,None	GTEx2015_v6,Artery_Tibial,INCENP,1.19954170149175e-06	BRCA1_known2;EWSR1-FLI1;p300_disc10;p300_disc5	ENSG00000134824.9	FADS2	NM_004265	FADS2	Intronic	BLD
rs3168072	WCadjBMI (Combined)	11	61862661	0.96	1	rs11605884	T	C	0.08	0.33	0.24	0.04	E022,6_EnhG;E055,6_EnhG;E056,3_TxFlnk;E083,7_Enh;E105,7_Enh	E055,11_TxEnh3;E056,11_TxEnh3;E059,11_TxEnh3;E083,11_TxEnh3	E005,H3K4me1_Enh;E006,H3K4me1_Enh;E013,H3K4me1_Enh;E022,H3K4me1_Enh;E024,H3K4me1_Enh;E055,H3K4me1_Enh;E056,H3K4me1_Enh;E057,H3K4me1_Enh;E058,H3K4me1_Enh;E080,H3K4me1_Enh;E083,H3K4me1_Enh;E089,H3K4me1_Enh;E095,H3K4me1_Enh;E104,H3K4me1_Enh;E105,H3K4me1_Enh;E108,H3K4me1_Enh;E113,H3K4me1_Enh;E114,H3K4me1_Enh;E123,H3K4me1_Enh;E005,H3K4me3_Pro;E055,H3K4me3_Pro;E056,H3K4me3_Pro;E059,H3K4me3_Pro;E094,H3K4me3_Pro;E113,H3K4me3_Pro;E007,H3K9ac_Pro;E023,H3K9ac_Pro;E027,H3K9ac_Pro;E069,H3K9ac_Pro;E083,H3K9ac_Pro;E087,H3K9ac_Pro;E107,H3K9ac_Pro;E117,H3K9ac_Pro;E011,H3K27ac_Enh;E013,H3K27ac_Enh;E022,H3K27ac_Enh;E050,H3K27ac_Enh;E055,H3K27ac_Enh;E056,H3K27ac_Enh;E059,H3K27ac_Enh;E061,H3K27ac_Enh;E069,H3K27ac_Enh;E071,H3K27ac_Enh;E072,H3K27ac_Enh;E073,H3K27ac_Enh;E074,H3K27ac_Enh;E095,H3K27ac_Enh;E104,H3K27ac_Enh;E105,H3K27ac_Enh;E115,H3K27ac_Enh;E123,H3K27ac_Enh		.	.	CAC-binding-protein;CCNT2_disc2;HNF4_known1;NF-E2_disc4;RXRA_known6;SRF_disc2;UF1H3BETA;YY1_disc5	ENSG00000134824.9	FADS2	NM_004265	FADS2	Intronic	
**rs3168072**	WCadjBMI (Combined)	11	61864038	1	1	**rs3168072**	A	T	0.01	0.32	0.21	0.04	E022,6_EnhG	E056,11_TxEnh3	E005,H3K4me1_Enh;E006,H3K4me1_Enh;E022,H3K4me1_Enh;E024,H3K4me1_Enh;E055,H3K4me1_Enh;E056,H3K4me1_Enh;E057,H3K4me1_Enh;E058,H3K4me1_Enh;E089,H3K4me1_Enh;E105,H3K4me1_Enh;E113,H3K4me1_Enh;E123,H3K4me1_Enh;E005,H3K4me3_Pro;E055,H3K4me3_Pro;E056,H3K4me3_Pro;E059,H3K4me3_Pro;E098,H3K4me3_Pro;E007,H3K9ac_Pro;E062,H3K9ac_Pro;E072,H3K9ac_Pro;E083,H3K9ac_Pro;E011,H3K27ac_Enh;E022,H3K27ac_Enh;E050,H3K27ac_Enh;E056,H3K27ac_Enh;E059,H3K27ac_Enh;E071,H3K27ac_Enh;E073,H3K27ac_Enh;E074,H3K27ac_Enh;E105,H3K27ac_Enh		.	GTEx2015_v6,Artery_Tibial,INCENP,2.71244316970806e-06	GCM;Maf_known2	ENSG00000134824.9	FADS2	NM_004265	FADS2	Intronic	
rs3168072	WCadjBMI (Combined)	11	61864838	0.91	0.99	rs12577276	A	G	0.2	0.33	0.21	0.04			E002,H3K9ac_Pro;E005,H3K9ac_Pro;E023,H3K9ac_Pro;E062,H3K9ac_Pro;E072,H3K9ac_Pro;E083,H3K9ac_Pro;E005,H3K4me3_Pro;E007,H3K4me3_Pro;E055,H3K4me3_Pro;E056,H3K4me3_Pro;E059,H3K4me3_Pro;E113,H3K4me3_Pro;E007,H3K4me1_Enh;E012,H3K4me1_Enh;E024,H3K4me1_Enh;E036,H3K4me1_Enh;E055,H3K4me1_Enh;E056,H3K4me1_Enh;E057,H3K4me1_Enh;E058,H3K4me1_Enh;E089,H3K4me1_Enh;E011,H3K27ac_Enh;E013,H3K27ac_Enh;E050,H3K27ac_Enh;E055,H3K27ac_Enh;E061,H3K27ac_Enh;E071,H3K27ac_Enh;E073,H3K27ac_Enh;E074,H3K27ac_Enh;E080,H3K27ac_Enh		.	.	EWSR1-FLI1;NRSF_disc4;Pax-5_disc1;VDR_4	ENSG00000134824.9	FADS2	NM_004265	FADS2	Intronic	

rs3168072	WCadjBMI (Combined)	11	61874245	0.9	-0.97	rs7115739	T	G	0.66	0.67	0.76	0.96	E024,7_Enh;E052,6_EnhG;E055,6_EnhG;E056,3_TxFlnk;E058,12_EnhBiv;E063,6_EnhG;E068,7_Enh;E079,7_Enh;E081,7_Enh;E083,7_Enh;E089,6_EnhG;E090,6_EnhG;E091,7_Enh;E095,6_EnhG;E096,6_EnhG;E105,6_EnhG;E107,7_Enh;E108,7_Enh;E114,7_Enh;E117,7_Enh;E120,6_EnhG;E121,6_EnhG;E126,7_Enh	E005,17_EnhW2;E007,17_EnhW2;E022,18_EnhAc;E023,11_TxEnh3;E027,17_EnhW2;E052,11_TxEnh3;E053,17_EnhW2;E055,11_TxEnh3;E056,11_TxEnh3;E057,17_EnhW2;E058,11_TxEnh3;E059,11_TxEnh3;E063,13_EnhA1;E065,18_EnhAc;E066,17_EnhW2;E068,11_TxEnh3;E069,17_EnhW2;E070,17_EnhW2;E071,17_EnhW2;E072,17_EnhW2;E073,11_TxEnh3;E074,11_TxEnh3;E076,17_EnhW2;E078,11_TxEnh3;E079,18_EnhAc;E080,17_EnhW2;E081,18_EnhAc;E083,13_EnhA1;E089,14_EnhA2;E090,14_EnhA2;E091,13_EnhA1;E092,17_EnhW2;E094,18_EnhAc;E095,15_EnhAF;E096,15_EnhAF;E097,18_EnhAc;E098,18_EnhAc;E099,17_EnhW2;E100,15_EnhAF;E104,15_EnhAF;E105,15_EnhAF;E107,13_EnhA1;E108,14_EnhA2;E111,17_EnhW2;E113,18_EnhAc;E115,19_DNase;E117,14_EnhA2;E120,13_EnhA1;E121,13_EnhA1	E001,H3K4me1_Enh;E002,H3K4me1_Enh;E006,H3K4me1_Enh;E008,H3K4me1_Enh;E009,H3K4me1_Enh;E015,H3K4me1_Enh;E018,H3K4me1_Enh;E021,H3K4me1_Enh;E022,H3K4me1_Enh;E024,H3K4me1_Enh;E052,H3K4me1_Enh;E055,H3K4me1_Enh;E056,H3K4me1_Enh;E057,H3K4me1_Enh;E058,H3K4me1_Enh;E059,H3K4me1_Enh;E063,H3K4me1_Enh;E068,H3K4me1_Enh;E073,H3K4me1_Enh;E079,H3K4me1_Enh;E081,H3K4me1_Enh;E083,H3K4me1_Enh;E089,H3K4me1_Enh;E090,H3K4me1_Enh;E091,H3K4me1_Enh;E093,H3K4me1_Enh;E094,H3K4me1_Enh;E095,H3K4me1_Enh;E096,H3K4me1_Enh;E098,H3K4me1_Enh;E104,H3K4me1_Enh;E105,H3K4me1_Enh;E107,H3K4me1_Enh;E108,H3K4me1_Enh;E113,H3K4me1_Enh;E114,H3K4me1_Enh;E117,H3K4me1_Enh;E120,H3K4me1_Enh;E121,H3K4me1_Enh;E125,H3K4me1_Enh;E126,H3K4me1_Enh;E003,H3K9ac_Pro;E007,H3K9ac_Pro;E008,H3K9ac_Pro;E015,H3K9ac_Pro;E023,H3K9ac_Pro;E026,H3K9ac_Pro;E027,H3K9ac_Pro;E038,H3K9ac_Pro;E052,H3K9ac_Pro;E062,H3K9ac_Pro;E063,H3K9ac_Pro;E072,H3K9ac_Pro;E083,H3K9ac_Pro;E107,H3K9ac_Pro;E117,H3K9ac_Pro;E120,H3K9ac_Pro;E121,H3K9ac_Pro;E008,H3K27ac_Enh;E055,H3K27ac_Enh;E056,H3K27ac_Enh;E059,H3K27ac_Enh;E063,H3K27ac_Enh;E065,H3K27ac_Enh;E069,H3K27ac_Enh;E071,H3K27ac_Enh;E073,H3K27ac_Enh;E074,H3K27ac_Enh;E076,H3K27ac_Enh;E089,H3K27ac_Enh;E090,H3K27ac_Enh;E091,H3K27ac_Enh;E095,H3K27ac_Enh;E096,H3K27ac_Enh;E104,H3K27ac_Enh;E105,H3K27ac_Enh;E108,H3K27ac_Enh;E120,H3K27ac_Enh;E121,H3K27ac_Enh;E055,H3K4me3_Pro;E056,H3K4me3_Pro;E059,H3K4me3_Pro;E083,H3K4me3_Pro;E113,H3K4me3_Pro;E117,H3K4me3_Pro	E059;E081;E083;E089;E090;E121;E127	HSMM,CTCF,Broad,None;HSMMtube,CTCF,Broad,None;MCF-7,CTCF,UT-A,None	.	ZID	ENSG00000221968.4	FADS3	NM_021727	FADS3	Intronic	
**rs28692724**	HIPadjBMI (Women)	14	77027445	1	1	**rs28692724**	C	T	0.25	0.32	0.29	0.29	E001,1_TssA;E003,1_TssA;E004,1_TssA;E005,1_TssA;E006,1_TssA;E007,10_TssBiv;E008,1_TssA;E009,1_TssA;E010,1_TssA;E011,1_TssA;E012,10_TssBiv;E013,1_TssA;E014,1_TssA;E015,1_TssA;E016,1_TssA;E017,1_TssA;E018,1_TssA;E019,1_TssA;E020,1_TssA;E021,1_TssA;E022,3_TxFlnk;E023,1_TssA;E024,1_TssA;E025,1_TssA;E026,1_TssA;E027,1_TssA;E028,1_TssA;E029,1_TssA;E030,1_TssA;E031,2_TssAFlnk;E032,1_TssA;E033,1_TssA;E034,1_TssA;E035,2_TssAFlnk;E036,1_TssA;E037,1_TssA;E038,1_TssA;E039,1_TssA;E040,1_TssA;E041,1_TssA;E042,3_TxFlnk;E043,1_TssA;E044,1_TssA;E045,1_TssA;E046,1_TssA;E047,1_TssA;E048,1_TssA;E049,1_TssA;E050,1_TssA;E051,2_TssAFlnk;E052,1_TssA;E053,1_TssA;E054,1_TssA;E055,1_TssA;E056,3_TxFlnk;E057,10_TssBiv;E058,10_TssBiv;E059,1_TssA;E061,1_TssA;E062,1_TssA;E063,1_TssA;E065,1_TssA;E066,1_T	E001,3_PromD1;E002,3_PromD1;E003,3_PromD1;E004,3_PromD1;E005,3_PromD1;E006,3_PromD1;E007,3_PromD1;E008,3_PromD1;E009,3_PromD1;E010,3_PromD1;E011,3_PromD1;E012,3_PromD1;E013,3_PromD1;E014,3_PromD1;E015,3_PromD1;E016,3_PromD1;E017,3_PromD1;E018,3_PromD1;E019,3_PromD1;E020,3_PromD1;E021,3_PromD1;E022,3_PromD1;E023,3_PromD1;E024,3_PromD1;E025,3_PromD1;E026,3_PromD1;E027,3_PromD1;E028,3_PromD1;E029,3_PromD1;E030,3_PromD1;E031,3_PromD1;E032,3_PromD1;E033,3_PromD1;E034,3_PromD1;E035,3_PromD1;E036,3_PromD1;E037,3_PromD1;E038,3_PromD1;E039,3_PromD1;E040,3_PromD1;E041,3_PromD1;E042,3_PromD1;E043,3_PromD1;E044,3_PromD1;E045,3_PromD1;E046,3_PromD1;E047,3_PromD1;E048,3_PromD1;E049,3_PromD1;E050,3_PromD1;E051,3_PromD1;E052,3_PromD1;E053,3_PromD1;E054,3_PromD1;E055,3_PromD1;E056,3_PromD1;E057,3_PromD1;E058,3_PromD1;E059,3_PromD1;E061,3_PromD1;E062,3_PromD1;E063,3_PromD1;E065,3_PromD1;E066,3_PromD1;E067,3_PromD1;E068,3_PromD1;E069,3_PromD1;E070,3_PromD1;E071,3_PromD1;E072,3_PromD1;E073,3_PromD1;E074,3_PromD1;E075,3_PromD1;E076,3_PromD1;E077,3_PromD1;E078,3_PromD1;E079,3_PromD1;E080,3_PromD1;E081,3_PromD1;E082,3_PromD1;E083,3_PromD1;E084,3_PromD1;E085,3_PromD1;E086,3_PromD1;E087,3_PromD1;E088,3_PromD1;E089,3_PromD1;E090,3_PromD1;E091,3_PromD1;E092,3_PromD1;E093,3_PromD1;E094,3_PromD1;E095,3_PromD1;E096,3_PromD1;E097,3_PromD1;E098,3_PromD1;E099,3_PromD1;E100,3_PromD1;E101,3_PromD1;E102,3_PromD1;E103,3_PromD1;E104,3_PromD1;E105,3_P	E001,H3K4me3_Pro;E002,H3K4me3_Pro;E003,H3K4me3_Pro;E004,H3K4me3_Pro;E005,H3K4me3_Pro;E006,H3K4me3_Pro;E007,H3K4me3_Pro;E008,H3K4me3_Pro;E009,H3K4me3_Pro;E010,H3K4me3_Pro;E011,H3K4me3_Pro;E012,H3K4me3_Pro;E013,H3K4me3_Pro;E014,H3K4me3_Pro;E015,H3K4me3_Pro;E016,H3K4me3_Pro;E017,H3K4me3_Pro;E018,H3K4me3_Pro;E019,H3K4me3_Pro;E020,H3K4me3_Pro;E021,H3K4me3_Pro;E022,H3K4me3_Pro;E023,H3K4me3_Pro;E024,H3K4me3_Pro;E025,H3K4me3_Pro;E026,H3K4me3_Pro;E027,H3K4me3_Pro;E028,H3K4me3_Pro;E029,H3K4me3_Pro;E030,H3K4me3_Pro;E031,H3K4me3_Pro;E032,H3K4me3_Pro;E033,H3K4me3_Pro;E034,H3K4me3_Pro;E035,H3K4me3_Pro;E036,H3K4me3_Pro;E037,H3K4me3_Pro;E038,H3K4me3_Pro;E039,H3K4me3_Pro;E040,H3K4me3_Pro;E041,H3K4me3_Pro;E042,H3K4me3_Pro;E043,H3K4me3_Pro;E044,H3K4me3_Pro;E045,H3K4me3_Pro;E046,H3K4me3_Pro;E047,H3K4me3_Pro;E048,H3K4me3_Pro;E049,H3K4me3_Pro;E050,H3K4me3_Pro;E051,H3K4me3_Pro;E052,H3K4me3_Pro;E053,H3K4me3_Pro;E054,H3K4me3_Pro;E055,H3K4me3_Pro;E056,H3K4me3_Pro;E057,H3K4me3_Pro;E058,H3K4me3_Pro;E059,H3K4me3_Pro;E061,H3K4me3_Pro;E062,H3K4me3_Pro;E063,H3K4me3_Pro;E065,H3K4me3_Pro;E066,H3K4me3_Pro;E067,H3K4me3_Pro;E068,H3K4me3_Pro;E069,H3K4me3_Pro;E070,H3K4me3_Pro;E071,H3K4me3_Pro;E072,H3K4me3_Pro;E073,H3K4me3_Pro;E074,H3K4me3_Pro;E075,H3K4me3_Pro;E076,H3K4me3_Pro;E077,H3K4me3_Pro;E078,H3K4me3_Pro;E079,H3K4me3_Pro;E080,H3K4me3_Pro;E082,H3K4me3_Pro;E083,H3K4me3_Pro;E084,H3K4me3_Pro;E085,H3K4me3_Pro;E086,H3K4me3_Pro;E087,H3K4me3_Pro;E088,H3K4me3_Pro;E089,H3K4me3_Pro;E090,H3K4me3_Pro;E091,H3K4me3_Pro;E092,H3K4me3_Pro;E093,H3K4me3_Pro;E094,H3K4me3_Pro;E095,H3K4me3_Pro;E096,H3K4me3_Pro;E097,H3K4me3_Pro;E098,H3K4me3_Pro;E099,H3K4me3_Pro;E100,H3K4me3_Pro;E101,H3K4me3_Pro;E102,H3K4me3_Pro;E103,H3K4me3_Pro;E104,H3K4me3_Pro;E105,H3K4me3_Pro;E106,H3K4me3_Pro;E107,H3K4me3_Pro;E108,H3K4me3_Pro;E109,H3K4me3_Pro;E110,H3K4me3_Pro;E111,H3K4me3_Pro;E112,H3K4me3_Pro;E113,H3K4me3_Pro;E114,H3K4me3_Pro;E115,H3K4me3_Pro;E116,H3K4me3_Pro;E117,H3K4me3_Pro;E118,H3K4me3_Pro;E119,H3K4me3_Pro;E120,H3K4me3_Pro;E121,H3K4me3_Pro;E122,H3K4me3_Pro;E123,H3K4me3_Pro;E124,H3K4me3_Pro;E125,H3K4	E004;E006;E034;E050;E051;E055;E056;E086;E088;E093;E100;E116;E119;E124;E126	Fibrobl,CTCF,UT-A,None	.	Myf_1;NRSF_known1;RFX5_known2	ENSG00000119669.3	IRF2BPL	NM_024496	IRF2BPL	Synonymous	ESC, ESDR, LNG, IPSC, FAT, STRM, BRST, BLD, MUS, BRN, SKIN, VAS, LIV, GI, ADRL, HRT, KID, PANC, PLCNT, THYM, OVRY, SPLN, CRVX, BONE
